# Fungal Biotransformation of 2′-Methylflavanone and 2′-Methylflavone as a Method to Obtain Glycosylated Derivatives

**DOI:** 10.3390/ijms22179617

**Published:** 2021-09-05

**Authors:** Agnieszka Krawczyk-Łebek, Monika Dymarska, Tomasz Janeczko, Edyta Kostrzewa-Susłow

**Affiliations:** Department of Chemistry, Faculty of Biotechnology and Food Science, Wrocław University of Environmental and Life Sciences, 50-375 Wrocław, Poland; monika.dymarska@upwr.edu.pl (M.D.); tomasz.janeczko@upwr.edu.pl (T.J.)

**Keywords:** flavonoids, biotransformations, glycosylation, methylflavanone, methylflavone, *O*-methylglucosides, *Beauveria bassiana*, *Isaria fumosorosea*

## Abstract

Methylated flavonoids are promising pharmaceutical agents due to their improved metabolic stability and increased activity compared to unmethylated forms. The biotransformation in cultures of entomopathogenic filamentous fungi is a valuable method to obtain glycosylated flavones and flavanones with increased aqueous solubility and bioavailability. In the present study, we combined chemical synthesis and biotransformation to obtain methylated and glycosylated flavonoid derivatives. In the first step, we synthesized 2′-methylflavanone and 2′-methylflavone. Afterwards, both compounds were biotransformed in the cultures of two strains of entomopathogenic filamentous fungi *Beauveria bassiana* KCH J1.5 and *Isaria fumosorosea* KCH J2. We determined the structures of biotransformation products based on NMR spectroscopy. Biotransformations of 2′-methyflavanone in the culture of *B. bassiana* KCH J1.5 resulted in three glycosylated flavanones: 2′-methylflavanone 6-*O*-*β*-d-(4″-*O*-methyl)-glucopyranoside, 3′-hydroxy-2′-methylflavanone 6-*O*-*β*-d-(4″-*O*-methyl)-glucopyranoside, and 2-(2′-methylphenyl)-chromane 4-*O*-*β*-d-(4″-*O*-methyl)-glucopyranoside, whereas in the culture of *I. fumosorosea* KCH J2, two other products were obtained: 2′-methylflavanone 3′-*O*-*β*-d-(4″-*O*-methyl)-glucopyranoside and 2-methylbenzoic acid 4-*O*-*β*-d-(4′-*O*-methyl)-glucopyranoside. 2′-Methylflavone was effectively biotransformed only by *I. fumosorosea* KCH J2 into three derivatives: 2′-methylflavone 3′-*O*-*β*-d-(4″-*O*-methyl)-glucopyranoside, 2′-methylflavone 4′-*O*-*β*-d-(4″-*O*-methyl)-glucopyranoside, and 2′-methylflavone 5′-*O*-*β*-d-(4″-*O*-methyl)-glucopyranoside. All obtained glycosylated flavonoids have not been described in the literature until now and need further research on their biological activity and pharmacological efficacy as potential drugs.

## 1. Introduction

**Scheme 1:**
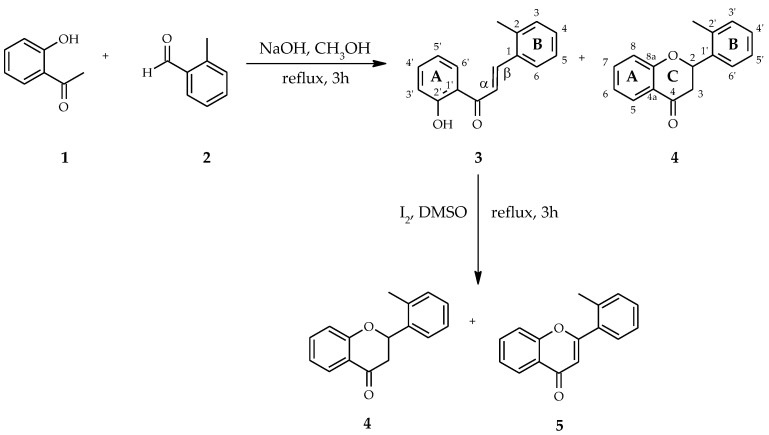
Synthesis of 2′-methylflavanone (**4**) and 2′-methylflavone (**5**).

Flavonoids are polyphenolic secondary metabolites of plants and common components of the human diet. These compounds exert various pharmacological activities, such as antimicrobial, anti-inflammatory, antitumor, antidiabetic, antiallergic, cardio-, and neuroprotective [1,2]. Their basic structure consists of C6–C3–C6 rings, in which two aromatic rings (A and B) are connected by ring C with three carbons and one oxygen heteroatom (Scheme 1). Flavonoids can be categorized into a few subclasses, based on the substitution pattern such as flavanones, flavones, flavonols, and flavanonols [2].

Naturally, flavonoids occur in the form of glycosides with one or more sugar moieties attached to a flavonoid aglycone via *O*- or *C*-glycosidic bonds [3]. In addition, they are usually substituted with hydroxyl, *O*-, and *C*- methyl moieties, which affect their bioactivity and bioavailability [4,5].

Studies comparing methylated versus unmethylated flavones indicate improved intestinal absorption and activity of the first ones [4,6,7,8]. Analyses conducted on the human’s oral SCC-9 cancer cells revealed that *O*-methylated 5,7-dimethoxyflavone and 5,7,4′-trimethoxyflavone were both 10 times more active as inhibitors of cell proliferation with IC_50_ values 5–8 µM, than the corresponding unmethylated analogs chrysin and apigenin. Moreover, 5,7-dimethoxyflavone was far better absorbed than chrysin and had high oral bioavailability and tissue accumulation in vivo during research conducted on rats [8]. However, there are no extensive studies on *C*-methylated flavonoids absorption and comparison of *O*-methylted versus *C*-methylated flavonoids. This situation may be related to the rare occurrence of *C*-methylated flavonoids in nature.

On the other hand, glycosylation of flavonoids strongly modulates their properties by improving aqueous solubility and facilitating intestinal absorption. Glycosylated flavonoids reach higher plasma levels and longer mean residence time in the blood than their aglycone forms. The influence of glycosylation on the biological activity of flavonoids is complicated, depending on the structural differences of the flavonoid core, the position, and the number of the sugar moieties attached. Moreover, the biological activity of flavonoids and their glycosides can differ while observed in vitro and in vivo [3,7,9]. The reason for that phenomenon is that flavonoids undergo extensive metabolism after oral administration, with absorption occurring in the small and the large intestines [9,10,11]. The intestinal microbiota also plays a significant role in their absorption and flavonoids affect gut health [9,10,12,13,14]. Among flavonoid glycosides flavones and flavanones are quite stable during heat processing in contrast to anthocyanins and isoflavones which can be degraded during thermal processing [15].

Many flavonoids exhibit a great biological potential; however, their extraction from natural sources is inefficient. Poor aqueous solubility and bioavailability of flavonoids’ aglycones obtained by synthesis limit their pharmacological application. Chemical glycosylation of flavonoids leads to low isolated yields caused by the need for drastic reaction conditions resulting in the decomposition of reaction substrate and laborious procedures required to achieve regio- and stereoselectivity [3,16,17]. However, biotransformation with entomopathogenic filamentous fungi as biocatalysts can be applied to efficient production of flavonoid’s glycosides [16,17,18,19,20,21,22,23,24,25].

The recent studies revealed a glycosyltransferase–methyltransferase gene pair that encodes a functional module responsible for methylglucosylation in the entomopathogenic filamentous fungi *B. bassiana*. The flavonoid substrates are structurally similar to the benzenediol lactones, which are drug-like fungal secondary metabolites. Thanks to this, they can be accepted as enzymes substrates and successfully glycosylated with 4″-*O*-methylglucose [26].

The presented results continue our previous research on *C*-methylated flavonoids [19,21]. In this study, we synthesized 2′-methylflavanone and 2′-methylflavone, and then biotransformed them in the cultures of entomopathogenic filamentous fungi strains of *B. bassiana* KCH J1.5 and *I. fumosorosea* KCH J2.

Biotransformation of 2′-methylflavanone in the culture of *B. bassiana* KCH J1.5 resulted in three products with glucosyl moieties attached at A and C ring. *I. fumosorosea* KCH J2 biotransformed 2′-methylflavanone into its derivative with glycosidic unit at C-3′ (B ring) and also a product of the C-ring cleavage with methyl-glycosyl moiety attached to the remaining aromatic ring. 2′-Methyflavone was efficiently biotransformed only by *I. fumosorosea* KCH J2 to obtain three glycosylation products with 4″-*O*-methyl-glucosyl moiety attached at positions 3′, 4′, and 5′ of the B ring.

According to our best knowledge, all the above-mentioned biotransformation products have not been described in the literature and can be considered as potentially bioactive compounds with improved bioavailability.

## 2. Results

The main aim of our study was to investigate the ability of entomopathogenic filamentous fungi *B. bassiana* KCH J1.5 and *I. fumosorosea* KCH J2 to glycosylate two flavonoid compounds, i.e., 2′-methylflavanone and 2′-methylflavone. Both strains of microorganisms had been effective in glycosylation described in our previous studies [19,21,27,28]. The first step of our study was a two-step synthesis of substrates for further biotransformation (Scheme 1) (Section 4.1. Substrates).

The biotransformation of 2′-methylflavanone in the cultures of both strains resulted in the formation of five new flavanone derivatives. 2′-Methyflavone was efficiently biotransformed only by *I. fumosorosea* KCH J2 to obtain three new products.

Biotransformations products were isolated and purified through the preparative Thin Layer Chromatography (TLC) method with chloroform and methanol (9:1 *v*/*v*) as eluent. Microbial transformations were conducted on a semi-preparative scale to establish the chemical structures of biotransformation products by Nuclear Magnetic Resonance (NMR) spectroscopy and to determine their isolated yields.

### 2.1. Biotransformations of 2′-Methylflavanone (***4***) in the Culture of B. bassiana KCH J1.5

**Scheme 2:**
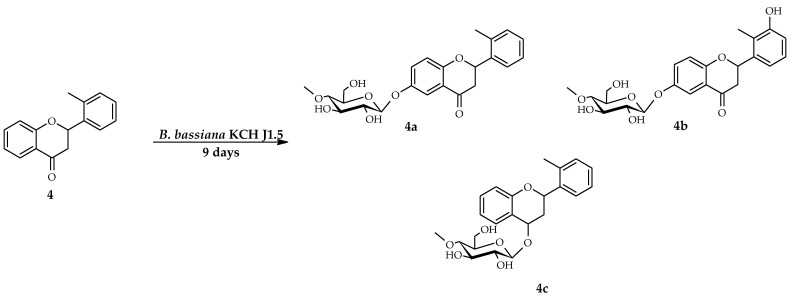
Microbial transformation of 2′-methylflavanone (**4**) in *B. bassiana* KCH J1.5 culture.

As a result of biotransformation of 2′-methylflavanone in the culture of *B. bassiana* KCH J1.5, three products were obtained: 2′-methylflavanone 6-*O*-*β*-d-(4″-*O*-methyl)-glucopyranoside (**4a**) with 8.1% yield (7.3 mg), 3′-hydroxy-2′-methylflavanone 6-*O*-*β*-d-(4″-*O*-methyl)-glucopyranoside (**4b**) with 3.6% yield (3.4 mg), and 2-(2′-methylphenyl)-chromane 4-*O*-*β*-d-(4″-*O*-methyl)-glucopyranoside (**4c**) with a 6.8% yield (5.9 mg) (Scheme 2).

**Scheme 3:**
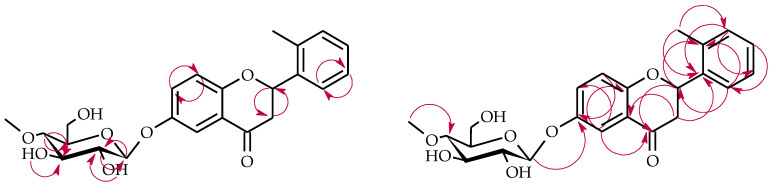
Key COSY (on the left) and HMBC (on the right) correlations for the structure elucidation of product **4a**.

**Scheme 4:**
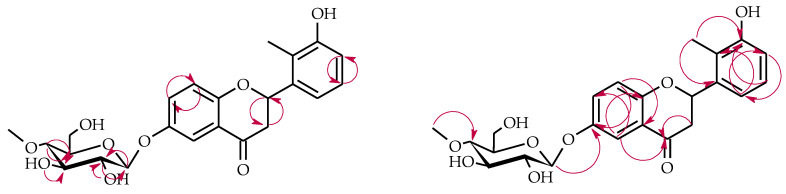
Key COSY (on the left) and HMBC (on the right) correlations for the structure elucidation of product **4b**.

**Scheme 5:**
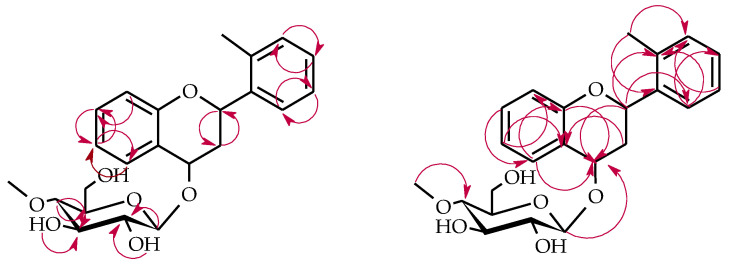
Key COSY (on the left) and HMBC (on the right) correlations for the structure elucidation of product **4c**.

The chemical structures of products **4a**–**4c** were established based on the NMR spectroscopy (Table 1 and Table 2, Scheme 3, Scheme 4 and Scheme 5 showing key COSY (Correlation Spectroscopy) and HMBC (Heteronuclear Multiple Bond Coherence) correlations)). 4″-*O*-methyl-glycosylations were observed in all three products. The presence of a glycosidic moiety in the products **4a**–**4c** was confirmed by five characteristic carbon signals observed in the region from about δ = 80 ppm to about δ = 62 ppm in the Carbon-13 Nuclear Magnetic Resonance (^13^C-NMR) spectra (Supplementary Materials: Figures S23, S43 and S59), as well as H2″–H6″ signals ranging from about δ = 3.8 ppm to about δ = 3.2 ppm in the Proton Nuclear Magnetic Resonance (^1^H-NMR) spectra (Supplementary Materials: Figures S20, S40, and S56). The attachment of a glycosidic unit to substrate **4** was also confirmed by a one-proton doublet from proton at the anomeric carbon atom at δ = 4.88 ppm (**4a** and **4b**) and at δ = 4.63 ppm (**4c**) in the ^1^H-NMR spectra. The coupling constant (*J* = 7.8 Hz) for the anomeric proton was evidence of the glucose *β*-configuration. The sugar unit C-4″ hydroxyl group has been *O*-methylated, because in the ^1^H-NMR spectra a three-proton singlet at δ = 3.57 ppm (**4a** and **4b**) and δ = 3.54 ppm (**4c**) with the corresponding signal at δ = 60.5 ppm (**4a**–**4c**) in the ^13^C-NMR spectra was observed. The C-4″ hydroxyl group methylation was detected based on the HMBC experiment, where the proton signal due to -OCH_3_ was correlated with the signal of C-4″ (about δ = 80 ppm) in the glycosidic unit (Supplementary Materials: Figures S34, S52, and S69). The three-proton singlet of the methyl group at C-2′ (δ = 2.43 ppm in **4a** and **4c**, δ = 2.27 ppm in **4b**) remained present as in the ^1^H-NMR spectrum of substrate **4**, indicating the methyl moiety retained.

In the case of product **4a**, 4″-*O*-methylglucosylation occurred at C-6, because in the HMBC experiment, the proton at the anomeric carbon atom (δ = 4.88 ppm) was coupled with the C-6 signal (δ = 153.2 ppm), which was shifted from δ = 122.3 ppm, indicating an electronegative atom attachment (Supplementary Materials: Figure S35). Moreover, in the ^1^H NMR spectrum the signal from proton at C-6 disappeared, the signals from the protons at C-5 and C-7 became shifted: proton at C-5 from δ = 7.87 ppm (in **4**) to δ = 7.49 ppm (in **4a**) and proton at C-7 from δ = 7.58 ppm (in **4**) to δ = 7.33 ppm (in **4a**) (Supplementary Materials: Figure S19). In the HMBC experiment several couplings were observed that confirmed glycosylation occurred at C-6: between the proton at C-5 (δ = 7.49 ppm), C-7 (δ = 7.33 ppm), C-8 (δ = 7.02 ppm), and the shifted C-6 (δ = 153.2 ppm) (Supplementary Materials: Figure S33) (Scheme 3).

In the case of compound **4b**, 4″-*O*-methylglucosylation occurred at C-6 same as in the product **4a**. However, the appearance of the singlet from proton at δ = 8.32 ppm in the ^1^H NMR spectrum showed that another substitution occurred: attachment of the hydroxyl group at C-3′ of B ring (Supplementary Materials: Figure S38). Moreover, the signal from proton at C-6 disappeared, and the signals from the protons at C-5 and C-7 became shifted: proton at C-5 from δ = 7.87 ppm (in **4**) to δ = 7.48 ppm (in **4b**) and proton at C-7 from δ = 7.58 ppm (in **4**) to δ = 7.33 ppm (in **4b**). The protons from B ring became shifted indicating electronegative atom substitution at C-3′: proton at C-6′ from δ = 7.64 ppm (in **4**) to δ = 7.11 ppm (in **4b**), proton at C-5′ from δ = 7.30 ppm (in **4**) to δ = 7.11 ppm (in **4b**), and proton at C-4′ from δ = 7.27 ppm (in **4**) to δ = 6.89 ppm (in **4b**) (Supplementary Materials: Figure S39). The three-proton singlet of the methyl group at C-2′ became shifted from δ = 2.44 ppm to δ = 2.27 ppm confirming that the attachment of the hydroxyl group occurred at adjacent C-3′ position (Supplementary Materials: Figure S40). In the HMBC experiment, proton at the anomeric carbon (δ = 4.88 ppm) was coupled with the C-6 signal (δ = 153.2 ppm), which was shifted from δ = 122.3 ppm (Supplementary Materials: Figure S52). Moreover, methyl moiety at C-2′ (δ = 2.27 ppm) was coupled with carbon at C-3′, which was shifted from δ = 129.3 ppm to δ = 156.3 ppm (Supplementary Materials: Figure S52) (Scheme 4).

In the case of product **4c**, 4″-*O*-methylglucosylation occurred at C-4, because in the HMBC spectrum, the proton at the anomeric carbon atom (δ = 4.63 ppm) was coupled with the C-4 signal (δ = 69.2 ppm), which was shifted from δ = 192.1 ppm, indicating the reduction of the carbonyl group at C-4 (Supplementary Materials: Figure S69). Furthermore, the signal from one proton at C-2 became shifted from δ = 5.84 ppm (in **4**) to δ = 5.47 ppm (in 4**c**) (Supplementary Materials: Figure S55). The signals from the pseudo-axial (δ = 2.45 ppm) and pseudo-equatorial (δ =1.96 ppm) protons at C-3 were shifted from δ = 3.15 ppm and δ = 2.81 ppm, respectively (Supplementary Materials: Figure S56). In the HMBC experiment, several couplings were observed that confirmed reduction and glycosylation at C-4: between the proton at C-5 (δ = 7.43 ppm) and C-4 (δ = 69.2 ppm), between the pseudo-axial proton at C-3 (δ = 2.45 ppm) and C-4 (Supplementary Materials: Figures S67 and S69) (Scheme 5).

### 2.2. Biotransformations of 2′-Methylflavanone (***4***) in the Culture of I. fumosorosea KCH J2

**Scheme 6:**
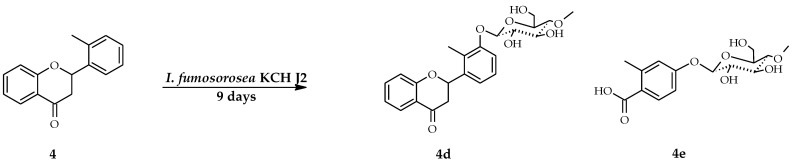
Microbial transformation of 2′-methylflavanone (**4**) in *I. fumosorosea* KCH J2 culture.

2′-Methylflavanone (**4**) was also utilized in microbial transformations performed in the culture of *I. fumosorosea* KCH J2. As the result the following were obtained: 2′-methylflavanone 3′-*O*-*β*-d-(4″-*O*-methyl)-glucopyranoside (**4d**) with a 3.7% yield (3.3 mg) and 2-methylbenzoic acid 4-*O*-*β*-d-(4′-*O*-methyl)-glucopyranoside (**4e**) with a 6.8% yield (4.7 mg) (Scheme 6).

**Scheme 7:**
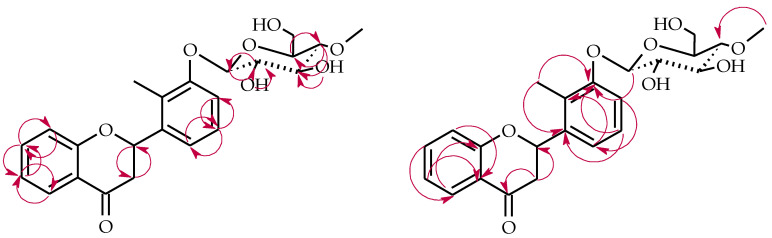
Key COSY (on the left) and HMBC (on the right) correlations for the structure elucidation of product **4d**.

The structure of the product **4d**, 2′-methylflavanone 3′-*O*-*β*-d-(4″-*O*-methyl)-glucopyranoside, was elucidated based on the NMR spectroscopy (Table 1 and Table 2, Scheme 7 showing key COSY and HMBC correlations). The resulting compound was 4″-*O*-methyl-glucosylated like products **4a**–**4c**. In this case, the attachment of the glucosyl moiety occurred at C-3. In the ^1^H NMR spectrum, shifted signals from the B ring were observed: from proton at C-6′ (shifted from δ = 7.64 ppm in **4** to δ = 7.33 ppm in **4d**), proton at C-5′ (shifted from δ = 7.30 ppm in **4** to δ = 7.24 ppm in **4d**), proton at C-4′ (shifted from δ = 7.27 ppm in **4** to δ = 7.18 ppm in **4d**) (Supplementary Materials: Figure S73). The methyl moiety at C-2′ was shifted from δ = 2.44 ppm in **4** to δ = 2.34 ppm in **4d**, indicating the attachment of the electronegative atom at the adjacent position at C-3′ (Supplementary Materials: Figure S74). The substitution with the glucosyl moiety was confirmed in the HMBC experiment by the coupling between the proton at the anomeric carbon (δ = 4.94 ppm) and C-3′ (δ = 156.9 ppm), the coupling between the methyl moiety at C-2′ (δ = 2.34 ppm) and the carbons C-2′ (δ = 126.2 ppm), C-1′ (δ = 139.3 ppm), and shifted C-3′ (δ = 156.9 ppm), and also between the proton at C-5′ (δ = 7.24 ppm) and carbons C-1′ (δ = 139.3 ppm) and C-3′ (δ = 156.9 ppm) (Supplementary Materials: Figures S88 and S86) (Scheme 7).

**Scheme 8:**
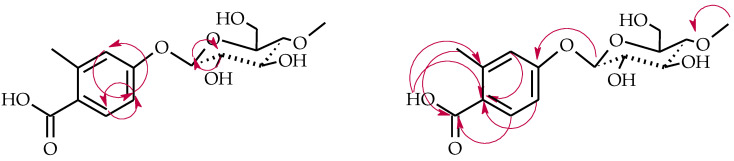
Key COSY (on the left) and HMBC (on the right) correlations for the structure elucidation of product **4e**.

The structure of compound **4e** was also determined with the use of the NMR spectroscopy (Table 1 and Table 2, Scheme 8 showing key COSY and HMBC correlations). In this case, we observed the cleavage of the C ring of substrate **4**. The resulting product was a benzoic acid derivative with the remaining methyl moiety and an attached 4′-*O*-methyl-glucosyl moiety. In the ^1^H NMR spectrum, we observed three signals from protons of the aromatic ring: doublet at C-6 (δ = 7.62 ppm), doublet of doublets at C-5 (δ = 7.29 ppm), and doublet at C-3 (δ = 6.86 ppm) (Supplementary Materials: Figure S91). In the HMBC experiment, we also observed the correlation between three-proton singlet from the methyl moiety at C-2 (δ = 2.65 ppm) and the carbonyl group (δ = 205.9 ppm) (Supplementary Materials: Figure S104). Moreover, one proton singlet from the carbonyl moiety at δ = 11.92 ppm was correlated with C-1 (δ = 158.4 ppm), C-2 (δ = 120.1 ppm), and C-6 (δ = 118.9 ppm) (Supplementary Materials: Figure S103). The shift of the signal from the carbonyl group from δ = 192.1 ppm (in substrate **4**) to δ = 205.9 ppm also proved that the observed product was carboxylic acid. The attachment of the glucosyl moiety was established by the appearance of the characteristic carbon signals from δ = 80.5 ppm to δ = 62.3 ppm in the ^13^C NMR spectrum (Supplementary Materials: Figure S95), as well as proton signals of δ_H_ ranging from δ = 3.87 ppm to δ = 3.15 ppm and a one-proton doublet from proton at the anomeric carbon atom at δ = 4.86 ppm in the ^1^H-NMR spectrum (Supplementary Materials: Figure S92). The coupling constant (*J* = 7.8 Hz) for the anomeric proton pointed to the glucose *β*-configuration and a three-proton singlet δ = 3.55 ppm in the ^1^H NMR spectrum confirmed *O*-methylation of the attached glucosyl unit. The glucosidic unit substitution at C-4 was confirmed by the HMBC experiment, because the signal from the protons at C-6 (δ = 7.62 ppm), at C-3 (δ = 6.86 ppm), and at the anomeric carbon C-1′ (δ = 4.86 ppm) was correlated with C-4 (δ = 150.8 ppm (Supplementary Materials: Figure S103).

### 2.3. Biotransformations of 2′-Methylflavone (***5***) in the Culture of I. fumosorosea KCH J2

**Scheme 9:**
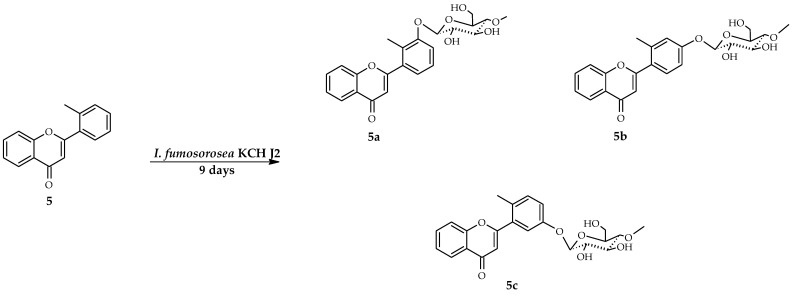
Microbial transformation of 2′-methylflavone (**5**) in the *I. fumosorosea* KCH J2 culture.

2′-Methylflavone (**5**) was biotransformed in the culture of *I. fumosorosea* KCH J2 with three biotransformation products obtained: 2′-methylflavone 3′-*O*-*β*-d-(4″-*O*-methyl)-glucopyranoside (**5a**) with a 10.3% yield (9.3 mg), 2′-methylflavone 4′-*O*-*β*-d-(4″-*O*-methyl)-glucopyranoside (**5b**) with a 10.9% yield (9.9 mg), and 2′-methylflavone 5′-*O*-*β*-d-(4″-*O*-methyl)-glucopyranoside (**5c**) with a 3.7% yield (3.4 mg) (Scheme 9).

**Scheme 10:**
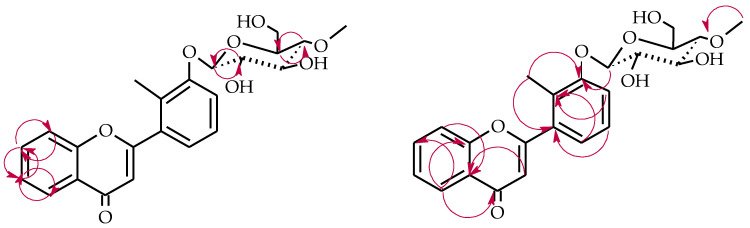
Key COSY (on the left) and HMBC (on the right) correlations for the structure elucidation of product **5a**.

**Scheme 11:**
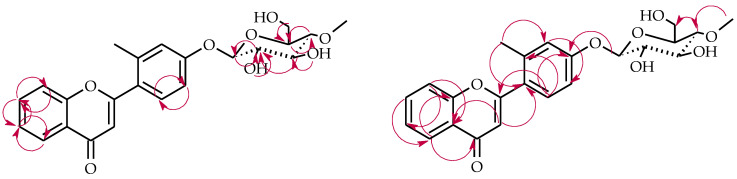
Key COSY (on the left) and HMBC (on the right) correlations for the structure elucidation of product **5b**.

**Scheme 12:**
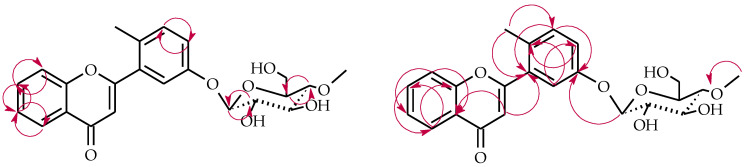
Key COSY (on the left) and HMBC (on the right) correlations for the structure elucidation of product **5c**.

The products **5a**–**5c** were analyzed by ^1^H-NMR, ^13^C-NMR, and correlations spectroscopy that allowed for the establishment of their chemical structures (Table 3 and Table 4, Scheme 10, Scheme 11 and Scheme 12 showing key COSY and HMBC correlations). In all three products occurred 4″-*O*-methylglycosylations that were confirmed by characteristic signals in the ^13^C NMR and ^1^H NMR spectra, analogically to the products **4a**–**4d** (Supplementary Materials: Figures S125, S122, S141, S138, S158, and S155). The presence of the three-proton singlet of the methyl group at C-2′ (δ = 2.37 ppm in **5a**, δ = 2.49 ppm in **5b,** and δ = 2.44 ppm in **5c**) in the ^1^H-NMR spectra of these products showed that the methyl moiety remained in all three products.

In product **5a**, the three-proton singlet of the methyl group at C-2′ was shifted from δ = 2.51 ppm (in **5**) to δ = 2.37 ppm in **5a**, indicating substitution at adjacent C-3′ position (Supplementary Materials: Figure S122). Glycosylation occurring at C-3′ was confirmed in the HMBC experiment by coupling of the proton at the anomeric carbon atom (δ = 5.00 ppm) with the C-3′ signal at δ = 157.2 ppm, which was shifted from δ = 132.1 ppm, indicating an electronegative atom attachment (Supplementary Materials: Figure S134). Additionally, in the ^13^C NMR spectrum the signals from C-2′ and C-4′ became shifted from δ = 137.7 ppm to δ = 127.4 ppm and δ = 131.6 ppm to δ = 118.2 ppm, respectively (Supplementary Materials: Figure S124). In the HMBC experiment the methyl moiety at C-2′ (δ = 2.37 ppm) was coupled with shifted C-3′ signal δ = 157.2 ppm same as doublet signal from proton at C-1″ also confirming that glycosylation occurred at C-3′ (Supplementary Materials: Figure S134) (Scheme 10).

The ^1^H NMR spectrum of product **5b** indicates that another product of glycosylation occurred. The protons from the A ring remained intact, but protons from B ring at C-6′, C-3′, and C-5′ became shifted, and the proton at C-4′ disappeared (Supplementary Materials: Figure S137). The three-proton singlet of the methyl group at C-2′ was only slightly shifted from δ = 2.51 ppm (in the substrate **5**) to δ = 2.49 ppm in **5a**, indicating substitution at further C-4′ position (Supplementary Materials: Figure S138). Glycosylation at C-4′ was confirmed in the HMBC experiment by coupling between the proton at the anomeric carbon atom (δ = 5.06 ppm) and the C-4′ signal (δ = 160.4 ppm), which was shifted from δ = 131.6 ppm (Supplementary Materials: Figure S151). Additionally, in the HMBC experiment, the methyl moiety at C-2′ (δ = 2.49 ppm) was coupled with shifted carbons signals: C-3′ (δ = 119.9 ppm), C-2′ (δ = 139.6 ppm), and C-1′ (δ = 127.4 ppm) (Supplementary Materials: Figure S151). The signal from proton at C-6′ (δ = 7.59 ppm) was also coupled with C-4′ (δ = 160.4 ppm) and C-2′ (δ = 139.6 ppm) in the HMBC experiment (Supplementary Materials: Figure S149) (Scheme 11).

In the case of **5c**, analysis of NMR spectra revealed that the resulting compound has a 4″-methyl-glucosyl moiety attached at C-5′. The ^1^H NMR spectra revealed that protons from A ring remained intact, but protons from the B ring at C-6′, C-3′, and C-4′ became shifted and coupling constants between them pointed that substitution occurred at C-5′ (Supplementary Materials: Figure S154). Glycosylation at C-5′ was confirmed in the HMBC experiment by coupling between the proton at the anomeric carbon atom (δ = 5.02 ppm) with the C-5′ signal (δ = 156.9 ppm), which was shifted from δ = 127.1 ppm, indicating the attachment of an electronegative atom (Supplementary Materials: Figure S167). Furthermore, in the HMBC experiment the signal from proton at C-6′ (δ = 7.35 ppm) was coupled with C-4′ (δ = 119.9 ppm) and C-2′ (δ = 131.0 ppm), signal from proton at C-3′ (δ = 7.31 ppm) was coupled with C-1′ (δ = 134.3 ppm) and shifted C-5′ (δ = 156.9 ppm) (Supplementary Materials: Figure S166) (Scheme 12).

## 3. Discussion

The main aim of the study was to investigate the ability of used strains of entomopathogenic filamentous fungi to biotransform 2′-methylflavanone and 2′-methylflavone, in order to obtain and characterize glycosylated and methylated derivatives with potentially improved bioavailability and bioactivity. The secondary objective was to receive compounds in amounts suitable for further preliminary in vitro studies of their interaction with biological membranes, liposomes, and human albumin, similar to our previous study [29]. The previous studies proved the ability of *B. bassiana* strains to glycosylate a wide variety of phenolic compounds, such as flavonoids, anthraquniones, and benzenediol lactones [26]. *B. bassiana* AM278 was able to attach a 4″-*O*-methyl-glycosyl moiety at C-7-OH (A ring) of flavanones such as pinocembrin, naringenin, eriodicytol and hesperetin, isoxanthohumol, and 8-prenylnaringenin. Hesperetin was also glycosylated at C-3′ (B ring) [22,30]. Our previous study with 6-methylflavanone as biotransformation substrate also resulted in the formation of 4″-*O*-methyl-glucosides at C-3′. Furthermore, they revealed its ability to catalyze hydroxylation and subsequent glycosylation of the methyl moiety attached at C-6 (A ring). However, in the case of 2′-methylflavanone, we did not observe products of glycosylation at C-3′. Instead, glycosylation occurred at C-6 and at C-4 (C ring). It can be assumed that the methyl moiety at C-2′ causes steric hindrance for enzymes responsible for methyl-glycosylation. The glycosylation at C-4 was previously observed in the case of 6-methylflavanone biotransformation in the culture of *I. fumosorosea* KCH J2 [21] and was probably preceded by the carbonyl group reduction also observed in 2′-methoxyflavanone and 3′-methoxyflavanone biotransformations [18].

Biotransformations of methoxyflavanones in the culture of *I. fumosorosea* KCH J2, in our earlier studies, resulted in the formation of 4″-*O*-methyl-glycosides attached at C-2′, C-3′, C-4′, C-5′ [18], whereas 6-methylflavanone in the culture of the same strain was glycosylated at C-4′ and C-4. Another *I. fumosorosea* strain, *I. fumosorosea* ACCC 37814, catalyzed glycosylation of naringenin at C-4′ and C-7. In the present study, 2′-methylflavanone was biotransformed into its 3′-*O*-*β*-d-(4″-*O*-methyl)-glucopyranoside, unlike the case of 2′-methoxyflavanone, which was glycosylated at C-2′ and C-5′ [18]. The second product obtained in this study was 2-methylbenzoic acid 4-*O*-*β*-d-(4′-*O*-methyl)-glucopyranoside (**4e**). It can be assumed that 2′-methyflavanone became glycosylated at C-4′ and then flavanone C-ring cleavage occurred.

Attempts to biotransform flavones in cultures of entomopathogenic filamentous fungi have been made in many previous studies resulting in 4″-*O*-methylglucosides attached at almost all possible positions: C-3, C-6, C-7, C-8, C-2′, C-3′, C-4′, and C-5′ [16,17,19,20,22,28,31]. In the presented study, in the culture of *I. fumosorosea* KCH J2, we also obtained glycosylated derivatives of 2′-methylflavone with the 4″-*O*-methyl-glucosyl moiety at C-3′, C-4′, and C-5′. These results can be compared to biotransformations performed by the same strain with 2′-methoxyflavone as a substrate. In that case the glycosylation occurred at C-3, C-8, C-2′, and C-5′. The glycosylation positions were different despite the identical substitution pattern of both compounds. On the other hand, *B. bassiana* KCH J1.5 was not able to biotransform 2′-methylflavone.

According to the literature, glycosylation improves aqueous solubility and bioavailability of flavonoids [3,7,9,11]. Similarly, in silico simulation using SwissADME tool to evaluate pharmacokinetic, drug-likeness and medicinal chemistry friendliness of small molecules, confirmed increased aqueous solubility of all obtained 4″-*O*-methylglucopyranosides. All products of biotransformation are predicted to be passively absorbed by the gastrointestinal tract. Glycosylated derivatives of 2′-methylflavanone and 2′-methylflavone are also predicted to lose an ability to passively permeate through the blood–brain barrier, but may be actively transported, unlike aglycones, by the P-glycoprotein.

The *C*-methylated flavonoids and their biological activity have not been thoroughly investigated. However, available data shed light on their antivirus [32], antifungal [33], antibacterial [34], anticancer, and antioxidant activities [35,36]. Furthermore, studies of two methylated flavonoids, i.e., 6-methylflavanone and 6-methylfavone, revealed that they act as positive modulators of γ-aminobutyric acid (GABA) [37,38].

The glycosylated flavanones and flavones with the methyl moiety presented in this manuscript have not been known until now and deserve further studies concerning their biological activity and bioavailability as potential drugs and diet supplements.

## 4. Materials and Methods

### 4.1. Substrates

The biotransformations substrates (**4**) and (**5**) were obtained for the first time by described below two-step synthesis (Scheme 1). The first step was the Claisen–Schmidt condensation between 2′-hydroxyacetophenone (**1**) and 2-methylbenzaldehyde (**2**) (purchased from Sigma-Aldrich (St. Louis, MO, USA)). The compounds were dissolved in methanol and the process was carried out under alkaline conditions at the boiling point of the solvent [21,39,40]. 2′-Hydroxy-2-methylchalcone (**3**) was obtained with a 63.8% yield along with 2′-methylflavanone (**4**) as a by-product with 28.2% yield. 2′-Methylflavone (**5**) was obtained by cyclization of 2′-hydroxy-2-methylchalcone (**3**) in the presence of iodine excess [21,39] with 45.5% yield along with 2′-methylflavanone as a by-product with 38.1% yield.

The physical data, including, color and form, melting point (°C), molecular ion mass, molecular formula, retention time t_R_ (min), retardation factor Rf, optical rotation ${\left[\alpha \right]}_{\;D}^{20}$ with concentration c (M), and NMR spectral data of the resulting compounds **4** and **5** are presented below, in Table 1, Table 2, Table 3 and Table 4, and in the Supplementary Materials.

#### 4.1.1. 2′-Methylflavanone (**4**)

Light-yellow crystals, mp = 47–48 °C, ESISMS m/z 239.3 ([M + H]^+^, C_16_H_14_O_2_), t_R_ = 17.20, Rf = 0.95,$\;{\left[\alpha \right]}_{\;D}^{20}$ = −7.0 (c = 2.80, acetone); ^1^H-NMR, see Table 1, ^13^C-NMR, see Table 2; Supplementary Materials: Figures S1–S16.

#### 4.1.2. 2′-Methylflavone (**5**)

Light-yellow oily liquid, ESIMS m/z 237.1 ([M + H]^+^, C_16_H_12_O_2_), t_R_ = 16.36, Rf = 0.95, ^1^H-NMR, see Table 3, ^13^C-NMR, see Table 4, Supplementary Materials: Figures S105–S118.

### 4.2. Microorganisms

Microorganisms for biotransformations—two strains of entomopathogenic filamentous fungi *I. fumosorosea* KCH J2 and *B. bassiana* KCH J1.5—were assembled from the Department of Chemistry of Wrocław University of Environmental and Life Sciences, Poland.

The description of material collection, fungi propagation, and genetic identification have already been described in our previous papers [19,27]. The filamentous fungi were maintained on potato slants at 4 °C and were subcultured before use in the biotransformations [19,27].

### 4.3. Analysis

The chromatographic methods (TLC, HPLC) were used to assess the course of the biotransformations. TLC analysis was carried out using TLC Silica gel 60/Kieselguhr F254 (0.2 mm thick) plates (Merck, Darmstadt, Germany) with a mixture of chloroform (Chempur, Piekary Śląskie, Poland) and methanol (Chempur, Piekary Śląskie, Poland) (9:1 *v/v*) as eluent. The products were observed without additional visualization under the ultraviolet lamp at λ = 254 nm and λ = 365 nm.

HPLC analyses were performed on a Dionex Ultimate 3000 instrument (Thermo Fisher Scientific, Waltham, MA, USA) with a DAD-3000 diode array detector using an analytical octadecylsilica (ODS) 2 column (4.6 mm × 250 mm, Waters, Milford, MA, USA) and pre-column. The gradient program was as follows: initial conditions—32.5% B in A, 4 min—40% B in A, 8 min—40% B in A, 10 min—45% B in A, 15 min—95% B in A, 18 min—95% B in A, 19 min—32.5% B in A, 23 min—32.5% B in A. The flow rate was 1 mL/min, the injection volume was 5 µL, and the detection wavelength was 280 nm [21].

The scale-up biotransformations products separation was attained using 500 µm and 1000 µm preparative TLC silica gel plates (Analtech, Gehrden, Germany) with a mixture of chloroform and methanol (9:1 *v*/*v*) as eluent. The compounds were extracted from the selected gel fractions using 20 mL ethyl acetate (Chempur, Piekary Śląskie, Poland) 3 times, and the solvent was evaporated under reduced pressure [21].

NMR analyses (^1^H-NMR, ^13^C-NMR, COSY, Heteronuclear Single Quantum Correlation (HSQC), HMBC) were performed using a DRX Avance^TM^ 600 MHz NMR spectrometer (Bruker, Billerica, MA, USA). The prepared samples of biotransformation substrates and products were dissolved in deuterated acetone.

Optical rotation was measured using digital polarimeter P-2000-Na (ABL&E-JASCO, Kraków, Poland).

Molecular formulas of all products were confirmed by analysis performed on the LC-MS 8045 SHIMADZU Triple Quadrupole Liquid Chromatograph Mass Spectrometer with electrospray ionization (ESI) source (Shimadzu, Kyoto, Japan), as described previously with minor modifications [28]. Analyses were conducted using method “MRM event from precursor ion search”. It means that in each analysis, in a sample with a pure compound, only a specific ion with a known molecular mass (determined by previous NMR analysis) was searched. The separation was achieved on the Kinetex column (2.6 µm C18 100 Å, 100 mm × 3 mm, Phenomenex, Torrance, CA, USA) operated at 30 °C. The mobile phase was a mixture of 0.1% aqueous formic acid *v/v* (A) and acetonitrile (B). The flow rate was 0.4 mL min^−1^ and the injection volume was 5 µL. The gradient program was as follows: initial conditions—80% B in A, 6.5 min—100% B, 7 min—80% B in A. The principal operating parameters for the LC-MS were set as follows: nebulizing gas flow: 3 L min^−1^, heating gas flow: 10 L min^−1^, interface temperature: 300 °C, drying gas flow: 10 L min^−1^, data acquisition range, *m*/*z* 100–1000 Da; ionization mode, negative and positive. Data were collected with LabSolutions version 5.97 (Shimadzu, Kyoto, Japan) software.

### 4.4. Screening Procedure

Biotransformations in the screening procedure were performed to assess the time needed for the complete microbial transformation of substrates **4** and **5**. As a growth medium for microorganisms we used modified Sabouraud medium (10 g aminobac (purchased from BTL, Warsaw, Poland), 30 g saccharose (purchased from Chempur, Piekary Śląskie, Poland), 1 L distilled water). In the first step, both cultures of the filamentous fungi strains were transferred from potato slants to 300 mL Erlenmeyer flasks with 100 mL modified Sabouraud liquid medium. These preincubation cultures were bred on a rotary shaker (DHN, Warsaw, Poland) (140 rpm) at 25 °C for 72 h. Second, 0.5 mL of the pre-grown cultures was transferred to another 300 mL Erlenmeyer flasks with 100 mL modified Sabouraud liquid medium and incubated same as in the pre-cultivation step. Afterwards, 10 mg of substrate **4** or **5** (dissolved in 0.5 mL of dimethyl sulfoxide (Chempur, Piekary Śląskie, Poland)) was added to each flask. The molar concentration of substrates **4** and **5** was 0.42 mM. All biotransformations were ended after confirming complete substrate conversion (or lack of further substrate conversion) after 9 days. During the experiments, the samples were collected after 3, 6, and 9 days of substrates incubation and extracted with 30 mL of ethyl acetate. Subsequently, the extracts were dried for 5 min with anhydrous magnesium sulfate (Chempur, Piekary Śląskie, Poland) and concentrated using a rotary evaporator (Heidolph, Schwabach, Germany) at 55 °C. All collected samples were analyzed by TLC and HPLC methods to check substrate conversion and the presence of flavonoid products visible on the TLC plates under an ultraviolet lamp. At the same time, the stability of the substrates under biotransformation conditions was investigated. Microorganisms cultivation with no substrate added was performed as a negative control [21].

### 4.5. The Semipreparative Biotransformations

The semipreparative biotransformations were performed in 2 L flasks with 500 mL of the modified Sabouraud medium each. One flask was used for each biotransformation. At first, 1 mL of the preincubation culture was transferred to the flask and incubated under the same conditions as during the screening procedure for 72 h. Afterwards, 50 mg of substrate **4** or **5** (dissolved in 2.5 mL of dimethyl sulfoxide) was added. The semipreparative biotransformations were performed for 9 days until the confirmation of complete substrate conversion (or lack of further substrate conversion). The molar concentration of substrates **4** and **5** was 0.42 mM. The post-reaction mixtures (mostly consisting of flavonoid products) were extracted 2 times (with 300 mL of ethyl acetate each time), then the joined extracts were dried for 5 min with anhydrous magnesium sulfate, and the solvent was evaporated. The biotransformations products were separated using preparative TLC plates. The fractions visible under ultraviolet lamp were marked, separated. extracted and afterwards analyzed by NMR and LC-MS [21].

The physical data, including color and form, melting point (°C), molecular ion mass, molecular formula, retention time t_R_ (min), retardation factor Rf, optical rotation ${\left[\alpha \right]}_{\;D}^{20}$ with concentration c (M), and NMR spectral data of the resulting compounds **4a**–**4e**, **5a**–**5c** are presented below, in Table 1, Table 2, Table 3 and Table 4, and in the Supplementary Materials.

#### 4.5.1. 2′-Methylflavanone 6-*O*-β-d-(4″-*O*-Methyl)-glucopyranoside (**4a**)

Light yellow crystals, mp = 161-162 °C, ESIMS *m*/*z* 430.7 ([M + H]^+^, C_23_H_26_O_8_), t_R_ = 8.29, Rf = 0.44,$\;{\left[\alpha \right]}_{\;D}^{20}$ = -63.2 (c = 0.730, acetone); ^1^H-NMR, see Table 1, ^13^C-NMR, see Table 2, Supplementary Materials: Figures S17–S36.

#### 4.5.2. 3′-Hydroxy-2′-Methylflavanone 6-*O*-β-d-(4″-*O*-Methyl)-glucopyranoside (**4b**)

Light-yellow crystals, mp = 102–103 °C, ESIMS *m*/*z* 445.3 ([M − H]^−^, C_23_H_26_O_9_), t_R_ = 4.15, Rf = 0.31,$\;{\left[\alpha \right]}_{\;D}^{20}$ = −82.5 (c = 0.340, acetone); ^1^H-NMR, see Table 1, ^13^C-NMR, see Table 2, Supplementary Materials: Figures S37–S52.

#### 4.5.3. 2-(2′-Methylphenyl)-chromane 4-*O*-β-d-(4″-*O*-Methyl)-glucopyranoside (**4c**)

Light-yellow crystals, mp = 78–80 °C, ESIMS *m*/*z* 461.2 (adduct [M + 46 − H]^−^ ([M + HCOOH − H]^−^) [21,41], C_23_H_28_O_7_), t_R_ = 11.11, Rf = 0.42,$\;{\left[\alpha \right]}_{\;D}^{20}$ = −32.5 (c = 0.590, acetone); ^1^H-NMR, see Table 1, ^13^C-NMR, see Table 2, Supplementary Materials: Figures S53–S70.

#### 4.5.4. 2′-Methylflavanone 3′-*O*-β-d-(4″-*O*-Methyl)-glucopyranoside (**4d**)

Light-yellow crystals, mp = 192–194 °C ESIMS *m*/*z* 431.2 ([M + H]^+^, C_23_H_26_O), t_R_ = 7.13, Rf = 0.48,$\;{\left[\alpha \right]}_{\;D}^{20}$ = −114.4 (c = 0.330, acetone); ^1^H-NMR, see Table 1, ^13^C-NMR, see Table 2, Supplementary Materials: Figures S71–S88.

#### 4.5.5. 2-Methylbenzoic Acid 4-*O*-β-d-(4″-*O*-Methyl)-glucopyranoside (**4e**)

Light-yellow crystals, mp = 176–178°C, ESIMS *m*/*z* 327.4 ([M − H]^−^, C_15_H_20_O_8_), t_R_ = 3.09, Rf = 0.39, ^1^H-NMR, see Table 1, ^13^C-NMR, see Table 2, Supplementary Materials: Figures S89–S104.

#### 4.5.6. 2′-Methylflavone 3′-*O*-β-d-(4″-*O*-Methyl)-glucopyranoside (**5a**)

Very light-yellow crystals, mp = 202–204 °C, ESIMS *m*/*z* 429.1 ([M + H]^+^, C_23_H_24_O_8_), t_R_ = 5.22, Rf = 0.55, ^1^H-NMR, see Table 3, ^13^C-NMR, see Table 4, Supplementary Materials: Figures S119–S134.

#### 4.5.7. 2′-Methylflavone 4′-*O*-β-d-(4″-*O*-Methyl)-glucopyranoside (**5b**)

Very light-yellow crystals, mp = 103–104 °C ESIMS *m*/*z* 429.1 ([M + H]^+^, C_23_H_24_O_8_), t_R_ = 5.03, Rf = 0.51, ^1^H-NMR, see Table 3, ^13^C-NMR, see Table 4, Supplementary Materials: Figures S135–S151.

#### 4.5.8. 2′-Methylflavone 5′-*O*-β-d-(4″-*O*-Methyl)-glucopyranoside (**5c**)

Very light-yellow crystals, mp = 58–60 °C ESIMS *m*/*z* 429.2 ([M + H]^+^, C_23_H_24_O_8_), t_R_ = 5.67, Rf = 0.59,^1^H-NMR, see Table 3, ^13^C-NMR, see Table 4, Supplementary Materials: Figures S152–S167.

## 5. Conclusions

In the present study, two methylated flavonoids belonging to different flavonoid subgroups, i.e., 2′-methylflavanone and 2′-methyflavone, were synthesized. Subsequently, compounds were biotransformed in the cultures of two entomopathogenic filamentous fungi strains: *B. bassiana* KCH J1.5 and *I. fumosorosea* KCH J2. The strain *B. bassiana* KCH J1.5 was able to glycosylate 2′-methylflavanone at C-6, C-4, and to hydroxylate flavanone skeleton at C-3′. It can be assumed that the reduction of the carbonyl group preceded the attachment of the glycosyl moiety at C-4. In all resulting flavanones, the 2′-methyl moiety remained intact. The inability to glycosylate the 2′-methylflavanone B ring indicates that the methyl moiety at C-2′ causes steric hindrance to the glycosyltransferase. Furthermore, *B. bassiana* KCH J1.5 was unable to biotransform 2′-methylflavone, the structure of which is planar and more rigid than flavanone. In the case of microbial transformation of 2′-methylflavanone carried out by *I. fumosorosea* KCH J2, another two products were obtained. The first one was a product of glycosylation at C-3′. The second one was a glycosylated product of the C-ring cleavage of the flavanone skeleton—2-methylbenzoic acid 4-*O*-*β*-d-(4′-*O*-methyl)-glucopyranoside.

On the other hand, 2′-methylflavone was effectively biotransformed into three glycosides with 4″-*O*-methyl-glucosyl moiety attached at C-3′, C-4′, and C-5′. All the above-mentioned biotransformation products have not been described in the literature until now. The biological activity and bioavailability of these compounds needs investigation in further studies.

## Figures and Tables

**Table 1:** ^1^H-NMR chemical shifts δ (ppm) and coupling constants *J* (Hz) of 2′-methylflavanone (**4**) and products of its biotransformations **4a**–**4e** in Acetone-d6, 600 MHz (Supplementary Materials: Figures S2, S18, S38, S54, S72, S90).

Proton	Compound					
	4	4a	4b	4c	4d	4e
H-2	5.84 (dd) *J* = 13.5, *J* = 2.6	5.78 (dd) *J* = 13.6, *J* = 2.6	5.77 (dd) *J* = 13.4, *J* = 2.7	5.47 (dd) *J* = 12.1, *J* = 1.7	5.86 (dd) *J* = 13.5, *J* = 2.6	-
H-3_ax_	3.15 (dd) *J* = 16.8, *J* = 13.5	3.12 (ddd) *J* = 16.8, *J* = 13.5, *J* = 3.3	3.08 (dd) *J* = 16.9, *J* = 13.4	2.45 (dt) *J* = 14.8, *J* = 2.0	3.12 (dd) *J* = 16.8, *J* = 13.4	-
H-3_eq_	2.81 (dd) *J* = 16.8, *J* = 2.6	2.80 (dd) *J* = 16.9, *J* = 2.7	2.79 (dd) *J* = 17.1 *J* = 2.7	1.96 (m)	2.81 (dd) *J* = 16.9, *J* = 2.7	-
H-4	-	-	-	4.97 (m)	-	-
H-5	7.87 (dd) *J* = 7.8, *J* = 1.7	7.49 (d) *J* = 3.1	7.48 (d) *J* = 3.1	7.43 (dd) *J* = 7.7, *J* = 1.6	7.86 (dd) *J* = 7.9, *J* = 1.7	-
H-6	7.10 (ddd) *J* = 7.9, *J* = 7.2, *J* = 0.9	-	-	6.93 (td) *J* = 7.4, *J* = 1.1	7.10 (td) *J* = 7.7, *J* = 1.0	-
H-7	7.58 (m)	7.33 (d) *J* = 8.9, *J* = 3.1	7.33 (dd) *J* = 8.9, *J* = 3.1	7.23 (m)	7.58 (ddd) *J* = 8.5, *J* = 7.2, *J* = 1.7	-
H-8	7.07 (dd) *J* = 8.5, *J* = 0.8	7.02 (dd) *J* = 9.0, *J* = 1.8	7.01 (dd) *J* = 8.9, *J* = 2.0	6.87 (m)	7.07 (m)	-
H-3′	7.30 (m)	7.28 (m)	-	7.23 (m)	-	(3) 6.86 (d) *J* = 9.0
H-4′	7.27 (m)	7.28 (m)	6.89 (dd) *J* = 6.6, *J* = 2.5	7.23 (m)	7.18 (d) *J* = 7.5	-
H-5′	7.30 (m)	7.28 (m)	7.11 (m)	7.23 (m)	7.24 (t) *J* = 8.0	(5) 7.29 (d) *J* = 9.0, *J* = 3.0
H-6′	7.64 (dd) *J* = 7.0, *J* = 2.0	7.62 (m)	7.11 (m)	7.54 (m)	7.33 (d) *J* = 7.7	(6) 7.62 (d) *J* = 2.9
H-1″	-	4.88 (d) *J* = 7.8	4.88 (d) *J* = 7.8	4.63 (d) *J* = 7.8	4.94 (d) *J* = 7.8	(1′) 4.86 (d) *J* = 7.8
H-2″	-	3.46 (ddd) *J* = 10.8, *J* = 6.6, *J* = 3.2	3.46 (m)	3.22 (m)	3.54 (m)	(2′) 3.50 (ddd) *J* = 9.6, *J* = 5.7, *J* = 1.7
H-3″	-	3.63 (m)	3.63 (m)	3.57 (dd) *J* = 8.9, *J* = 3.4	3.64 (td) *J* = 9.0, *J* = 4.0	(3′) 3.61 (m)
H-4″	-	3.24 (m)	3.24 (m)	3.14 (m)	3.23 (m)	(4′) 3.15 (m)
H-5″	-	3.46 (ddd) *J* = 10.8, *J* = 6.6, *J* = 3.2	3.46 (m)	3.36 (dd) *J* = 9.7, *J* = 5.0	3.47 (ddd) *J* = 9.8, *J* = 4.8, *J* = 2.2	(5′) 3.45 (tt) *J* = 5.3, *J* = 3.6
H-6″	-	3.83 (ddd) *J* = 8.0, *J* = 4.1, *J* = 1.9 3.70 (dd) *J* = 7.2, *J* = 4.4	3.83 (ddd) *J* = 12.7, *J* = 8.2, *J* = 4.1 3.70 (dd) *J* = 7.1, *J* = 4.5	3.87 (m) 3.72 (dd) *J* = 14.0, *J* = 3.9	3.84 (ddd) *J* = 11.6, *J* = 5.1, *J* = 2.2 3.70 (ddd) *J* = 11.8, *J* = 7.1, *J* = 4.8	(6′) 3.87 (qd) *J* = 5.7, *J* = 3.0 3.68 (m)
C4″-OCH_3_	-	3.57 (s)	3.57 (s)	3.54 (s)	3.57 (s)	(4′-OCH_3_) 3.55 (s)
C2′-CH_3_	2.44 (s)	2.43 (s)	2.27 (s)	2.43 (s)	2.34 (s)	(2-CH_3_) 2.65 (s)
2″-OH	-	4.69 (d) *J* = 3.9	4.69 (d) *J* = 3.3	4.44 (d) *J* = 4.0	4.67 (d) *J* = 4.6	(2′-OH) 4.64 (d) *J* = 3.6
3″-OH	-	4.40 (d) *J* = 3.9	4.39 (d) *J* = 3.7	4.23 (d) *J* = 3.7	4.44(d) *J* = 4.1	(3′-OH) 4.41 (d) *J* = 4.0
6″-OH	-	3.72 (t) *J* = 3.1	3.72 (m)	3.72 (dd) *J* = 14.0, *J* = 3.9	3.76 (m)	-
-COOH		-	-		-	11.92 (s)

**Table 2:** ^13^C-NMR chemical shifts δ (ppm) and coupling constants *J* (Hz) of 2′-methylflavanone (**4**) and products of its biotransformations **4a**–**4e** in Acetone-d6, 151 MHz (Supplementary Materials: Figures S5, S21, S41, S57, S75, S93).

Carbon	Compound					
	4	4a	4b	4c	4d	4e
C- 2	77.6	77.7	77.9	71.1	77.6	-
C-3	43.8	43.8	43.9	34.5	44.0	-
C-4	192.1	191.9	192.0	69.2	192.0	(C=O) 205.9
C-4a	122.0	122.1	123.1	122.8	122.0	-
C-5	127.4	114.0	113.9	132.9	127.4	-
C-6	122.3	153.2	153.2	121.1	122.3	-
C-7	136.8	126.7	126.7	130.3	136.8	-
C-8	118.9	119.8	119.9	117.5	118.9	-
C-8a	162.7	158.1	158.1	156.5	162.7	-
C-1′	138.1	138.2	122.1	140.4	139.3	(1) 158.4
C-2′	136.5	136.5	139.5	136.2	126.2	(2) 120.1
C-3′	129.3	131.5	156.3	128.4	156.9	(3) 119.3
C-4′	131.5	129.3	115.6	131.2	116.1	(4) 150.8
C-5′	127.1	127.1	127.3	126.9	127.5	(5) 127.6
C-6′	126.9	126.9	118.1	126.7	120.5	(6) 118.9
C-1″	-	102.7	102.7	100.7	102.2	(1′) 102.8
C-2″	-	75.0	75.0	75.0	75.1	(2′) 77.3
C-3″	-	77.9	77.9	78.2	78.1	(3′) 78.1
C-4″	-	80.0	80.0	80.7	80.1	(4′) 80.5
C-5″	-	77.0	77.0	77.0	77.0	(5′) 74.9
C-6″	-	62.0	62.0	62.6	62.1	(6′) 62.3
4″-OCH_3_	-	60.5	60.5	60.5	60.5	(4′-OCH_3_) 60.6
2′-CH_3_	19.1	19.1	11.1	19.3	11.4	(2-CH_3_) 27.0

**Table 3:** ^1^H-NMR chemical shifts δ (ppm) and coupling constants *J*(Hz) of 2′-methylflavone (**5**) and products of its biotransformations **5a**–**5c** in Acetone-d6, 600 MHz (Supplementary Materials: Figures S106, S120, S136, S153).

Proton	Compound			
	5	5a	5b	5c
H-3	6.42 (s)	6.39 (s)	6.39 (s)	6.44 (s)
H-5	8.15 (dd) *J* = 7.9, *J* = 1.7	8.15 (m)	8.14 (dd) *J* = 8.0, *J* = 1.6	8.14 (dd) *J* = 8.0, *J* = 1.7
H-6	7.50 (m)	7.51 (t) *J* = 7.4,	7.50 (m)	7.51 (m)
H-7	7.82 (ddd) *J* = 8.7, *J* = 7.2, *J* = 1.7	7.82 (m)	7.80 (ddd) *J* = 8.7, *J* = 7.2, *J* = 1.7	7.82 (ddd) *J* = 8.7, *J* = 7.2, *J* = 1.7
H-8	7.64 (m)	7.63 (d) *J* = 8.4	7.63 (d) *J* = 8.4	7.66 (dd) *J* = 8.4 *J* = 0.8
H-3′	7.40 (m)	-	7.06 (m)	7.31 (d) *J* = 8.4
H-4′	7.50 (m)	7.36 (d) *J* = 8.1	-	7.17 (dd) *J* = 8.4, *J* = 2.7
H-5′	7.40 (m)	7.32 (t) *J* = 7.9	7.06 (m)	-
H-6′	7.64 (m)	7.28 (d) *J* = 6.9	7.59 (d) *J* = 8.4	7.35 (d) *J* = 2.6
H-1″	-	5.00 (d) *J* = 7.7	5.06 (d) *J* = 7.8	5.02 (d) *J* = 7.8
H-2″	-	3.55 (d) *J* = 6.6	3.49 (m)	3.47 (dd) *J* = 8.4, *J* = 3.2
H-3″	-	3.65 (m)	3.65 (tt) *J* = 6.7, *J* = 3.4	3.63 (m)
H-4″	-	3.25 (t) *J* = 9.3	3.23 (m)	3.20 (m)
H-5″	-	3.51 (ddd) *J* = 9.6, *J* = 4.7, *J* = 2.0	3.53 (ddd) *J* = 9.8, *J* = 5.0, *J* = 2.1	3.50 (ddd) *J* = 9.8, *J* = 5.0, *J* = 2.1
H-6″	-	3.86 (m) 3.71 (ddd) *J* = 10.8, *J* = 7.8, *J* = 4.5	3.86 (ddd) *J* = 11.4, *J* = 5.2, *J* = 2.0 3.70 (ddd) *J* = 11.5, *J* = 6.5, *J* = 5.1	3.84 (ddd) *J* = 11.6, *J* = 5.1, *J* = 2.1 3.68 (m)
C4″-OCH_3_	-	3.57 (s)	3.57 (s)	3.55 (s)
C2′-CH_3_	2.51 (s)	2.37 (s)	2.49 (s)	2.44 (s)
2″-OH	-	4.72 (d) *J* = 4.3	4.72 (d) *J* = 4.2	4.67 (d) *J* = 3.8
3″-OH	-	4.46 (d) *J* = 4.0	4.46 (d) *J* = 4.1	4.41 (d) *J* = 3.8
6″-OH	-	3.80 (dd) *J* = 14.3, *J* = 7.8	3.82 (m)	3.78 (dd) *J* = 10.2, *J* = 4.3

**Table 4:** ^13^C-NMR chemical shifts δ (ppm) and coupling constants *J* (Hz) of 2′-methylflavone (**5**) and products of its biotransformations **5a**–**5c** in Acetone-d6, 151 MHz (Supplementary Materials: Figures S108, S123, S139, S156).

Carbon	Compound			
	5	5a	5b	5c
C-2	166.7	166.5	166.5	166.2
C-3	112.4	112.7	111.9	112.4
C-4	177.8	177.8	177.8	177.8
C-4a	124.7	124.7	124.7	124.7
C-5	126.0	126.1	126.0	126.0
C-6	126.2	126.2	126.1	126.2
C-7	134.9	134.9	134.8	134.9
C-8	119.2	119.2	119.2	119.3
C-8a	157.4	157.4	157.3	157.4
C-1′	133.7	135.1	127.4	134.3
C-2′	137.7	127.4	139.6	131.0
C-3′	132.1	157.2	119.9	133.1
C-4′	131.6	118.2	160.4	119.9
C-5′	127.1	127.8	114.9	156.9
C-6′	130.2	123.8	131.7	118.1
C-1″	-	102.2	101.3	101.8
C-2″	-	75.0	74.9	75.0
C-3″	-	78.1	78.0	78.0
C-4″	-	80.1	80.1	80.2
C-5″	-	77.1	77.2	77.1
C-6″	-	62.1	62.1	62.2
4″-OCH_3_	-	60.6	60.6	60.5
2′-CH_3_	20.5	13.5	21.0	19.8

## Data Availability

Samples of the compounds **4**, **4a**–**4e**, **5**, **5a**–**5c** are available from the authors.

## Supplementary Materials

The following are available online at https://www.mdpi.com/article/10.3390/ijms22179617/s1, Figures S1–S167.
