# Supplementary material for: Fungal Biotransformation of 2′-Methylflavanone and 2′-Methylflavone as a Method to Obtain Glycosylated Derivatives
Source: Int J Mol Sci. 2021 Sep 5;22(17):9617. doi: 10.3390/ijms22179617 (PMC8431774; doi:10.3390/ijms22179617)
Supplement: Supplementary file 1 [file ijms-22-09617-s001.zip › ijms-1341735-supplementary.pdf]

Supplementary materials

Fungal biotransformation of 2′-methylflavanone and 2′-methylflavone as a method to obtain glycosylated derivatives

Agnieszka Krawczyk-Łebek*, Monika Dymarska, Tomasz Janeczko and Edyta Kostrzewa-Susłow*

Department of Chemistry, Faculty of Biotechnology and Food Science, Wrocław University of Environmental and Life Sciences, Wrocław, Poland
*Correspondence: agnieszka.krawczyk-lebek@upwr.edu.pl, edyta.kostrzewa-suslow@upwr.edu.pl

Content

Figure S1. MS analysis of 2′-methyflavanone (**4**)
Figure S2. ^{1}H NMR spectrum (*δ*, acetone-d6, 600 MHz) of 2′-methyflavanone (**4**)
Figure S3 ^{1}H NMR spectrum expansion (*δ*, acetone-d6, 600 MHz) of 2′-methyflavanone (**4**)
Figure S4. ^{1}H NMR spectrum expansion (*δ*, acetone-d6, 600 MHz) of 2′-methyflavanone (**4**)
Figure S5. ^{13}C NMR spectrum (*δ*, acetone-d6, 151 MHz) of 2′-methyflavanone (**4**)
Figure S6. ^{13}C NMR spectrum expansion (*δ*, acetone-d6, 151 MHz) of 2′-methyflavanone (**4**)
Figure S7. ^{13}C NMR spectrum expansion (*δ*, acetone-d6, 151 MHz) of 2′-methyflavanone (**4**)
Figure S8. COSY contour map – ^{1}H x ^{1}H of 2′-methyflavanone (**4**)
Figure S9. COSY contour map – ^{1}H x ^{1}H expansion of 2′-methyflavanone (**4**)
Figure S10. COSY contour map – ^{1}H x ^{1}H expansion of 2′-methyflavanone (**4**)
Figure S11. HSQC contour map – ^{1}H x ^{13}C of 2′-methyflavanone (**4**)
Figure S12. HSQC contour map – ^{1}H x ^{13}C expansion of 2′-methyflavanone (**4**)
Figure S13. HSQC contour map – ^{1}H x ^{13}C expansion of 2′-methyflavanone (**4**)
Figure S14. HMBC contour map – ^{1}H x ^{13}C of 2′-methyflavanone (**4**)
Figure S15. HMBC contour map – ^{1}H x ^{13}C expansion of 2′-methyflavanone (**4**)
Figure S16. HMBC contour map – ^{1}H x ^{13}C expansion of 2′-methyflavanone (**4**)
Figure S17. MS analysis of 2′-methylflavanone 6-*O*-*β*-D-(4′′-*O*-methyl)-glucopyranoside (**4a**)
Figure S18. ^{1}H NMR spectrum (*δ*, acetone-d6, 600 MHz) of 2′-methylflavanone 6-*O*-*β*-D-(4′′-*O*-methyl)-glucopyranoside (**4a**)
Figure S19. ^{1}H NMR spectrum expansion (*δ*, acetone-d6, 600 MHz) of 2′-methylflavanone 6-*O*-*β*-D-(4′′-*O*-methyl)-glucopyranoside (**4a**)
Figure S20. ^{1}H NMR spectrum expansion (*δ*, acetone-d6, 600 MHz) of 2′-methylflavanone 6-*O*-*β*-D-(4′′-*O*-methyl)-glucopyranoside (**4a**)
Figure S21. ^{13}C NMR spectrum expansion (*δ*, acetone-d6, 151 MHz) of 2′-methylflavanone 6-*O*-*β*-D-(4′′-*O*-methyl)-glucopyranoside (**4a**)
Figure S22. ^{13}C NMR spectrum expansion (*δ*, acetone-d6, 151 MHz) of 2′-methylflavanone 6-*O*-*β*-D-(4′′-*O*-methyl)-glucopyranoside (**4a**)
Figure S23. ^{13}C NMR spectrum expansion (*δ*, acetone-d6, 151 MHz) of 2′-methylflavanone 6-*O*-*β*-D-(4′′-*O*-methyl)-glucopyranoside (**4a**)

Figure S24. COSY contour map – ^{1}H x ^{1}H of 2′-methylflavanone 6-*O*-*β*-D-(4′′-*O*-methyl)-glucopyranoside (**4a**)
Figure S25. COSY contour map – ^{1}H x ^{1}H expansion of 2′-methylflavanone 6-*O*-*β*-D-(4′′-*O*-methyl)-glucopyranoside (**4a**)
Figure S26. COSY contour map – ^{1}H x ^{1}H expansion of 2′-methylflavanone 6-*O*-*β*-D-(4′′-*O*-methyl)-glucopyranoside (**4a**)
Figure S27. HSQC contour map – ^{1}H x ^{13}C of 2′-methylflavanone 6-*O*-*β*-D-(4′′-*O*-methyl)-glucopyranoside (**4a**)
Figure S28. HSQC contour map – ^{1}H x ^{13}C expansion of 2′-methylflavanone 6-*O*-*β*-D-(4′′-*O*-methyl)-glucopyranoside (**4a**)
Figure S29. HSQC contour map – ^{1}H x ^{13}C expansion of 2′-methylflavanone 6-*O*-*β*-D-(4′′-*O*-methyl)-glucopyranoside (**4a**)
Figure S30. HSQC contour map – ^{1}H x ^{13}C expansion of 2′-methylflavanone 6-*O*-*β*-D-(4′′-*O*-methyl)-glucopyranoside (**4a**)
Figure S31. HMBC contour map – ^{1}H x ^{13}C of 2′-methylflavanone 6-*O*-*β*-D-(4′′-*O*-methyl)-glucopyranoside (**4a**)
Figure S32. HMBC contour map – ^{1}H x ^{13}C expansion of 2′-methylflavanone 6-*O*-*β*-D-(4′′-*O*-methyl)-glucopyranoside (**4a**)
Figure S33. HMBC contour map – ^{1}H x ^{13}C expansion of 2′-methylflavanone 6-*O*-*β*-D-(4′′-*O*-methyl)-glucopyranoside (**4a**)
Figure S34. HMBC contour map – ^{1}H x ^{13}C expansion of 2′-methylflavanone 6-*O*-*β*-D-(4′′-*O*-methyl)-glucopyranoside (**4a**)
Figure S35. HMBC contour map – ^{1}H x ^{13}C expansion of 2′-methylflavanone 6-*O*-*β*-D-(4′′-*O*-methyl)-glucopyranoside (**4a**)
Figure S36. HMBC contour map – ^{1}H x ^{13}C expansion of 2′-methylflavanone 6-*O*-*β*-D-(4′′-*O*-methyl)-glucopyranoside (**4a**)
Figure S37. MS analysis of 3′-hydroxy-2′-methylflavanone 6-*O*-*β*-D-(4′′-*O*-methyl)-glucopyranoside (**4b**)
Figure S38. ^{1}H NMR spectrum (*δ*, acetone-d6, 600 MHz) of 3′-hydroxy-2′-methylflavanone 6-*O*-*β*-D-(4′′-*O*-methyl)-glucopyranoside (**4b**)
Figure S39. ^{1}H NMR spectrum expansion (*δ*, acetone-d6, 600 MHz) of 3′-hydroxy-2′-methylflavanone 6-*O*-*β*-D-(4′′-*O*-methyl)-glucopyranoside (**4b**)
Figure S40. ^{1}H NMR spectrum expansion (*δ*, acetone-d6, 600 MHz) of 3′-hydroxy-2′-methylflavanone 6-*O*-*β*-D-(4′′-*O*-methyl)-glucopyranoside (**4b**)
Figure S41. ^{13}C NMR spectrum (*δ*, acetone-d6, 151 MHz) of 3′-hydroxy-2′-methylflavanone 6-*O*-*β*-D-(4′′-*O*-methyl)-glucopyranoside (**4b**)
Figure S42. ^{13}C NMR spectrum expansion (*δ*, acetone-d6, 151 MHz) of 3′-hydroxy-2′-methylflavanone 6-*O*-*β*-D-(4′′-*O*-methyl)-glucopyranoside (**4b**)
Figure S43. ^{13}C NMR spectrum expansion (*δ*, acetone-d6, 151 MHz) of 3′-hydroxy-2′-methylflavanone 6-*O*-*β*-D-(4′′-*O*-methyl)-glucopyranoside (**4b**)

Figure S44. COSY contour map – ^{1}H x ^{1}H of 3′-hydroxy-2′-methylflavanone 6-*O*-*β*-D-(4′′-*O*-methyl)-glucopyranoside (**4b**)
Figure S45. COSY contour map – ^{1}H x ^{1}H expansion of 3′-hydroxy-2′-methylflavanone 6-*O*-*β*-D-(4′′-*O*-methyl)-glucopyranoside (**4b**)
Figure S46. COSY contour map – ^{1}H x ^{1}H expansion of 3′-hydroxy-2′-methylflavanone 6-*O*-*β*-D-(4′′-*O*-methyl)-glucopyranoside (**4b**)
Figure S47. HSQC contour map – ^{1}H x ^{13}C of 3′-hydroxy-2′-methylflavanone 6-*O*-*β*-D-(4′′-*O*-methyl)-glucopyranoside (**4b**)
Figure S48. HSQC contour map – ^{1}H x ^{13}C expansion of 3′-hydroxy-2′-methylflavanone 6-*O*-*β*-D-(4′′-*O*-methyl)-glucopyranoside (**4b**)
Figure S49. HSQC contour map – ^{1}H x ^{13}C expansion of 3′-hydroxy-2′-methylflavanone 6-*O*-*β*-D-(4′′-*O*-methyl)-glucopyranoside (**4b**)
Figure S50. HMBC contour map – ^{1}H x ^{13}C 3′-hydroxy-2′-methylflavanone 6-*O*-*β*-D-(4′′-*O*-methyl)-glucopyranoside (**4b**)
Figure S51. HMBC contour map – ^{1}H x ^{13}C expansion of 3′-hydroxy-2′-methylflavanone 6-*O*-*β*-D-(4′′-*O*-methyl)-glucopyranoside (**4b**)
Figure S52. HMBC contour map – ^{1}H x ^{13}C expansion of 3′-hydroxy-2′-methylflavanone 6-*O*-*β*-D-(4′′-*O*-methyl)-glucopyranoside (**4b**)
Figure S53. MS analysis of 2-(2′-methylphenyl)-chromane 4-*O*-*β*-D-(4′′-*O*-methyl)-glucopyranoside (**4c**)
Figure S54. ^{1}H NMR spectrum (*δ*, acetone-d6, 600 MHz) of 2-(2′-methylphenyl)-chromane 4-*O*-*β*-D-(4′′-*O*-methyl)-glucopyranoside (**4c**)
Figure S55. ^{1}H NMR spectrum expansion (*δ*, acetone-d6, 600 MHz) of 2-(2′-methylphenyl)-chromane 4-*O*-*β*-D-(4′′-*O*-methyl)-glucopyranoside (**4c**)
Figure S56. ^{1}H NMR spectrum expansion (*δ*, acetone-d6, 600 MHz) of 2-(2′-methylphenyl)-chromane 4-*O*-*β*-D-(4′′-*O*-methyl)-glucopyranoside (**4c**)
Figure S57. ^{13}C NMR spectrum (*δ*, acetone-d6, 151 MHz) of 2-(2′-methylphenyl)-chromane 4-*O*-*β*-D-(4′′-*O*-methyl)-glucopyranoside (**4c**)
Figure S58. ^{13}C NMR spectrum expansion (*δ*, acetone-d6, 151 MHz) of 2-(2′-methylphenyl)-chromane 4-*O*-*β*-D-(4′′-*O*-methyl)-glucopyranoside (**4c**)
Figure S59. ^{13}C NMR spectrum expansion (*δ*, acetone-d6, 151 MHz) of 2-(2′-methylphenyl)-chromane 4-*O*-*β*-D-(4′′-*O*-methyl)-glucopyranoside (**4c**)
Figure S60. COSY contour map – ^{1}H x ^{1}H of 2-(2′-methylphenyl)-chromane 4-*O*-*β*-D-(4′′-*O*-methyl)-glucopyranoside (**4c**)
Figure S61. COSY contour map – ^{1}H x ^{1}H expansion of of 2-(2′-methylphenyl)-chromane 4-*O*-*β*-D-(4′′-*O*-methyl)-glucopyranoside (**4c**)
Figure S62. COSY contour map – ^{1}H x ^{1}H expansion of of 2-(2′-methylphenyl)-chromane 4-*O*-*β*-D-(4′′-*O*-methyl)-glucopyranoside (**4c**)
Figure S63. HSQC contour map – ^{1}H x ^{13}C of 2-(2′-methylphenyl)-chromane 4-*O*-*β*-D-(4′′-*O*-methyl)-glucopyranoside (**4c**)

Figure S64. HSQC contour map – ^{1}H x ^{13}C expansion of 2-(2′-methylphenyl)-chromane 4-*O*-*β*-D-(4′′-*O*-methyl)-glucopyranoside (**4c**)
Figure S65. HSQC contour map – ^{1}H x ^{13}C expansion of 2-(2′-methylphenyl)-chromane 4-*O*-*β*-D-(4′′-*O*-methyl)-glucopyranoside (**4c**)
Figure S66. HMBC contour map – ^{1}H x ^{13}C of 2-(2′-methylphenyl)-chromane 4-*O*-*β*-D-(4′′-*O*-methyl)-glucopyranoside (**4c**)
Figure S67. HMBC contour map – ^{1}H x ^{13}C expansion of 2-(2′-methylphenyl)-chromane 4-*O*-*β*-D-(4′′-*O*-methyl)-glucopyranoside (**4c**)
Figure S68. HMBC contour map – ^{1}H x ^{13}C expansion of 2-(2′-methylphenyl)-chromane 4-*O*-*β*-D-(4′′-*O*-methyl)-glucopyranoside (**4c**)
Figure S69. HMBC contour map – ^{1}H x ^{13}C expansion of 2-(2′-methylphenyl)-chromane 4-*O*-*β*-D-(4′′-*O*-methyl)-glucopyranoside (**4c**)
Figure S70. HMBC contour map – ^{1}H x ^{13}C expansion of 2-(2′-methylphenyl)-chromane 4-*O*-*β*-D-(4′′-*O*-methyl)-glucopyranoside (**4c**)
Figure S71. MS analysis of 2′-methylflavanone 3′-*O*-*β*-D-(4′′-*O*-methyl)-glucopyranoside (**4d**)
Figure S72. ^{1}H NMR spectrum (*δ*, acetone-d6, 600 MHz) of 2′-methylflavanone 3′-*O*-*β*-D-(4′′-*O*-methyl)-glucopyranoside (**4d**)
Figure S73. ^{1}H NMR spectrum expansion (*δ*, acetone-d6, 600 MHz) of 2′-methylflavanone 3′-*O*-*β*-D-(4′′-*O*-methyl)-glucopyranoside (**4d**)
Figure S74. ^{1}H NMR spectrum expansion (*δ*, acetone-d6, 600 MHz) of 2′-methylflavanone 3′-*O*-*β*-D-(4′′-*O*-methyl)-glucopyranoside (**4d**)
Figure S75. ^{13}C NMR spectrum (*δ*, acetone-d6, 151 MHz) of 2′-methylflavanone 3′-*O*-*β*-D-(4′′-*O*-methyl)-glucopyranoside (**4d**)
Figure S76. ^{13}C NMR spectrum expansion (*δ*, acetone-d6, 151 MHz) of 2′-methylflavanone 3′-*O*-*β*-D-(4′′-*O*-methyl)-glucopyranoside (**4d**)
Figure S77. ^{13}C NMR spectrum expansion (*δ*, acetone-d6, 151 MHz) of 2′-methylflavanone 3′-*O*-*β*-D-(4′′-*O*-methyl)-glucopyranoside (**4d**)
Figure S78. COSY contour map – ^{1}H x ^{1}H of 2′-methylflavanone 3′-*O*-*β*-D-(4′′-*O*-methyl)-glucopyranoside (**4d**)
Figure S79. COSY contour map – ^{1}H x ^{1}H expansion of 2′-methylflavanone 3′-*O*-*β*-D-(4′′-*O*-methyl)-glucopyranoside (**4d**)
Figure S80. COSY contour map – ^{1}H x ^{1}H expansion of 2′-methylflavanone 3′-*O*-*β*-D-(4′′-*O*-methyl)-glucopyranoside (**4d**)
Figure S81. HSQC contour map – ^{1}H x ^{13}C of 2′-methylflavanone 3′-*O*-*β*-D-(4′′-*O*-methyl)-glucopyranoside (**4d**)
Figure S82. HSQC contour map – ^{1}H x ^{13}C expansion of 2′-methylflavanone 3′-*O*-*β*-D-(4′′-*O*-methyl)-glucopyranoside (**4d**)
Figure S83. HSQC contour map – ^{1}H x ^{13}C expansion of 2′-methylflavanone 3′-*O*-*β*-D-(4′′-*O*-methyl)-glucopyranoside (**4d**)
Figure S84. HSQC contour map – ^{1}H x ^{13}C expansion of 2′-methylflavanone 3′-*O*-*β*-D-(4′′-*O*-methyl)-glucopyranoside (**4d**)

Figure S85. HMBC contour map – ^{1}H x ^{13}C of 2′-methylflavanone 3′-*O*-*β*-D-(4′′-*O*-methyl)-glucopyranoside (**4d**)
Figure S86. HMBC contour map – ^{1}H x ^{13}C expansion of 2′-methylflavanone 3′-*O*-*β*-D-(4′′-*O*-methyl)-glucopyranoside (**4d**)
Figure S87. HMBC contour map – ^{1}H x ^{13}C expansion of 2′-methylflavanone 3′-*O*-*β*-D-(4′′-*O*-methyl)-glucopyranoside (**4d**)
Figure S88. HMBC contour map – ^{1}H x ^{13}C expansion of 2′-methylflavanone 3′-*O*-*β*-D-(4′′-*O*-methyl)-glucopyranoside (**4d**)
Figure S89. MS analysis of 2-methylbenzoic acid 4-*O*-*β*-D-(4′-*O*-methyl)-glucopyranoside (**4e**)
Figure S90. ^{1}H NMR spectrum (*δ*, acetone-d6, 600 MHz) of 2-methylbenzoic acid 4-*O*-*β*-D-(4′-*O*-methyl)-glucopyranoside (**4e**)
Figure S91. ^{1}H NMR spectrum expansion (*δ*, acetone-d6, 600 MHz) of 2-methylbenzoic acid 4-*O*-*β*-D-(4′-*O*-methyl)-glucopyranoside (**4e**)
Figure S92. ^{1}H NMR spectrum expansion (*δ*, acetone-d6, 600 MHz) of 2-methylbenzoic acid 4-*O*-*β*-D-(4′-*O*-methyl)-glucopyranoside (**4e**)
Figure S93. ^{13}C NMR spectrum (*δ*, acetone-d6, 151 MHz) of 2-methylbenzoic acid 4-*O*-*β*-D-(4′-*O*-methyl)-glucopyranoside (**4e**)
Figure S94. ^{13}C NMR spectrum expansion (*δ*, acetone-d6, 151 MHz) of 2-methylbenzoic acid 4-*O*-*β*-D-(4′-*O*-methyl)-glucopyranoside (**4e**)
Figure S95. ^{13}C NMR spectrum expansion (*δ*, acetone-d6, 151 MHz) of 2-methylbenzoic acid 4-*O*-*β*-D-(4′-*O*-methyl)-glucopyranoside (**4e**)
Figure S96. COSY contour map – ^{1}H x ^{1}H of 2-methylbenzoic acid 4-*O*-*β*-D-(4′-*O*-methyl)-glucopyranoside (**4e**)
Figure S97. COSY contour map – ^{1}H x ^{1}H expansion of 2-methylbenzoic acid 4-*O*-*β*-D-(4′-*O*-methyl)-glucopyranoside (**4e**)
Figure S98. COSY contour map – ^{1}H x ^{1}H expansion of 2-methylbenzoic acid 4-*O*-*β*-D-(4′-*O*-methyl)-glucopyranoside (**4e**)
Figure S99. HSQC contour map – ^{1}H x ^{13}C 2-methylbenzoic acid 4-*O*-*β*-D-(4′-*O*-methyl)-glucopyranoside (**4e**)
Figure S100. HSQC contour map – ^{1}H x ^{13}C expansion of 2-methylbenzoic acid 4-*O*-*β*-D-(4′-*O*-methyl)-glucopyranoside (**4e**)
Figure S101. HSQC contour map – ^{1}H x ^{13}C expansion of 2-methylbenzoic acid 4-*O*-*β*-D-(4′-*O*-methyl)-glucopyranoside (**4e**)
Figure S102. HMBC contour map – ^{1}H x ^{13}C of 2-methylbenzoic acid 4-*O*-*β*-D-(4′-*O*-methyl)-glucopyranoside (**4e**)
Figure S103. HMBC contour map – ^{1}H x ^{13}C expansion of 2-methylbenzoic acid 4-*O*-*β*-D-(4′-*O*-methyl)-glucopyranoside (**4e**)
Figure S104. HMBC contour map – ^{1}H x ^{13}C expansion of 2-methylbenzoic acid 4-*O*-*β*-D-(4′-*O*-methyl)-glucopyranoside (**4e**)
Figure S105. MS analysis of 2′-methylflavone (**5**)

Figure S106. ^{1}H NMR spectrum (*δ*, acetone-d6, 600 MHz) of 2′-methylflavone (**5**)
Figure S107. ^{1}H NMR spectrum expansion (*δ*, acetone-d6, 600 MHz) 2′-methylflavone (**5**)
Figure S108. ^{13}C NMR spectrum (*δ*, acetone-d6, 151 MHz) of 2′-methylflavone (**5**)
Figure S109. ^{13}C NMR spectrum expansion (*δ*, acetone-d6, 151 MHz) of 2′-methylflavone (**5**)
Figure S110. COSY contour map – ^{1}H x ^{1}H of 2′-methylflavone (**5**)
Figure S111. COSY contour map – ^{1}H x ^{1}H expansion of 2′-methylflavone (**5**)
Figure S112. COSY contour map – ^{1}H x ^{1}H expansion of 2′-methylflavone (**5**)
Figure S113. HSQC contour map – ^{1}H x ^{13}C of 2′-methylflavone (**5**)
Figure S114. HSQC contour map – ^{1}H x ^{13}C expansion of 2′-methylflavone (**5**)
Figure S115. HSQC contour map – ^{1}H x ^{13}C expansion of 2′-methylflavone (**5**)
Figure S116. HMBC contour map – ^{1}H x ^{13}C of 2′-methylflavone (**5**)
Figure S117. HMBC contour map – ^{1}H x ^{13}C expansion of 2′-methylflavone (**5**)
Figure S118. HMBC contour map – ^{1}H x ^{13}C expansion of 2′-methylflavone (**5**)
Figure S119. MS analysis of 2′-methylflavone 3′-*O*-*β*-D-(4′′-*O*-methyl)-glucopyranoside (**5a**)
Figure S120. ^{1}H NMR spectrum (*δ*, acetone-d6, 600 MHz) of 2′-methylflavone 3′-*O*-*β*-D-(4′′-*O*-methyl)-glucopyranoside (**5a**)
Figure S121. ^{1}H NMR spectrum expansion (*δ*, acetone-d6, 600 MHz) of 2′-methylflavone 3′-*O*-*β*-D-(4′′-*O*-methyl)-glucopyranoside (**5a**)
Figure S122. ^{1}H NMR spectrum expansion (*δ*, acetone-d6, 600 MHz) of 2′-methylflavone 3′-*O*-*β*-D-(4′′-*O*-methyl)-glucopyranoside (**5a**)
Figure S123. ^{13}C NMR spectrum (*δ*, acetone-d6, 151 MHz) of 2′-methylflavone 3′-*O*-*β*-D-(4′′-*O*-methyl)-glucopyranoside (**5a**)
Figure S124. ^{13}C NMR spectrum expansion (*δ*, acetone-d6, 151 MHz) of 2′-methylflavone 3′-*O*-*β*-D-(4′′-*O*-methyl)-glucopyranoside (**5a**)
Figure S125. ^{13}C NMR spectrum expansion (*δ*, acetone-d6, 151 MHz) of 2′-methylflavone 3′-*O*-*β*-D-(4′′-*O*-methyl)-glucopyranoside (**5a**)
Figure S126. COSY contour map – ^{1}H x ^{1}H of 2′-methylflavone 3′-*O*-*β*-D-(4′′-*O*-methyl)-glucopyranoside (**5a**)
Figure S127. COSY contour map – ^{1}H x ^{1}H expansion of 2′-methylflavone 3′-*O*-*β*-D-(4′′-*O*-methyl)-glucopyranoside (**5a**)
Figure S128. COSY contour map – ^{1}H x ^{1}H expansion of 2′-methylflavone 3′-*O*-*β*-D-(4′′-*O*-methyl)-glucopyranoside (**5a**)
Figure S129. HSQC contour map – ^{1}H x ^{13}C of 2′-methylflavone 3′-*O*-*β*-D-(4′′-*O*-methyl)-glucopyranoside (**5a**)
Figure S130. HSQC contour map – ^{1}H x ^{13}C expansion of 2′-methylflavone 3′-*O*-*β*-D-(4′′-*O*-methyl)-glucopyranoside (**5a**)
Figure S131. HSQC contour map – ^{1}H x ^{13}C expansion of 2′-methylflavone 3′-*O*-*β*-D-(4′′-*O*-methyl)-glucopyranoside (**5a**)
Figure S132. HMBC contour map – ^{1}H x ^{13}C expansion of 2′-methylflavone 3′-*O*-*β*-D-(4′′-*O*-methyl)-glucopyranoside (**5a**)

Figure S133. HMBC contour map – ^{1}H x ^{13}C expansion of 2′-methylflavone 3′-*O*-*β*-D-(4′′-*O*-methyl)-glucopyranoside (**5a**)
Figure S134. HMBC contour map – ^{1}H x ^{13}C expansion of 2′-methylflavone 3′-*O*-*β*-D-(4′′-*O*-methyl)-glucopyranoside (**5a**)
Figure S135. MS analysis of 2′-methylflavone 4′-*O*-*β*-D-(4′′-*O*-methyl)-glucopyranoside (**5b**)
Figure S136. ^{1}H NMR spectrum (*δ*, acetone-d6, 600 MHz) of 2′-methylflavone 4′-*O*-*β*-D-(4′′-*O*-methyl)-glucopyranoside (**5b**)
Figure S137. ^{1}H NMR spectrum expansion (*δ*, acetone-d6, 600 MHz) of 2′-methylflavone 4′-*O*-*β*-D-(4′′-*O*-methyl)-glucopyranoside (**5b**)
Figure S138. ^{1}H NMR spectrum expansion (*δ*, acetone-d6, 600 MHz) of 2′-methylflavone 4′-*O*-*β*-D-(4′′-*O*-methyl)-glucopyranoside (**5b**)
Figure S139. ^{13}C NMR spectrum of (*δ*, acetone-d6, 151 MHz) 2′-methylflavone 4′-*O*-*β*-D-(4′′-*O*-methyl)-glucopyranoside (**5b**)
Figure S140. ^{13}C NMR spectrum expansion (*δ*, acetone-d6, 151 MHz) of 2′-methylflavone 4′-*O*-*β*-D-(4′′-*O*-methyl)-glucopyranoside (**5b**)
Figure S141. ^{13}C NMR spectrum expansion (*δ*, acetone-d6, 151 MHz) of 2′-methylflavone 4′-*O*-*β*-D-(4′′-*O*-methyl)-glucopyranoside (**5b**)
Figure S142. COSY contour map – ^{1}H x ^{1}H 2′-methylflavone 4′-*O*-*β*-D-(4′′-*O*-methyl)-glucopyranoside (**5b**)
Figure S143. COSY contour map – ^{1}H x ^{1}H expansion of 2′-methylflavone 4′-*O*-*β*-D-(4′′-*O*-methyl)-glucopyranoside (**5b**)
Figure S144. COSY contour map – ^{1}H x ^{1}H expansion of 2′-methylflavone 4′-*O*-*β*-D-(4′′-*O*-methyl)-glucopyranoside (**5b**)
Figure S145. HSQC contour map – ^{1}H x ^{13}C of 2′-methylflavone 4′-*O*-*β*-D-(4′′-*O*-methyl)-glucopyranoside (**5b**)
Figure S146. HSQC contour map – ^{1}H x ^{13}C expansion of 2′-methylflavone 4′-*O*-*β*-D-(4′′-*O*-methyl)-glucopyranoside (**5b**)
Figure S147. HSQC contour map – ^{1}H x ^{13}C expansion of 2′-methylflavone 4′-*O*-*β*-D-(4′′-*O*-methyl)-glucopyranoside (**5b**)
Figure S148. HMBC contour map – ^{1}H x ^{13}C of 2′-methylflavone 4′-*O*-*β*-D-(4′′-*O*-methyl)-glucopyranoside (**5b**)
Figure S149. HMBC contour map – ^{1}H x ^{13}C expansion of 2′-methylflavone 4′-*O*-*β*-D-(4′′-*O*-methyl)-glucopyranoside (**5b**)
Figure S150. HMBC contour map – ^{1}H x ^{13}C expansion of 2′-methylflavone 4′-*O*-*β*-D-(4′′-*O*-methyl)-glucopyranoside (**5b**)
Figure S151. HMBC contour map – ^{1}H x ^{13}C expansion of 2′-methylflavone 4′-*O*-*β*-D-(4′′-*O*-methyl)-glucopyranoside (**5b**)
Figure S152. MS analysis of 2′-methylflavone 5′-*O*-*β*-D-(4′′-*O*-methyl)-glucopyranoside (**5c**)
Figure S153. ^{1}H NMR spectrum (*δ*, acetone-d6, 600 MHz) of 2′-methylflavone 5′-*O*-*β*-D-(4′′-*O*-methyl)-glucopyranoside (**5c**)

Figure S154. ^{1}H NMR spectrum expansion (*δ,* acetone-d6, 600 MHz) of 2′-methylflavone 5′-*O*-*β*-D-(4′′-*O*-methyl)-glucopyranoside (**5c**)
Figure S155. ^{1}H NMR spectrum expansion (*δ,* acetone-d6, 600 MHz) of 2′-methylflavone 5′-*O*-*β*-D-(4′′-*O*-methyl)-glucopyranoside (**5c**)
Figure S156. ^{13}C NMR spectrum (*δ,* acetone-d6, 151 MHz) of 2′-methylflavone 5′-*O*-*β*-D-(4′′-*O*-methyl)-glucopyranoside (**5c**)
Figure S157. ^{13}C NMR spectrum expansion (*δ,* acetone-d6, 151 MHz) of 2′-methylflavone 5′-*O*-*β*-D-(4′′-*O*-methyl)-glucopyranoside (**5c**)
Figure S158. ^{13}C NMR spectrum expansion (*δ,* acetone-d6, 151 MHz) of 2′-methylflavone 5′-*O*-*β*-D-(4′′-*O*-methyl)-glucopyranoside (**5c**)
Figure S159. COSY contour map – ^{1}H x ^{1}H of 2′-methylflavone 5′-*O*-*β*-D-(4′′-*O*-methyl)-glucopyranoside (**5c**)
Figure S160. COSY contour map – ^{1}H x ^{1}H expansion of 2′-methylflavone 5′-*O*-*β*-D-(4′′-*O*-methyl)-glucopyranoside (**5c**)
Figure S161. COSY contour map – ^{1}H x ^{1}H expansion of 2′-methylflavone 5′-*O*-*β*-D-(4′′-*O*-methyl)-glucopyranoside (**5c**)
Figure S162. HSQC contour map – ^{1}H x ^{13}C of 2′-methylflavone 5′-*O*-*β*-D-(4′′-*O*-methyl)-glucopyranoside (**5c**)
Figure S163. HSQC contour map – ^{1}H x ^{13}C expansion of 2′-methylflavone 5′-*O*-*β*-D-(4′′-*O*-methyl)-glucopyranoside (**5c**)
Figure S164. HSQC contour map – ^{1}H x ^{13}C expansion of 2′-methylflavone 5′-*O*-*β*-D-(4′′-*O*-methyl)-glucopyranoside (**5c**)
Figure S165. HMBC contour map – ^{1}H x ^{13}C of 2′-methylflavone 5′-*O*-*β*-D-(4′′-*O*-methyl)-glucopyranoside (**5c**)
Figure S166. HMBC contour map – ^{1}H x ^{13}C expansion of 2′-methylflavone 5′-*O*-*β*-D-(4′′-*O*-methyl)-glucopyranoside (**5c**)
Figure S167. HMBC contour map – ^{1}H x ^{13}C expansion of 2′-methylflavone 5′-*O*-*β*-D-(4′′-*O*-methyl)-glucopyranoside (**5c**)

Molecular formula: $C_{16}H_{14}O_2$

Formula weight: 238.10

Ionization mode: positive

Precursor: $[M + H]^+$ 239.30

239.3000>121.1000 CE (Collision Energy): -22.0

239.3000>224.1000 CE: -17.0

239.3000>198.1500 CE: -8.0

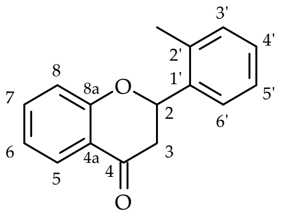

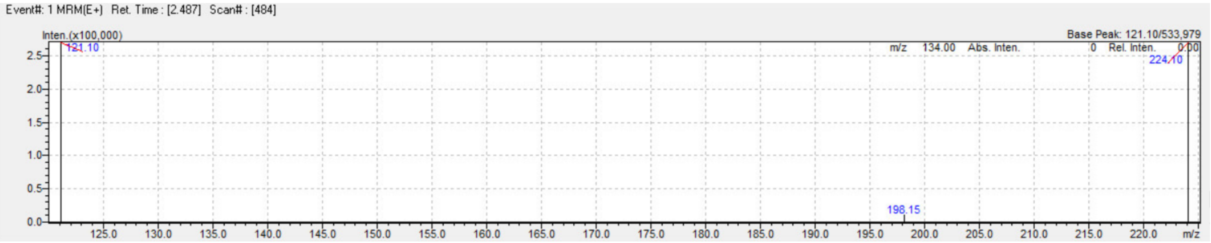

Figure S1. MS analysis of 2′-methyflavanone (**4**)

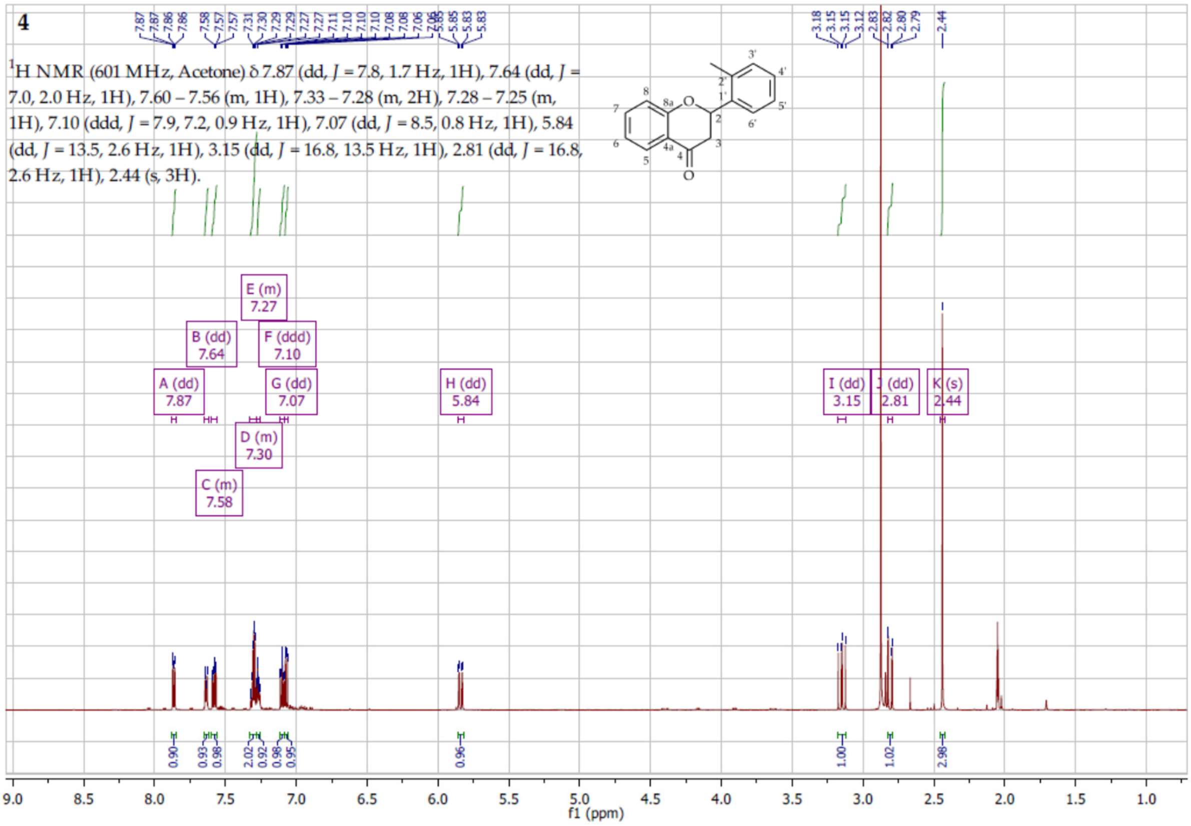

Figure S2. ¹H NMR spectrum (δ, acetone-d6, 600 MHz) of 2′-methyflavanone (**4**)

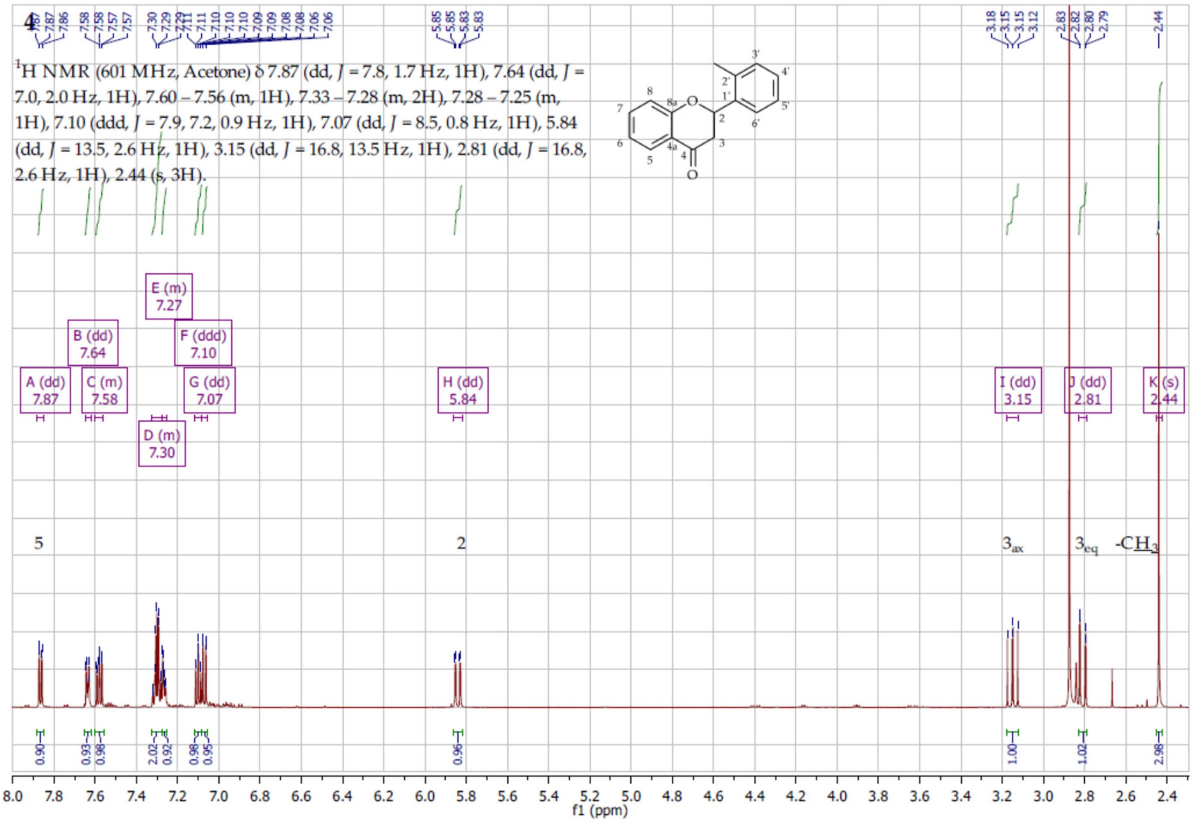

Figure S3 ^{1}H NMR spectrum expansion (δ, acetone-d6, 600 MHz) of 2′-methyflavanone (**4**)

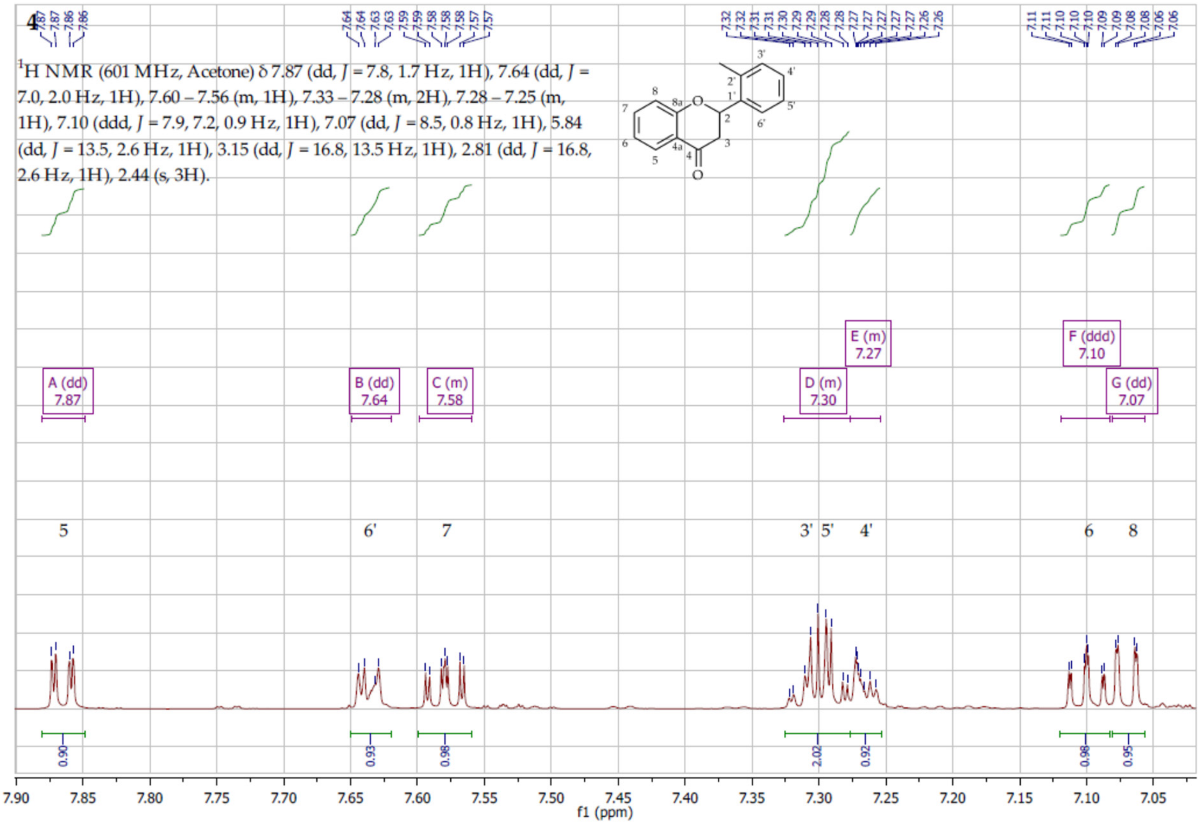

Figure S4. ^{1}H NMR spectrum expansion (δ, acetone-d6, 600 MHz) of 2′-methyflavanone (**4**)

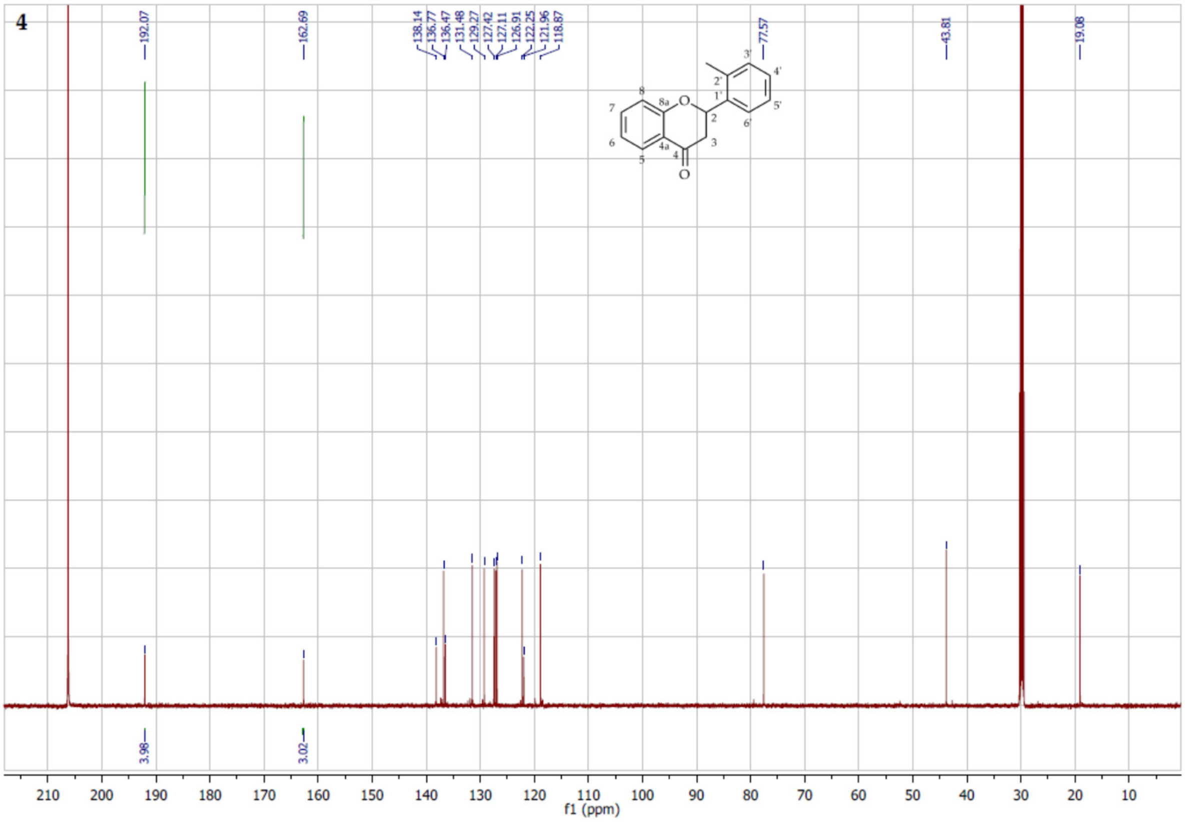

Figure S5. ^{13}C NMR spectrum (δ, acetone-d6, 151 MHz) of 2′-methyflavanone (**4**)

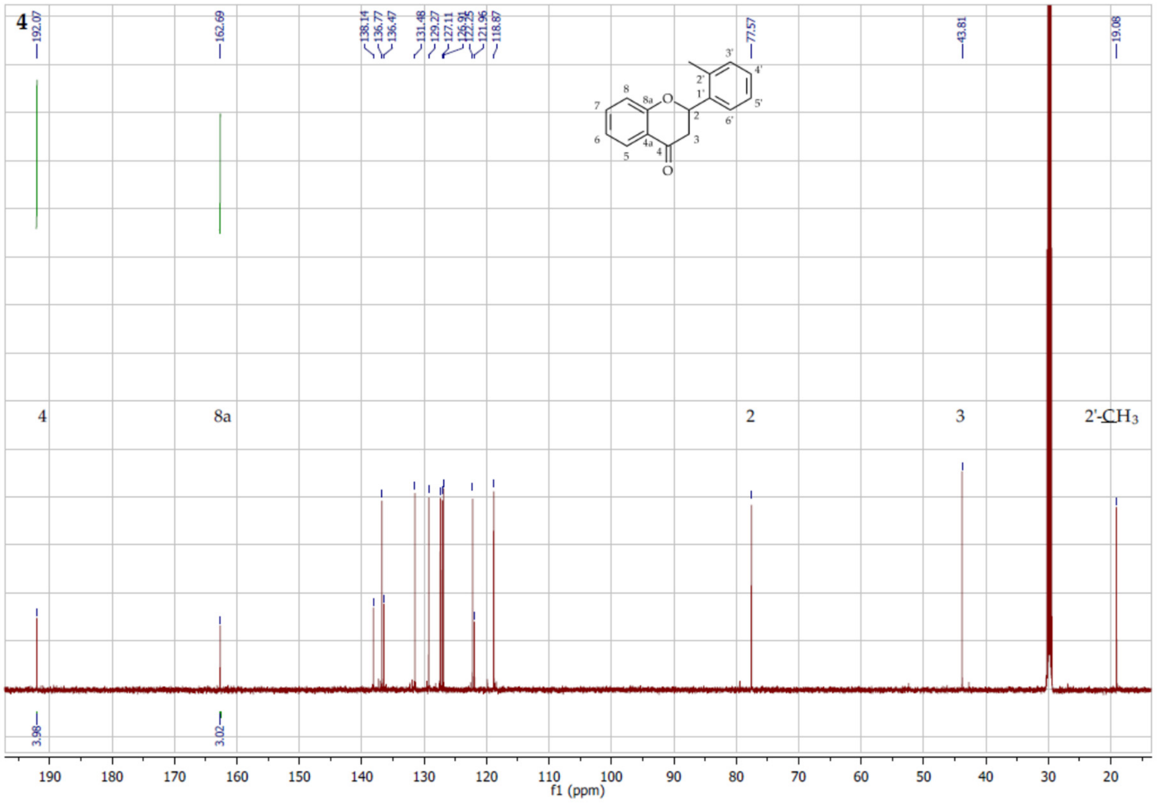

Figure S6. ^{13}C NMR spectrum expansion (δ, acetone-d6, 151 MHz) of 2′-methyflavanone (**4**)

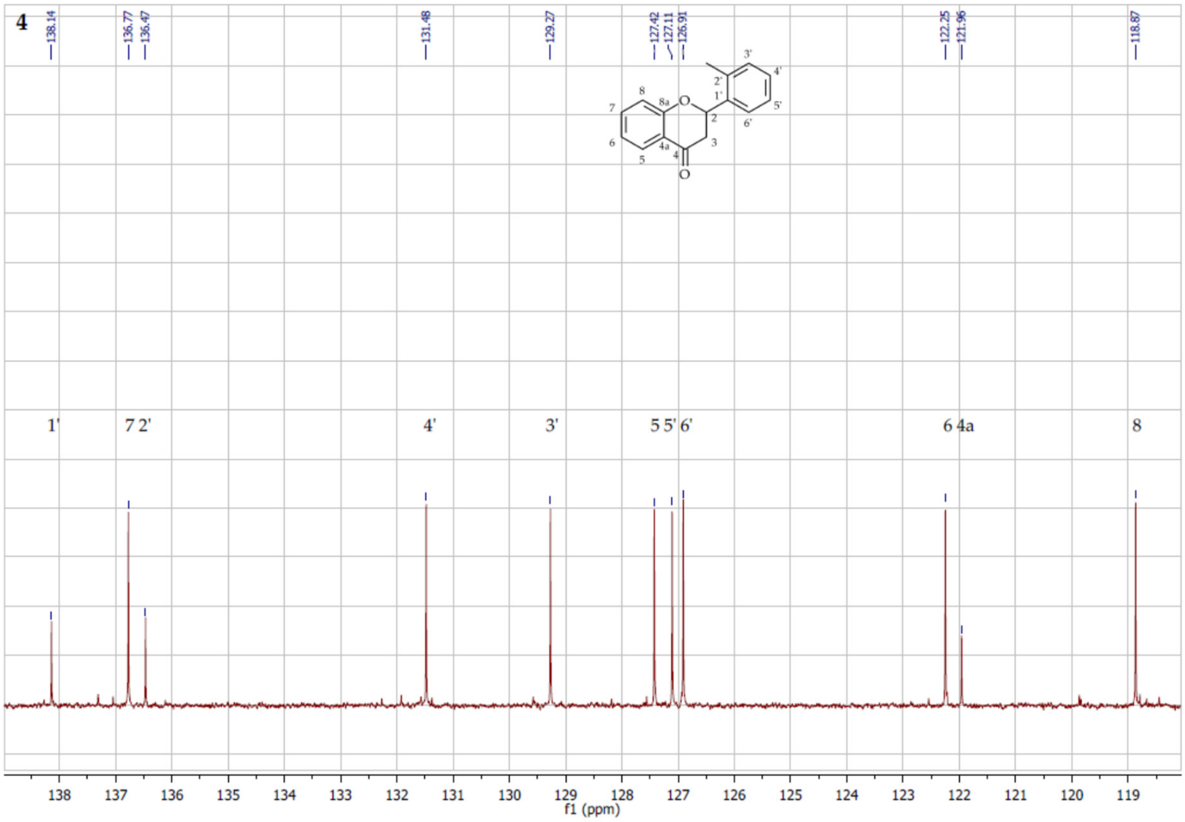

Figure S7. ^{13}C NMR spectrum expansion (δ, acetone-d6, 151 MHz) of 2′-methyflavanone (**4**)

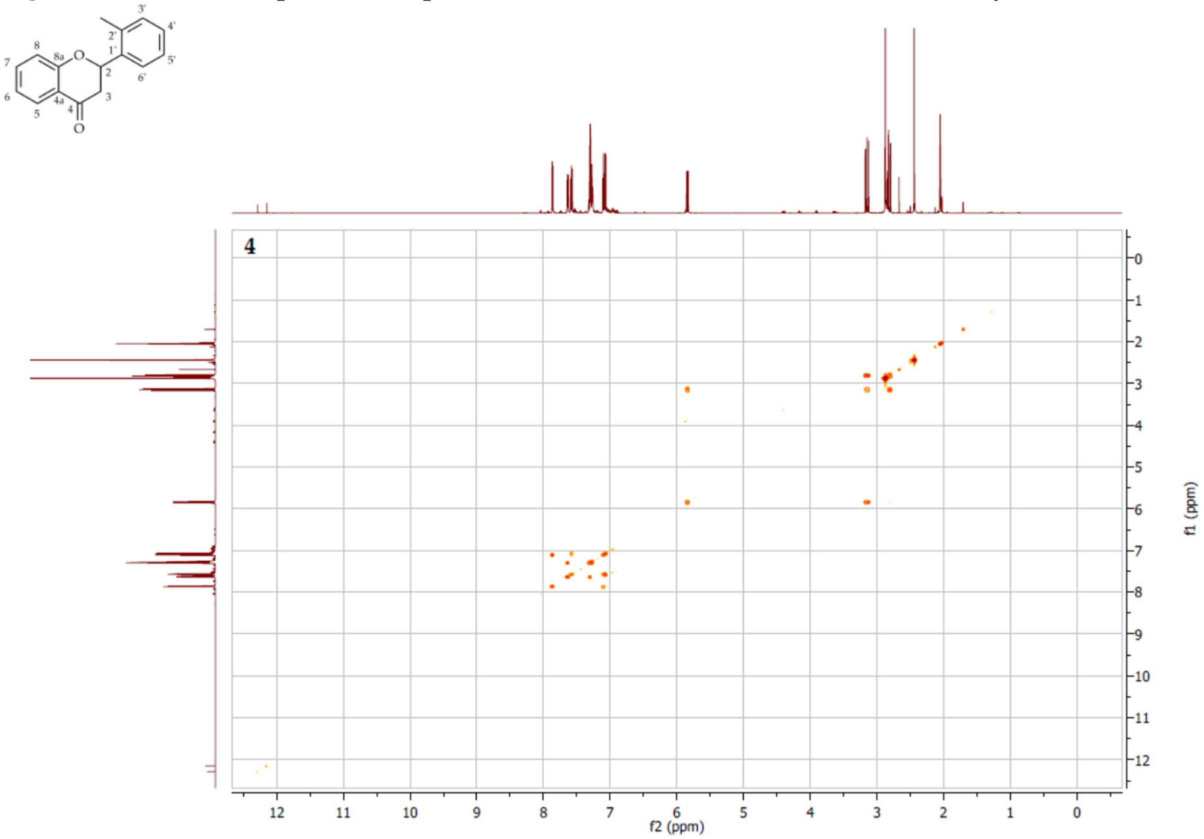

Figure S8. COSY contour map – ^{1}H x ^{1}H of 2′-methyflavanone (**4**)

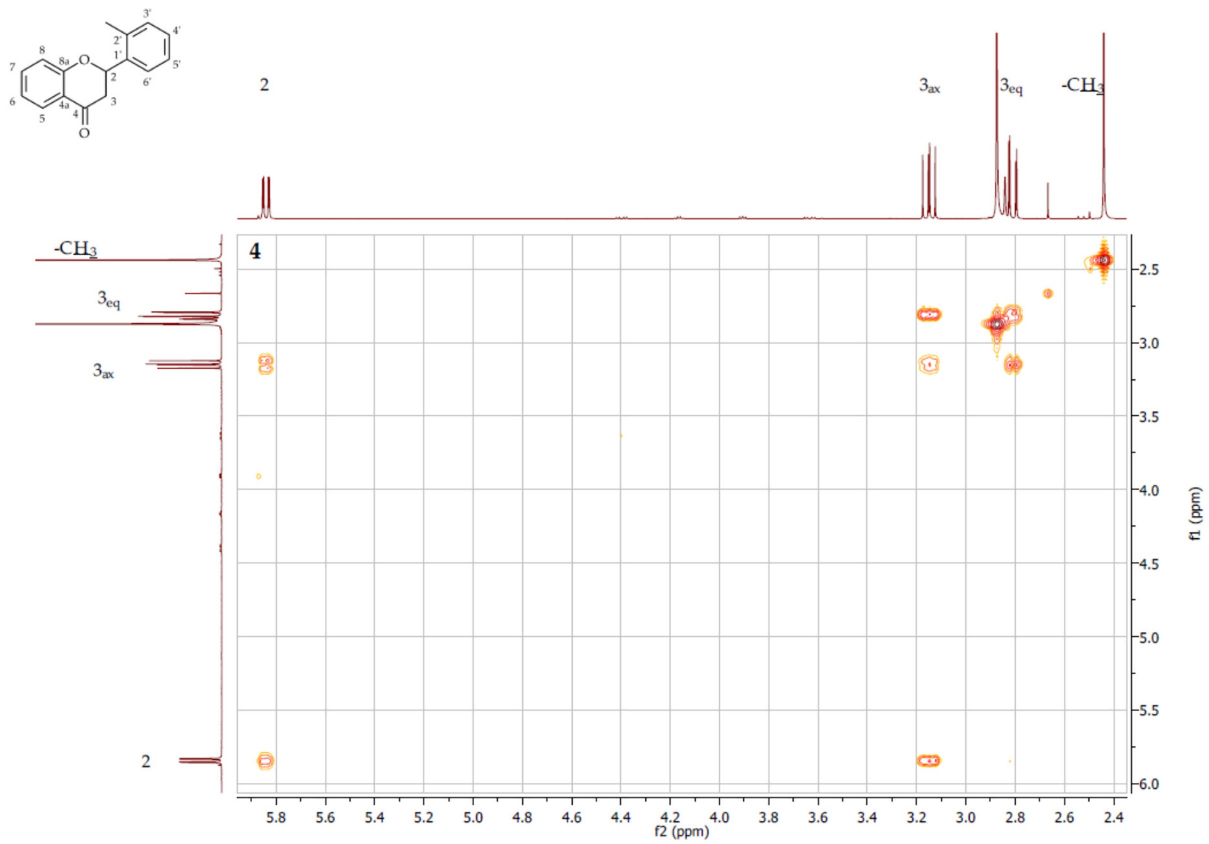

Figure S9. COSY contour map – ^{1}H x ^{1}H expansion of 2′-methyflavanone (**4**)

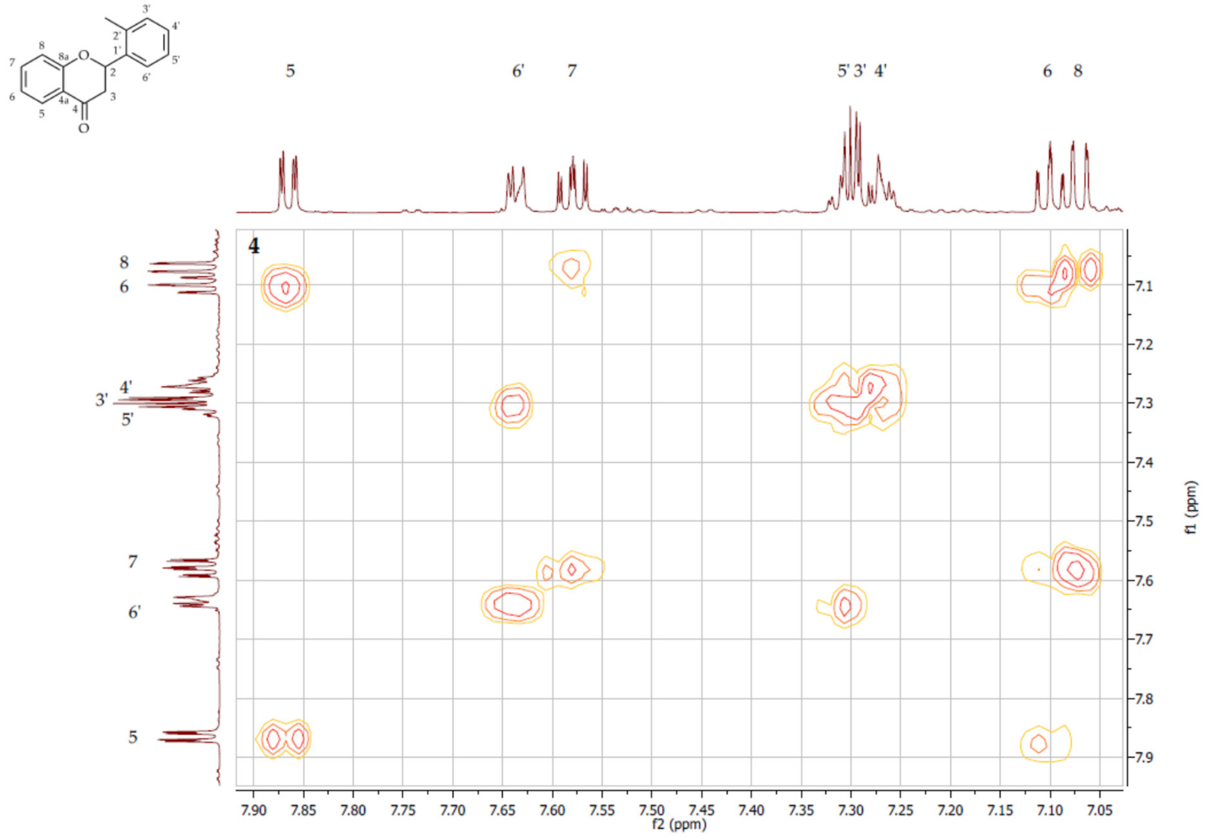

Figure S10. COSY contour map – ^{1}H x ^{1}H expansion of 2′-methyflavanone (**4**)

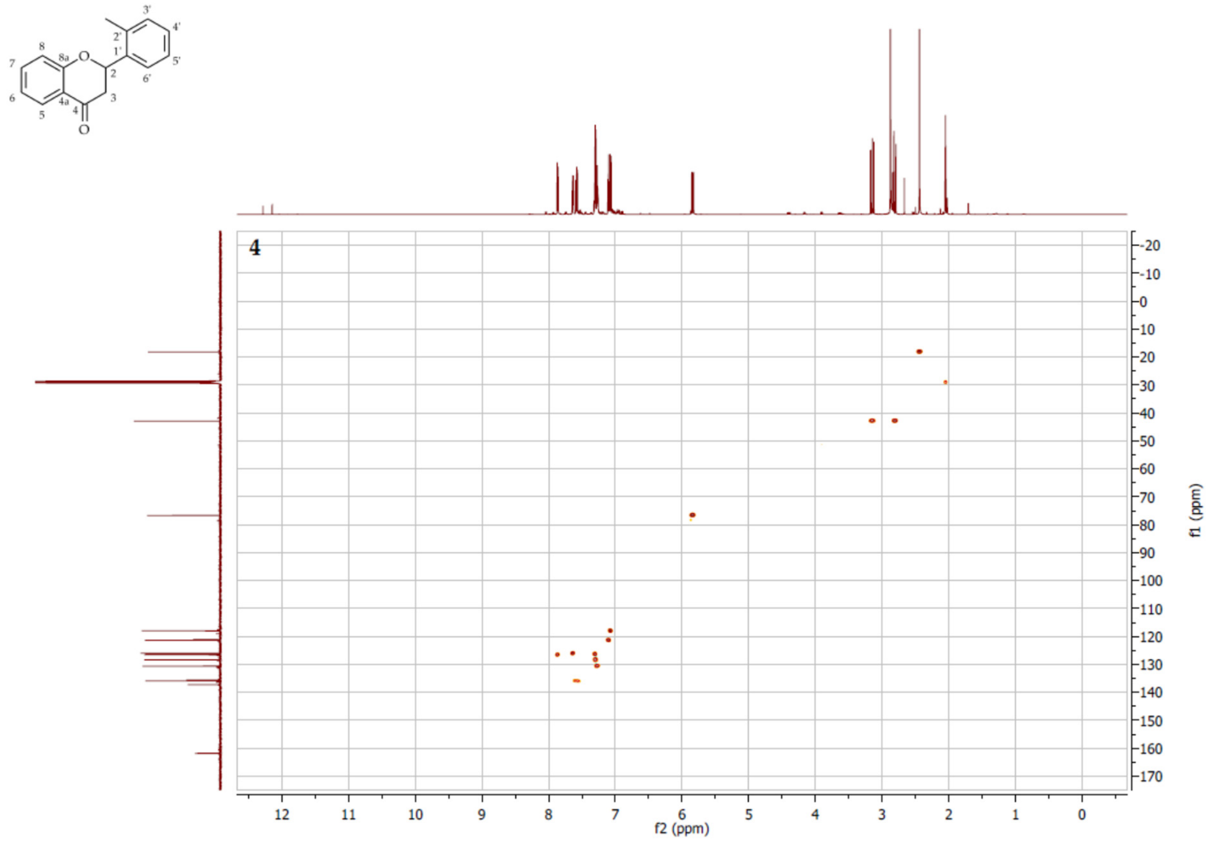

Figure S11. HSQC contour map – ^{1}H x ^{13}C of 2′-methyflavanone (**4**)

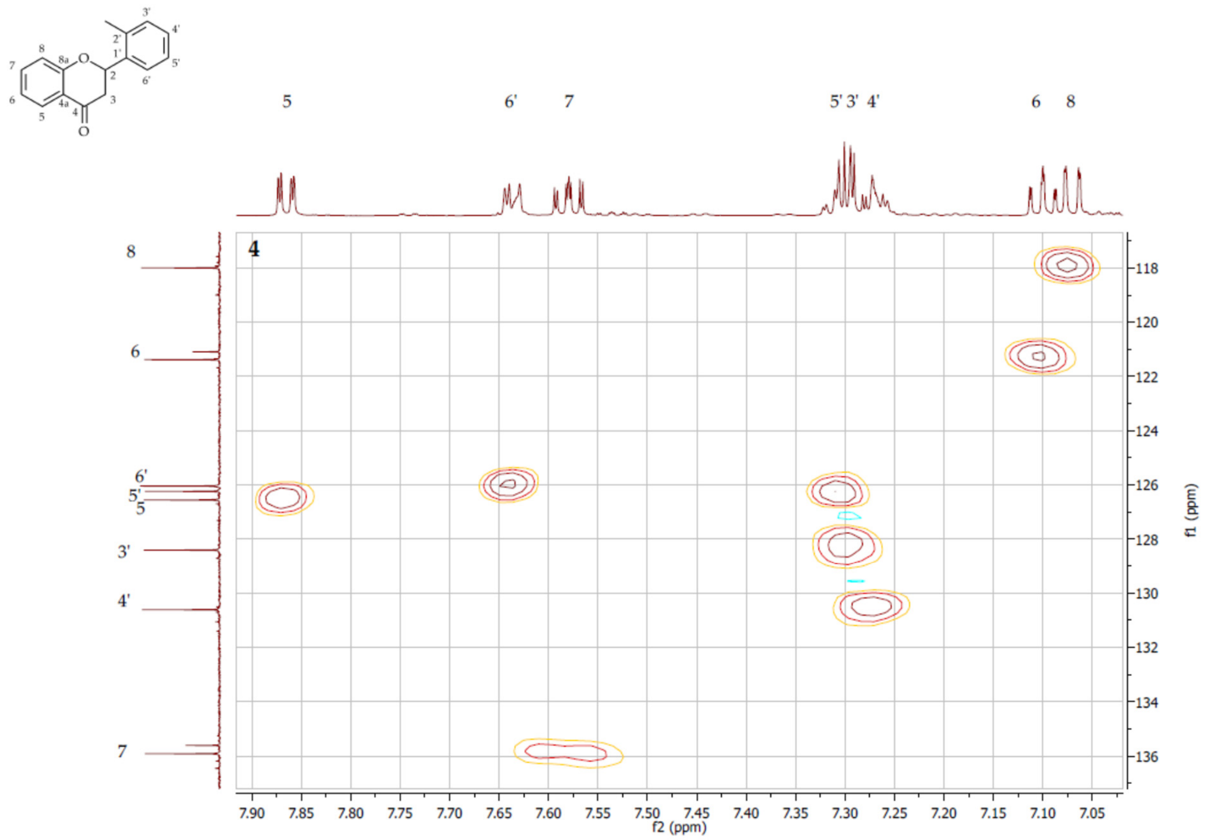

Figure S12. HSQC contour map – ^{1}H x ^{13}C expansion of 2′-methyflavanone (**4**)

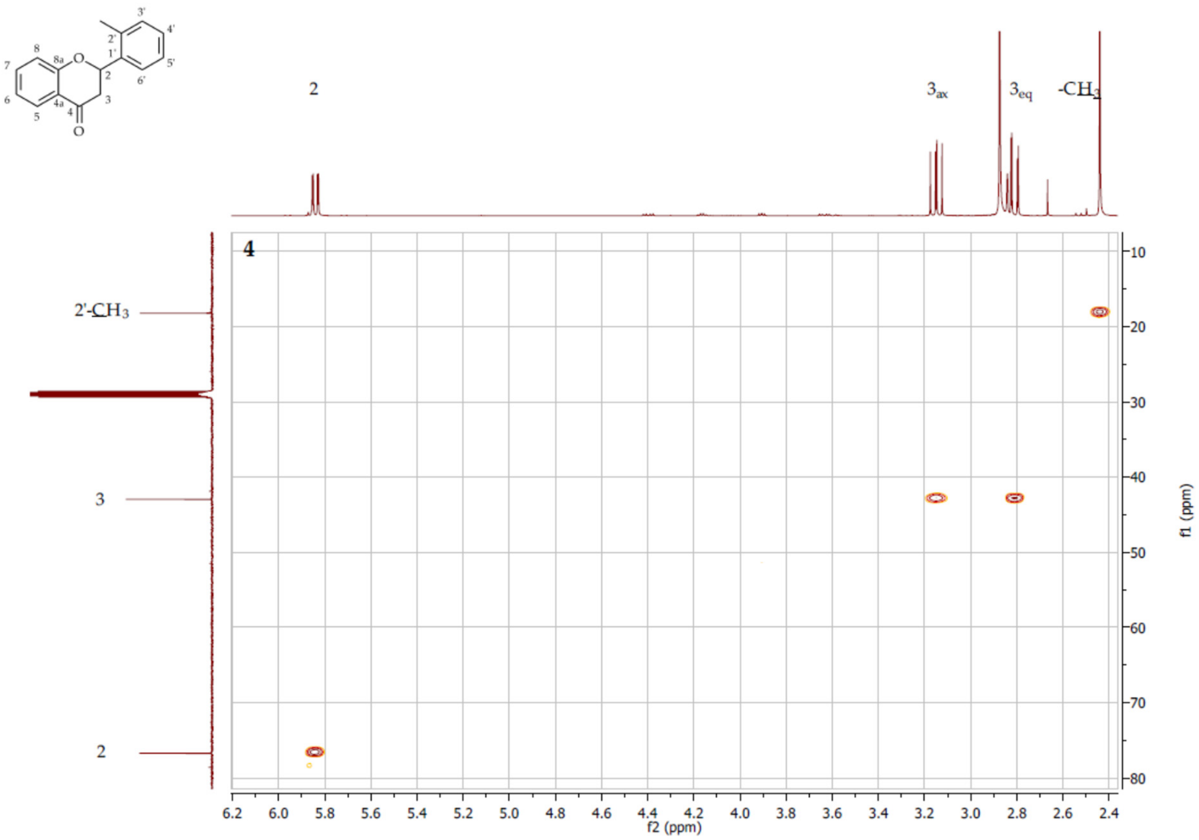

Figure S13. HSQC contour map – ^{1}H x ^{13}C expansion of 2′-methyflavanone (**4**)

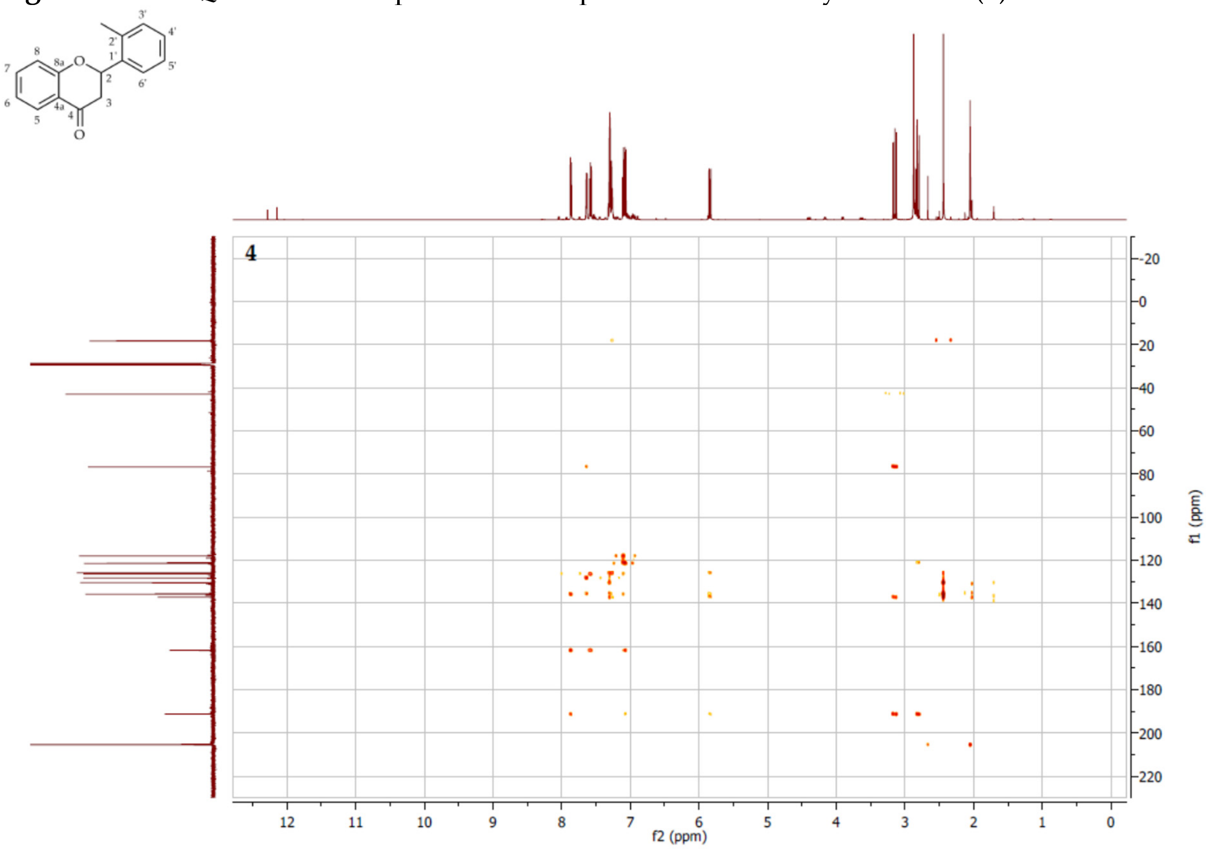

Figure S14. HMBC contour map – ^{1}H x ^{13}C of 2′-methyflavanone (**4**)

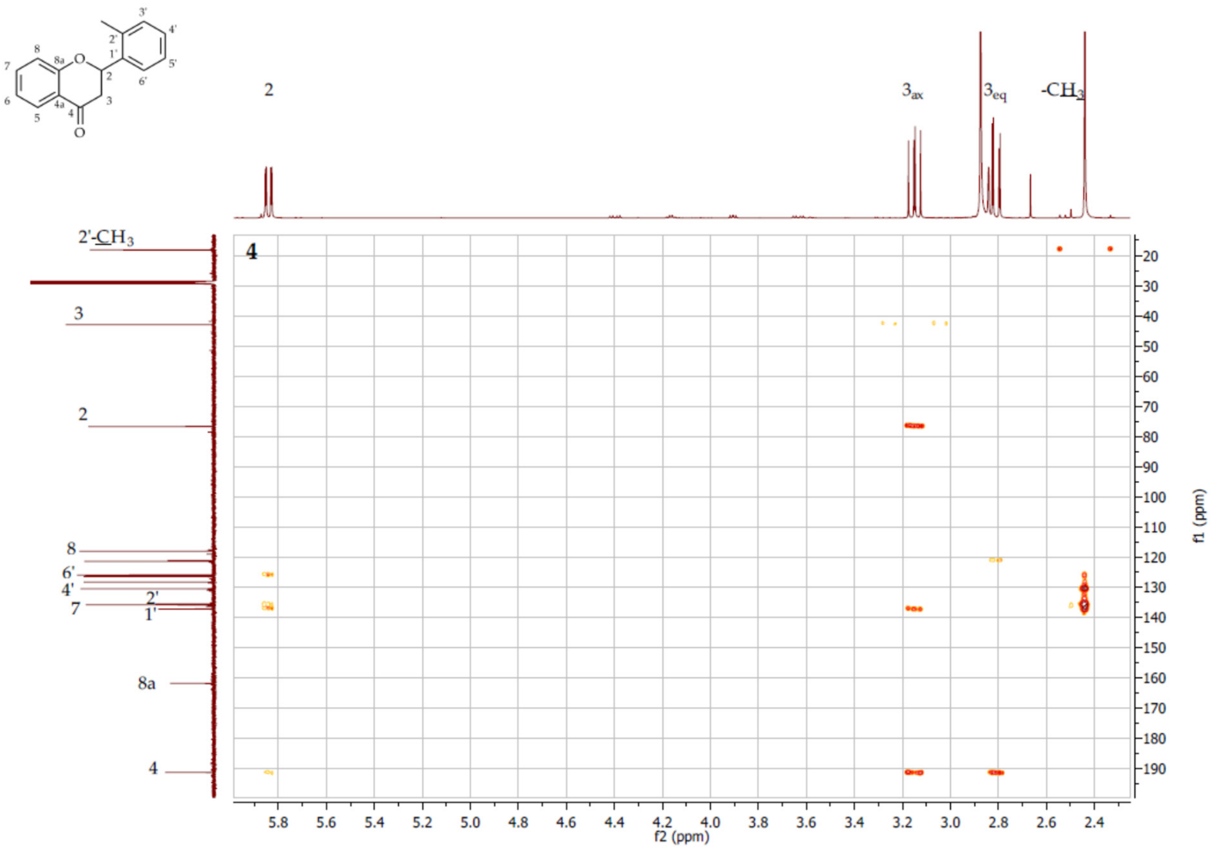

Figure S15. HMBC contour map – ^{1}H x ^{13}C expansion of 2′-methyflavanone (**4**)

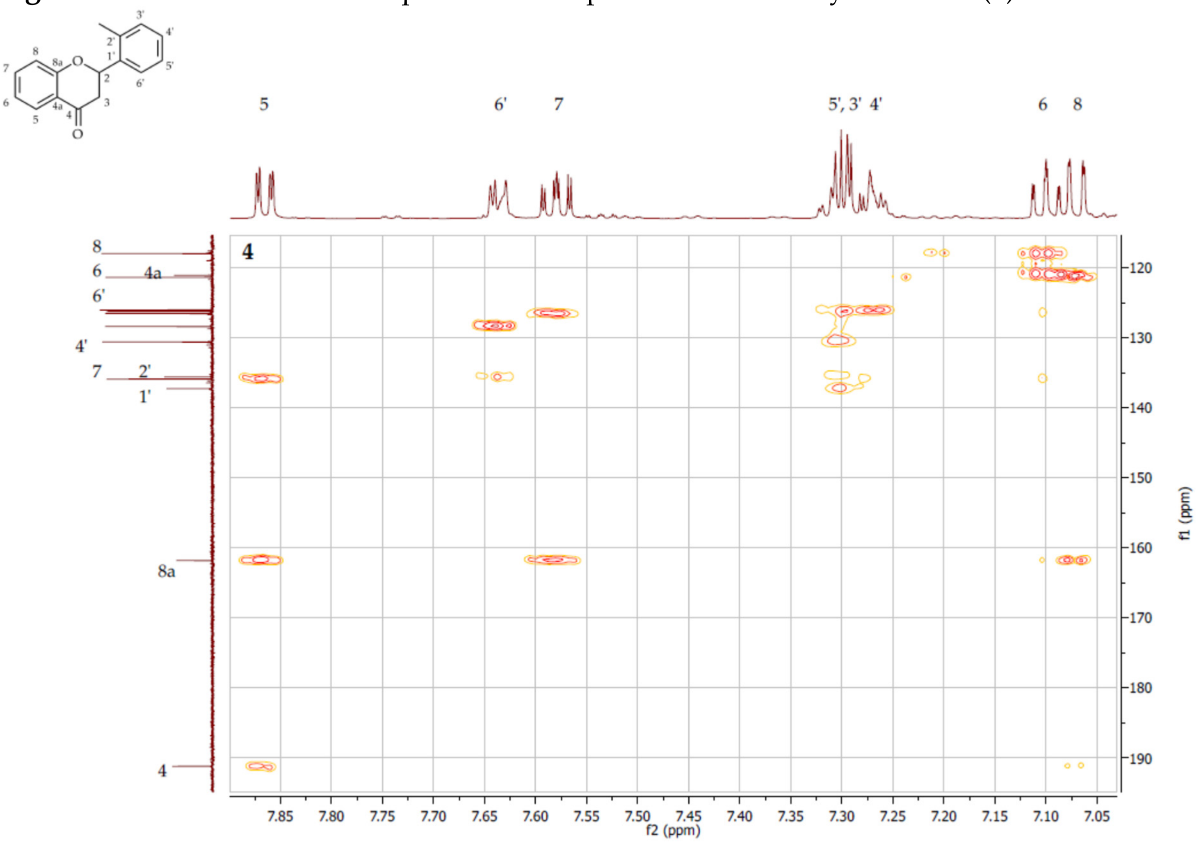

Figure S16. HMBC contour map – ^{1}H x ^{13}C expansion of 2′-methyflavanone (**4**)

Molecular formula: $C_{23}H_{26}O_8$
Formula weight: 430.16
Ionization mode: positive
Precursor: $[M + H]^+$ 430.70
430.7000>311.2500 CE: -24.0
430.7000>226.8500 CE: -17.0
430.7000>362.9000 CE: -10.0

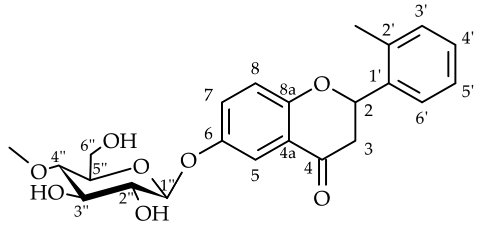

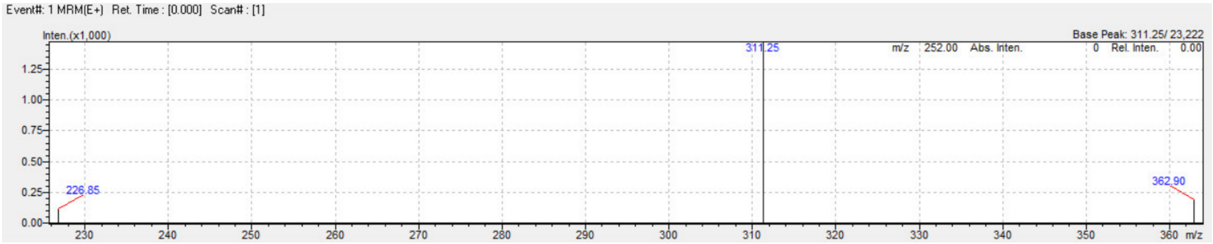

Figure S17. MS analysis of 2′-methylflavanone 6-*O*-*β*-D-(4′′-*O*-methyl)-glucopyranoside (**4a**)

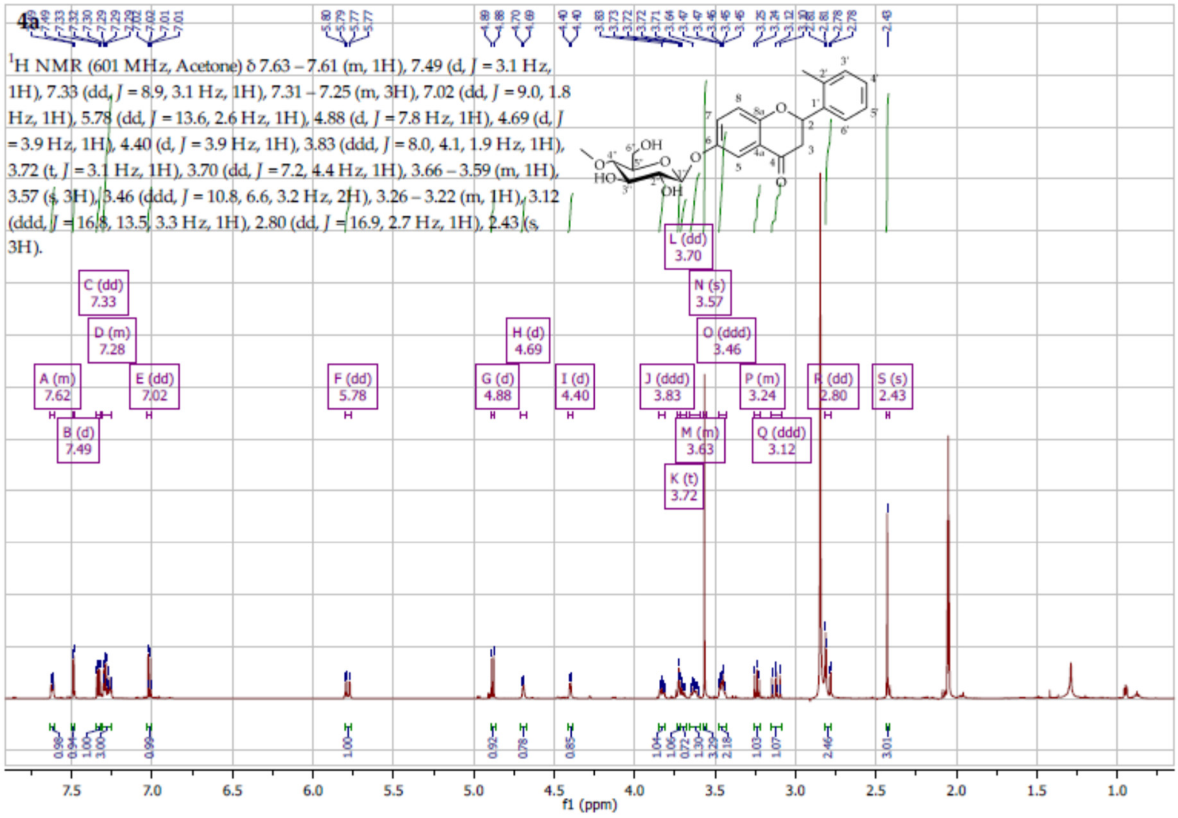

Figure S18. ^{1}H NMR spectrum (*δ*, acetone-d6, 600 MHz) of 2′-methylflavanone 6-*O*-*β*-D-(4′′-*O*-methyl)-glucopyranoside (**4a**)

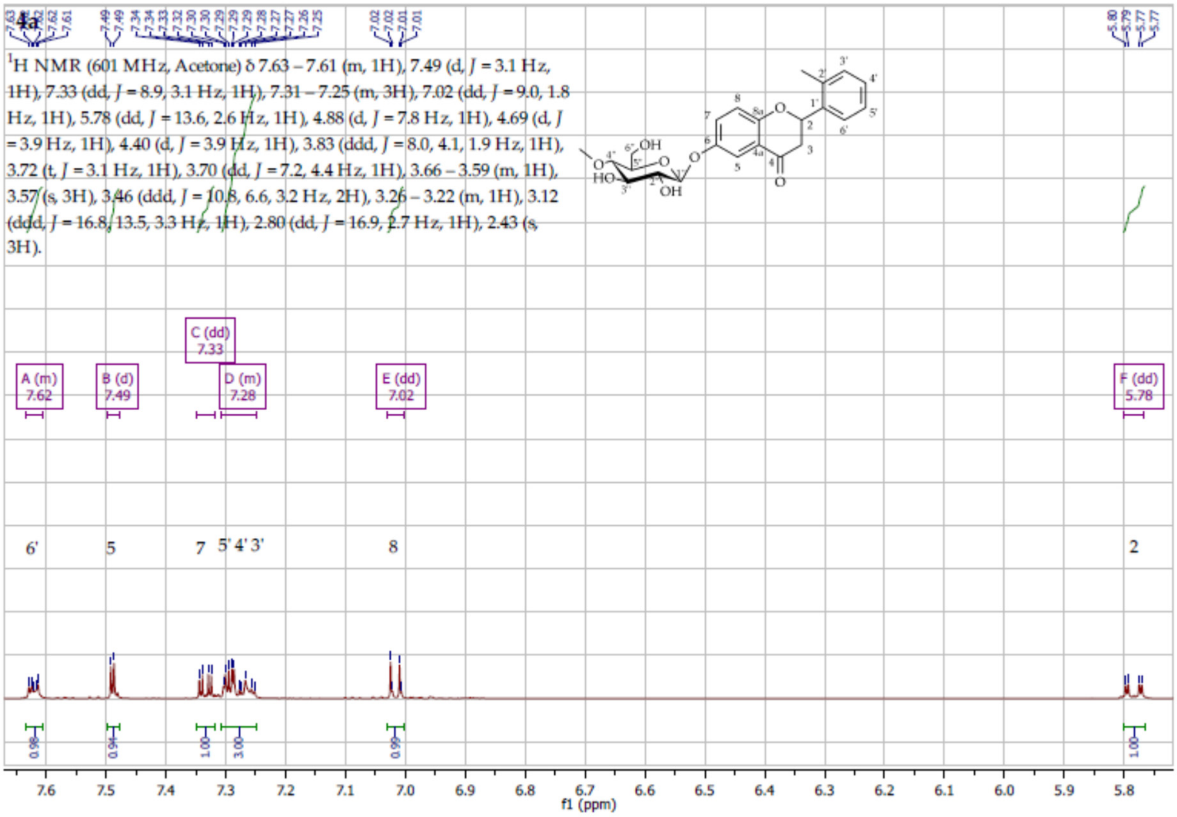

Figure S19. ^{1}H NMR spectrum expansion (*δ*, acetone-d6, 600 MHz) of 2′-methylflavanone 6-*O*-*β*-D-(4′′-*O*-methyl)-glucopyranoside (**4a**)

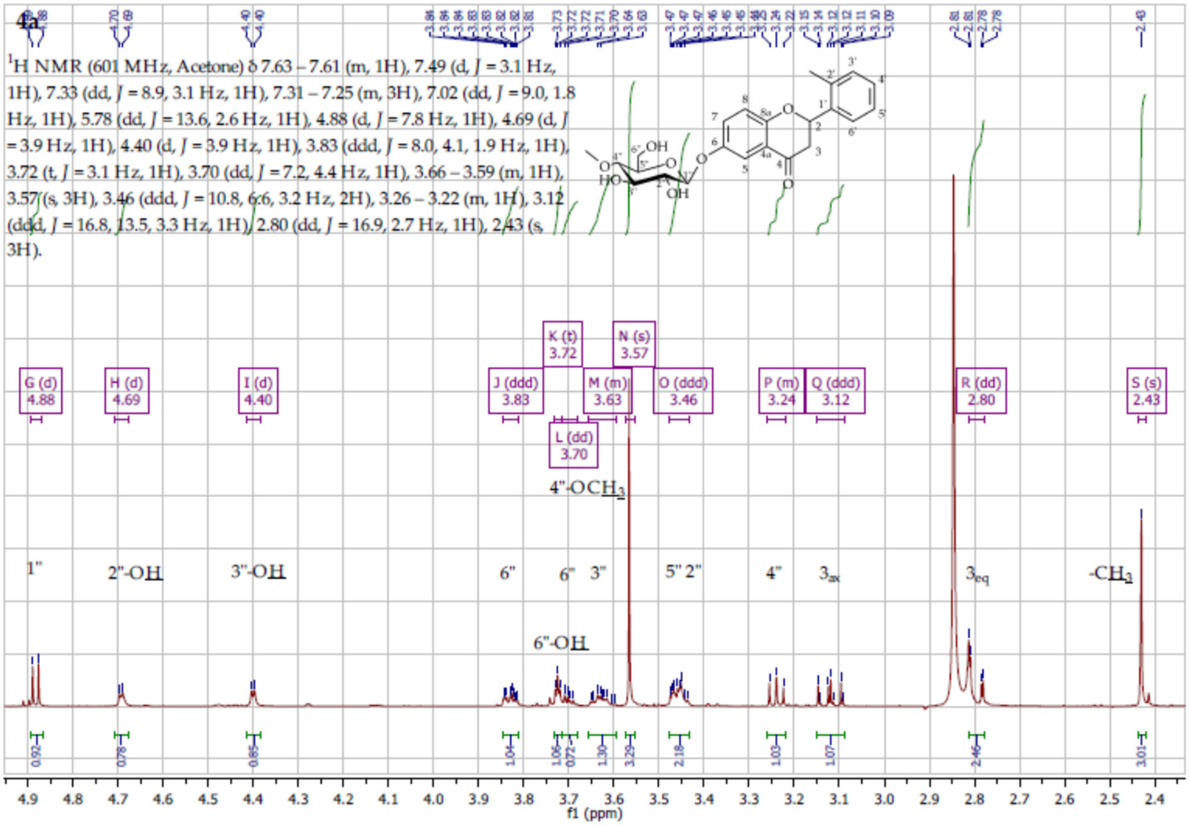

Figure S20. ^{1}H NMR spectrum expansion (*δ*, acetone-d6, 600 MHz) of 2′-methylflavanone 6-*O*-*β*-D-(4′′-*O*-methyl)-glucopyranoside (**4a**)

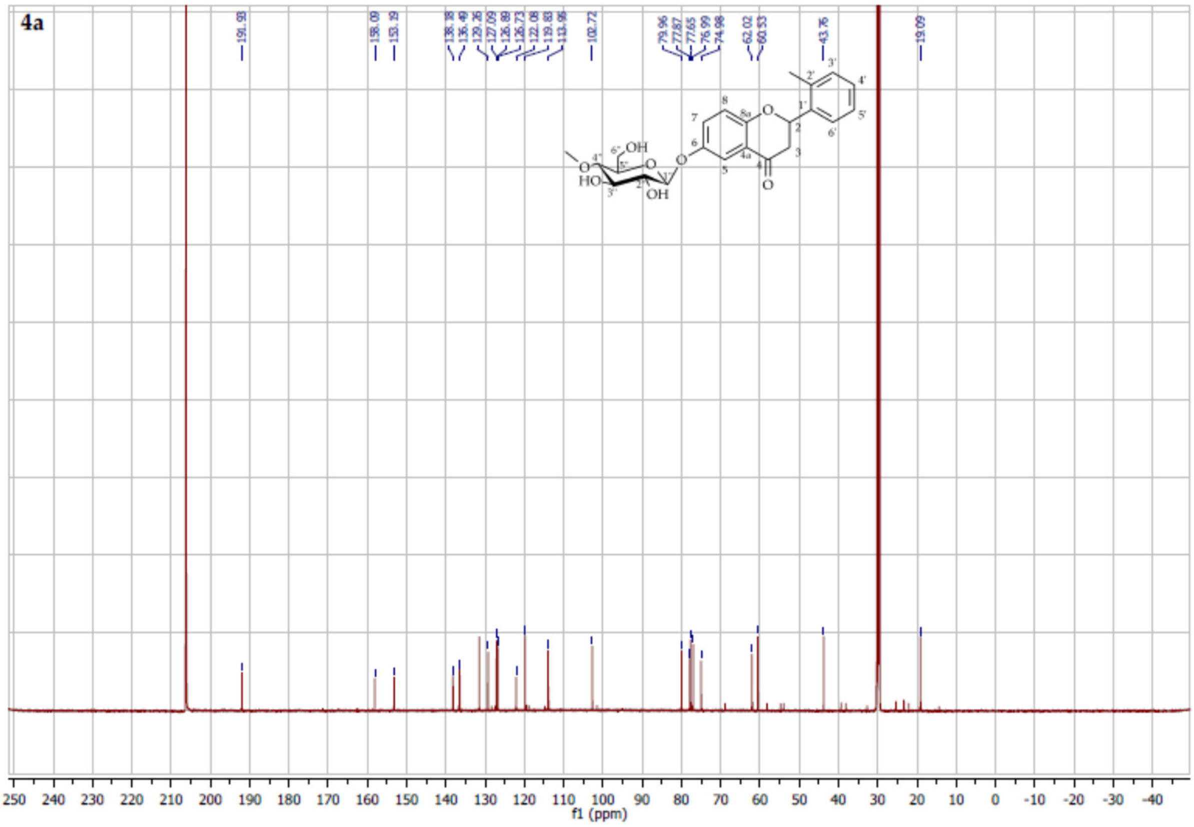

Figure S21. ^{13}C NMR spectrum expansion (*δ*, acetone-d6, 151 MHz) of 2′-methylflavanone 6-*O*-*β*-D-(4′′-*O*-methyl)-glucopyranoside (**4a**)

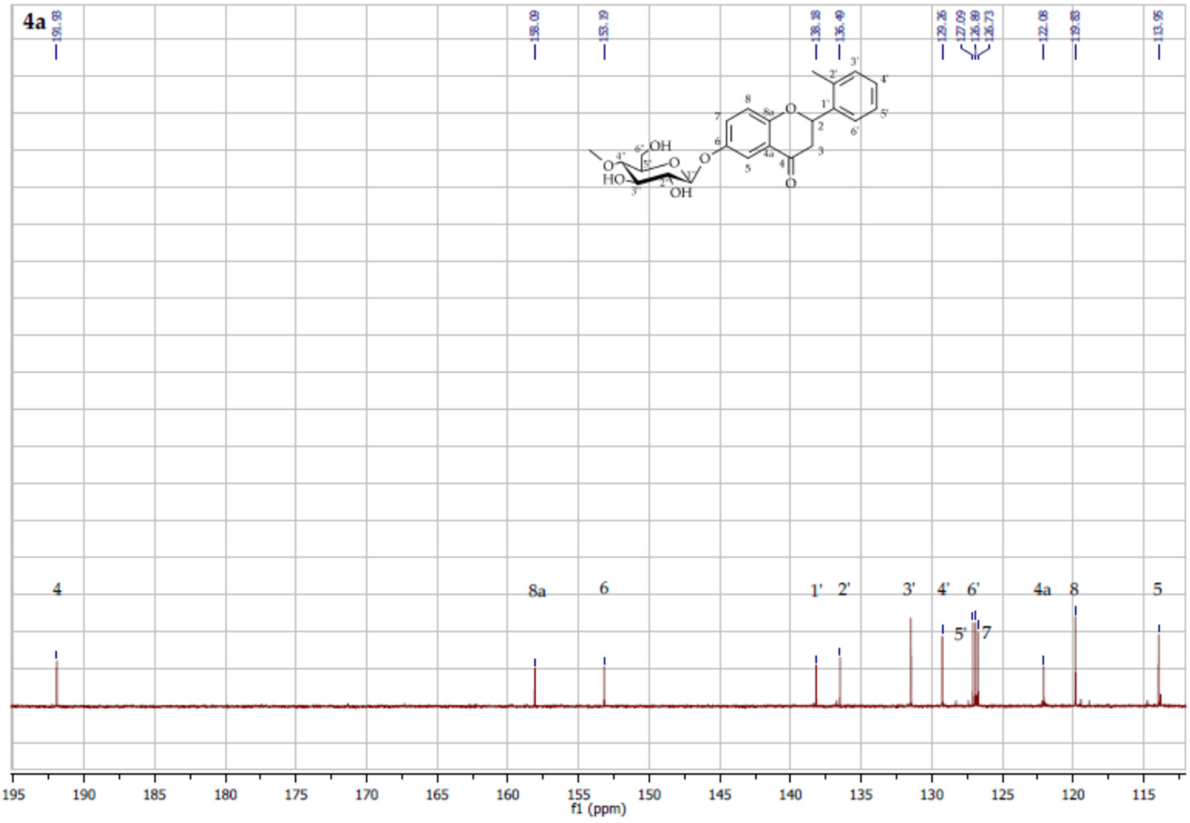

Figure S22. ^{13}C NMR spectrum expansion (*δ*, acetone-d6, 151 MHz) of 2′-methylflavanone 6-*O*-*β*-D-(4′′-*O*-methyl)-glucopyranoside (**4a**)

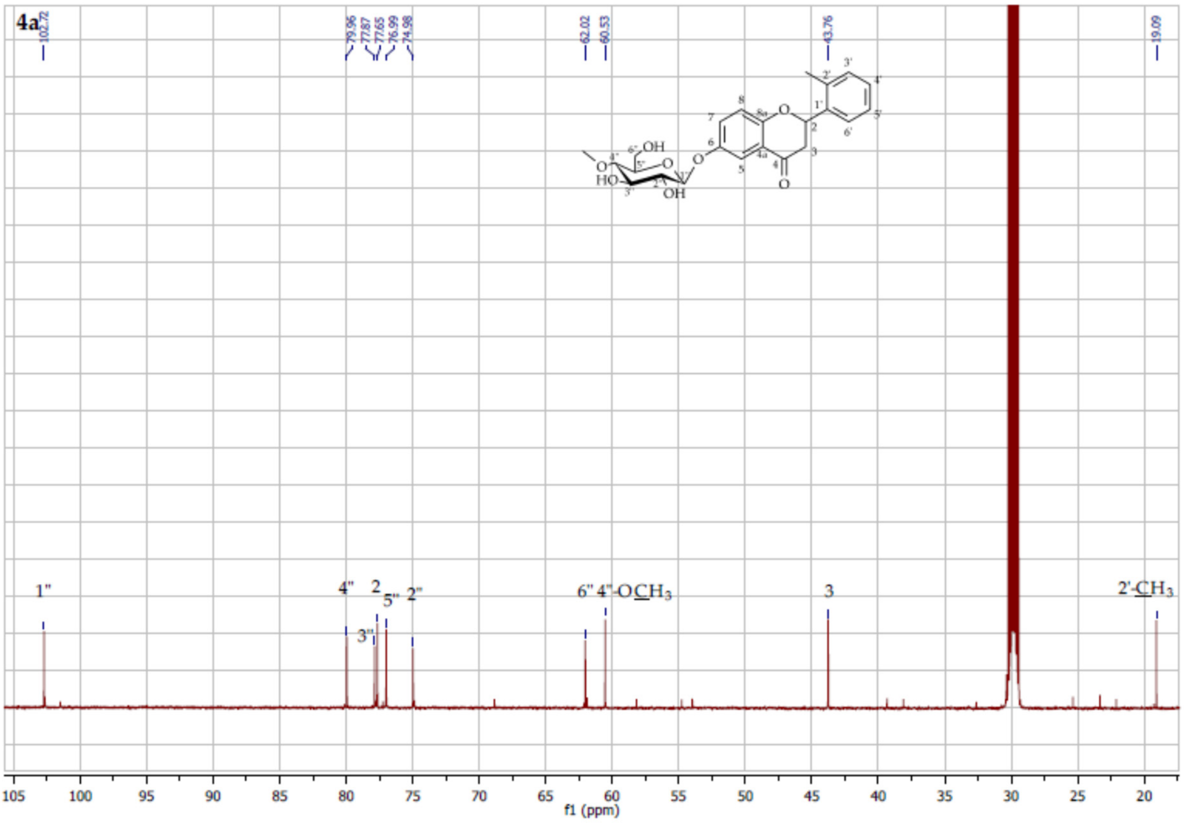

Figure S23. ^{13}C NMR spectrum expansion (*δ*, acetone-d6, 151 MHz) of 2′-methylflavanone 6-*O*-*β*-D-(4′′-*O*-methyl)-glucopyranoside (**4a**)

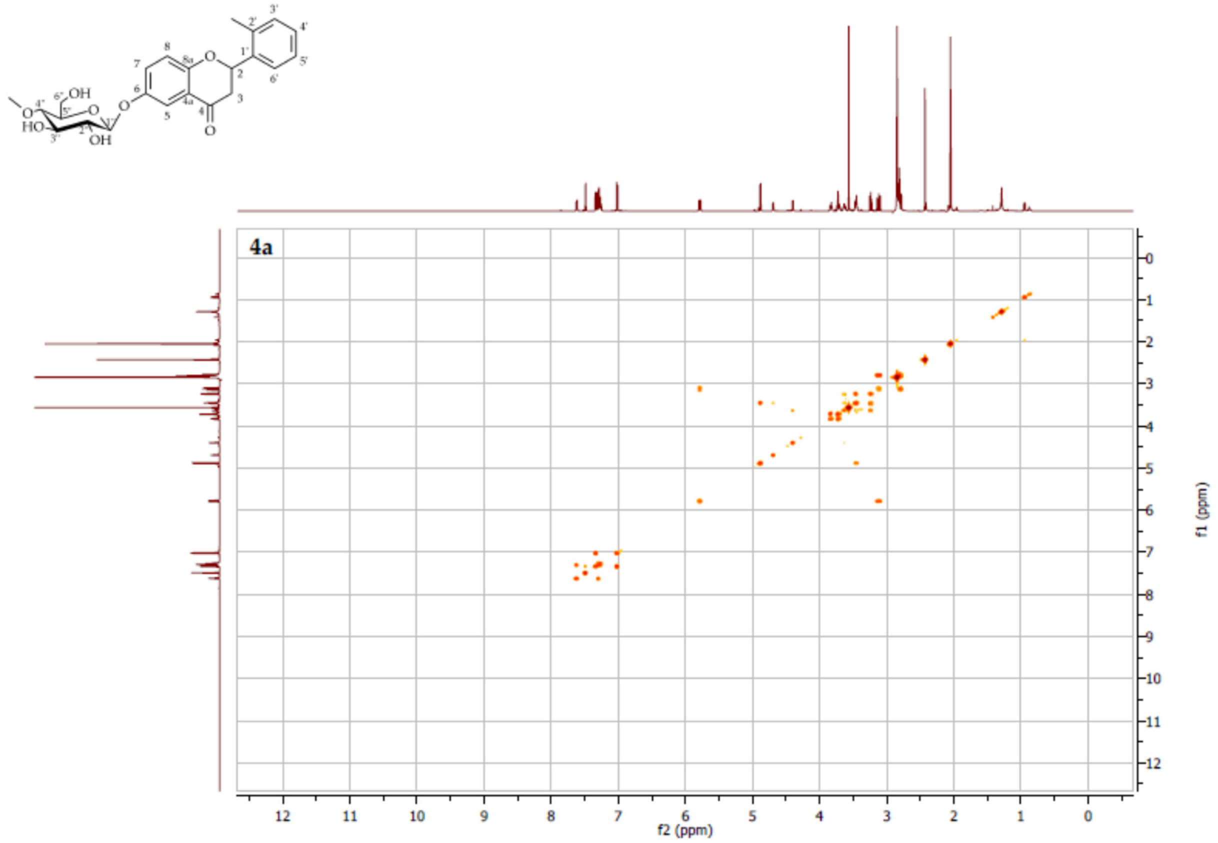

Figure S24. COSY contour map – ^1H x ^1H of 2′-methylflavanone 6-*O*-*β*-D-(4′′-*O*-methyl)-glucopyranoside (**4a**)

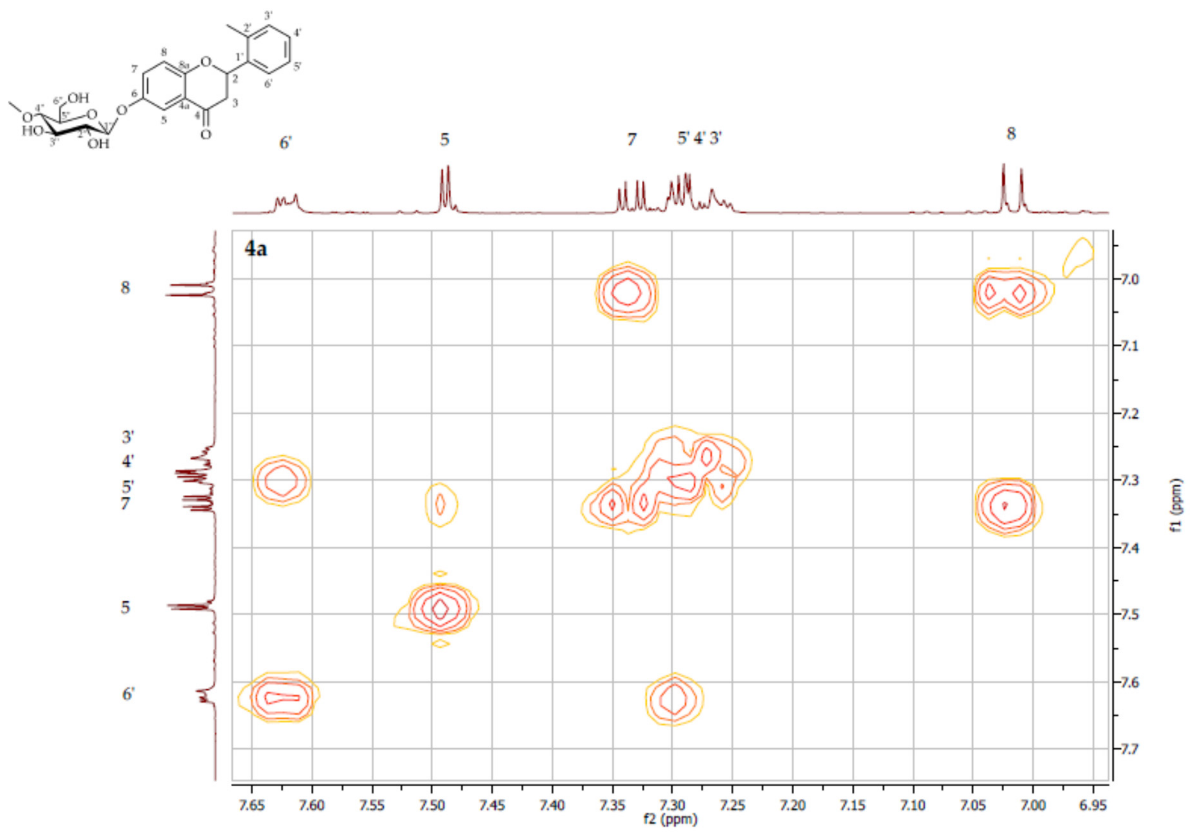

Figure S25. COSY contour map – $^{1}H \times {}^{1}H$ expansion of 2′-methylflavanone 6-*O*-*β*-D-(4′′-*O*-methyl)-glucopyranoside (**4a**)

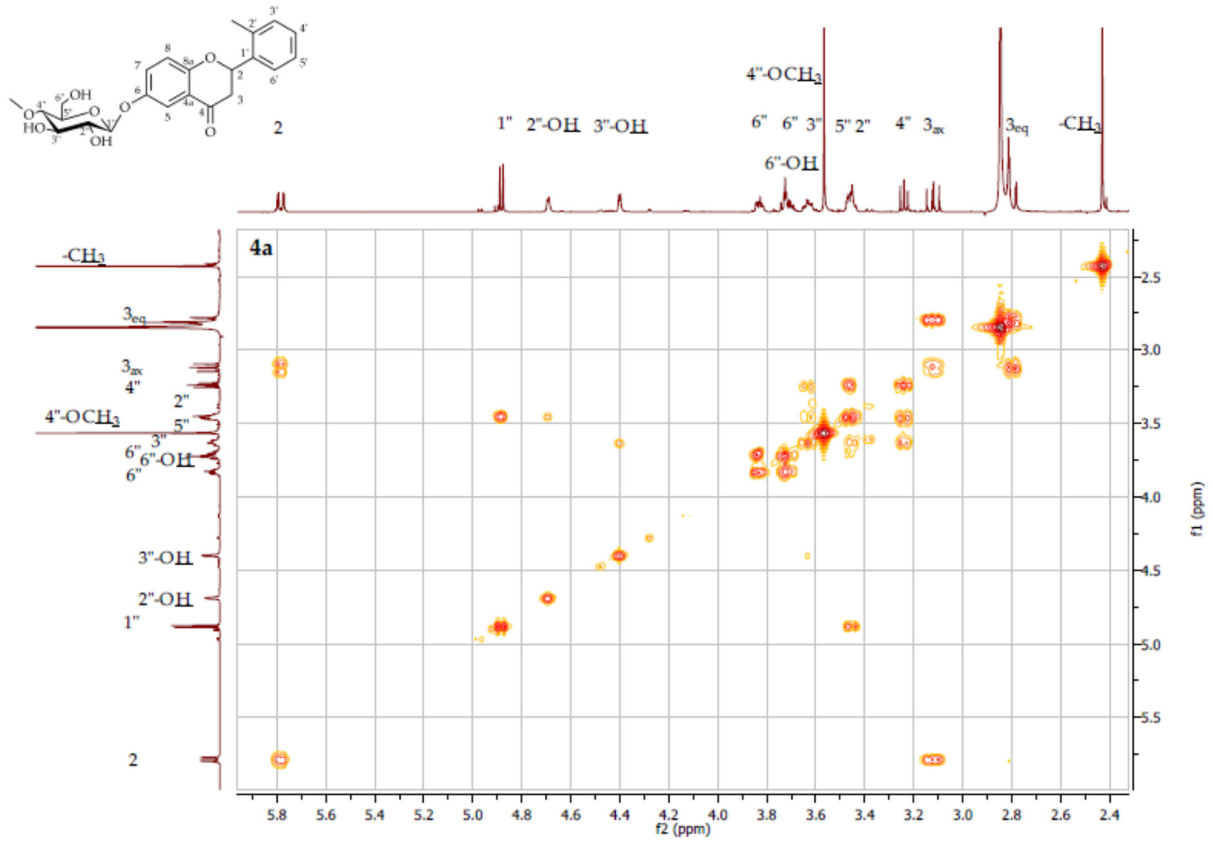

Figure S26. COSY contour map – $^{1}H \times {}^{1}H$ expansion of 2′-methylflavanone 6-*O*-*β*-D-(4′′-*O*-methyl)-glucopyranoside (**4a**)

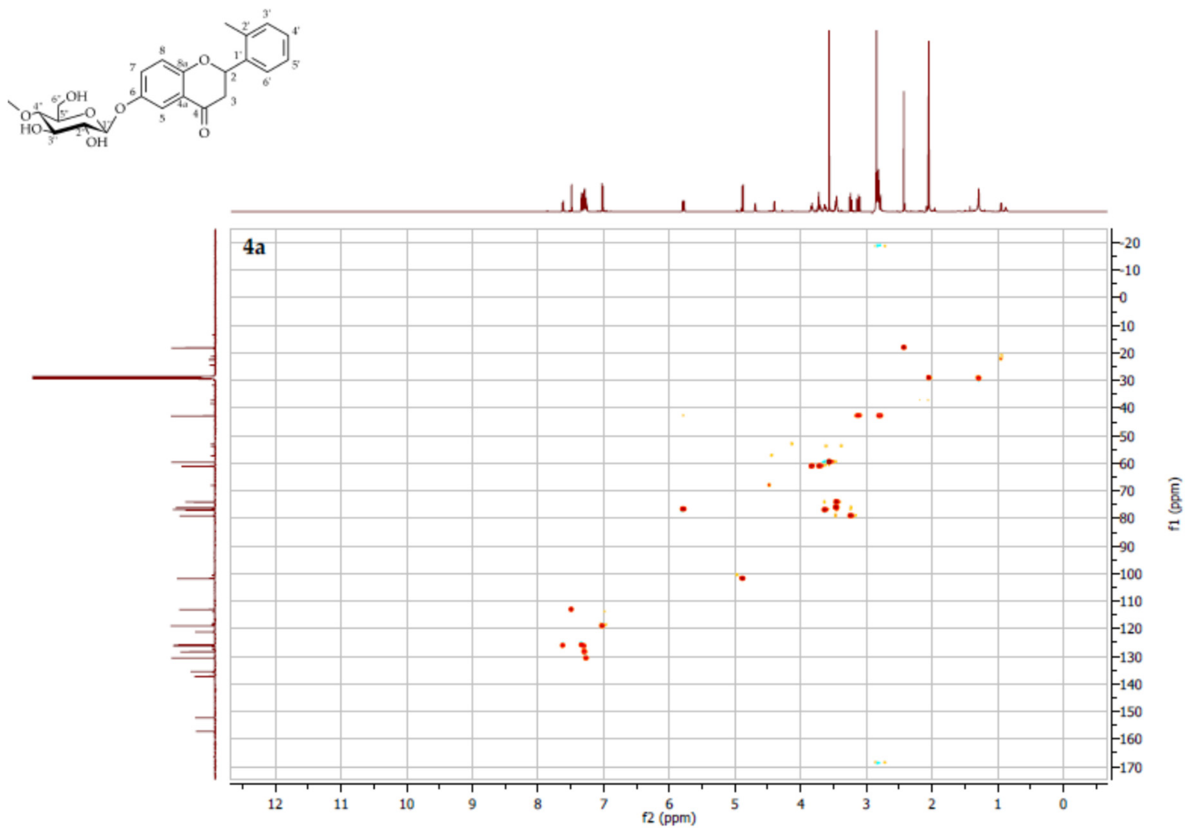

Figure S27. HSQC contour map – ^{1}H x ^{13}C of 2′-methylflavanone 6-*O*-*β*-D-(4′′-*O*-methyl)-glucopyranoside (**4a**)

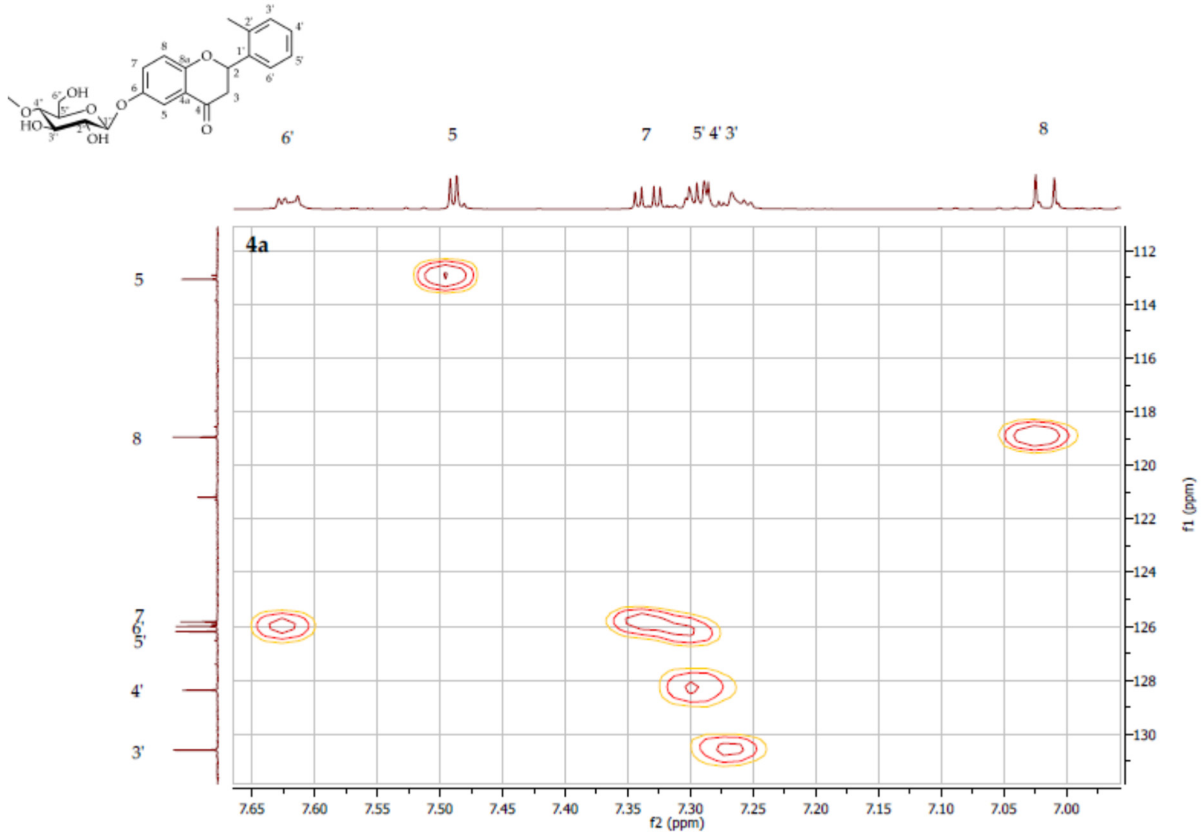

Figure S28. HSQC contour map – ^{1}H x ^{13}C expansion of 2′-methylflavanone 6-*O*-*β*-D-(4′′-*O*-methyl)-glucopyranoside (**4a**)

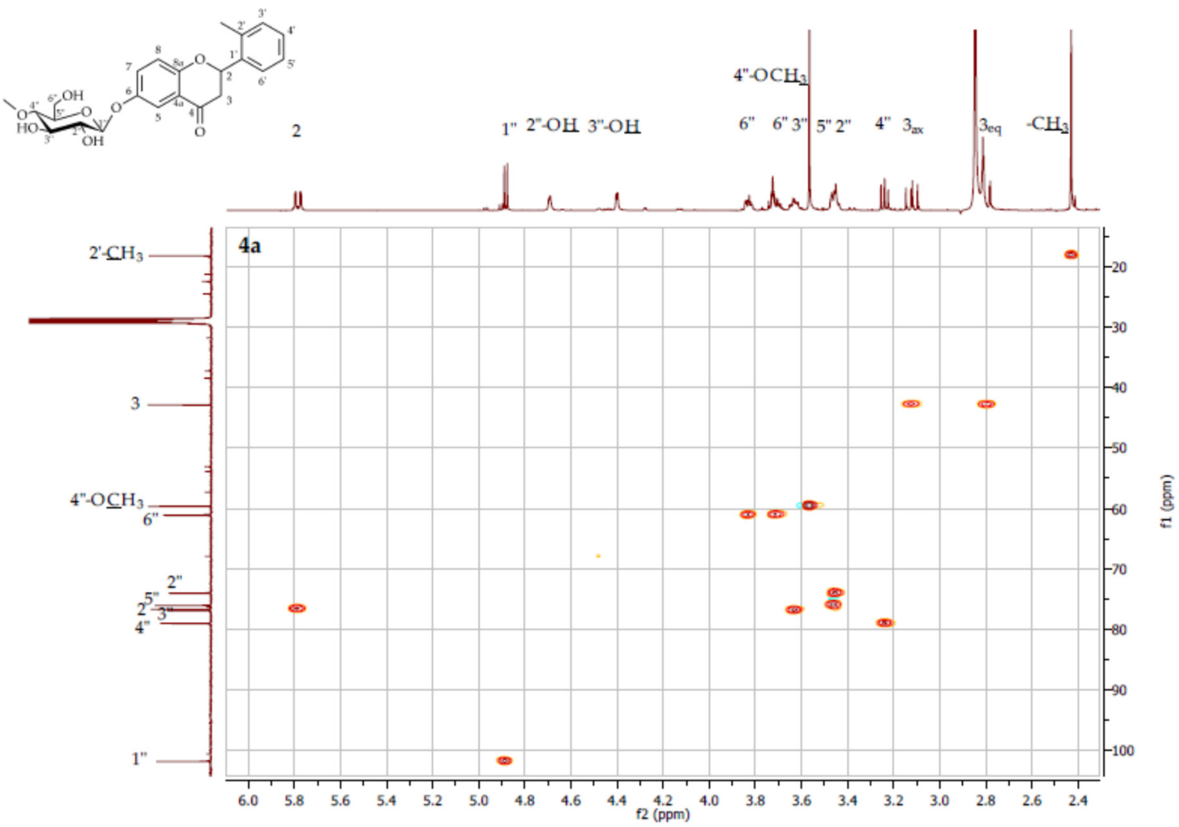

Figure S29. HSQC contour map – ^{1}H x ^{13}C expansion of 2′-methylflavanone 6-*O*-*β*-D-(4′′-*O*-methyl)-glucopyranoside (**4a**)

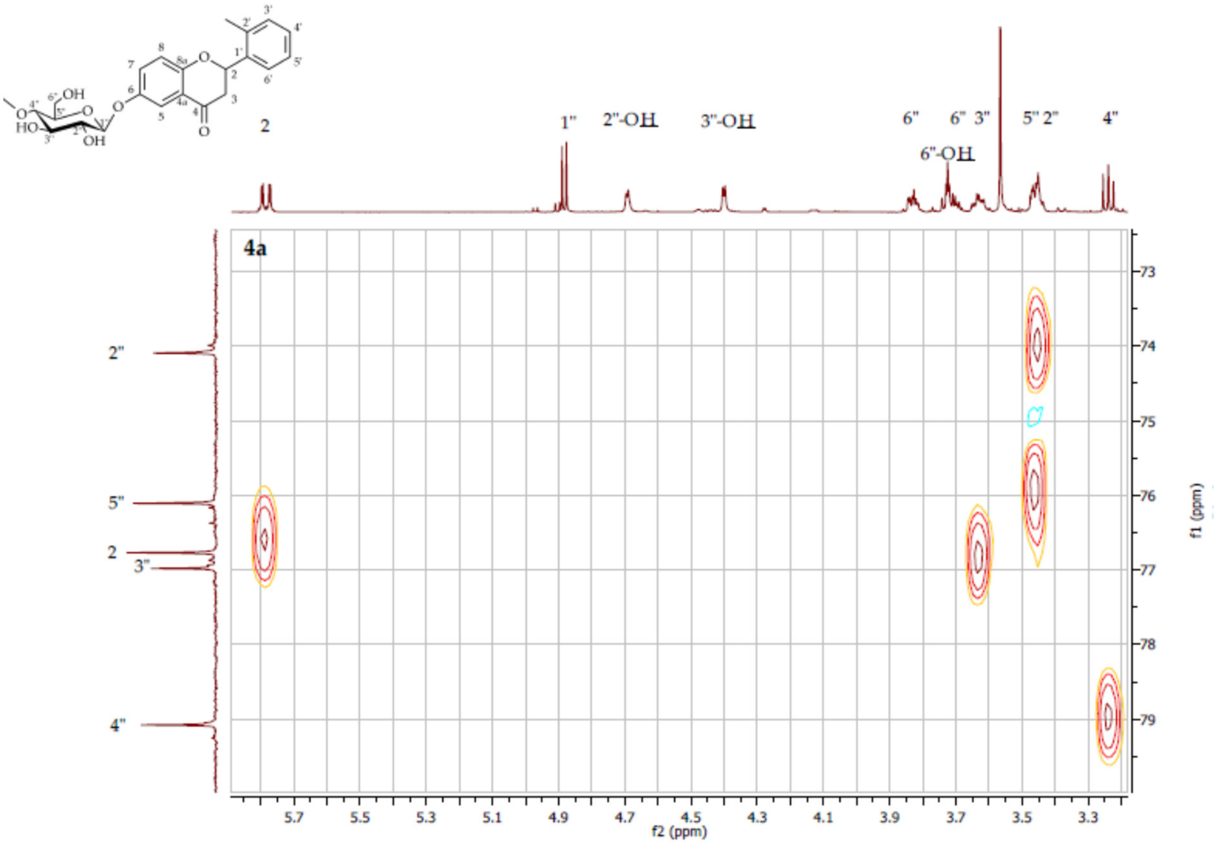

Figure S30. HSQC contour map – ^{1}H x ^{13}C expansion of 2′-methylflavanone 6-*O*-*β*-D-(4′′-*O*-methyl)-glucopyranoside (**4a**)

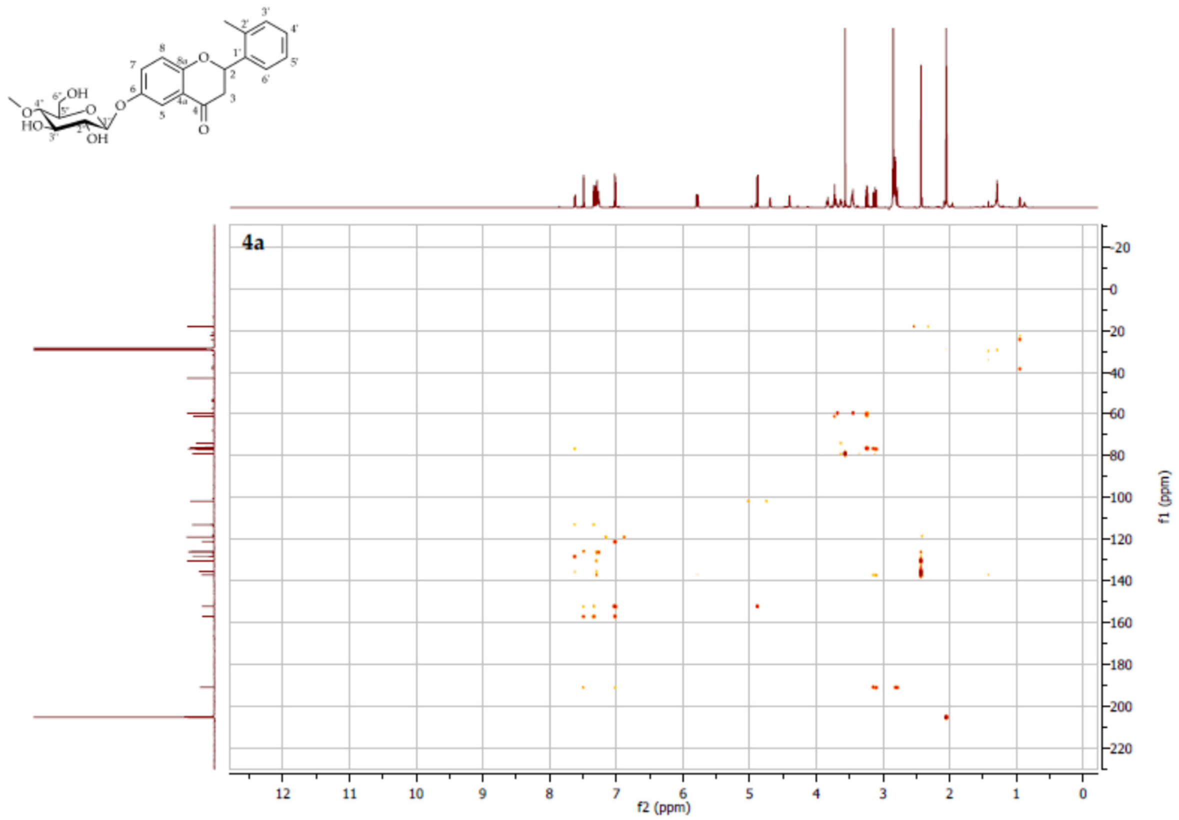

Figure S31. HMBC contour map – ^{1}H x ^{13}C of 2′-methylflavanone 6-*O*-*β*-D-(4′′-*O*-methyl)-glucopyranoside (**4a**)

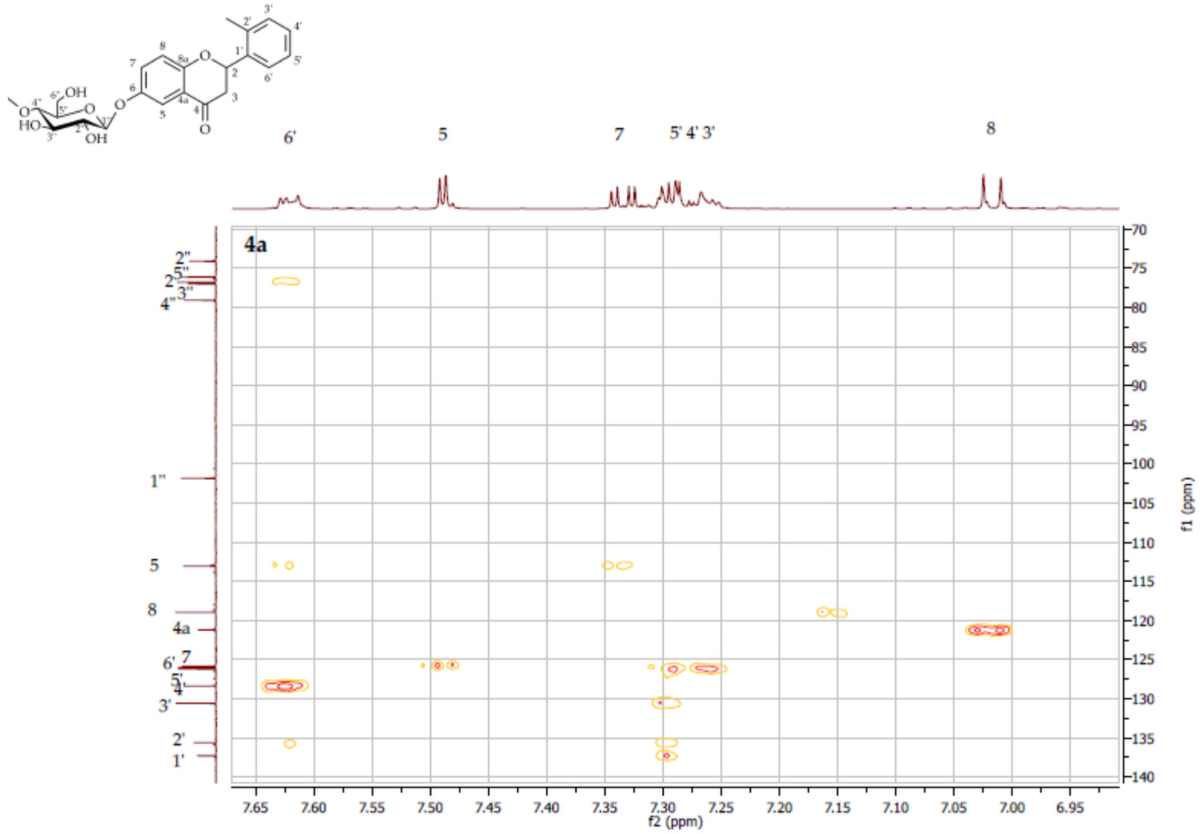

Figure S32. HMBC contour map – ^{1}H x ^{13}C expansion of 2′-methylflavanone 6-*O*-*β*-D-(4′′-*O*-methyl)-glucopyranoside (**4a**)

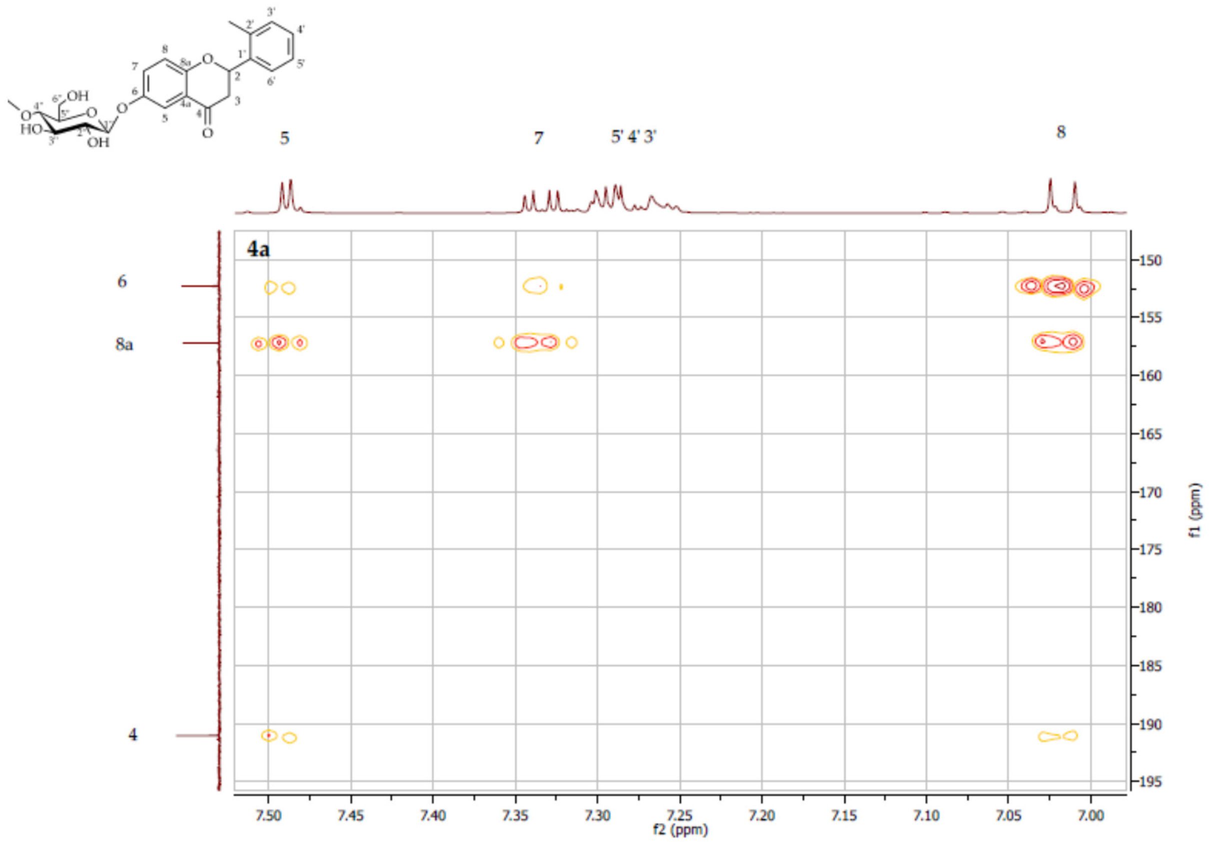

Figure S33. HMBC contour map – ^{1}H x ^{13}C expansion of 2′-methylflavanone 6-*O*-*β*-D-(4′′-*O*-methyl)-glucopyranoside (**4a**)

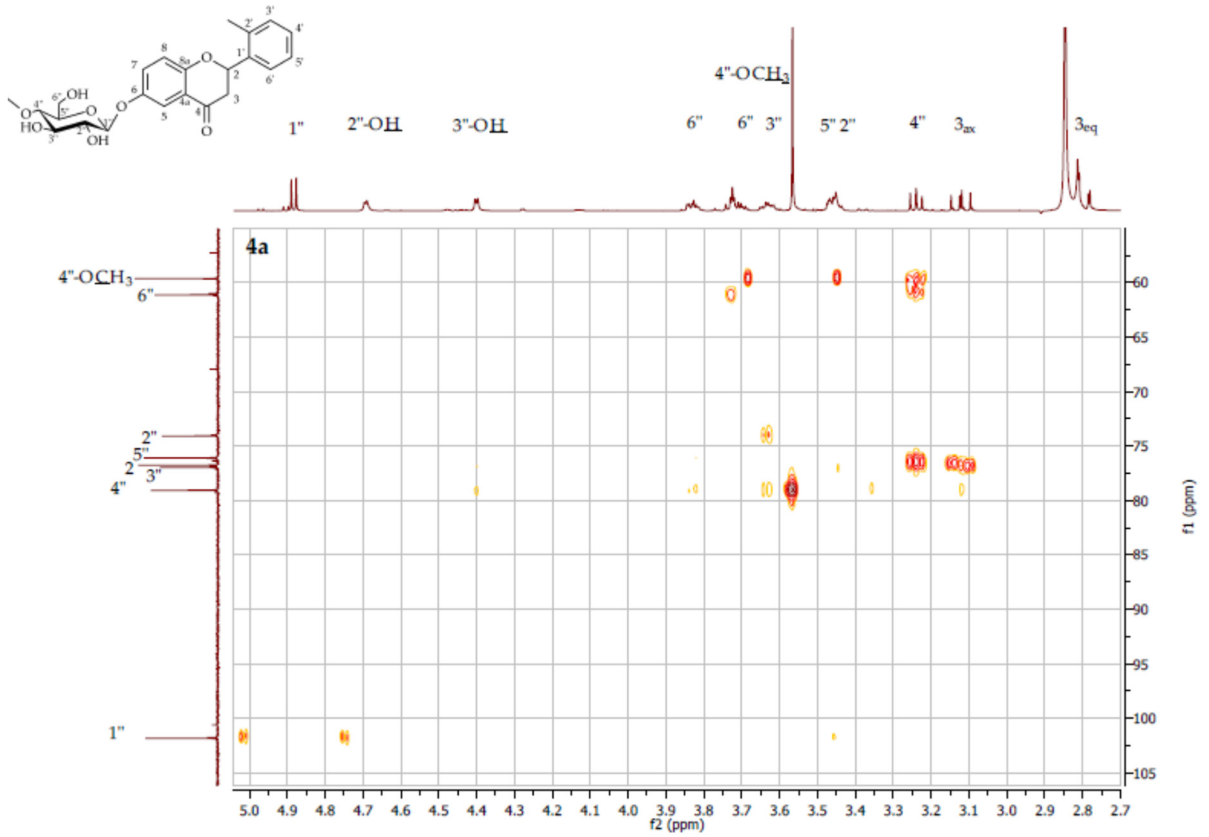

Figure S34. HMBC contour map – ^{1}H x ^{13}C expansion of 2′-methylflavanone 6-*O*-*β*-D-(4′′-*O*-methyl)-glucopyranoside (**4a**)

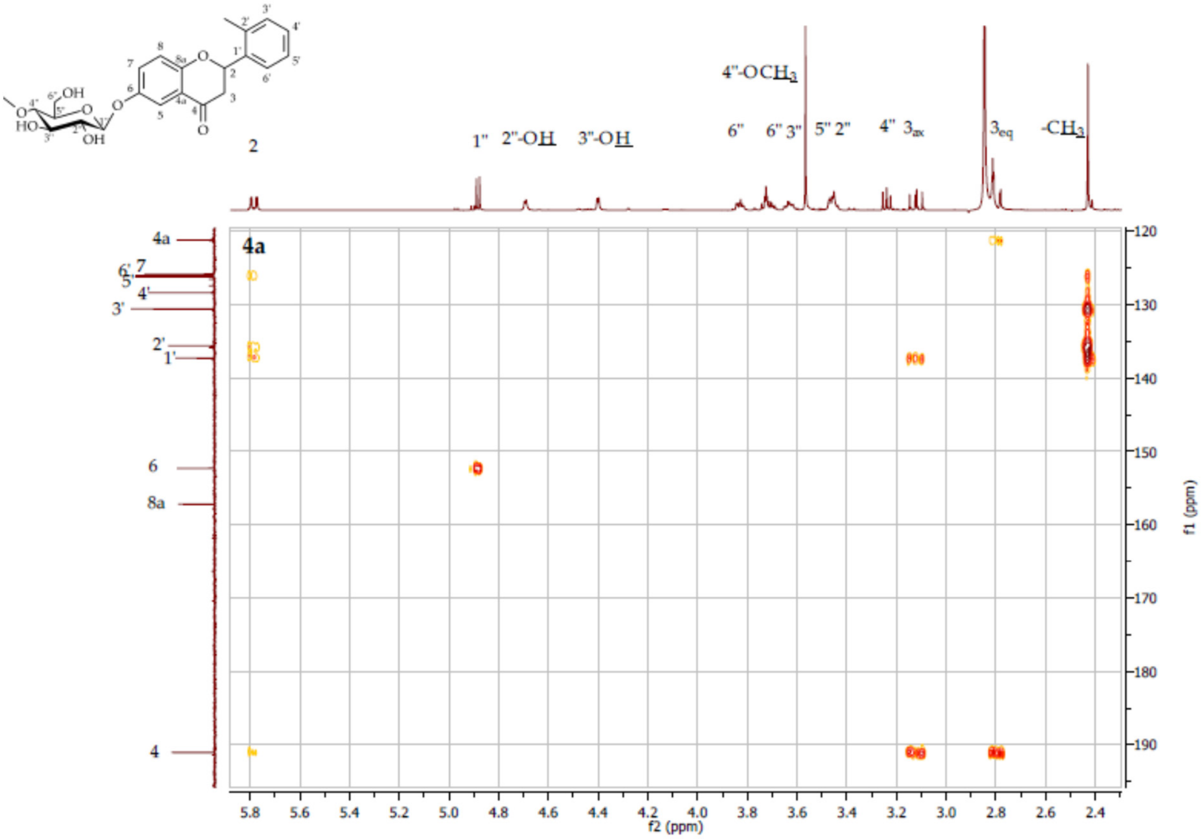

Figure S35. HMBC contour map – ^{1}H x ^{13}C expansion of 2′-methylflavanone 6-*O*-*β*-D-(4′′-*O*-methyl)-glucopyranoside (**4a**)

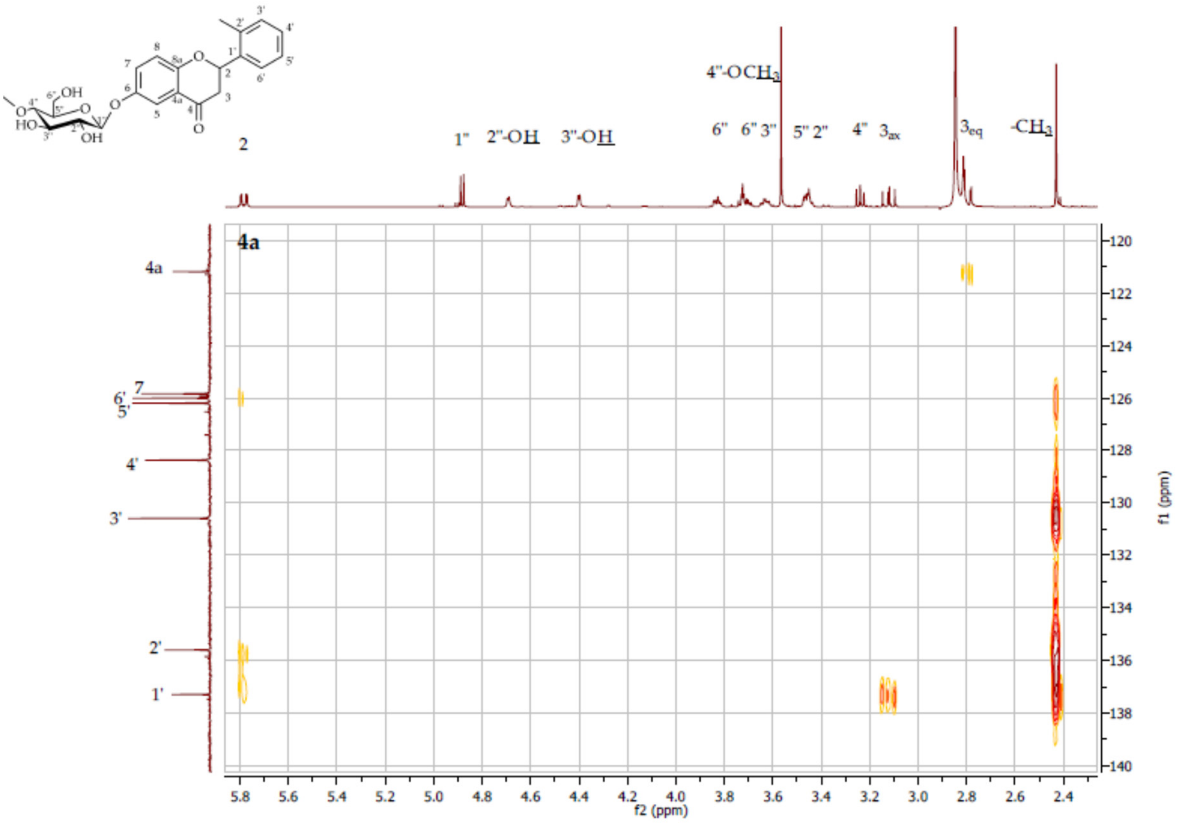

Figure S36. HMBC contour map – ^{1}H x ^{13}C expansion of 2′-methylflavanone 6-*O*-*β*-D-(4′′-*O*-methyl)-glucopyranoside (**4a**)

Molecular formula: $C_{23}H_{26}O_9$
Formula weight: 446.16
Ionization mode: negative
Precursor:[M – H]⁻ 445.30
445.3000>269.0500 CE: 18.0
445.3000>251.1000 CE: 29.0

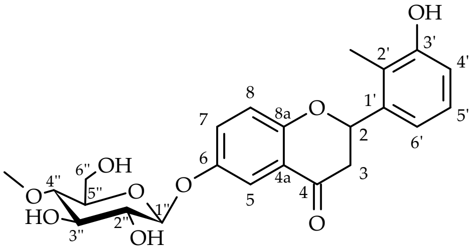

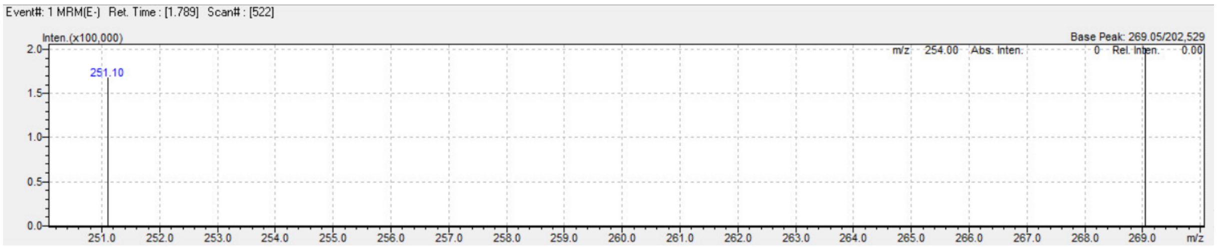

Figure S37. MS analysis of 3′-hydroxy-2′-methylflavanone 6-*O*-*β*-D-(4′′-*O*-methyl)-glucopyranoside (**4b**)

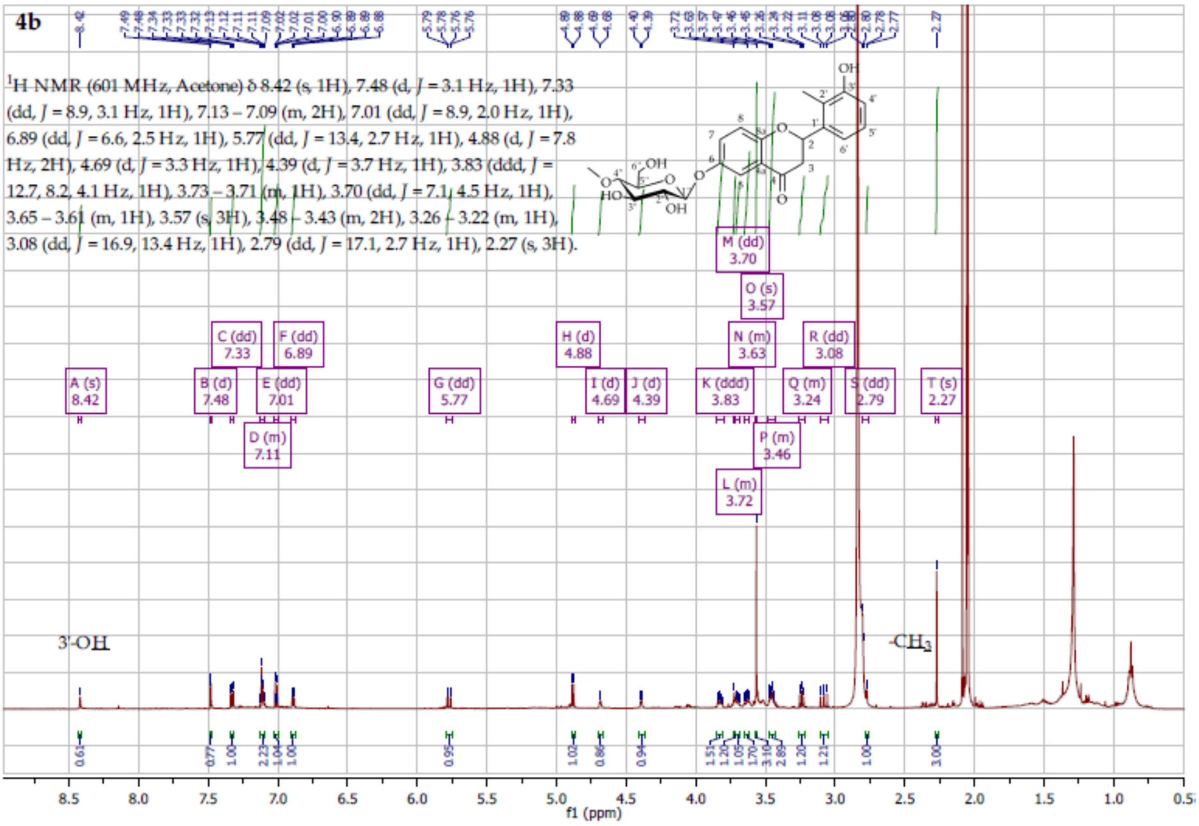

Figure S38. ¹H NMR spectrum (*δ*, acetone-d6, 600 MHz) of 3′-hydroxy-2′-methylflavanone 6-*O*-*β*-D-(4′′-*O*-methyl)-glucopyranoside (**4b**)

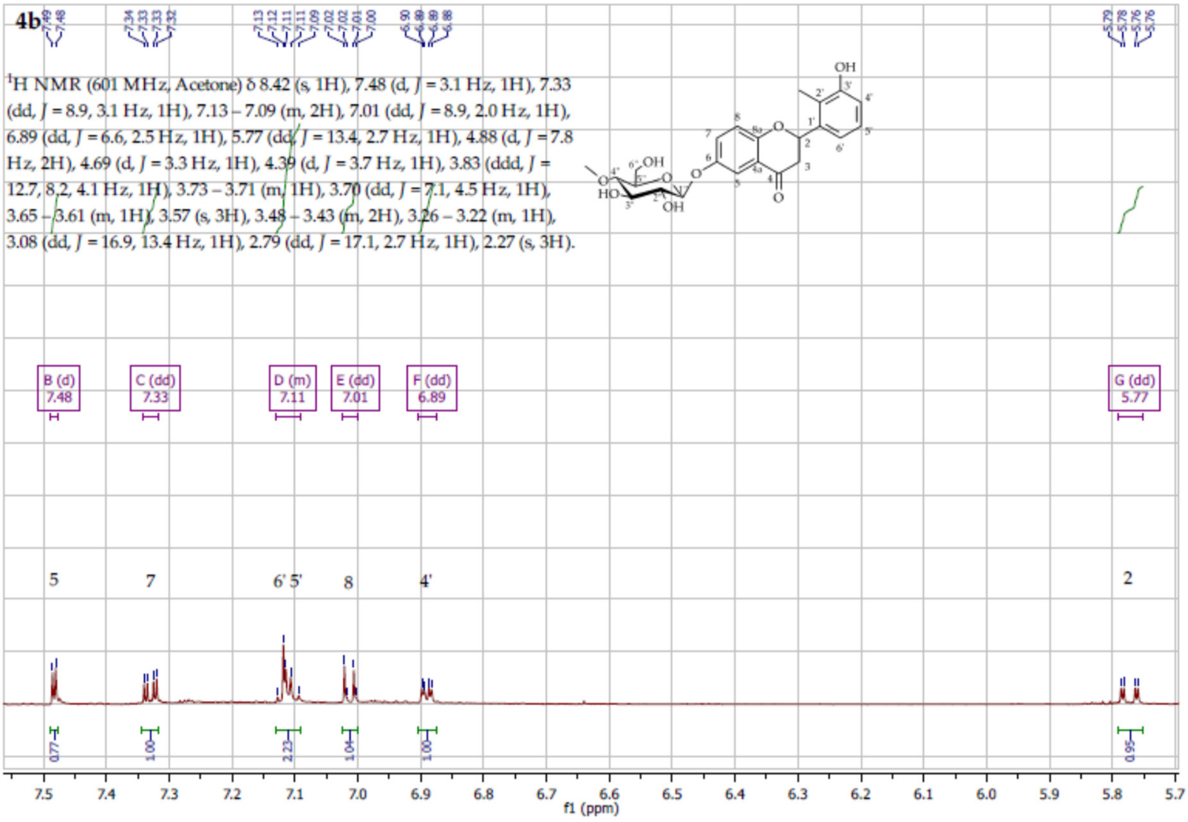

Figure S39. ^{1}H NMR spectrum expansion (*δ*, acetone-d6, 600 MHz) of 3′-hydroxy-2′-methylflavanone 6-*O*-*β*-D-(4′′-*O*-methyl)-glucopyranoside (**4b**)

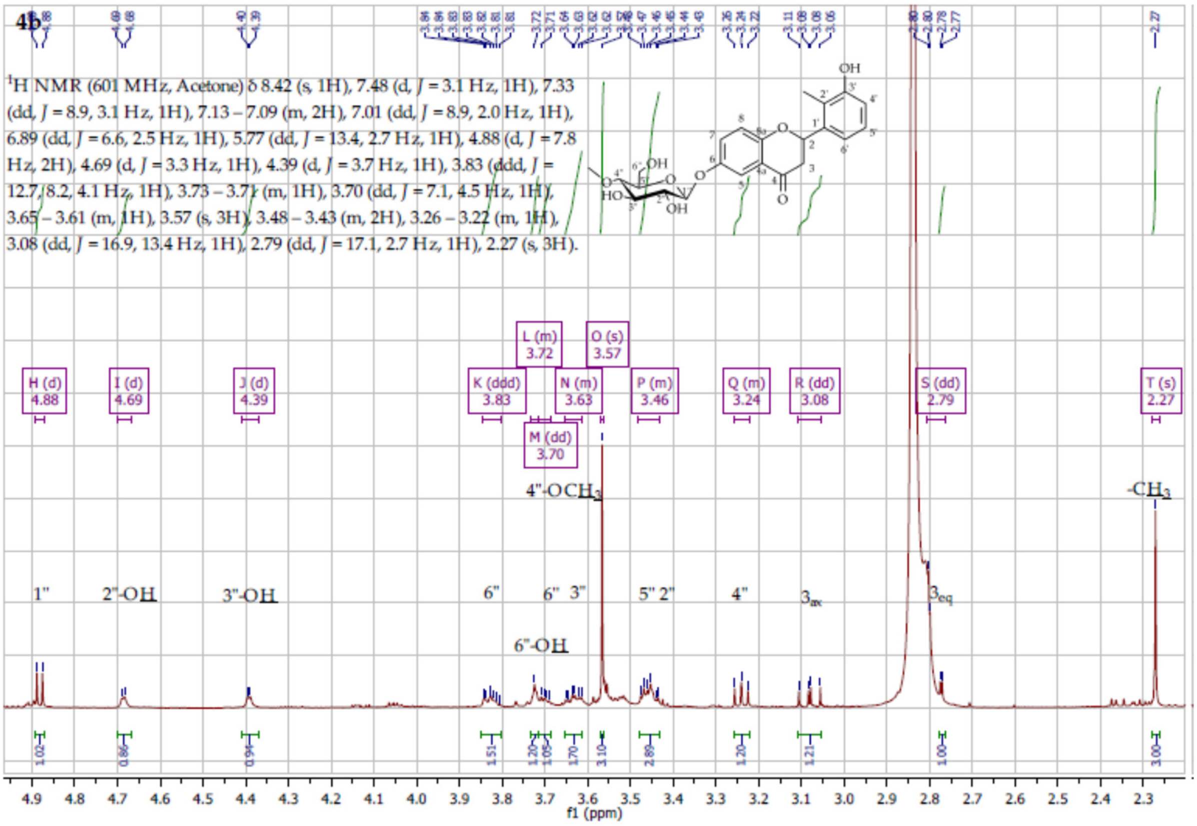

Figure S40. ^{1}H NMR spectrum expansion (*δ*, acetone-d6, 600 MHz) of 3′-hydroxy-2′-methylflavanone 6-*O*-*β*-D-(4′′-*O*-methyl)-glucopyranoside (**4b**)

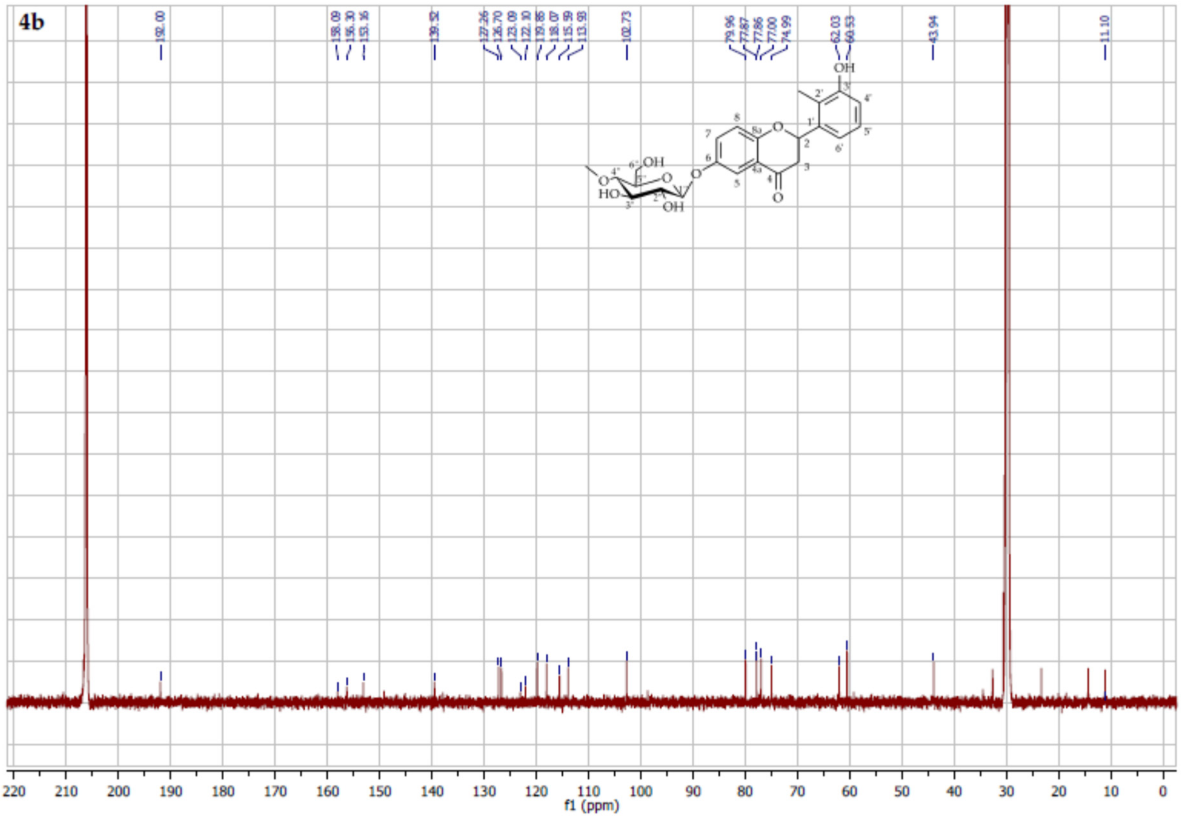

Figure S41. ^{13}C NMR spectrum (*δ*, acetone-d6, 151 MHz) of 3′-hydroxy-2′-methylflavanone 6-*O*-*β*-D-(4′′-*O*-methyl)-glucopyranoside (**4b**)

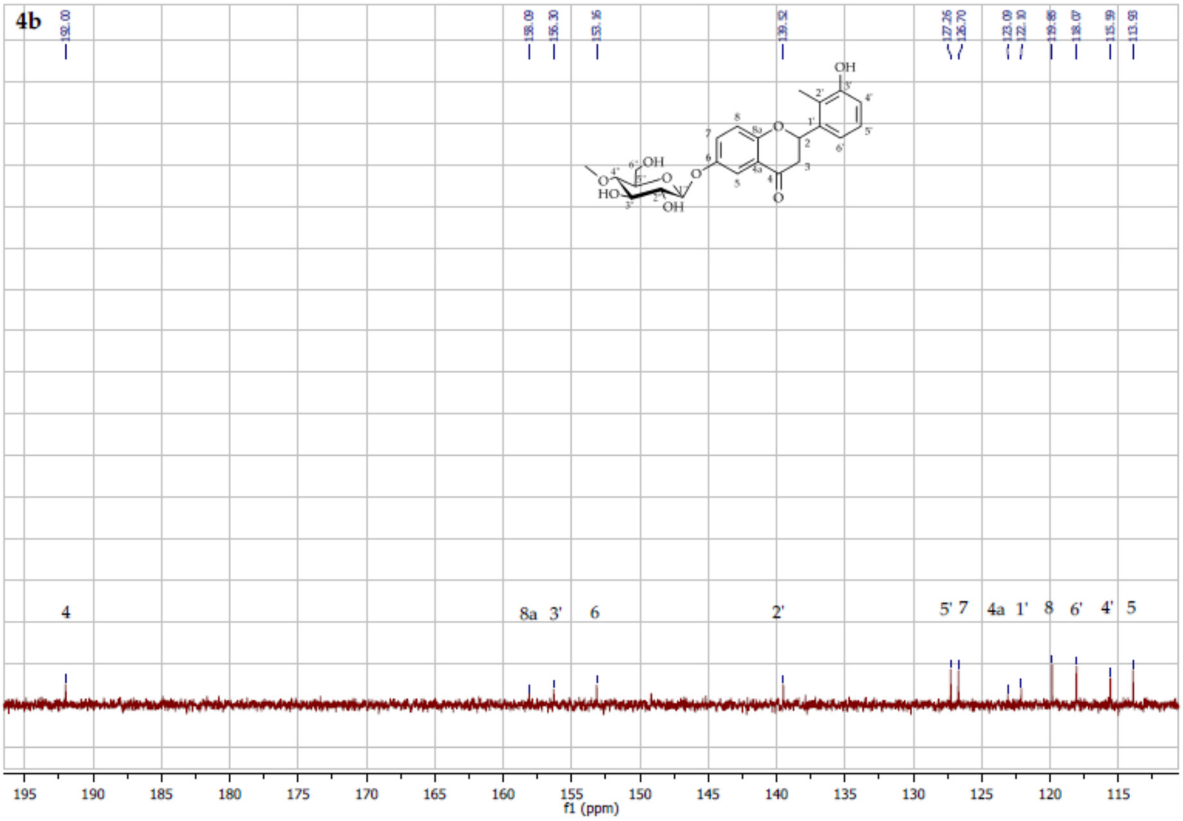

Figure S42. ^{13}C NMR spectrum expansion (*δ*, acetone-d6, 151 MHz) of 3′-hydroxy-2′-methylflavanone 6-*O*-*β*-D-(4′′-*O*-methyl)-glucopyranoside (**4b**)

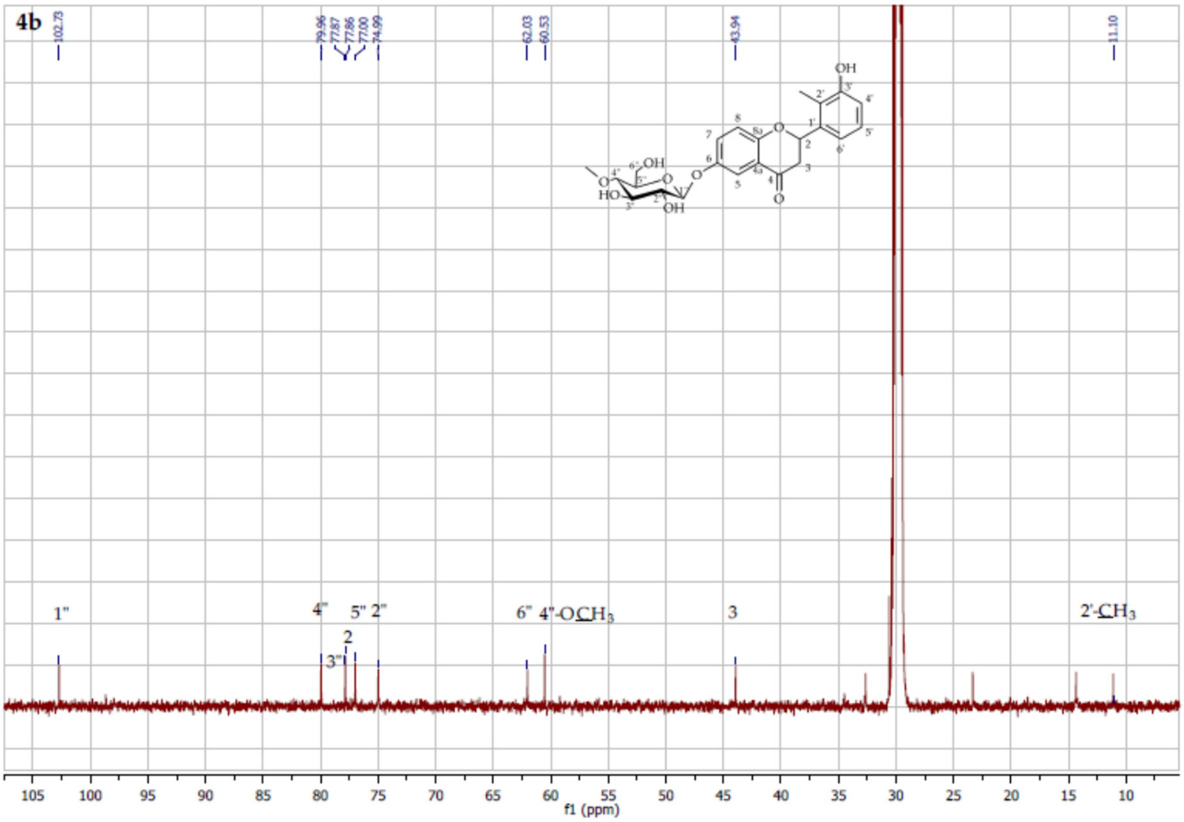

Figure S43. ^{13}C NMR spectrum expansion (*δ*, acetone-d6, 151 MHz) of 3′-hydroxy-2′-methylflavanone 6-*O*-*β*-D-(4′′-*O*-methyl)-glucopyranoside (**4b**)

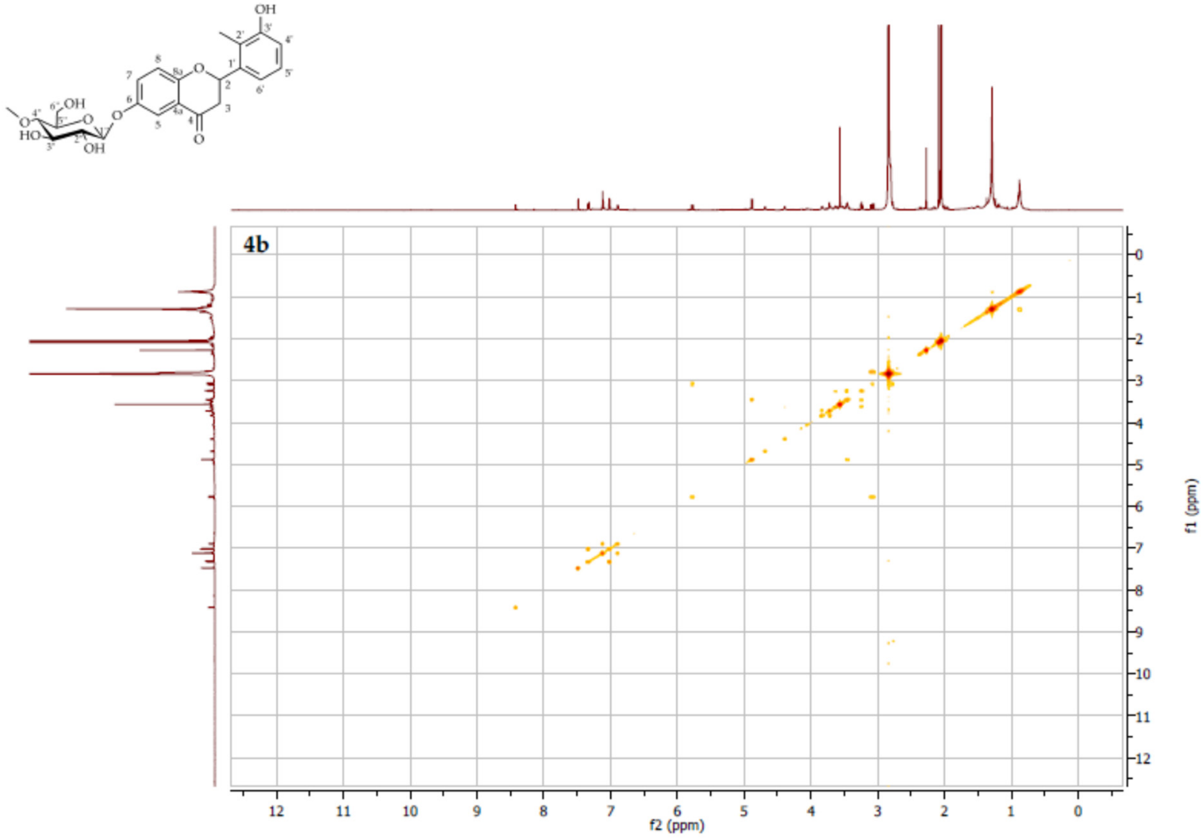

Figure S44. COSY contour map – ^{1}H x ^{1}H of 3′-hydroxy-2′-methylflavanone 6-*O*-*β*-D-(4′′-*O*-methyl)-glucopyranoside (**4b**)

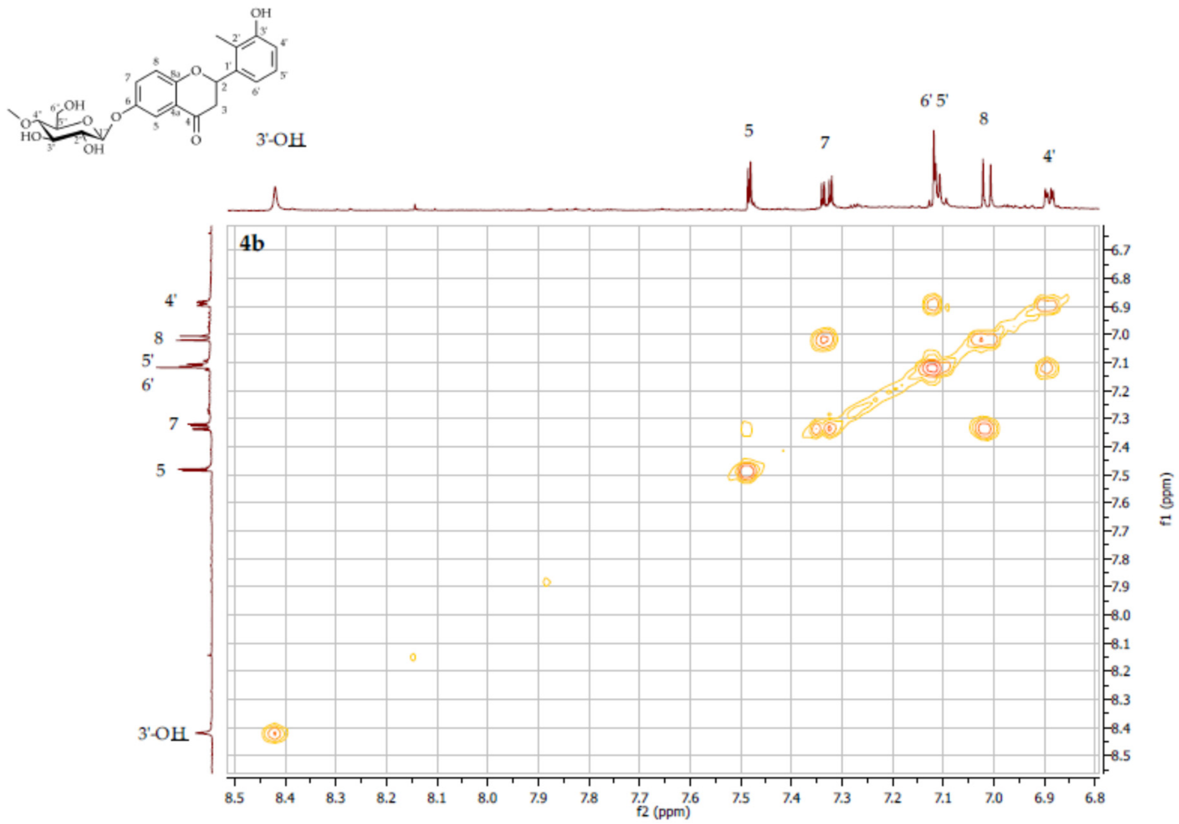

Figure S45. COSY contour map – ^{1}H x ^{1}H expansion of 3′-hydroxy-2′-methylflavanone 6-*O*-*β*-D-(4′′-*O*-methyl)-glucopyranoside (**4b**)

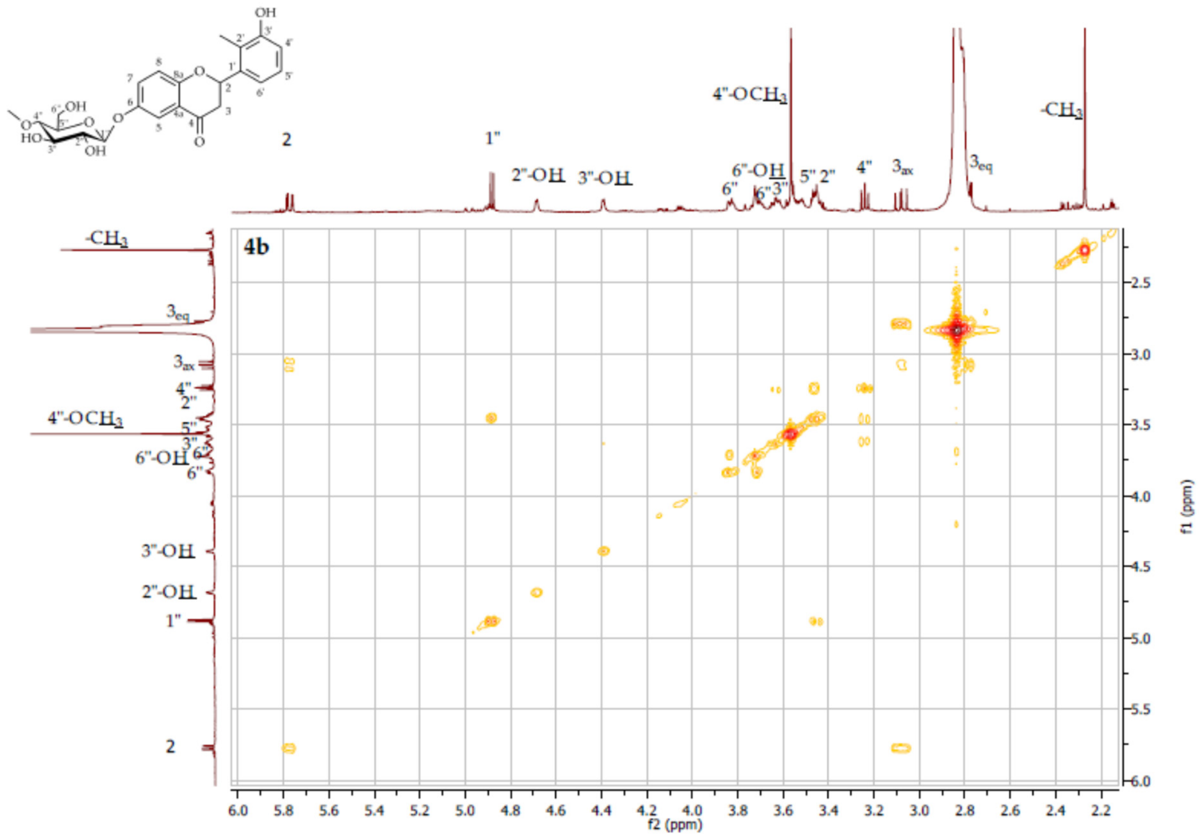

Figure S46. COSY contour map – ^{1}H x ^{1}H expansion of 3′-hydroxy-2′-methylflavanone 6-*O*-*β*-D-(4′′-*O*-methyl)-glucopyranoside (**4b**)

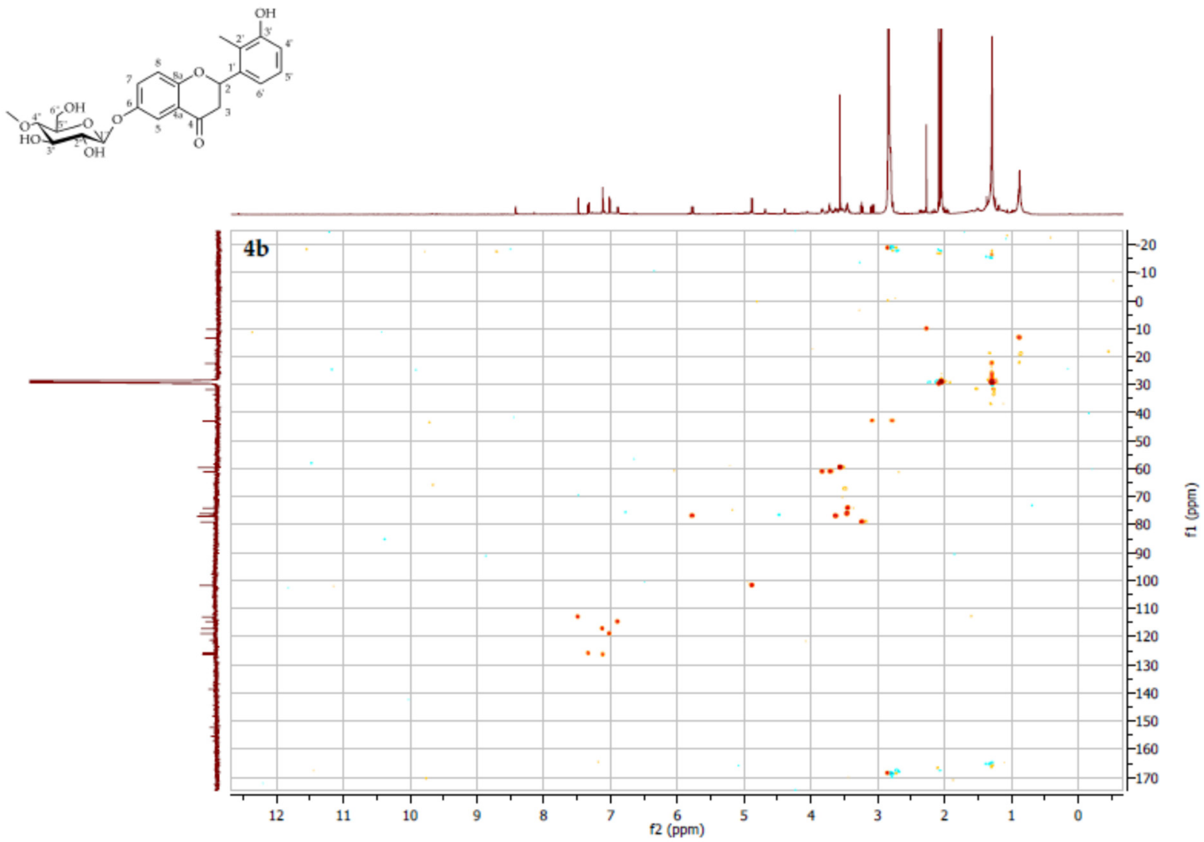

Figure S47. HSQC contour map – ^{1}H x ^{13}C of 3′-hydroxy-2′-methylflavanone 6-*O*-*β*-D-(4′′-*O*-methyl)-glucopyranoside (**4b**)

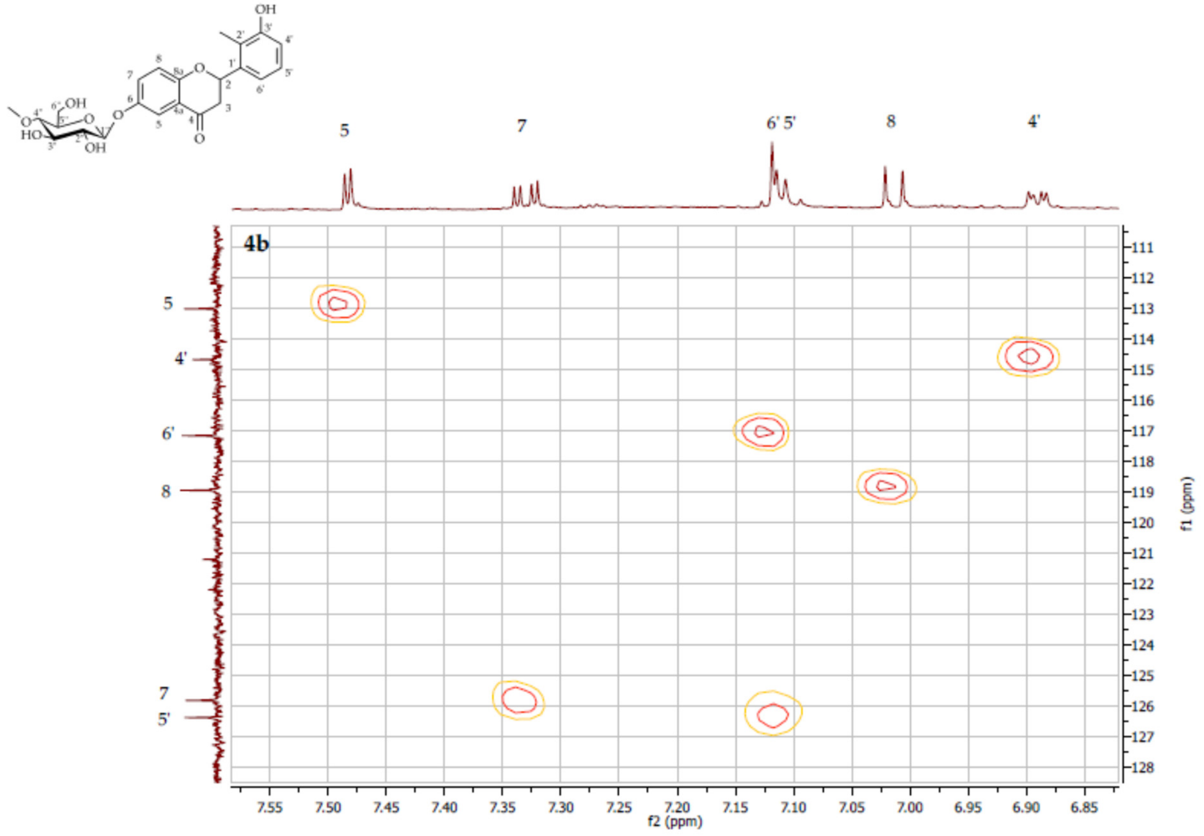

Figure S48. HSQC contour map – ^{1}H x ^{13}C expansion of 3′-hydroxy-2′-methylflavanone 6-*O*-*β*-D-(4′′-*O*-methyl)-glucopyranoside (**4b**)

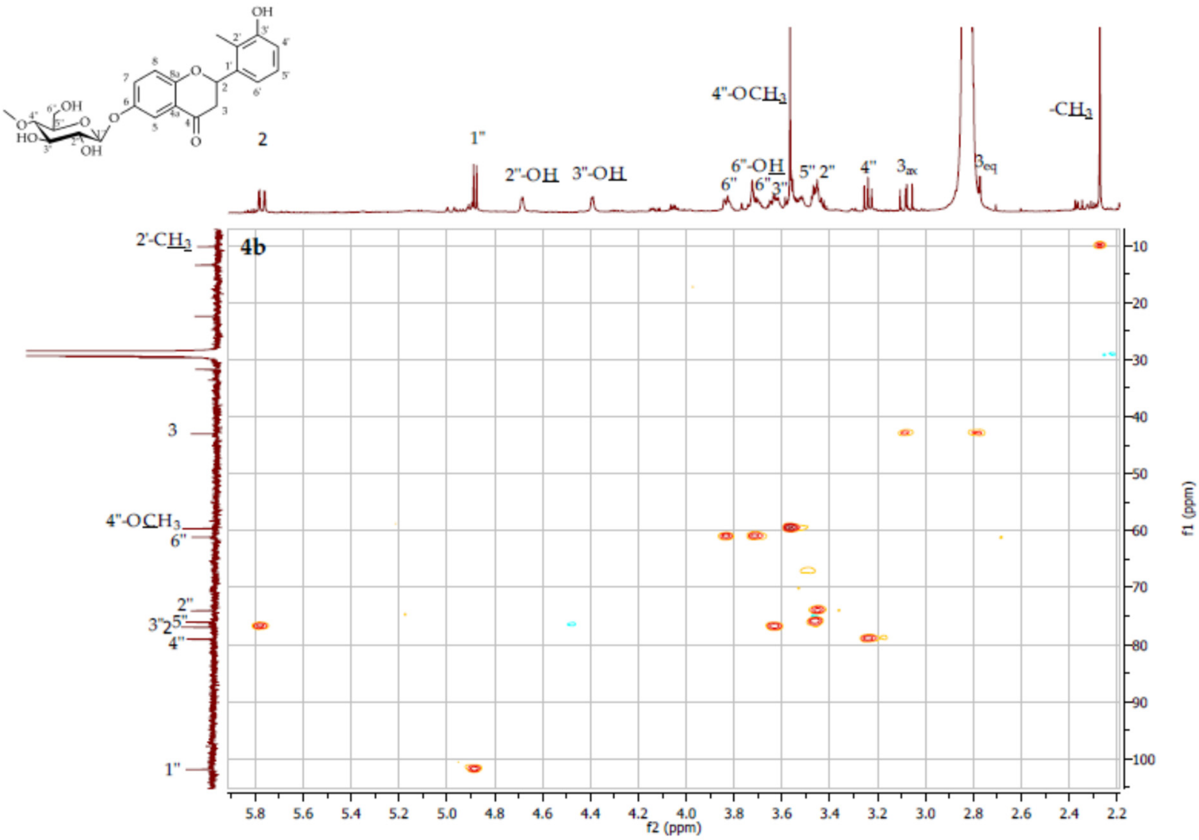

Figure S49. HSQC contour map – ^{1}H x ^{13}C expansion of 3′-hydroxy-2′-methylflavanone 6-*O*-*β*-D-(4′′-*O*-methyl)-glucopyranoside (**4b**)

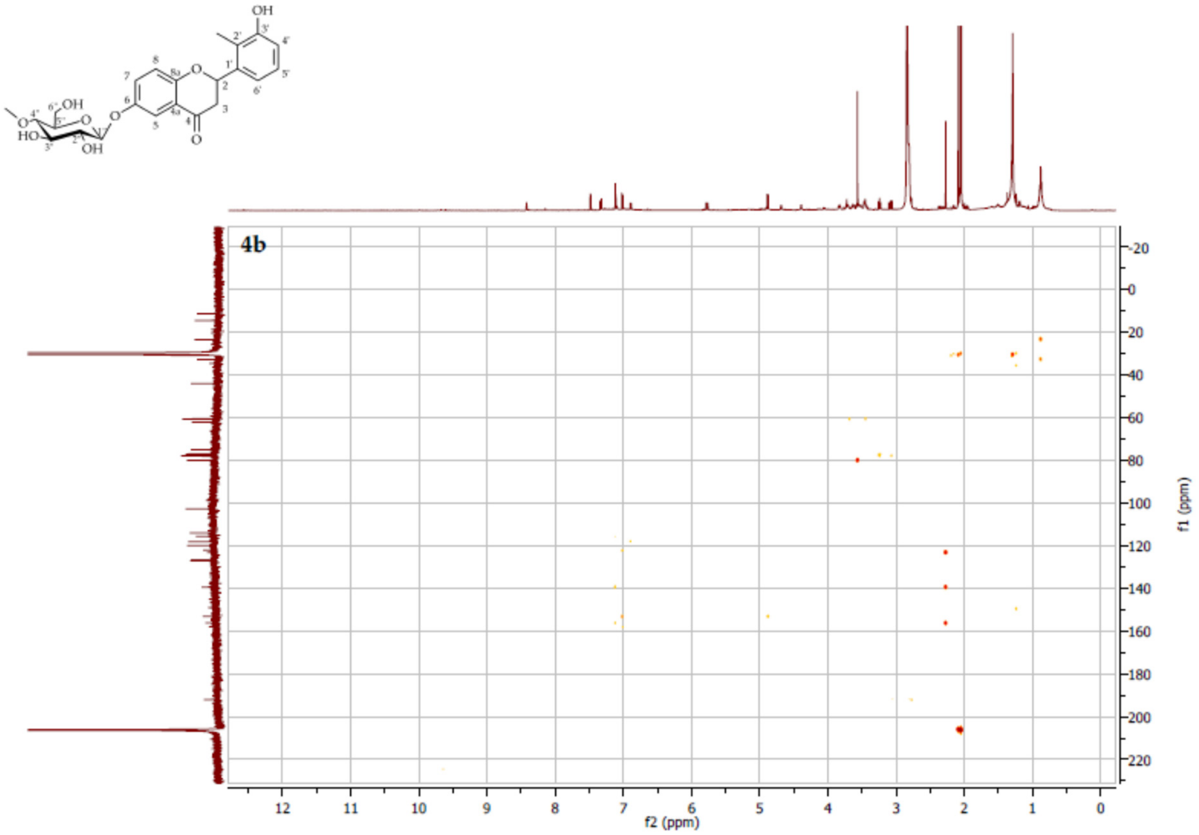

Figure S50. HMBC contour map – ^{1}H x ^{13}C 3′-hydroxy-2′-methylflavanone 6-*O*-*β*-D-(4′′-*O*-methyl)-glucopyranoside (**4b**)

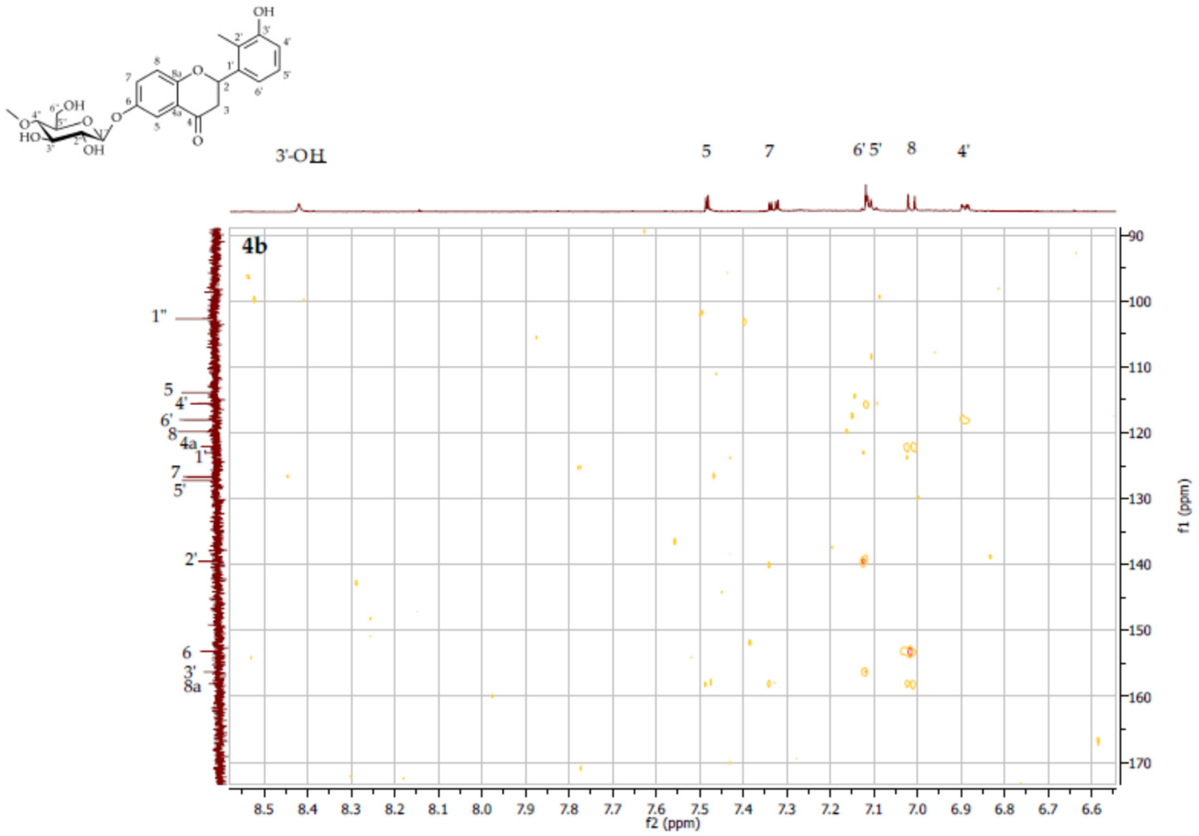

Figure S51. HMBC contour map – ^{1}H x ^{13}C expansion of 3′-hydroxy-2′-methylflavanone 6-*O*-*β*-D-(4′′-*O*-methyl)-glucopyranoside (**4b**)

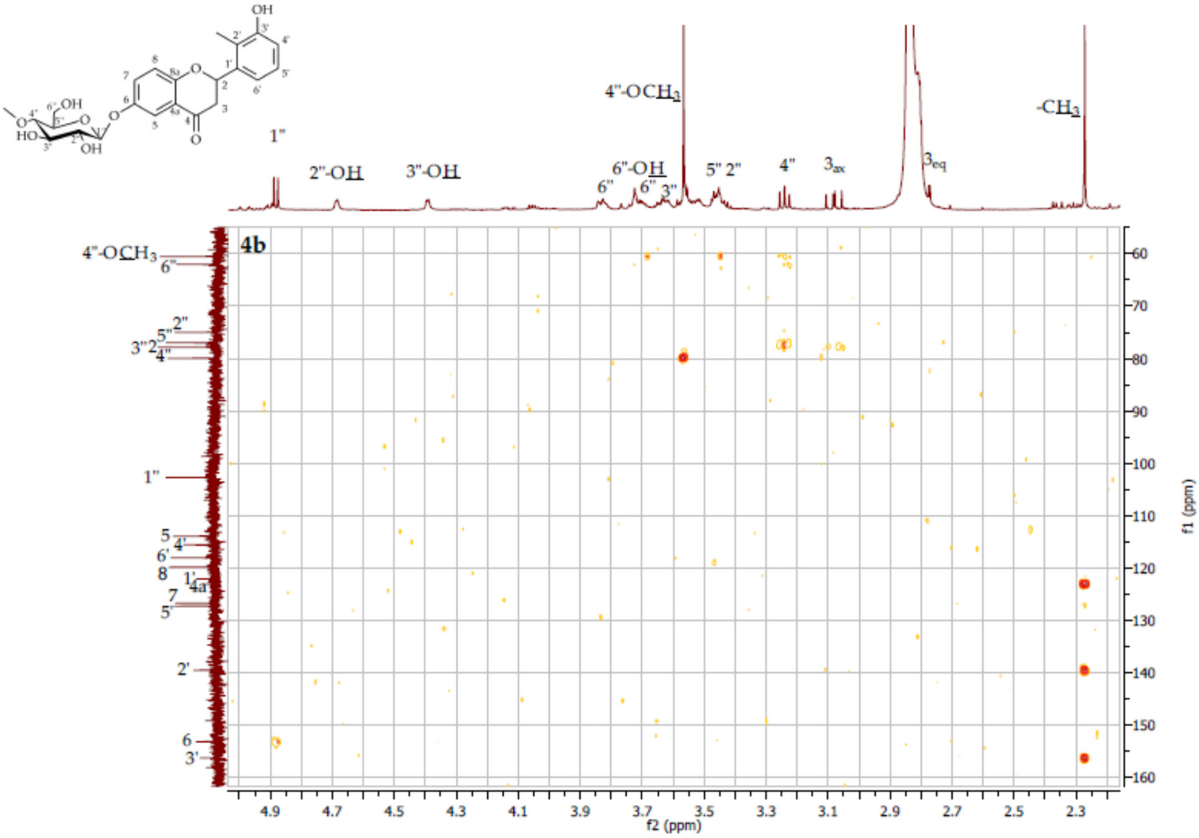

Figure S52. HMBC contour map – ^{1}H x ^{13}C expansion of 3′-hydroxy-2′-methylflavanone 6-*O*-*β*-D-(4′′-*O*-methyl)-glucopyranoside (**4b**)

Molecular formula: $C_{23}H_{28}O_7$

Formula weight: 416.18

Ionization mode: negative

Precursor: adduct $[M + 46 - H]^-$ ($[M + HCOOH - H]^-$) 461.20

461.2000>45.0500 CE: 12.0

461.2000>309.1500 CE: 23.0

461.2000>325.1500 CE: 22.0

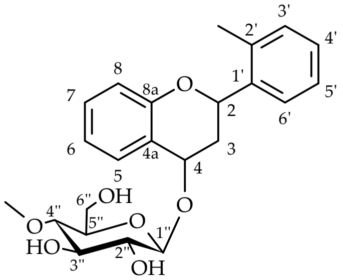

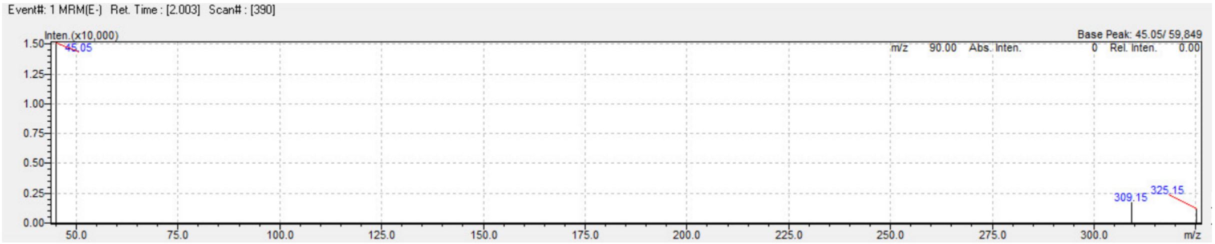

Figure S53. MS analysis of 2-(2′-methylphenyl)-chromane 4-*O*-*β*-D-(4′′-*O*-methyl)-glucopyranoside (**4c**)

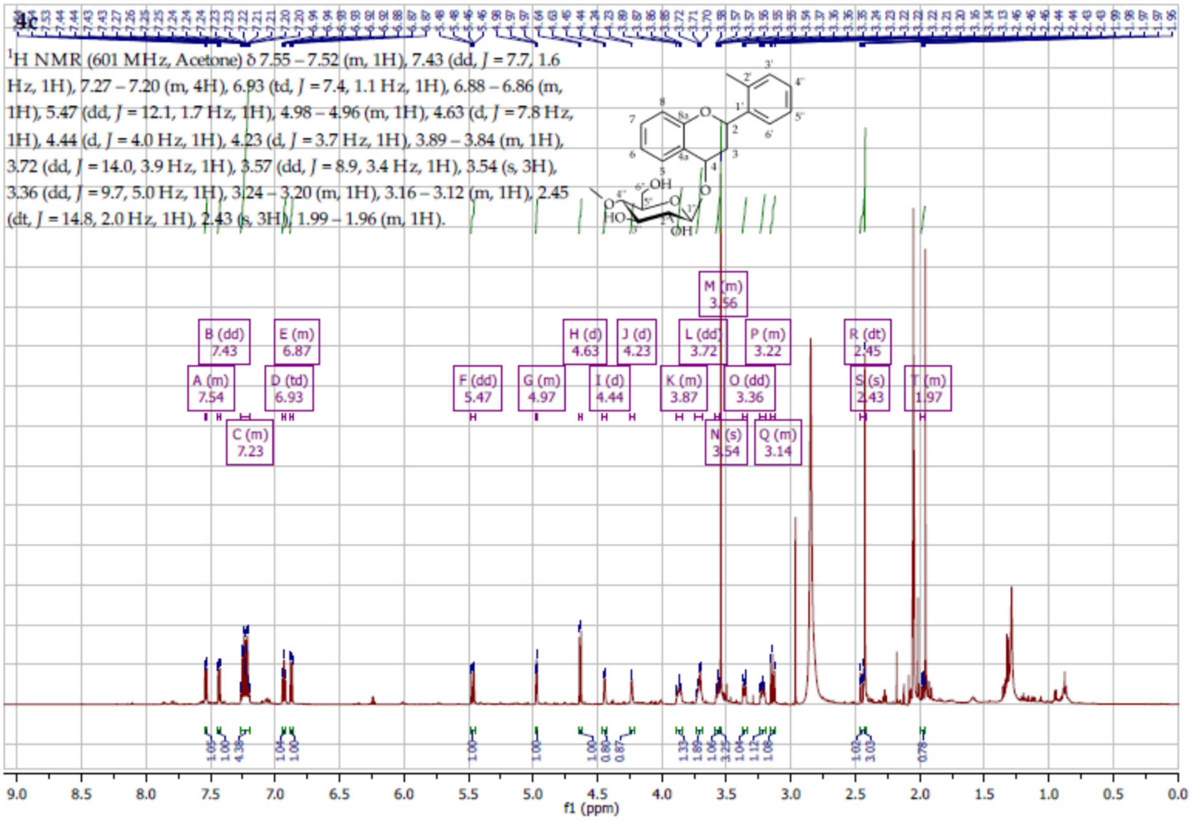

Figure S54. ¹H NMR spectrum (*δ*, acetone-d6, 600 MHz) of 2-(2′-methylphenyl)-chromane 4-*O*-*β*-D-(4′′-*O*-methyl)-glucopyranoside (**4c**)

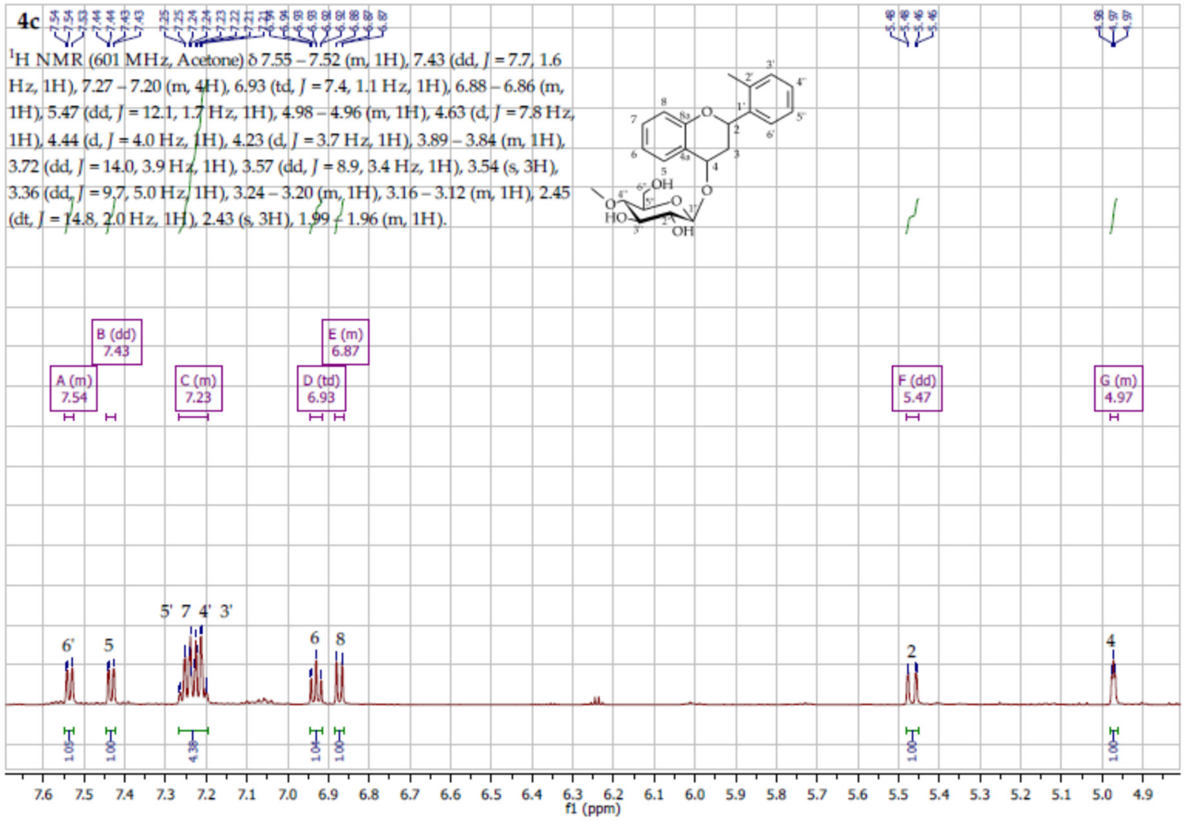

Figure S55. ^{1}H NMR spectrum expansion (δ, acetone-d6, 600 MHz) of 2-(2′-methylphenyl)-chromane 4-*O*-*β*-D-(4′′-*O*-methyl)-glucopyranoside (**4c**)

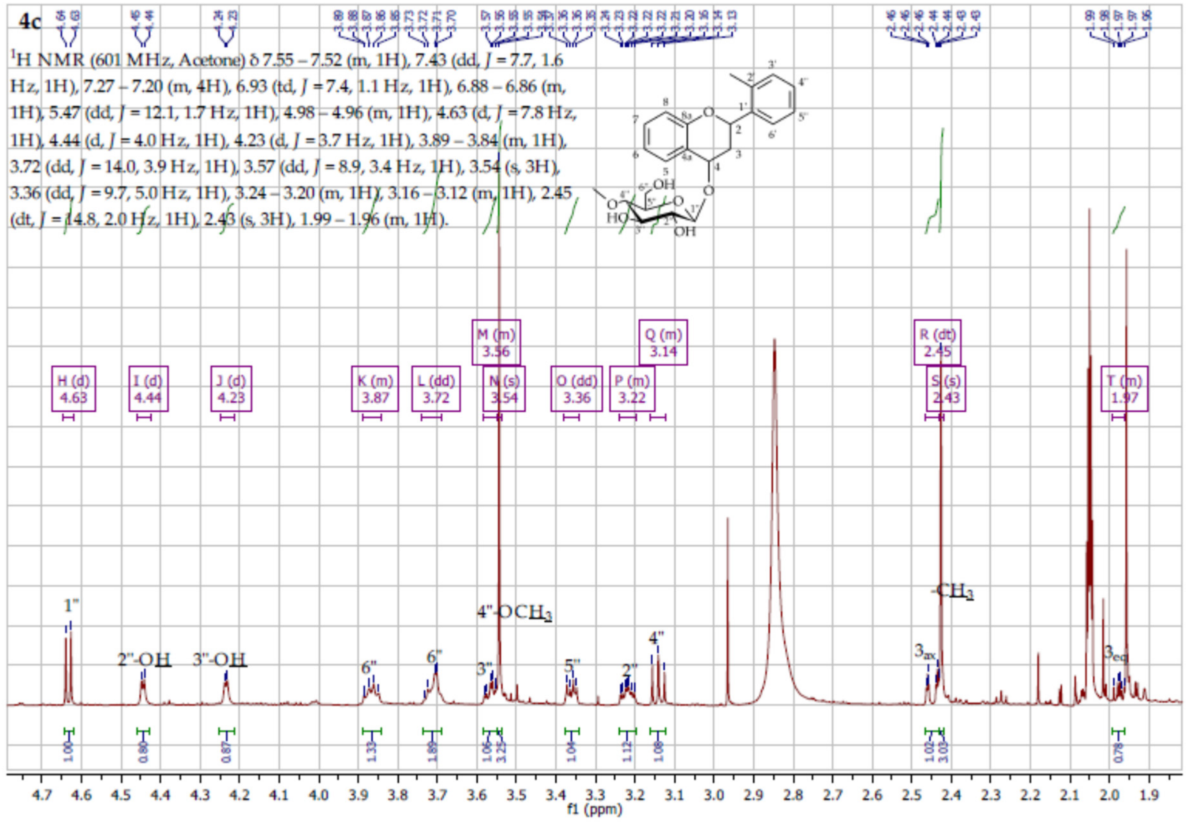

Figure S56. ^{1}H NMR spectrum expansion (δ, acetone-d6, 600 MHz) of 2-(2′-methylphenyl)-chromane 4-*O*-*β*-D-(4′′-*O*-methyl)-glucopyranoside (**4c**)

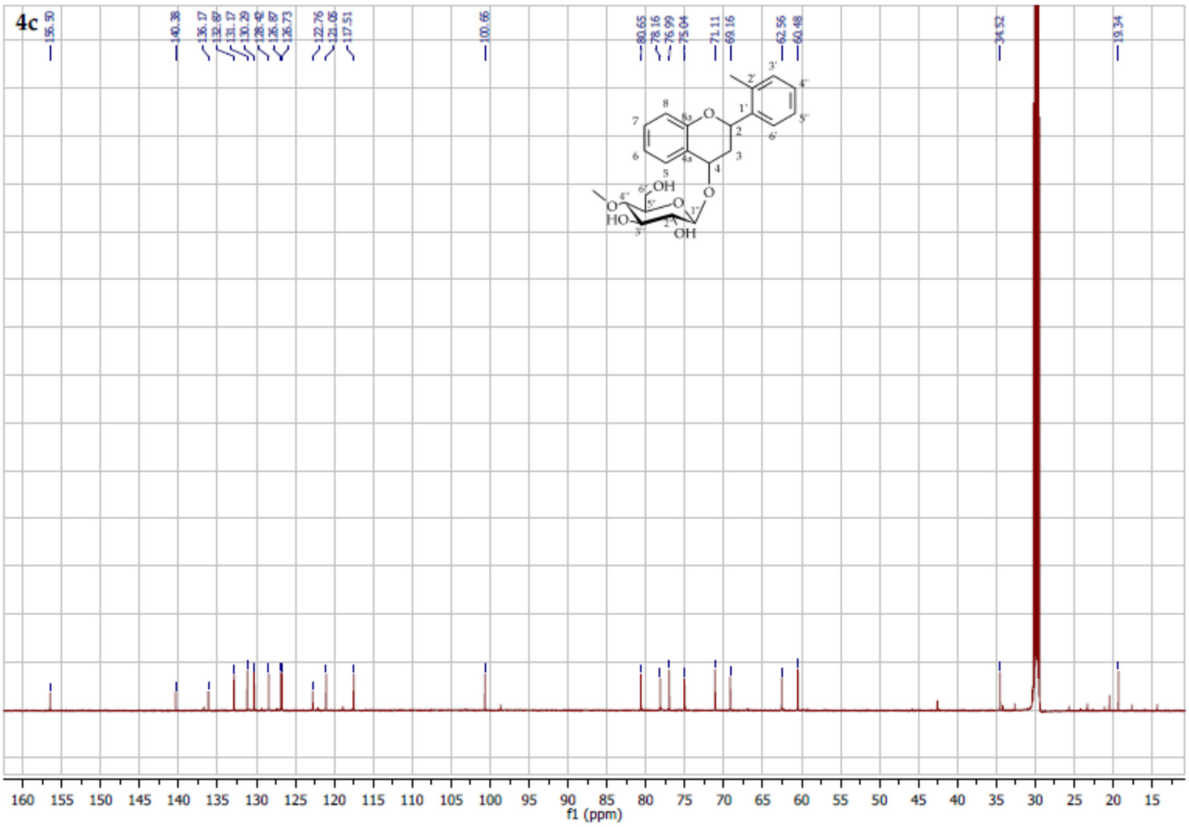

Figure S57. ^{13}C NMR spectrum (*δ*, acetone-d6, 151 MHz) of 2-(2′-methylphenyl)-chromane 4-*O*-*β*-D-(4′′-*O*-methyl)-glucopyranoside (**4c**)

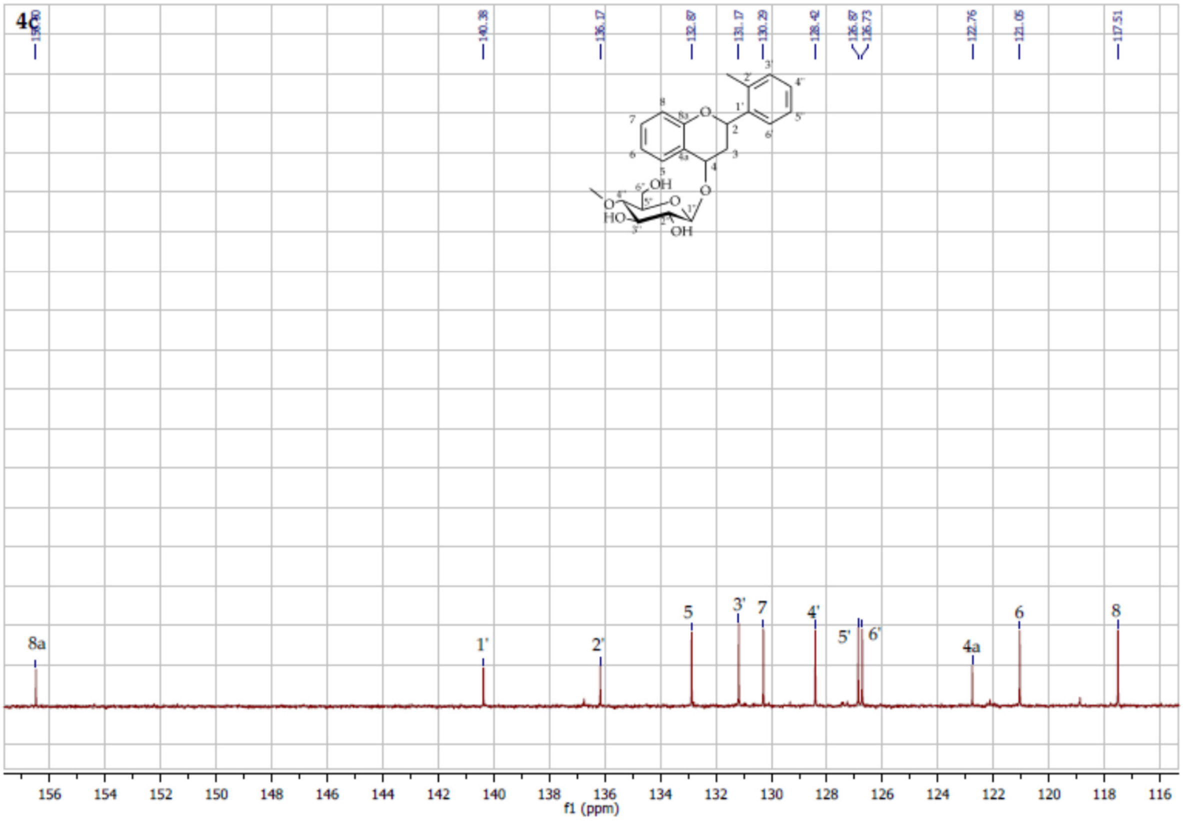

Figure S58. ^{13}C NMR spectrum expansion (*δ*, acetone-d6, 151 MHz) of 2-(2′-methylphenyl)-chromane 4-*O*-*β*-D-(4′′-*O*-methyl)-glucopyranoside (**4c**)

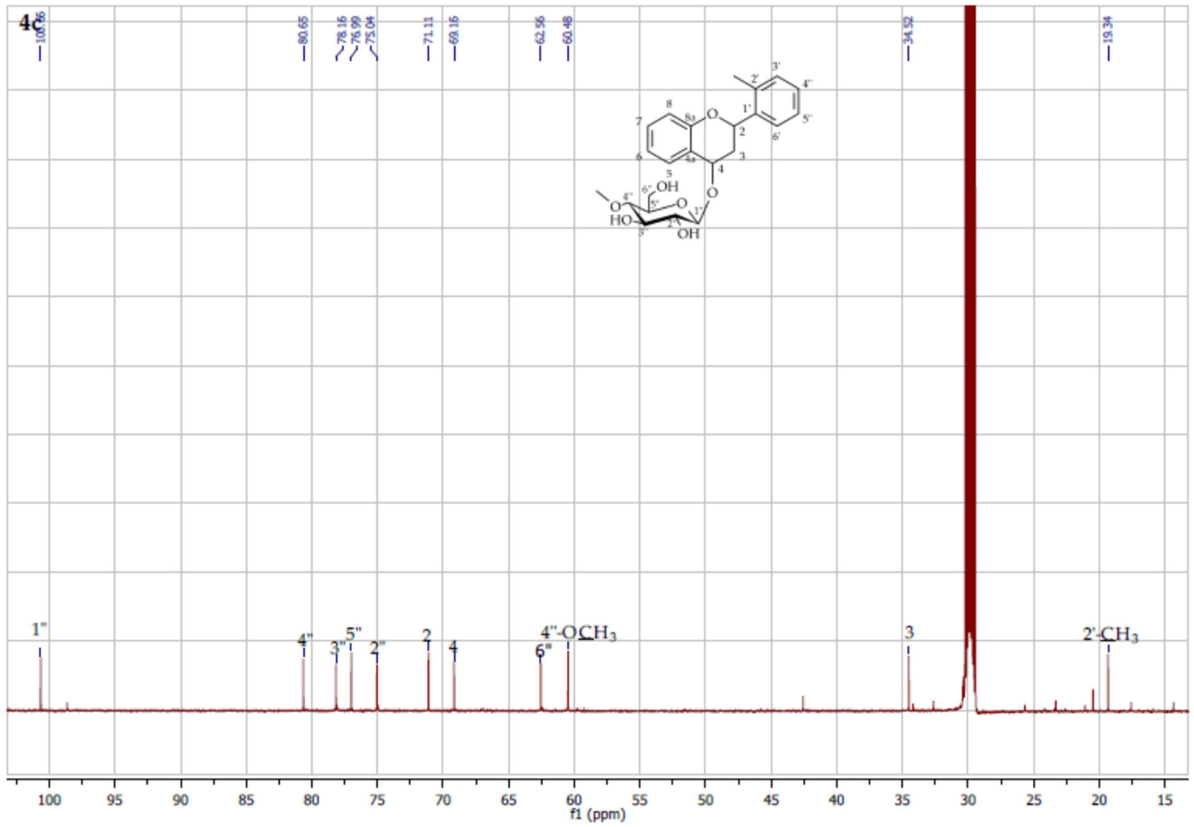

Figure S59. ^{13}C NMR spectrum expansion (δ, acetone-d6, 151 MHz) of 2-(2′-methylphenyl)-chromane 4-*O*-*β*-D-(4′′-*O*-methyl)-glucopyranoside (**4c**)

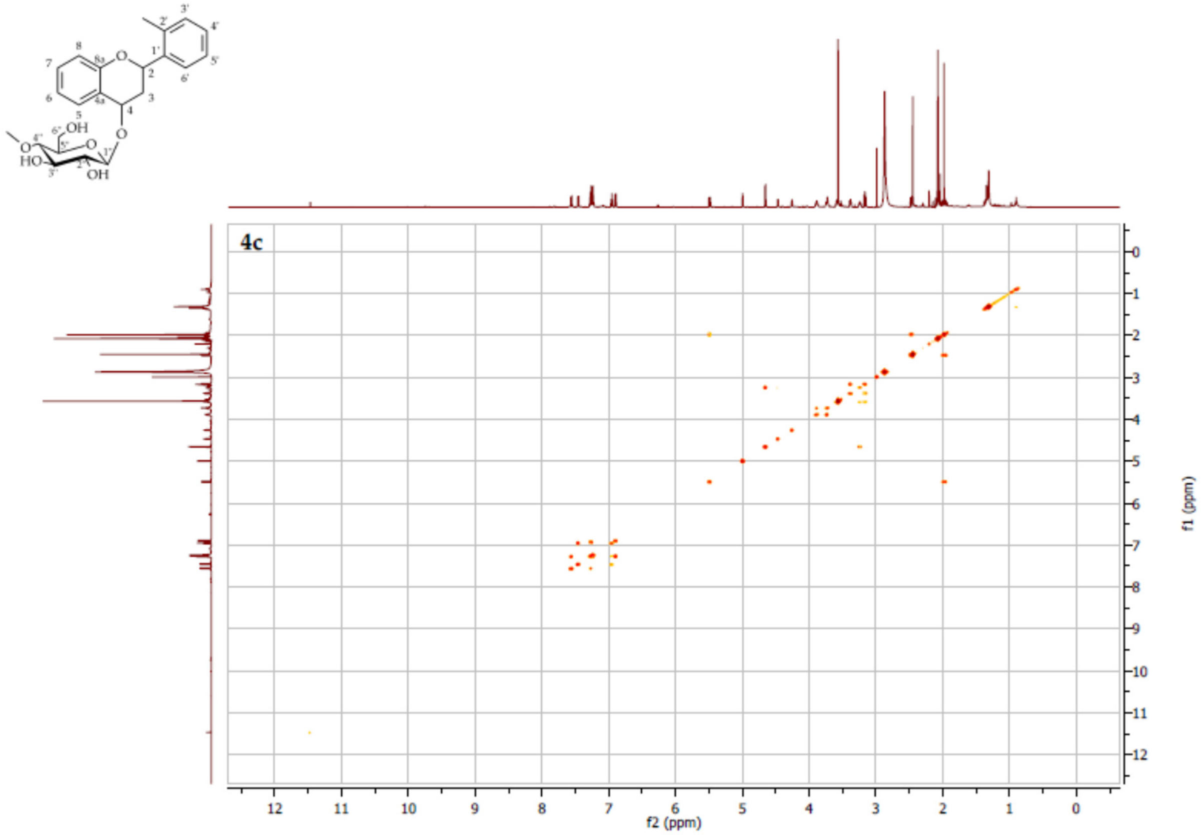

Figure S60. COSY contour map – ^{1}H x ^{1}H of 2-(2′-methylphenyl)-chromane 4-*O*-*β*-D-(4′′-*O*-methyl)-glucopyranoside (**4c**)

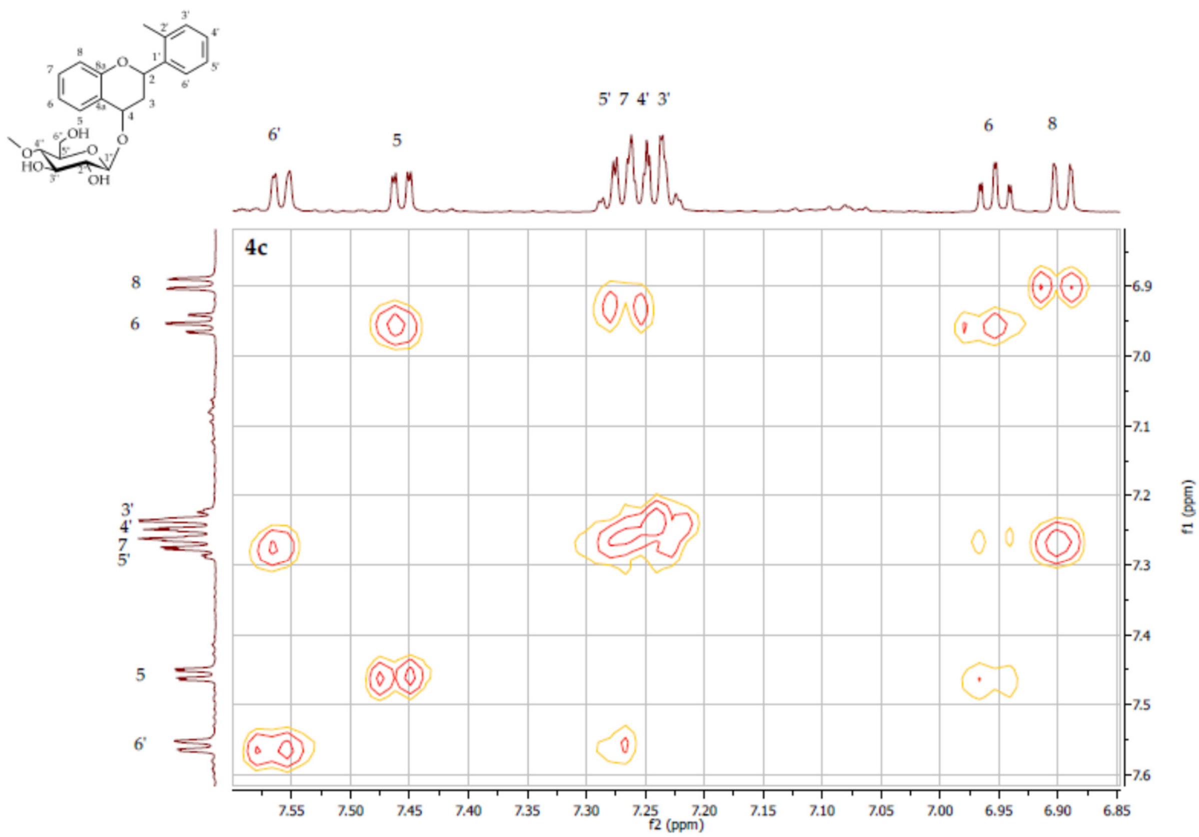

Figure S61. COSY contour map – ¹H x ¹H expansion of of 2-(2′-methylphenyl)-chromane 4-*O*-*β*-D-(4′′-*O*-methyl)-glucopyranoside (**4c**)

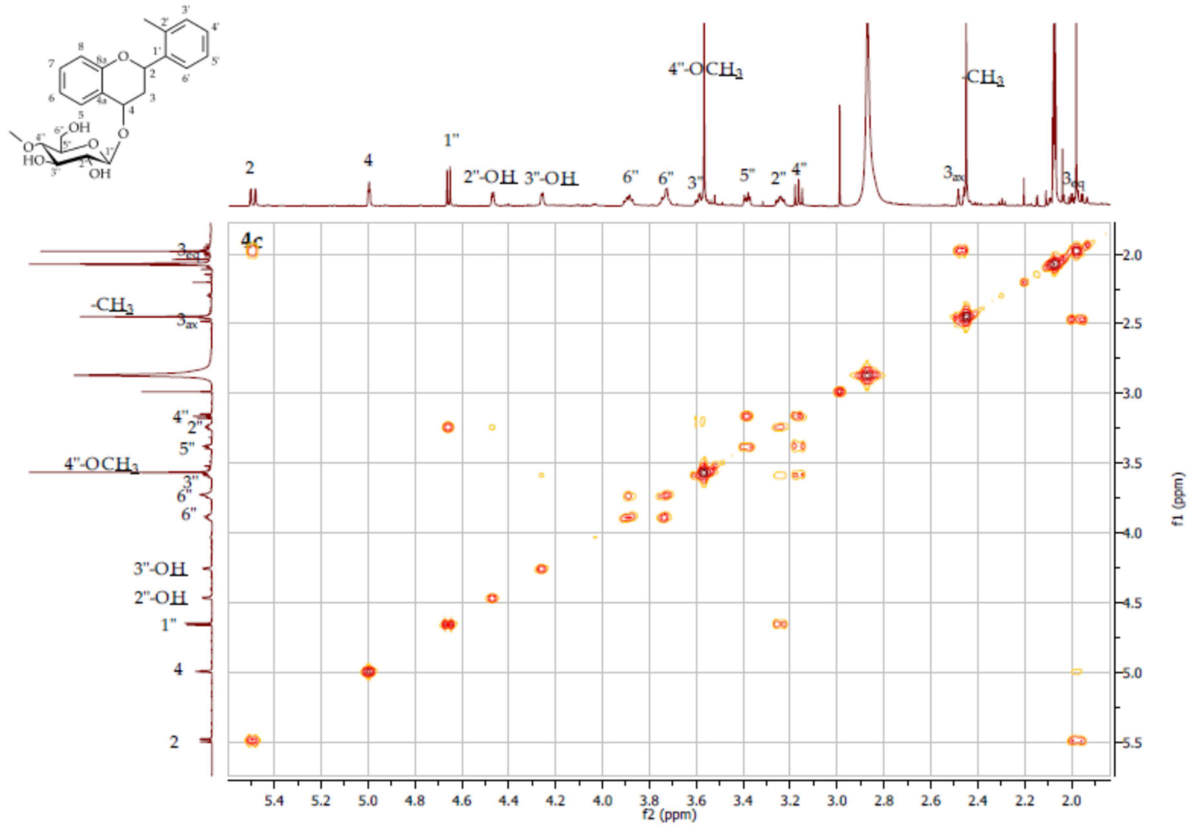

Figure S62. COSY contour map – ¹H x ¹H expansion of of 2-(2′-methylphenyl)-chromane 4-*O*-*β*-D-(4′′-*O*-methyl)-glucopyranoside (**4c**)

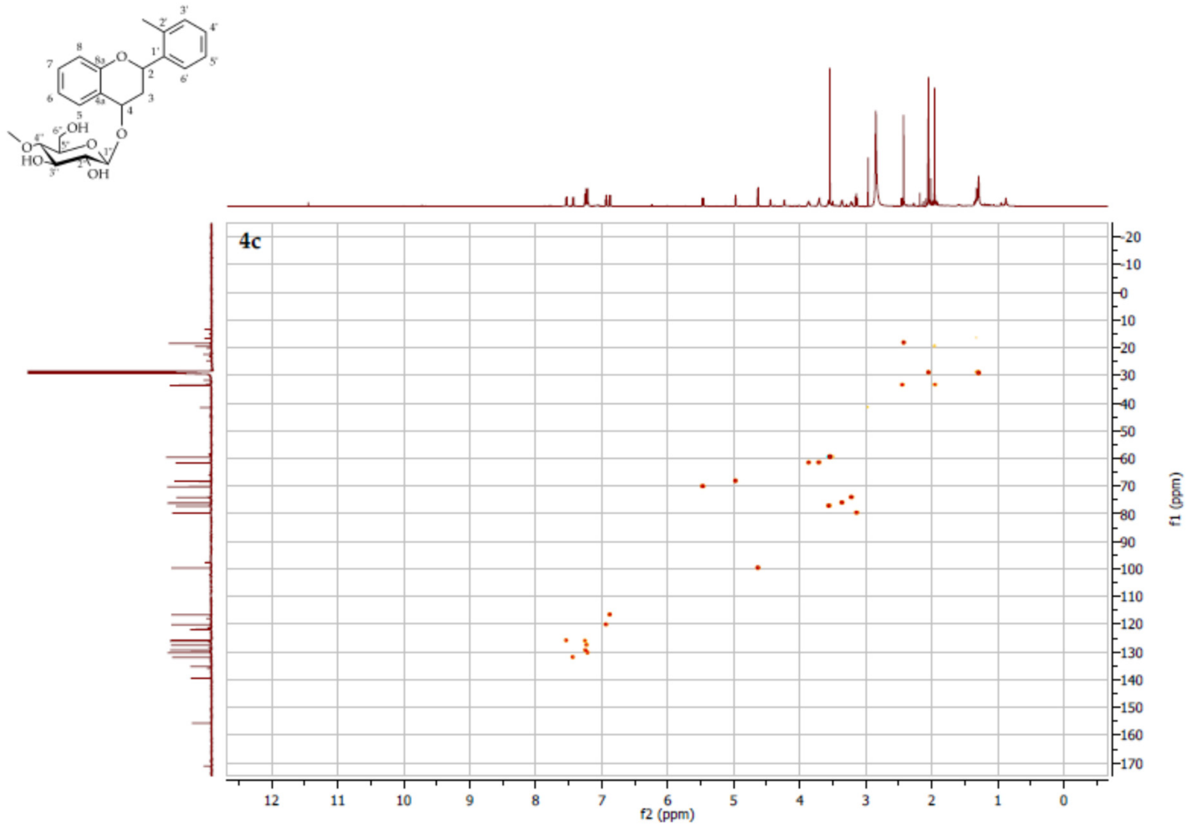

Figure S63. HSQC contour map – ^{1}H x ^{13}C of 2-(2′-methylphenyl)-chromane 4-*O*-*β*-D-(4′′-*O*-methyl)-glucopyranoside (**4c**)

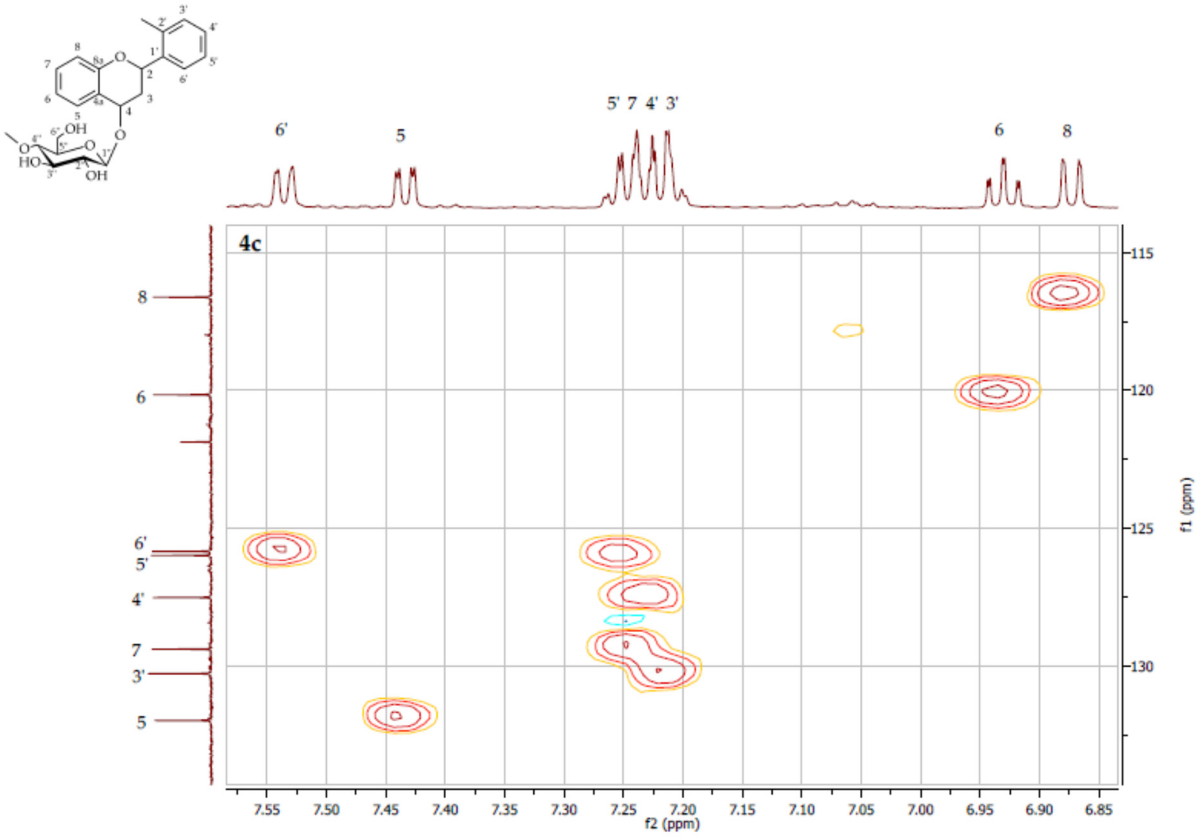

Figure S64. HSQC contour map – ^{1}H x ^{13}C expansion of 2-(2′-methylphenyl)-chromane 4-*O*-*β*-D-(4′′-*O*-methyl)-glucopyranoside (**4c**)

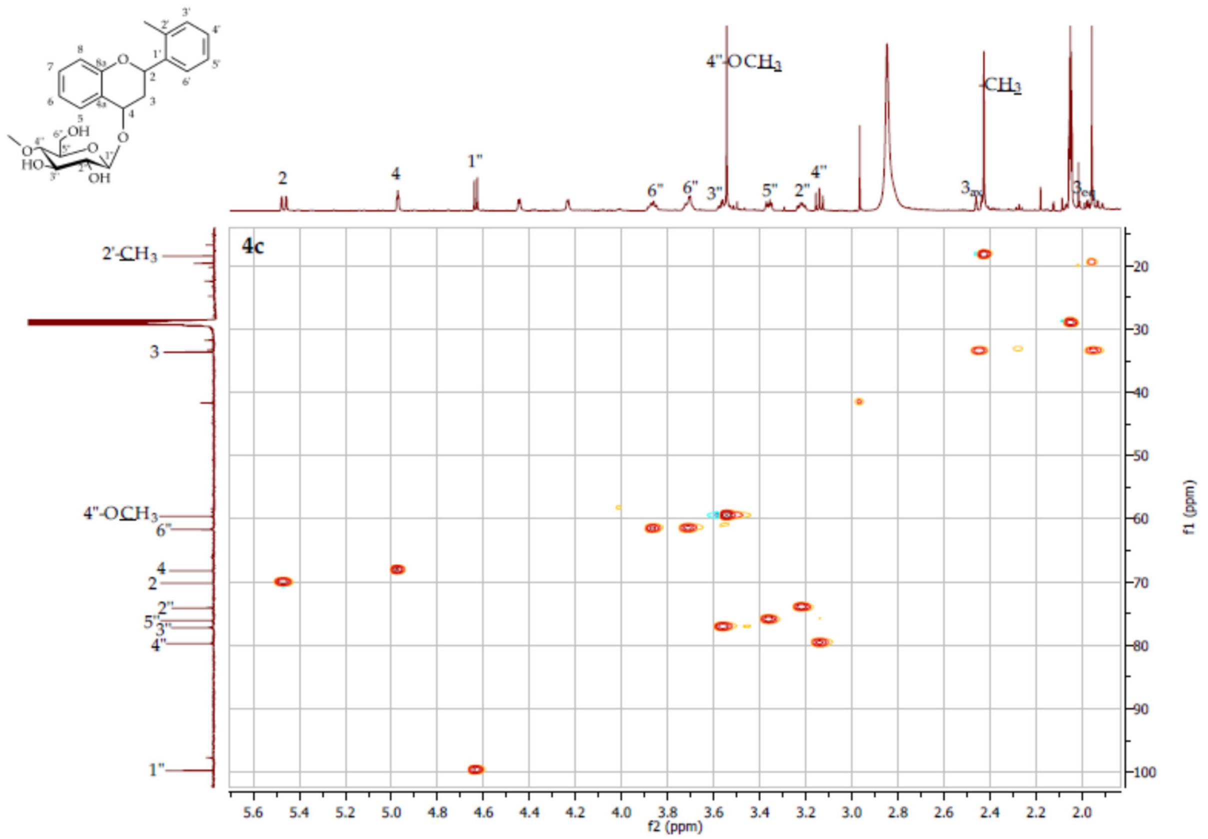

Figure S65. HSQC contour map – ^1H x ^{13}C expansion of 2-(2′-methylphenyl)-chromane 4-*O*-*β*-D-(4′′-*O*-methyl)-glucopyranoside (**4c**)

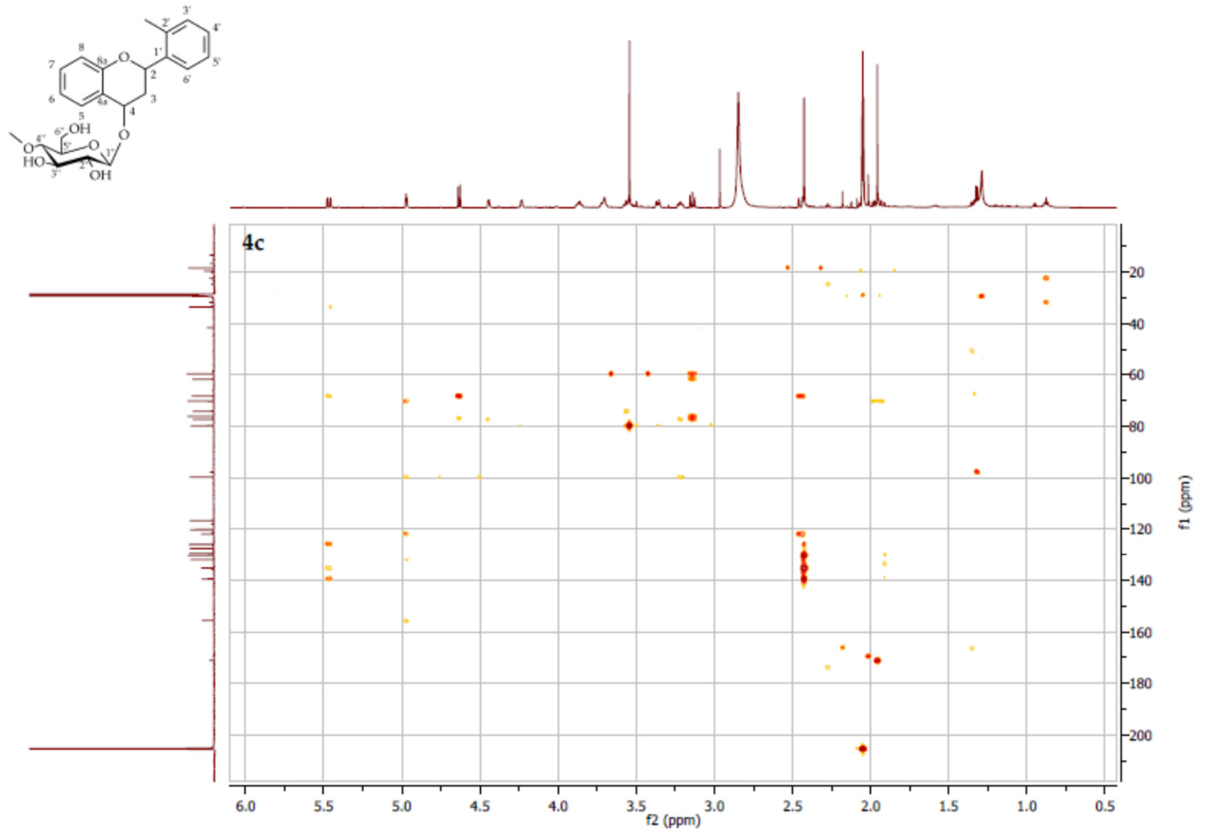

Figure S66. HMBC contour map – ^1H x ^{13}C of 2-(2′-methylphenyl)-chromane 4-*O*-*β*-D-(4′′-*O*-methyl)-glucopyranoside (**4c**)

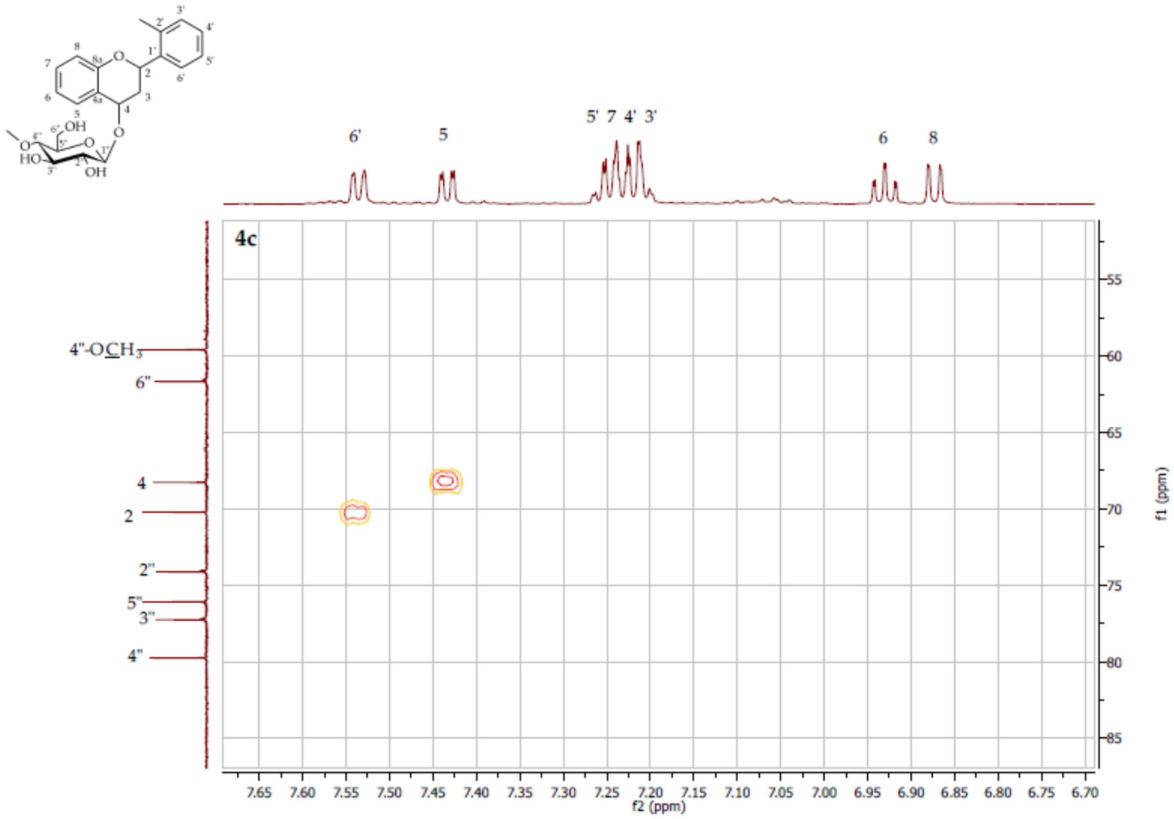

Figure S67. HMBC contour map – ^{1}H x ^{13}C expansion of 2-(2′-methylphenyl)-chromane 4-*O*-*β*-D-(4′′-*O*-methyl)-glucopyranoside (**4c**)

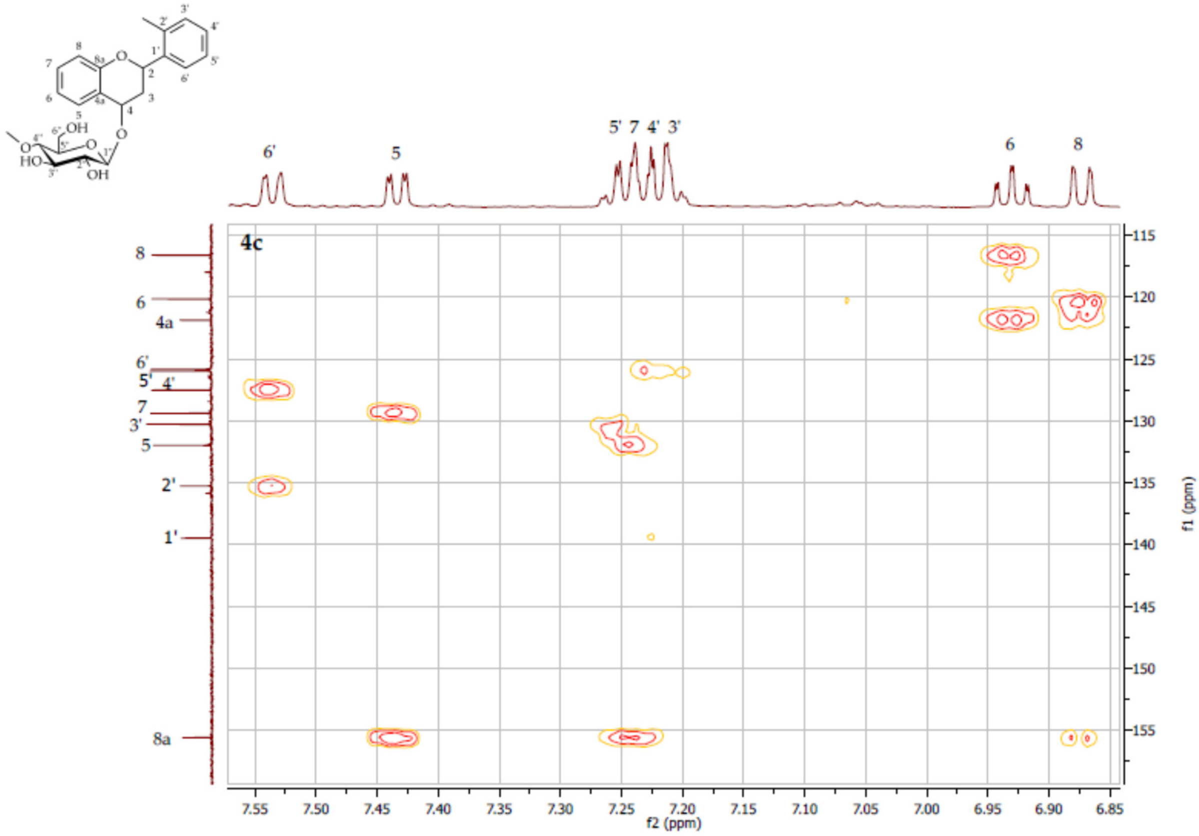

Figure S68. HMBC contour map – ^{1}H x ^{13}C expansion of 2-(2′-methylphenyl)-chromane 4-*O*-*β*-D-(4′′-*O*-methyl)-glucopyranoside (**4c**)

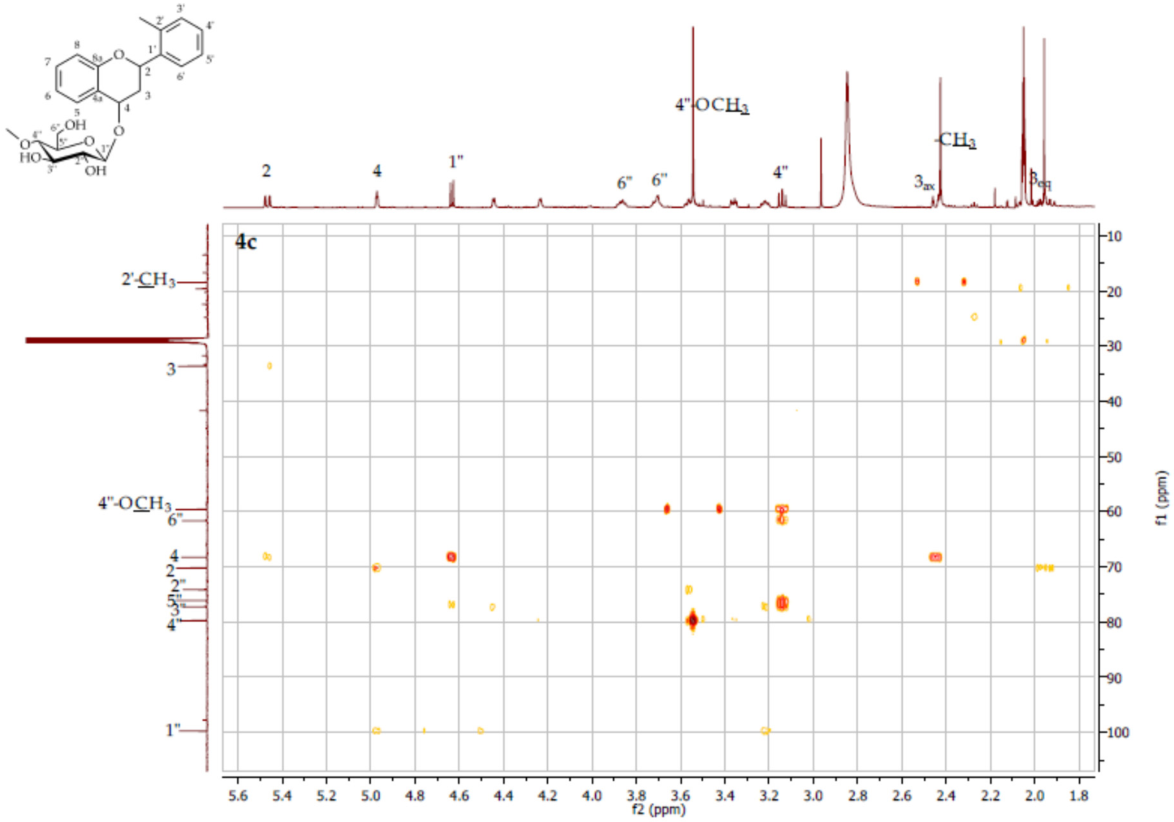

Figure S69. HMBC contour map – ^{1}H x ^{13}C expansion of 2-(2′-methylphenyl)-chromane 4-*O*-*β*-D-(4′′-*O*-methyl)-glucopyranoside (**4c**)

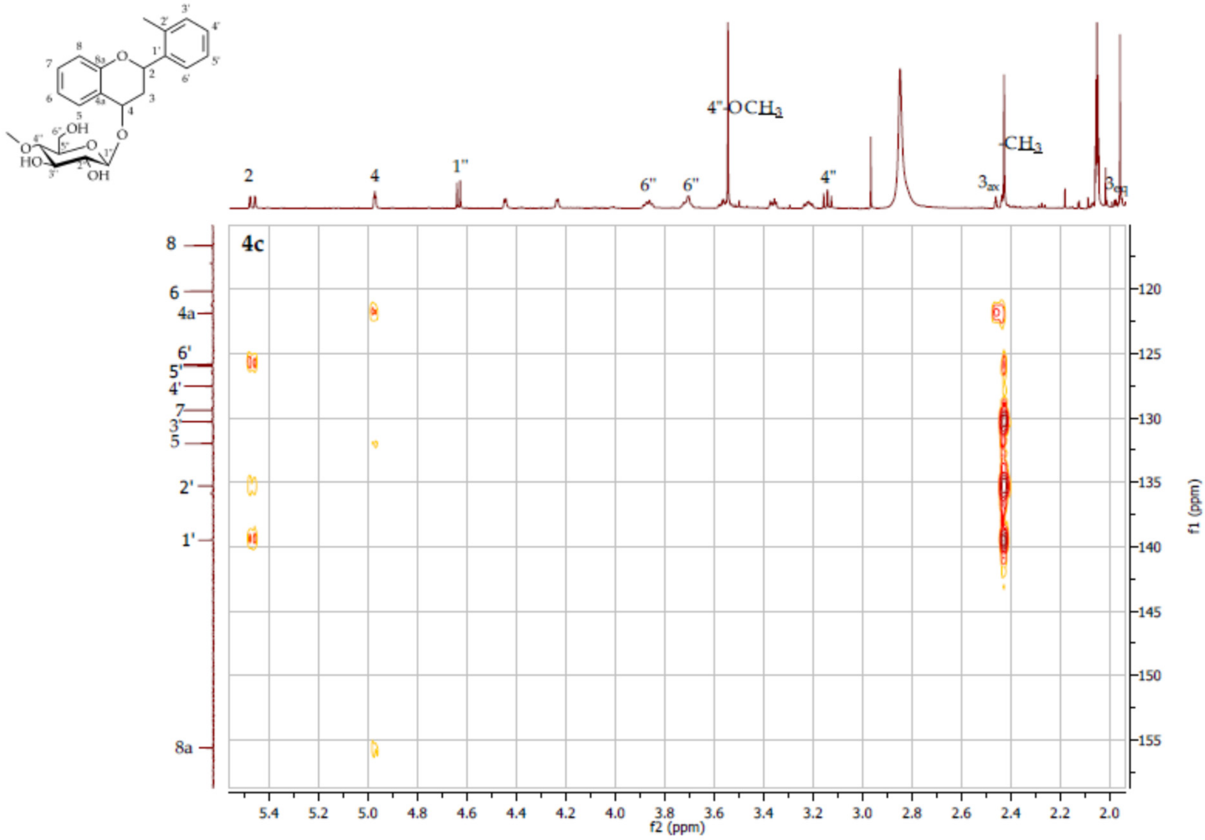

Figure S70. HMBC contour map – ^{1}H x ^{13}C expansion of 2-(2′-methylphenyl)-chromane 4-*O*-*β*-D-(4′′-*O*-methyl)-glucopyranoside (**4c**)

Molecular formula: $C_{23}H_{26}O_8$

Formula weight: 430.16

Ionization mode: positive

Precursor: $[M + H]^+$ 431.20

431.2000>390.0500 CE: -8.0

431.2000>312.3000 CE: -23.0

431.2000>255.1000 CE: -15.0

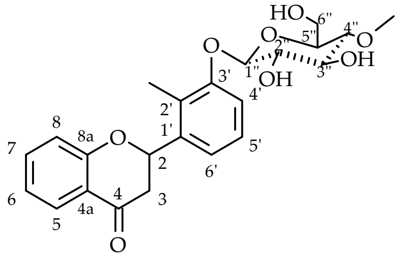

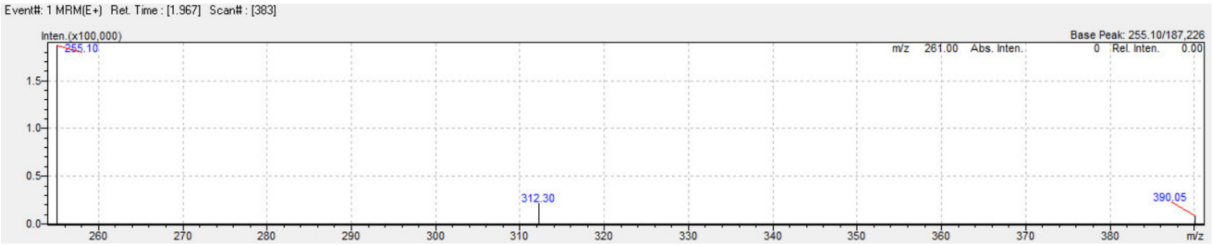

Figure S71. MS analysis of 2′-methylflavanone 3′-*O*-*β*-D-(4′′-*O*-methyl)-glucopyranoside (**4d**)

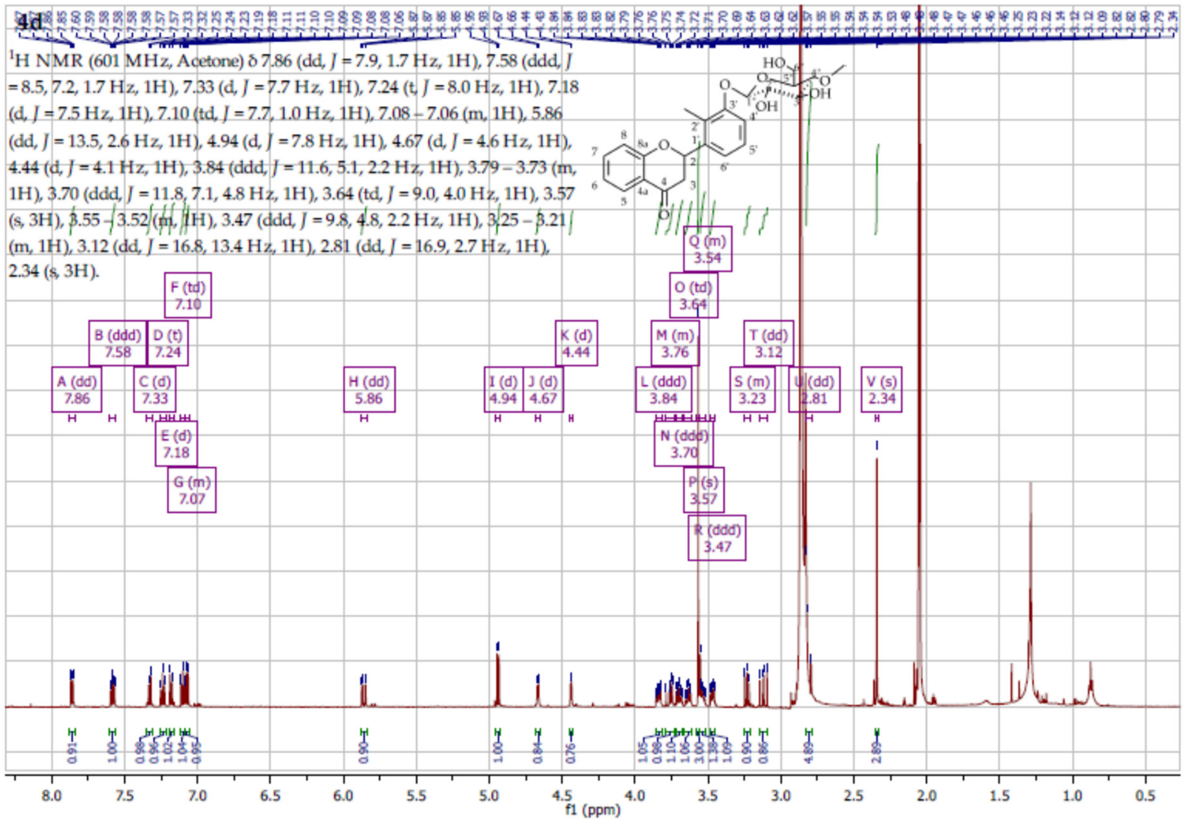

Figure S72. ^{1}H NMR spectrum (*δ*, acetone-d6, 600 MHz) of 2′-methylflavanone 3′-*O*-*β*-D-(4′′-*O*-methyl)-glucopyranoside (**4d**)

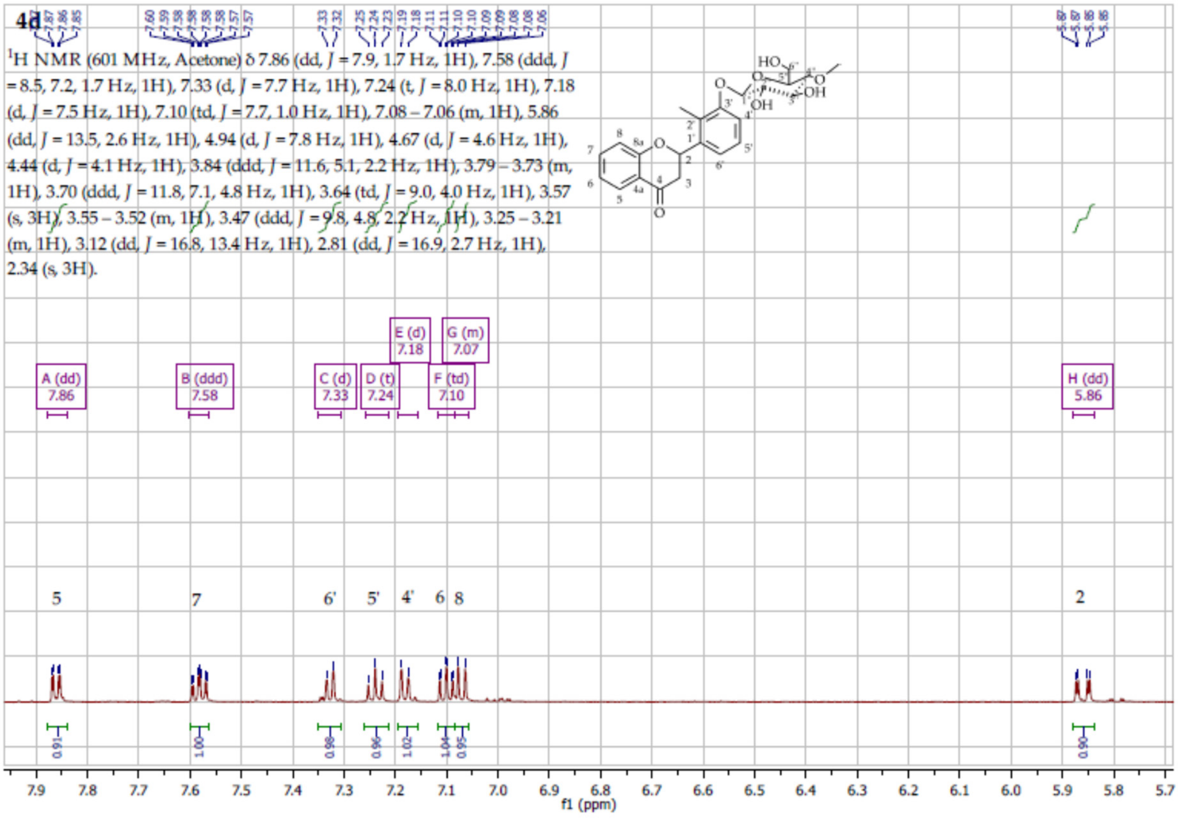

Figure S73. ^{1}H NMR spectrum expansion (*δ*, acetone-d6, 600 MHz) of 2′-methylflavanone 3′-*O*-*β*-D-(4′′-*O*-methyl)-glucopyranoside (**4d**)

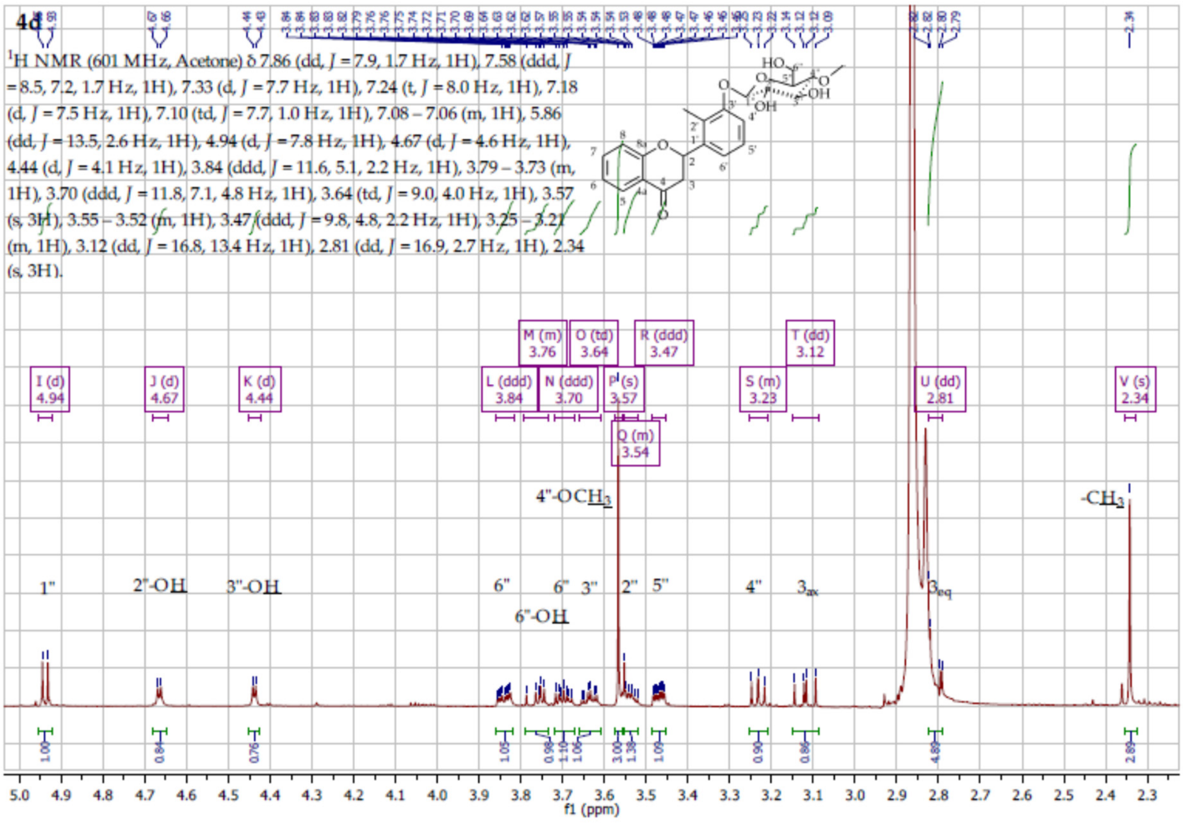

Figure S74. ^{1}H NMR spectrum expansion (*δ*, acetone-d6, 600 MHz) of 2′-methylflavanone 3′-*O*-*β*-D-(4′′-*O*-methyl)-glucopyranoside (**4d**)

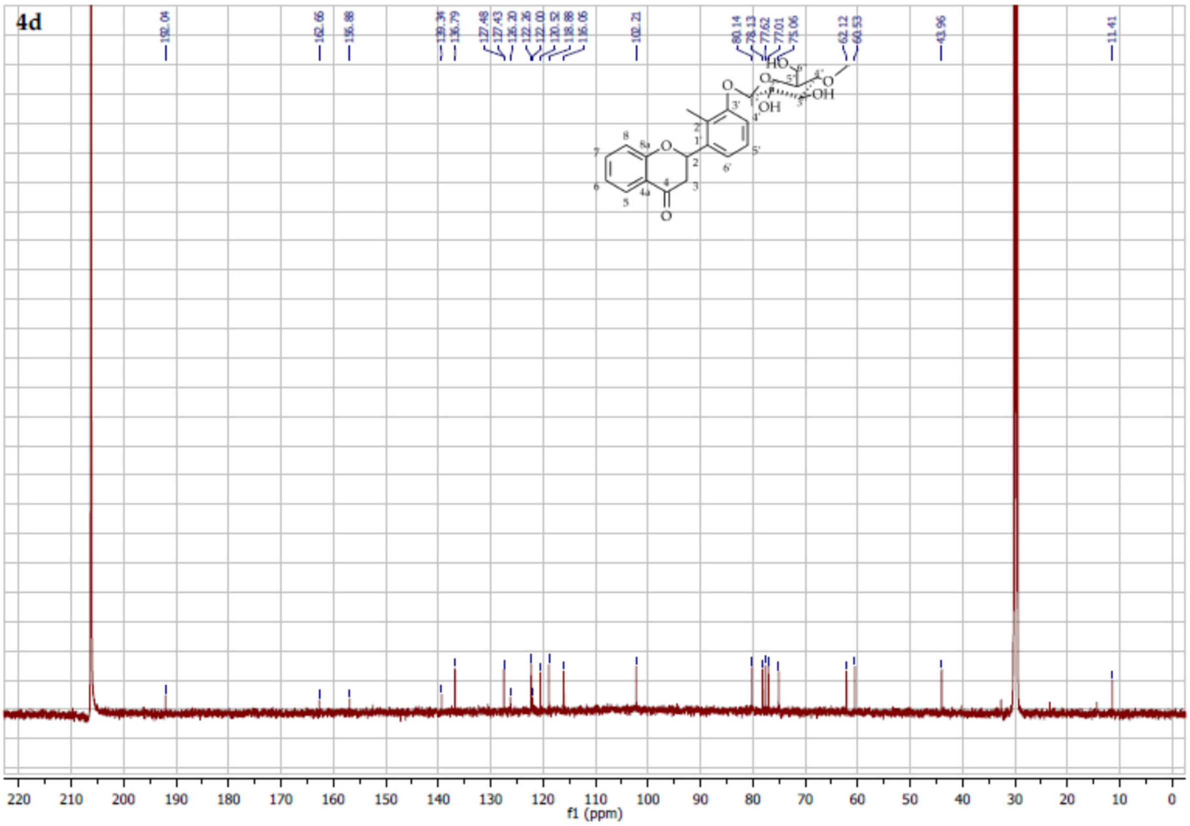

Figure S75. ^{13}C NMR spectrum (*δ*, acetone-d6, 151 MHz) of 2′-methylflavanone 3′-*O*-*β*-D-(4′′-*O*-methyl)-glucopyranoside (**4d**)

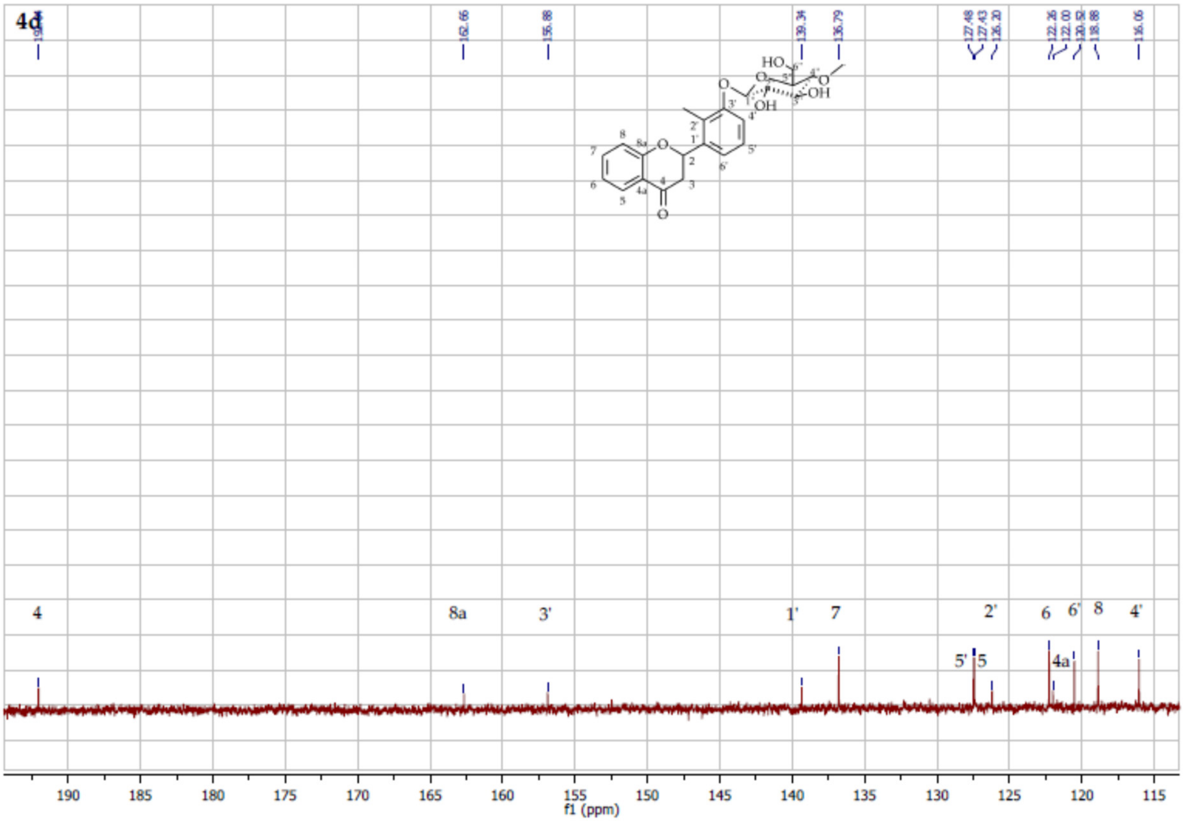

Figure S76. ^{13}C NMR spectrum expansion (*δ*, acetone-d6, 151 MHz) of 2′-methylflavanone 3′-*O*-*β*-D-(4′′-*O*-methyl)-glucopyranoside (**4d**)

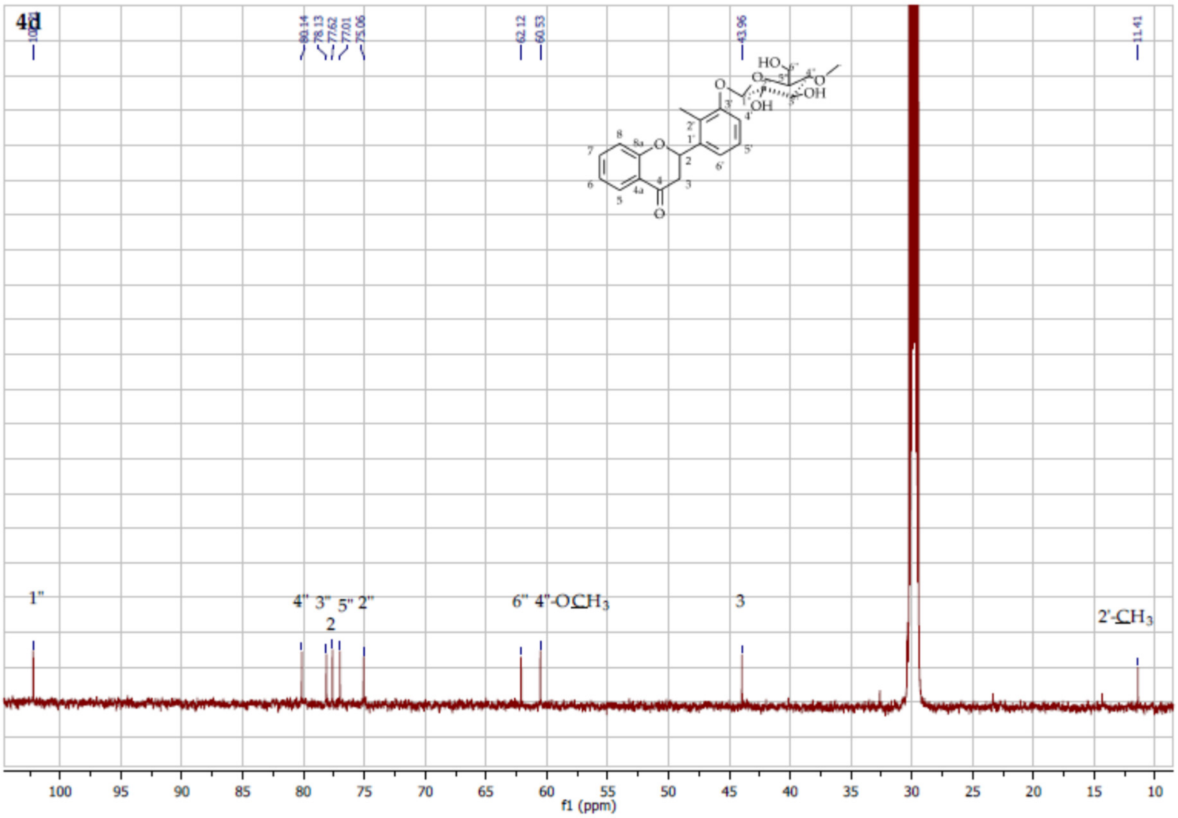

Figure S77. ^{13}C NMR spectrum expansion (δ, acetone-d6, 151 MHz) of 2′-methylflavanone 3′-*O*-*β*-D-(4′′-*O*-methyl)-glucopyranoside (**4d**)

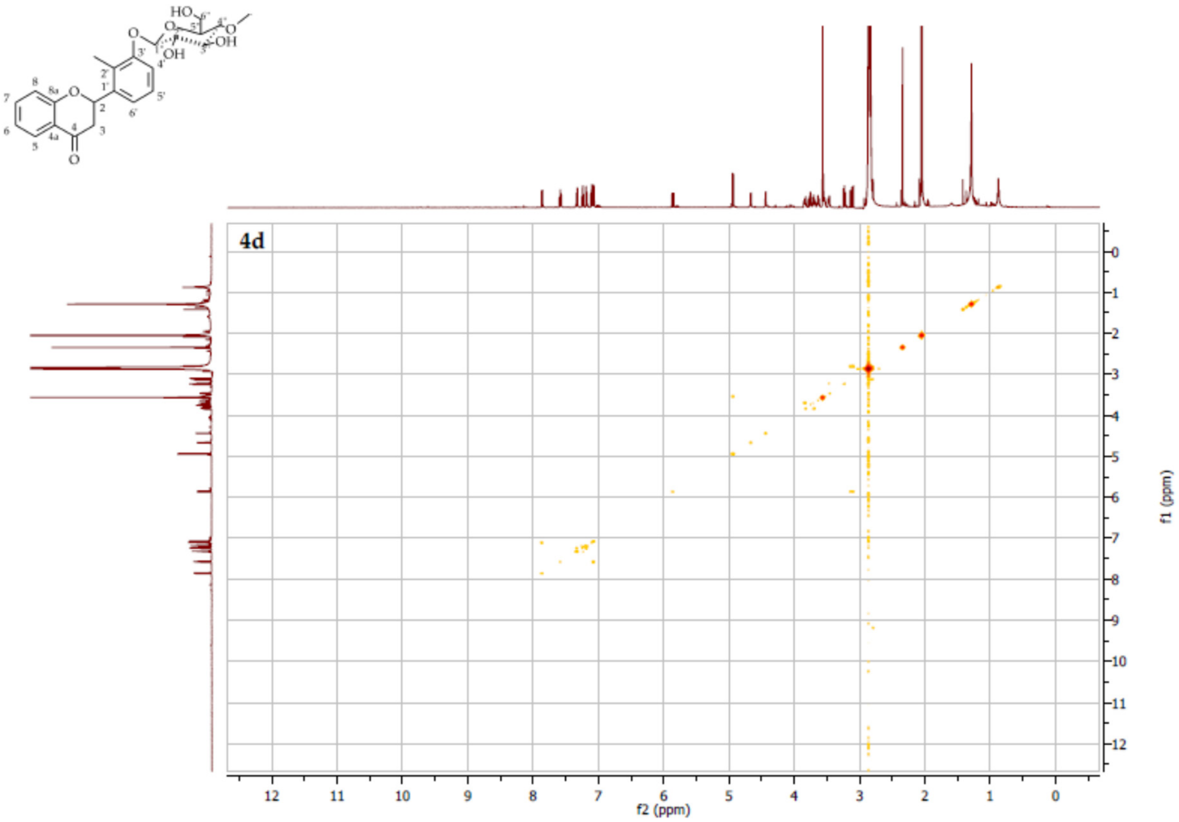

Figure S78. COSY contour map – ^{1}H x ^{1}H of 2′-methylflavanone 3′-*O*-*β*-D-(4′′-*O*-methyl)-glucopyranoside (**4d**)

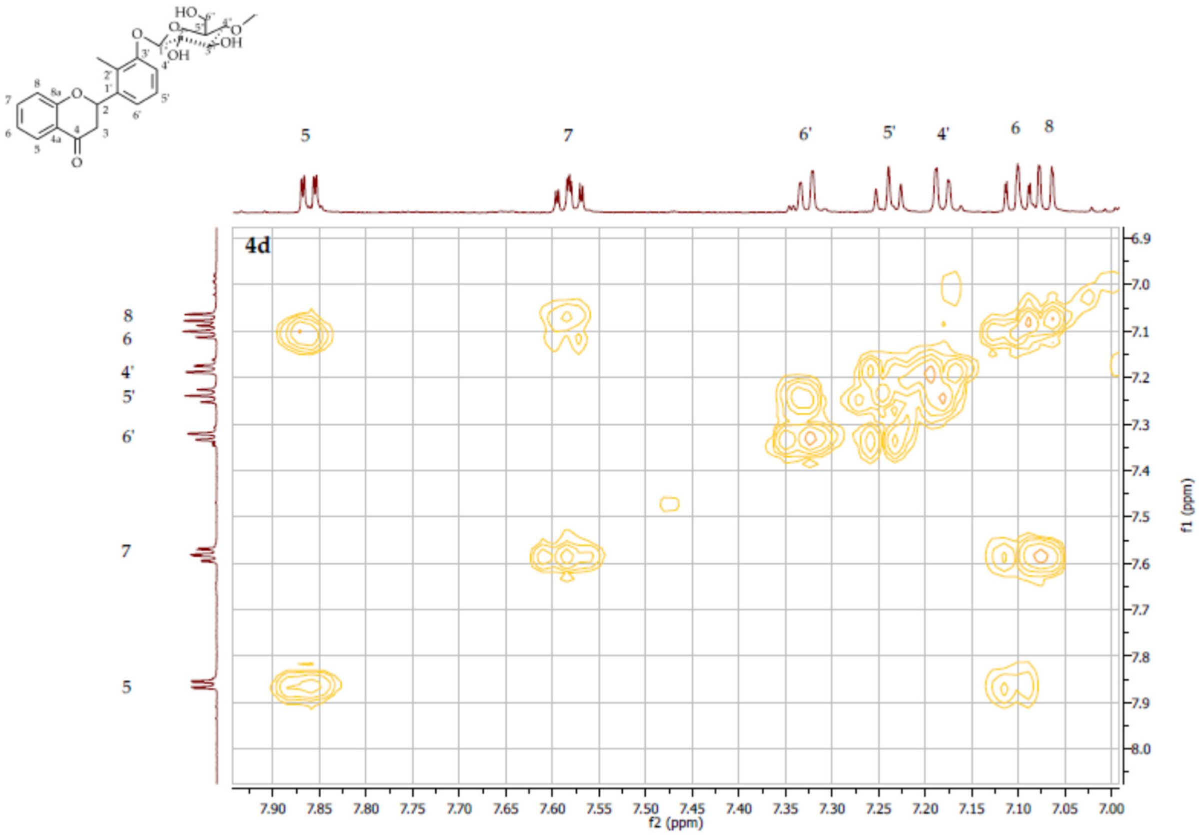

Figure S79. COSY contour map – ¹H x ¹H expansion of 2′-methylflavanone 3′-*O*-*β*-D-(4′′-*O*-methyl)-glucopyranoside (**4d**)

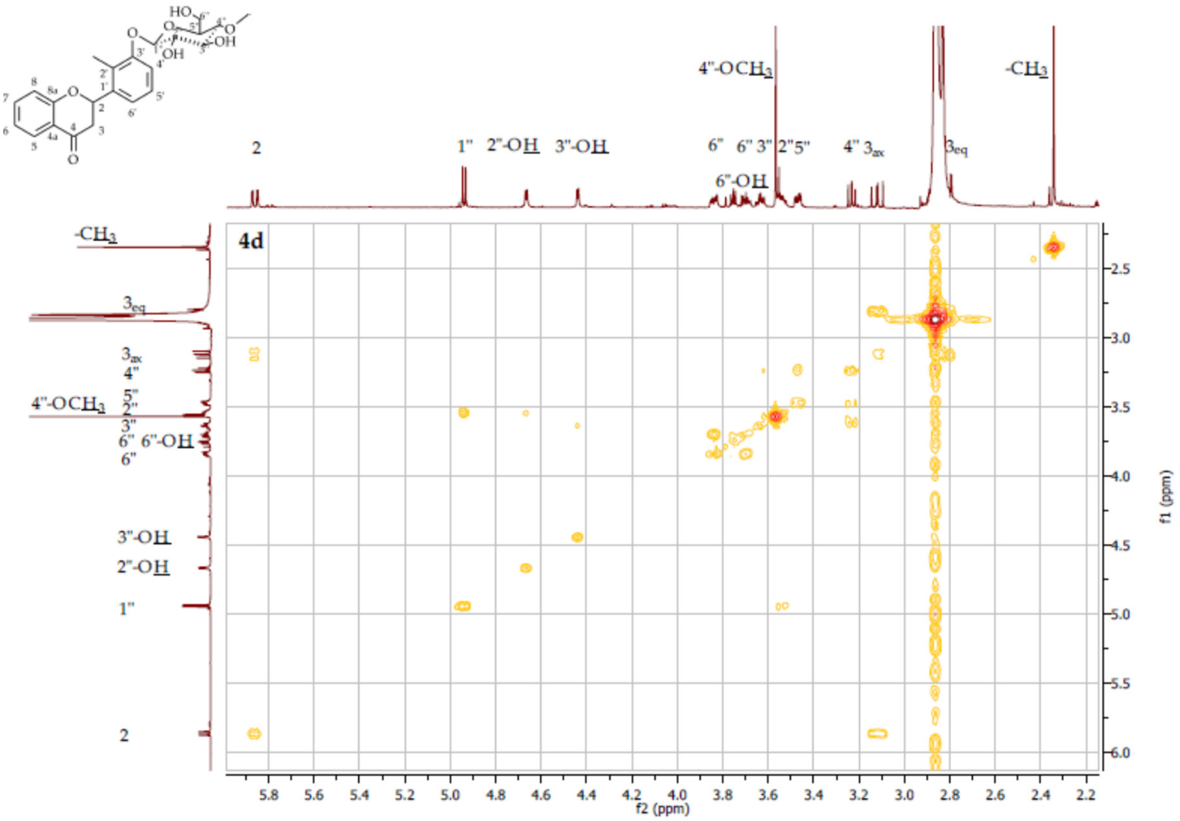

Figure S80. COSY contour map – ¹H x ¹H expansion of 2′-methylflavanone 3′-*O*-*β*-D-(4′′-*O*-methyl)-glucopyranoside (**4d**)

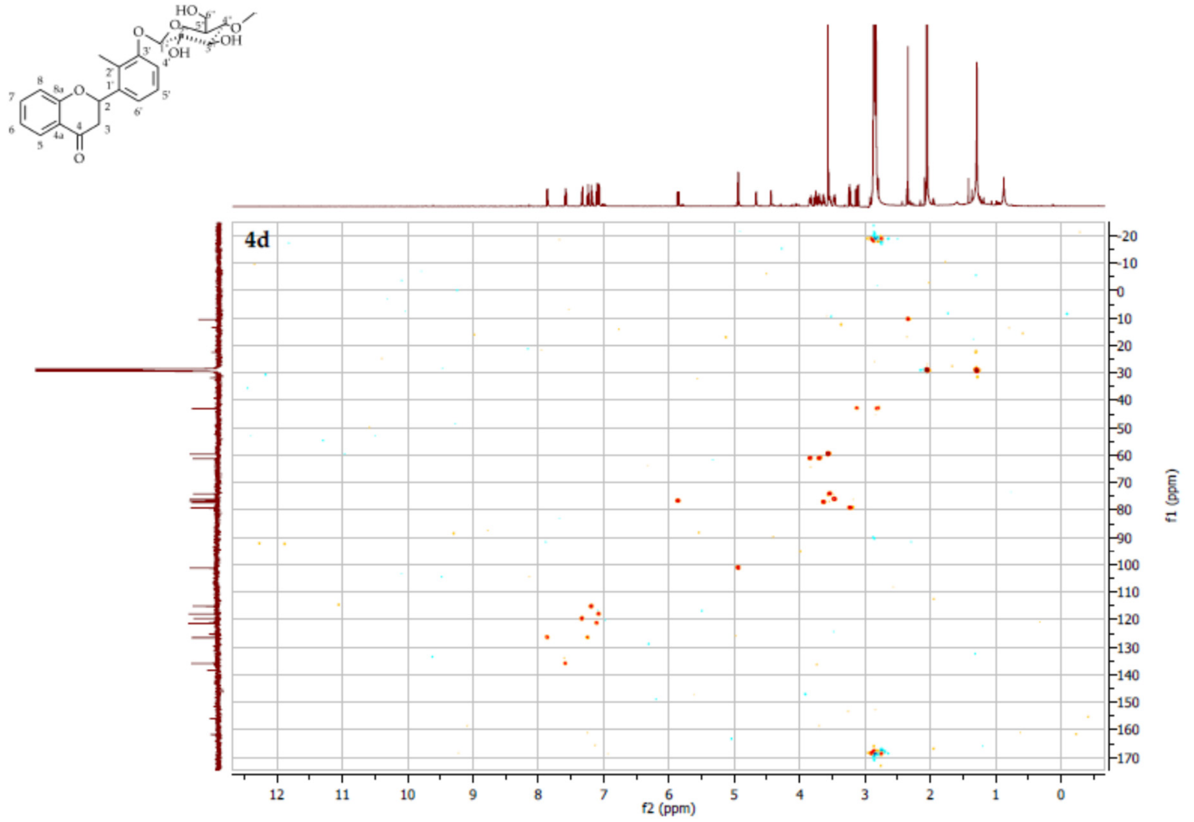

Figure S81. HSQC contour map – ^{1}H x ^{13}C of 2′-methylflavanone 3′-*O*-*β*-D-(4′′-*O*-methyl)-glucopyranoside (**4d**)

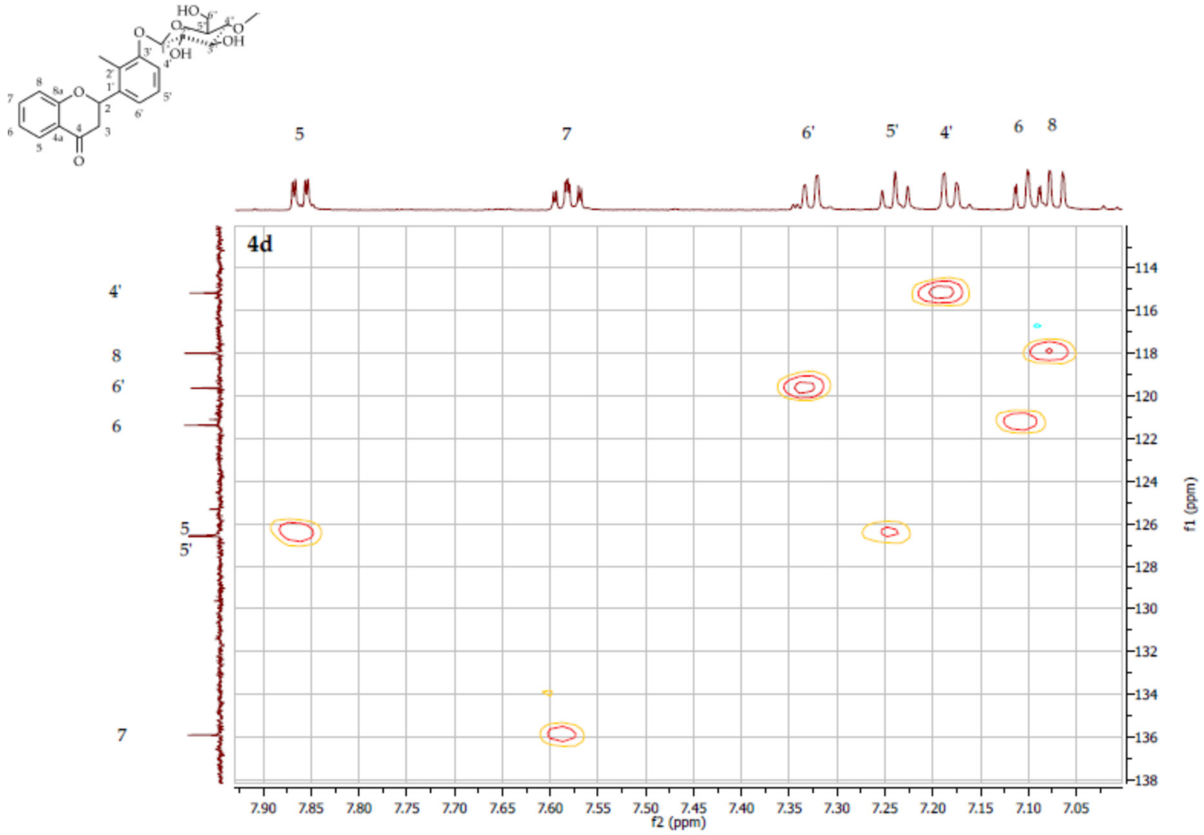

Figure S82. HSQC contour map – ^{1}H x ^{13}C expansion of 2′-methylflavanone 3′-*O*-*β*-D-(4′′-*O*-methyl)-glucopyranoside (**4d**)

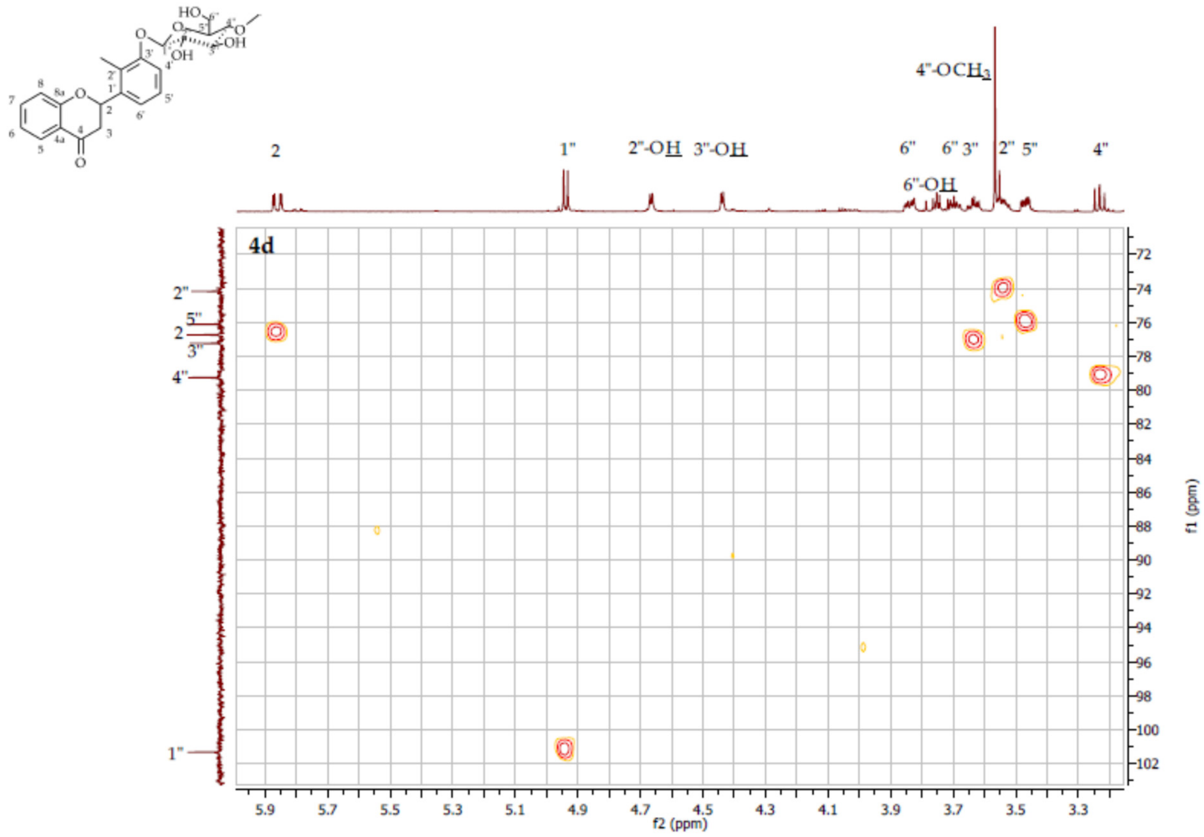

Figure S83. HSQC contour map – ^{1}H x ^{13}C expansion of 2′-methylflavanone 3′-*O*-*β*-D-(4′′-*O*-methyl)-glucopyranoside (**4d**)

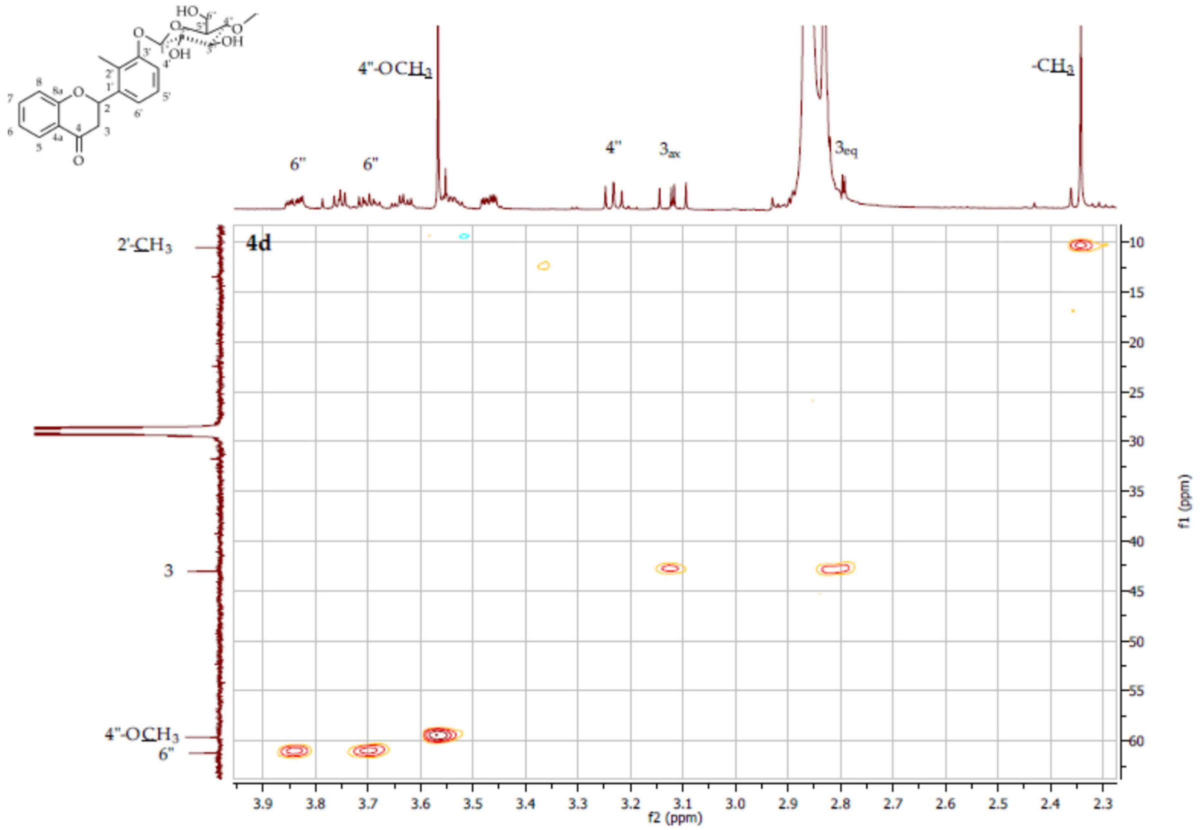

Figure S84. HSQC contour map – ^{1}H x ^{13}C expansion of 2′-methylflavanone 3′-*O*-*β*-D-(4′′-*O*-methyl)-glucopyranoside (**4d**)

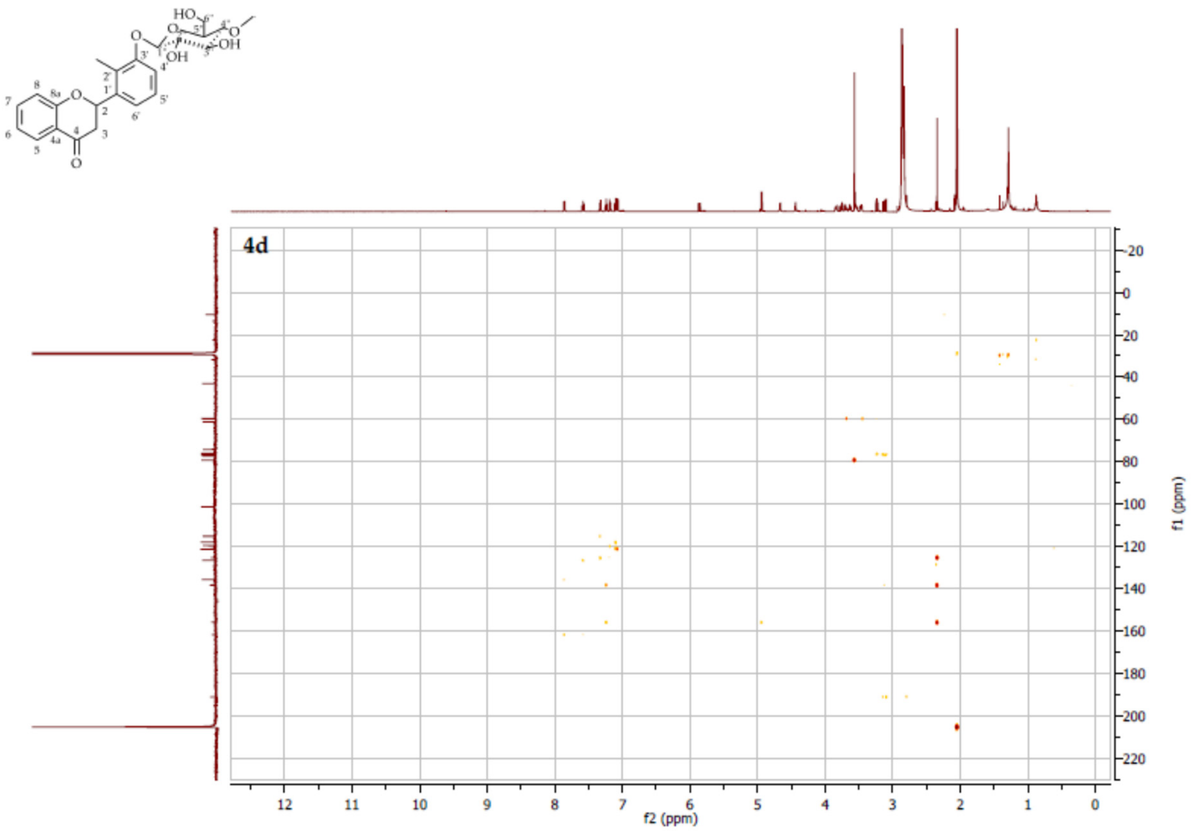

Figure S85. HMBC contour map – ^{1}H x ^{13}C of 2′-methylflavanone 3′-*O*-*β*-D-(4′′-*O*-methyl)-glucopyranoside (**4d**)

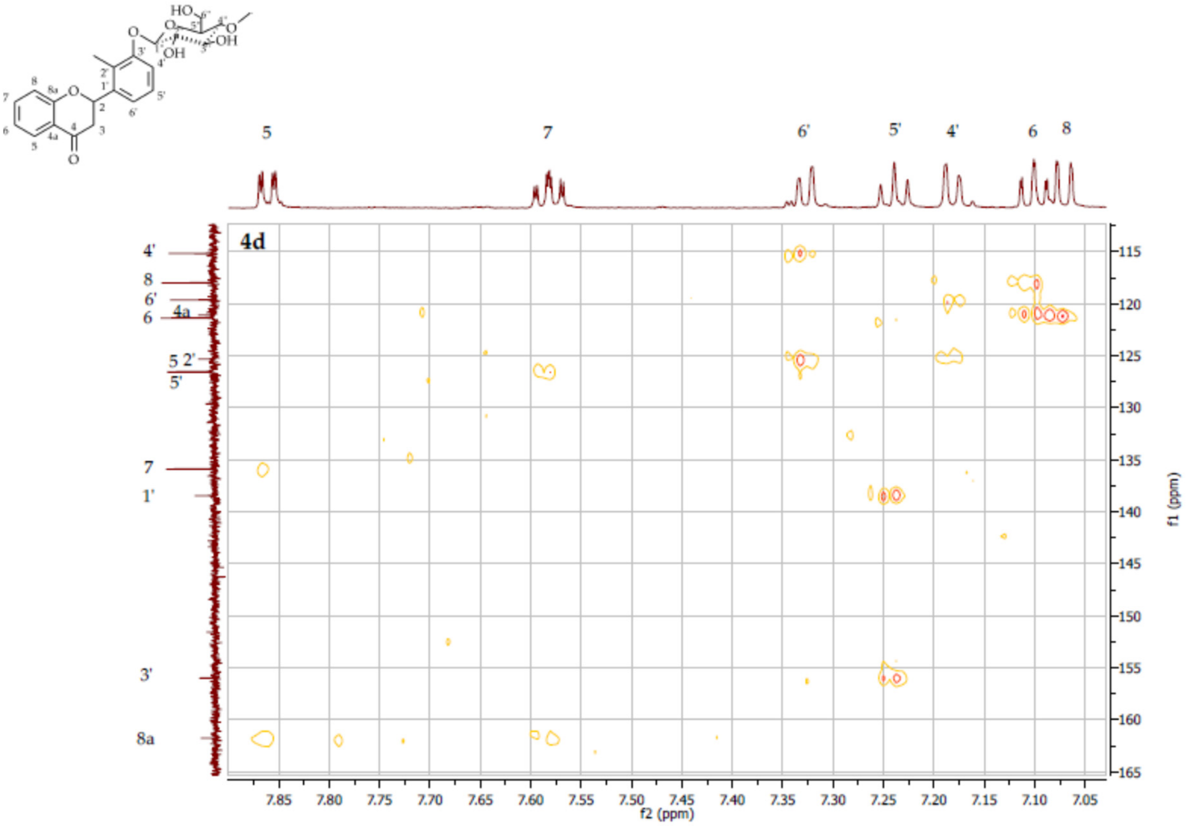

Figure S86. HMBC contour map – ^{1}H x ^{13}C expansion of 2′-methylflavanone 3′-*O*-*β*-D-(4′′-*O*-methyl)-glucopyranoside (**4d**)

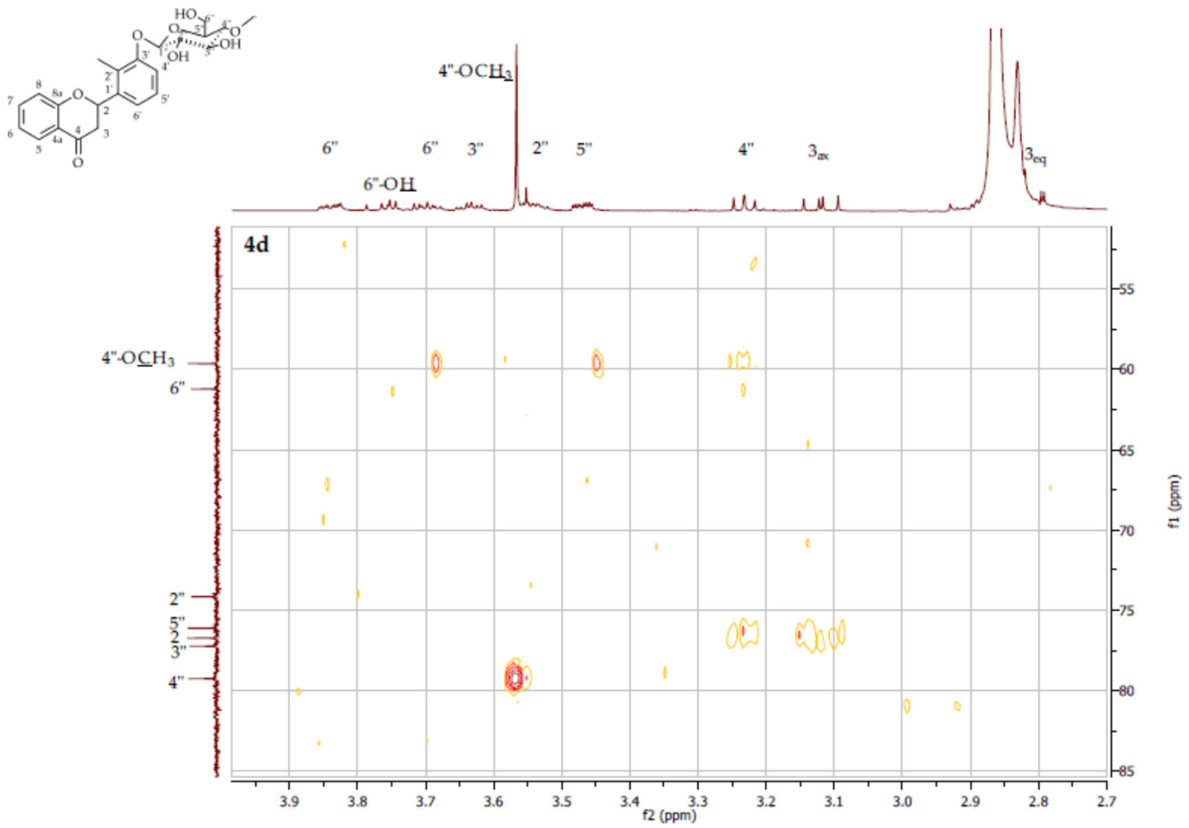

Figure S87. HMBC contour map – $^{1}H \times ^{13}C$ expansion of 2′-methylflavanone 3′-*O*-*β*-D-(4′′-*O*-methyl)-glucopyranoside (**4d**)

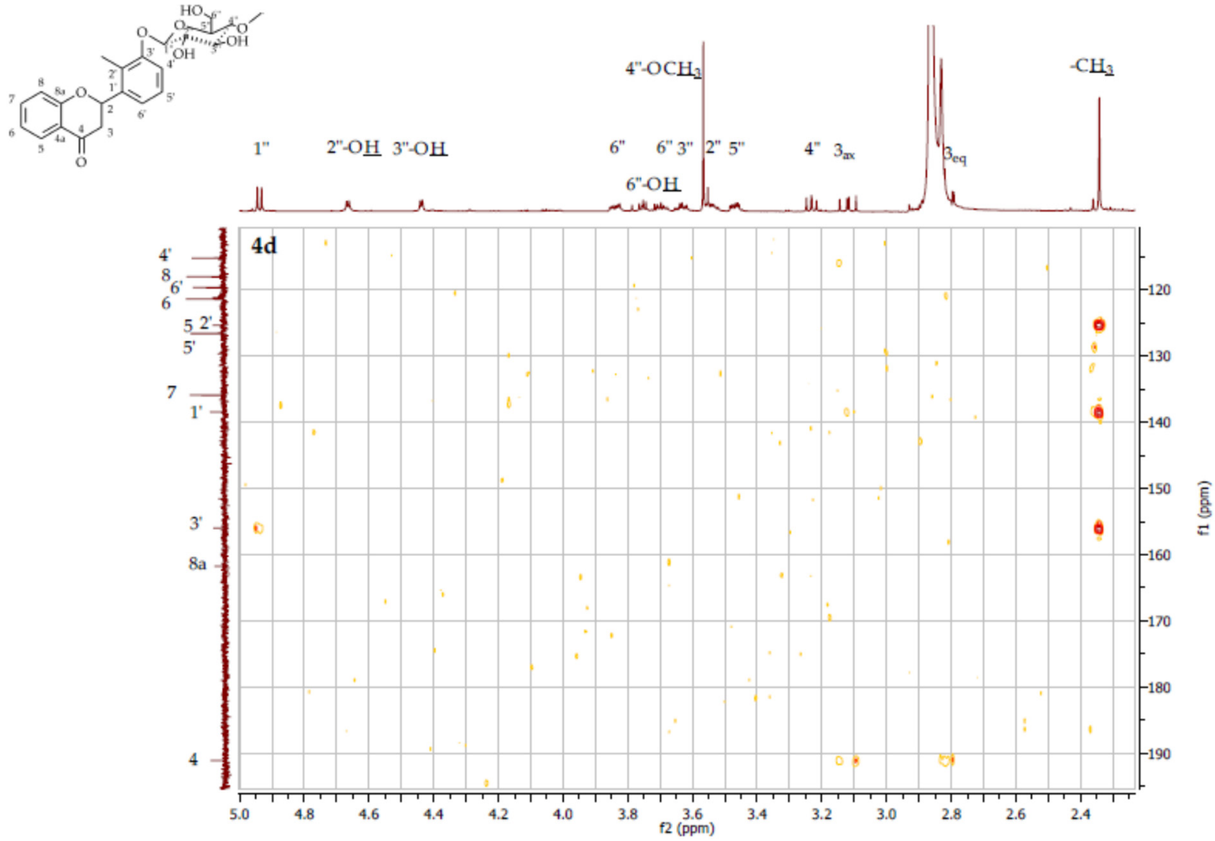

Figure S88. HMBC contour map – $^{1}H \times ^{13}C$ expansion of 2′-methylflavanone 3′-*O*-*β*-D-(4′′-*O*-methyl)-glucopyranoside (**4d**)

Molecular formula: $C_{15}H_{20}O_8$
Formula weight: 328.12
Ionization mode: negative
Precursor: $[M - H]^-$ 327.40
327.4000>151.1000 CE: 15.0
327.4000>150.1000 CE: 27.0
327.4000>108.0500 CE: 33.0

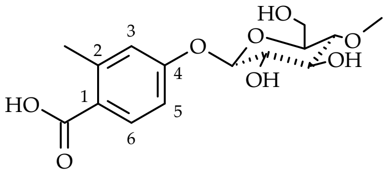

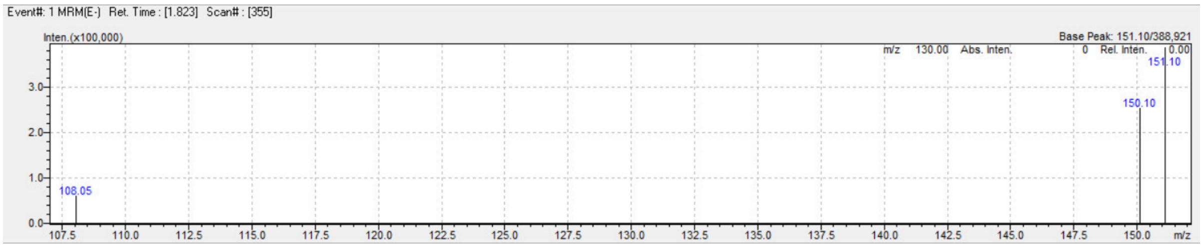

Figure S89. MS analysis of 2-methylbenzoic acid 4-*O*-*β*-D-(4′-*O*-methyl)-glucopyranoside (**4e**)

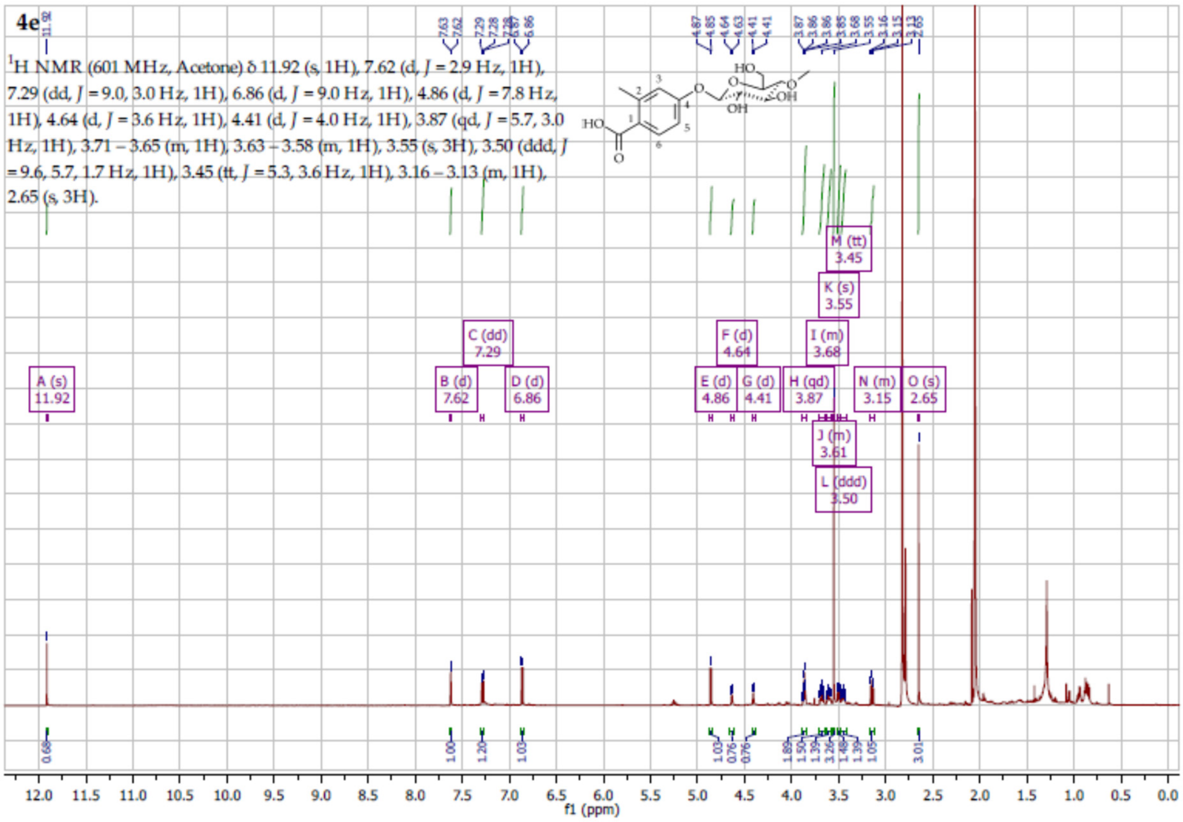

Figure S90. ^{1}H NMR spectrum (*δ*, acetone-d6, 600 MHz) of 2-methylbenzoic acid 4-*O*-*β*-D-(4′-*O*-methyl)-glucopyranoside (**4e**)

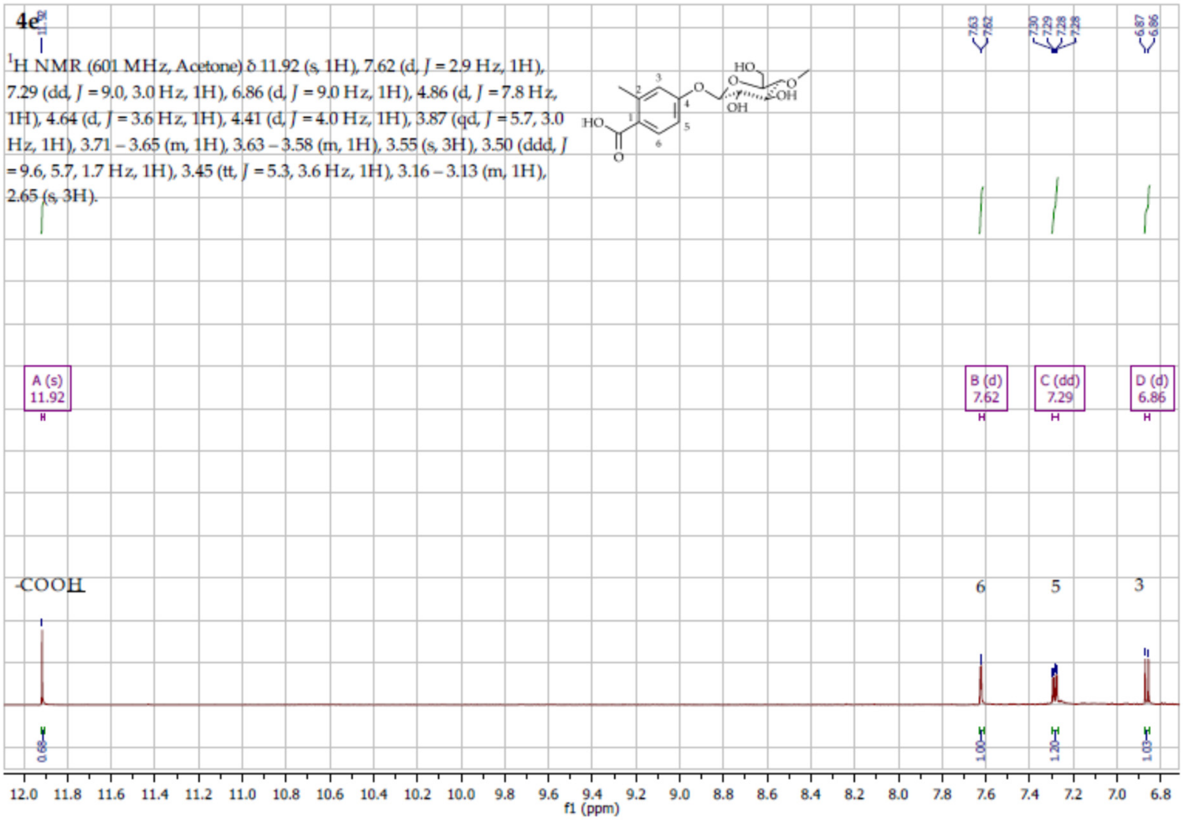

Figure S91. ¹H NMR spectrum expansion (*δ,* acetone-d6, 600 MHz) of 2-methylbenzoic acid 4-*O*-*β*-D-(4′-*O*-methyl)-glucopyranoside (**4e**)

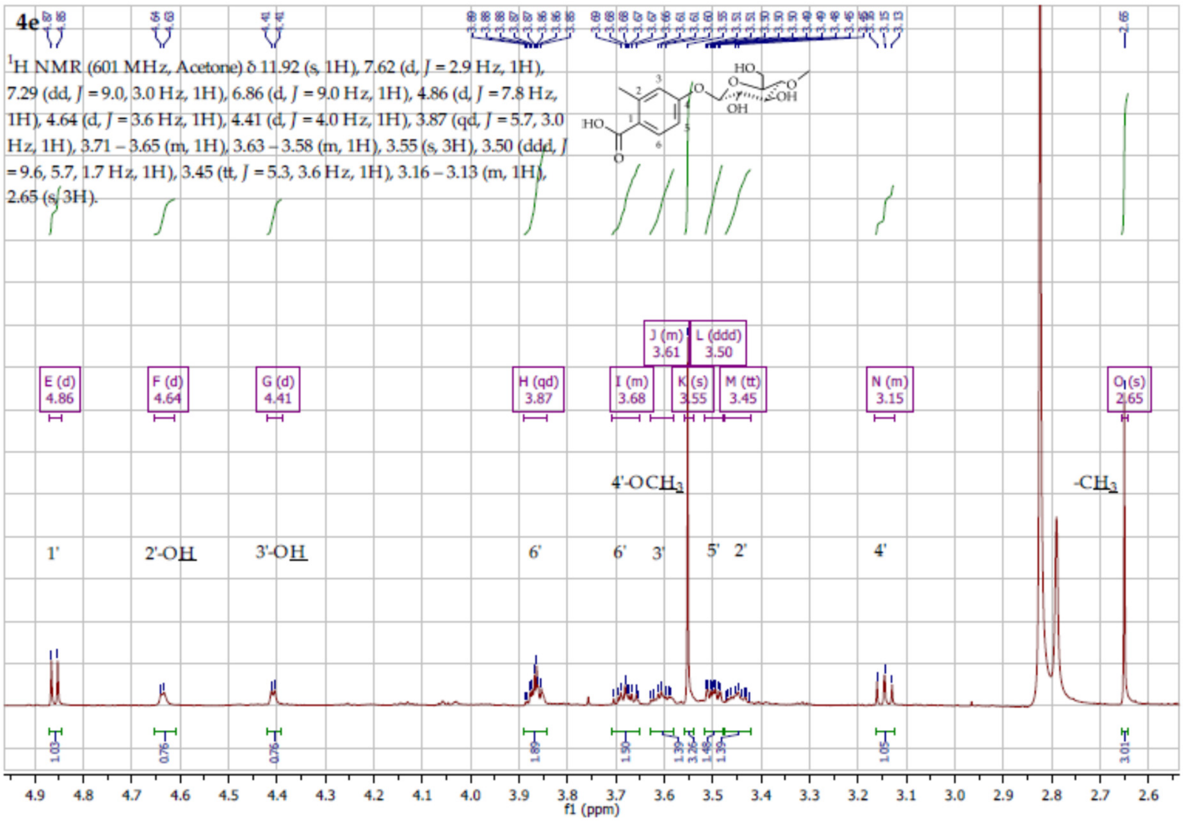

Figure S92. ¹H NMR spectrum expansion (*δ,* acetone-d6, 600 MHz) of 2-methylbenzoic acid 4-*O*-*β*-D-(4′-*O*-methyl)-glucopyranoside (**4e**)

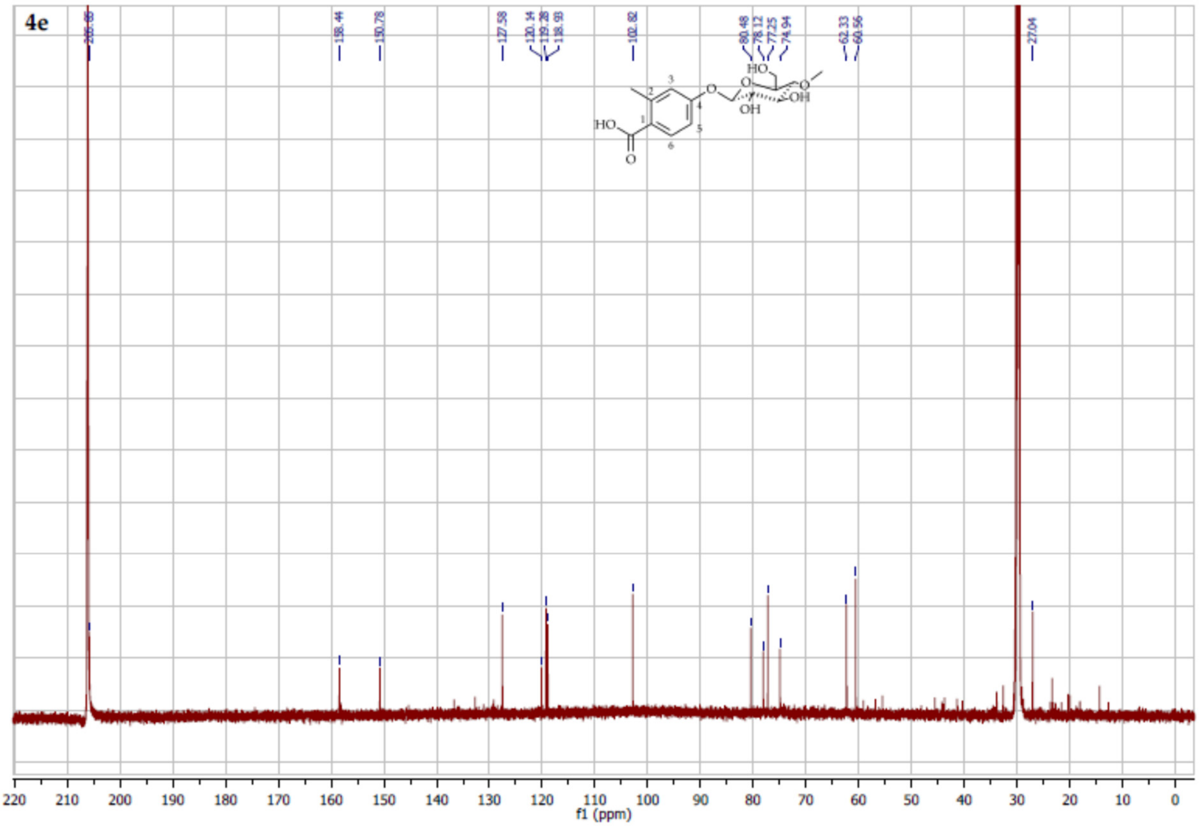

Figure S93. ^{13}C NMR spectrum (δ, acetone-d6, 151 MHz) of 2-methylbenzoic acid 4-*O*-*β*-D-(4′-*O*-methyl)-glucopyranoside (**4e**)

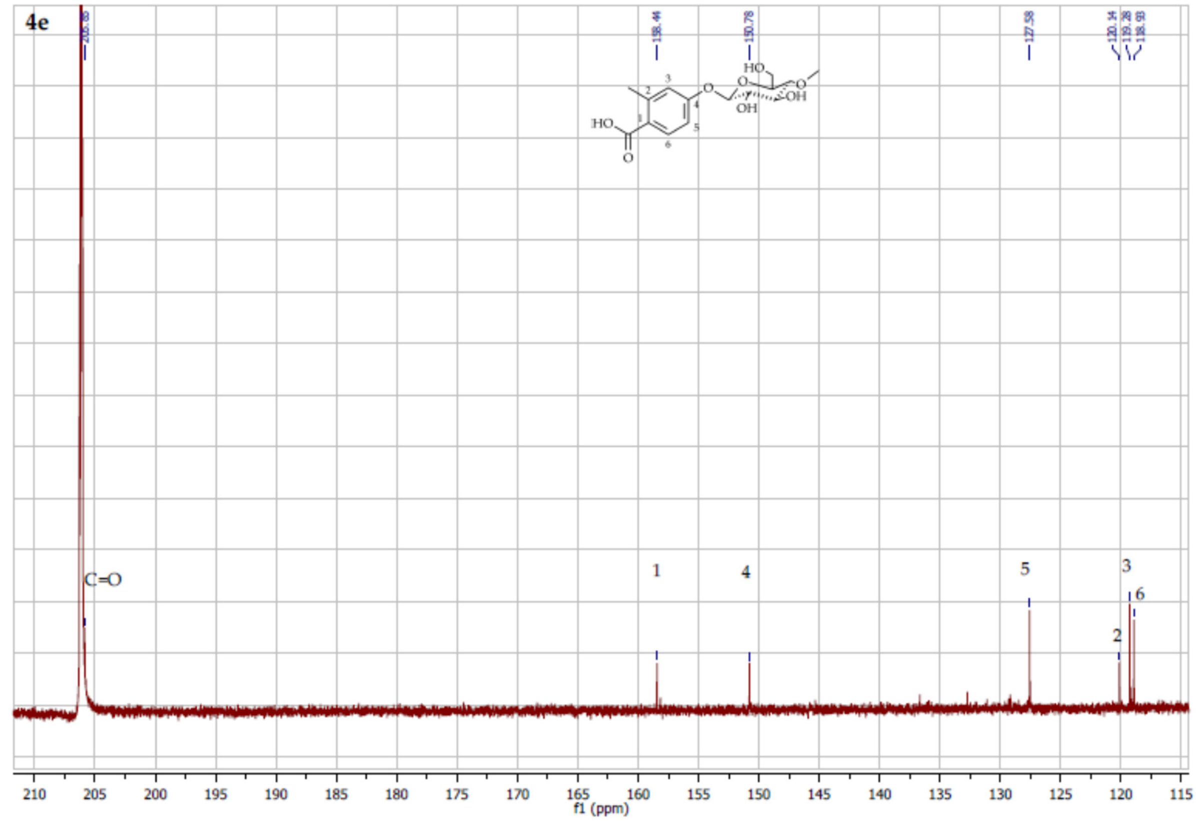

Figure S94. ^{13}C NMR spectrum expansion (δ, acetone-d6, 151 MHz) of 2-methylbenzoic acid 4-*O*-*β*-D-(4′-*O*-methyl)-glucopyranoside (**4e**)

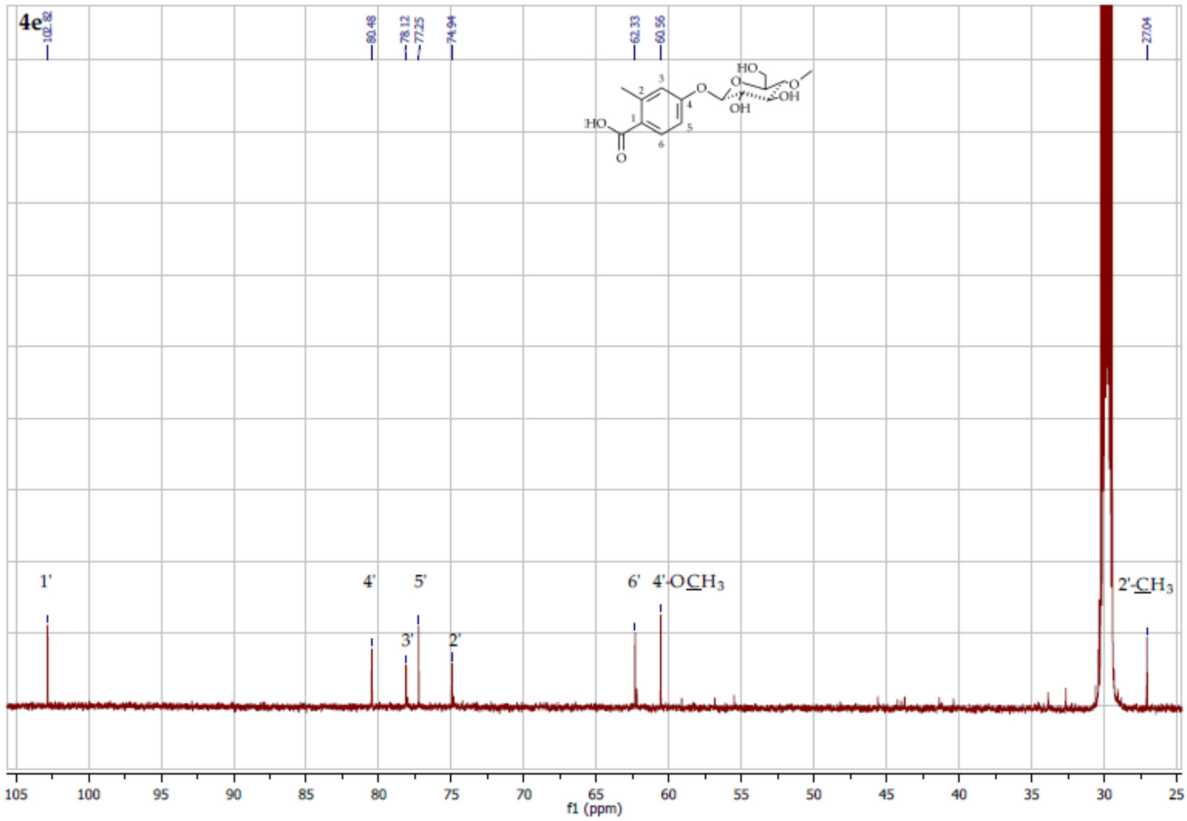

Figure S95. ^{13}C NMR spectrum expansion (*δ*, acetone-d6, 151 MHz) of 2-methylbenzoic acid 4-*O*-*β*-D-(4′-*O*-methyl)-glucopyranoside (**4e**)

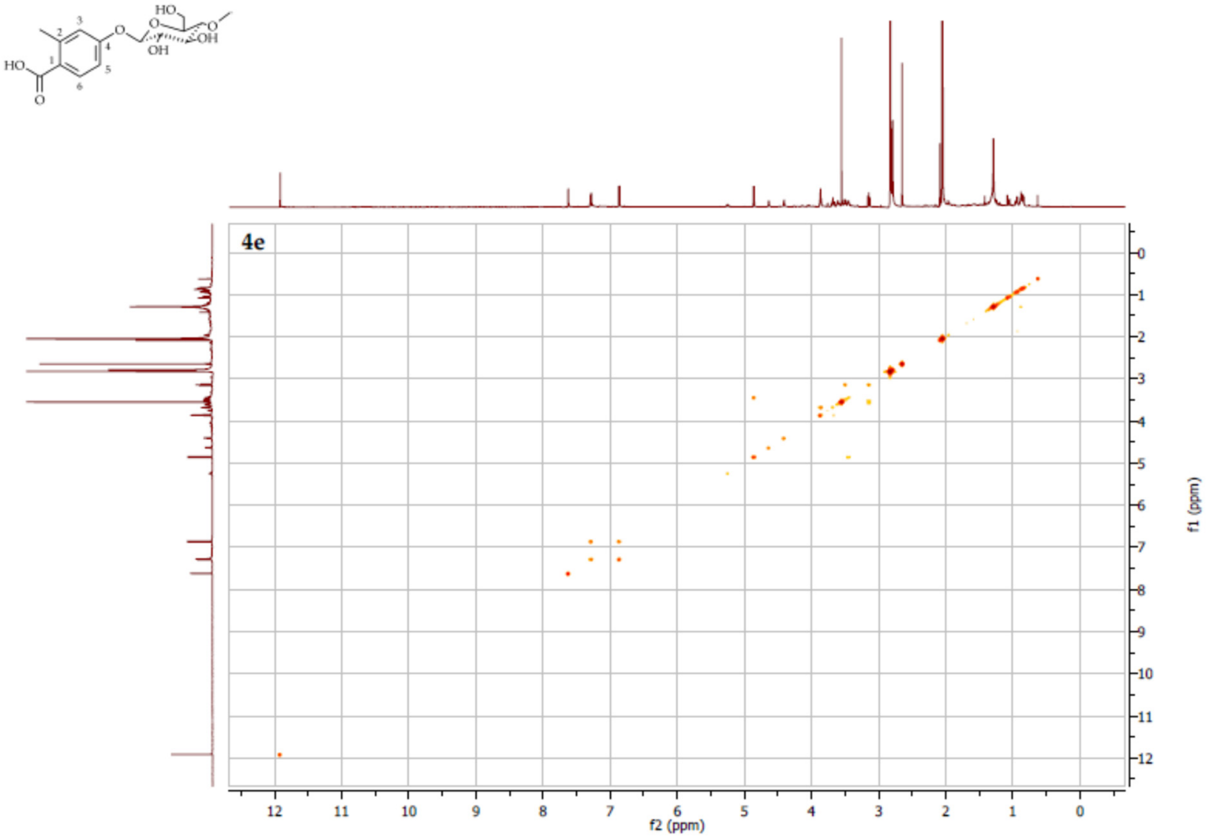

Figure S96. COSY contour map – ^1H x ^1H of 2-methylbenzoic acid 4-*O*-*β*-D-(4′-*O*-methyl)-glucopyranoside (**4e**)

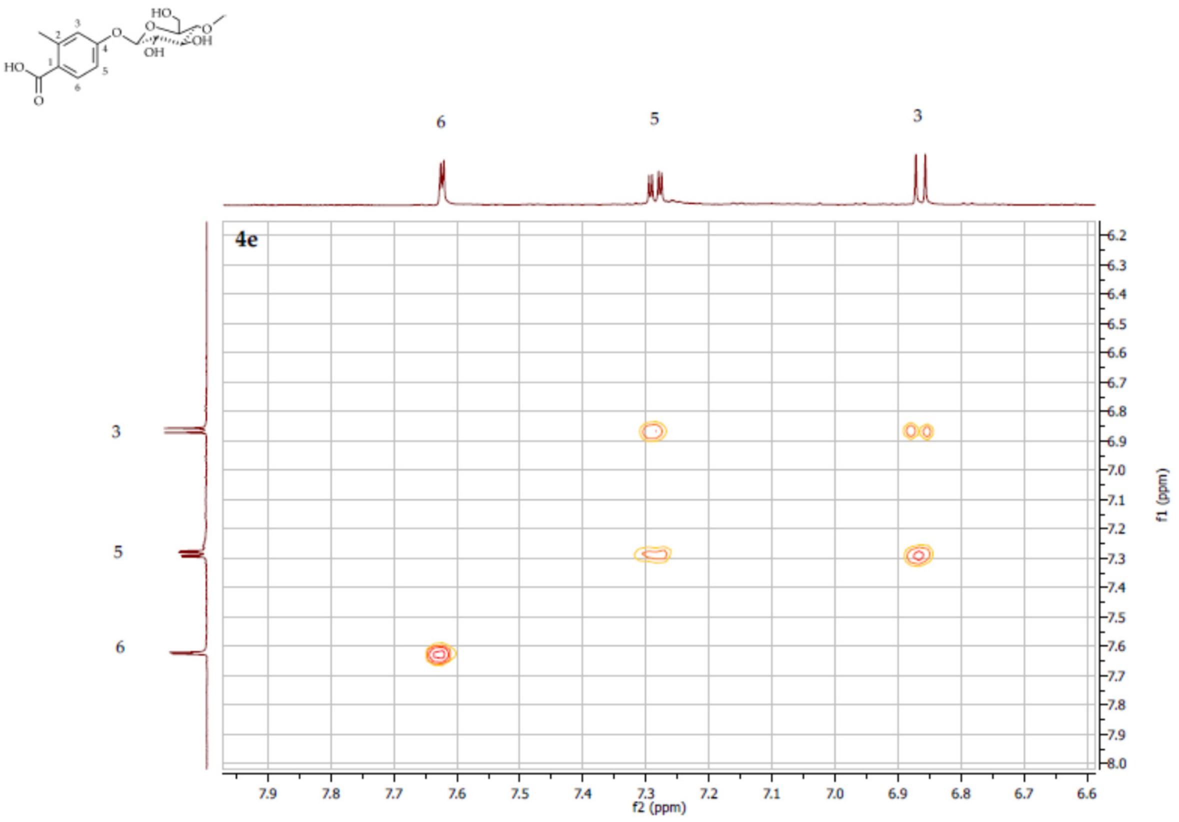

Figure S97. COSY contour map – ^{1}H x ^{1}H expansion of 2-methylbenzoic acid 4-*O*-*β*-D-(4′-*O*-methyl)-glucopyranoside (**4e**)

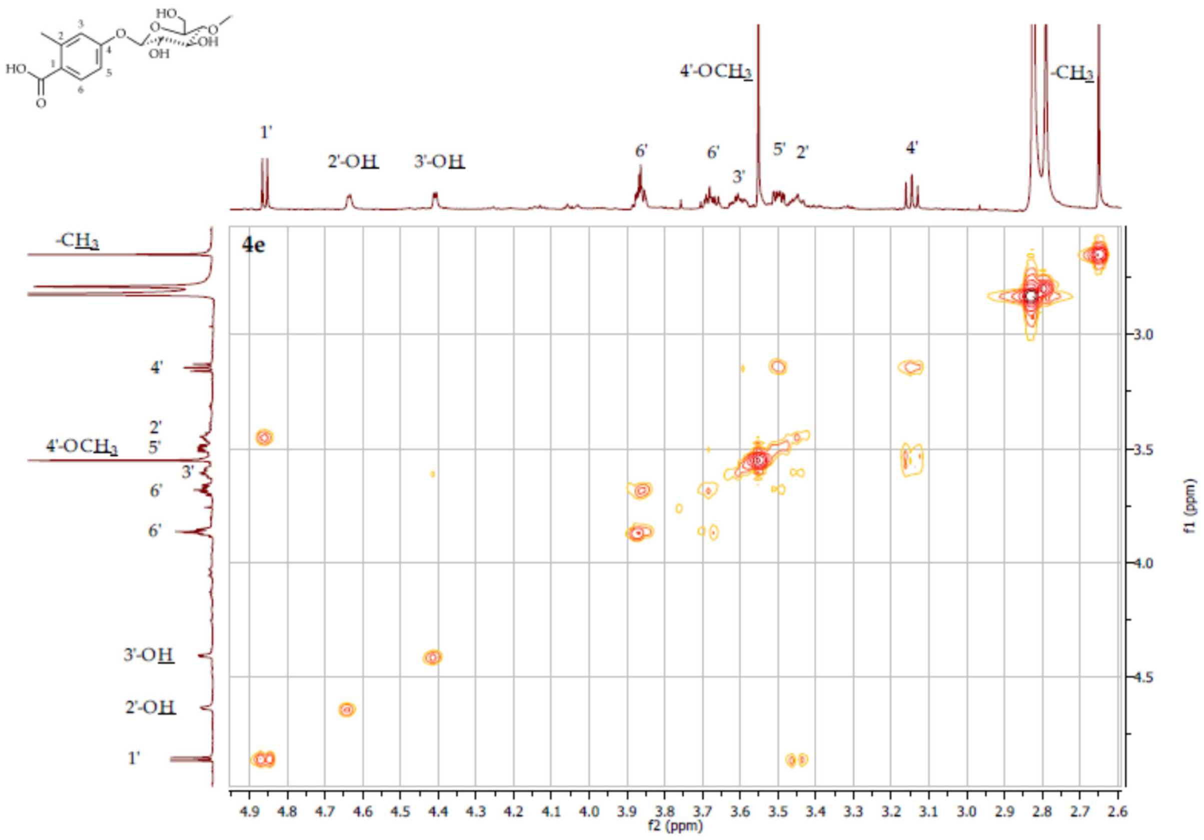

Figure S98. COSY contour map – ^{1}H x ^{1}H expansion of 2-methylbenzoic acid 4-*O*-*β*-D-(4′-*O*-methyl)-glucopyranoside (**4e**)

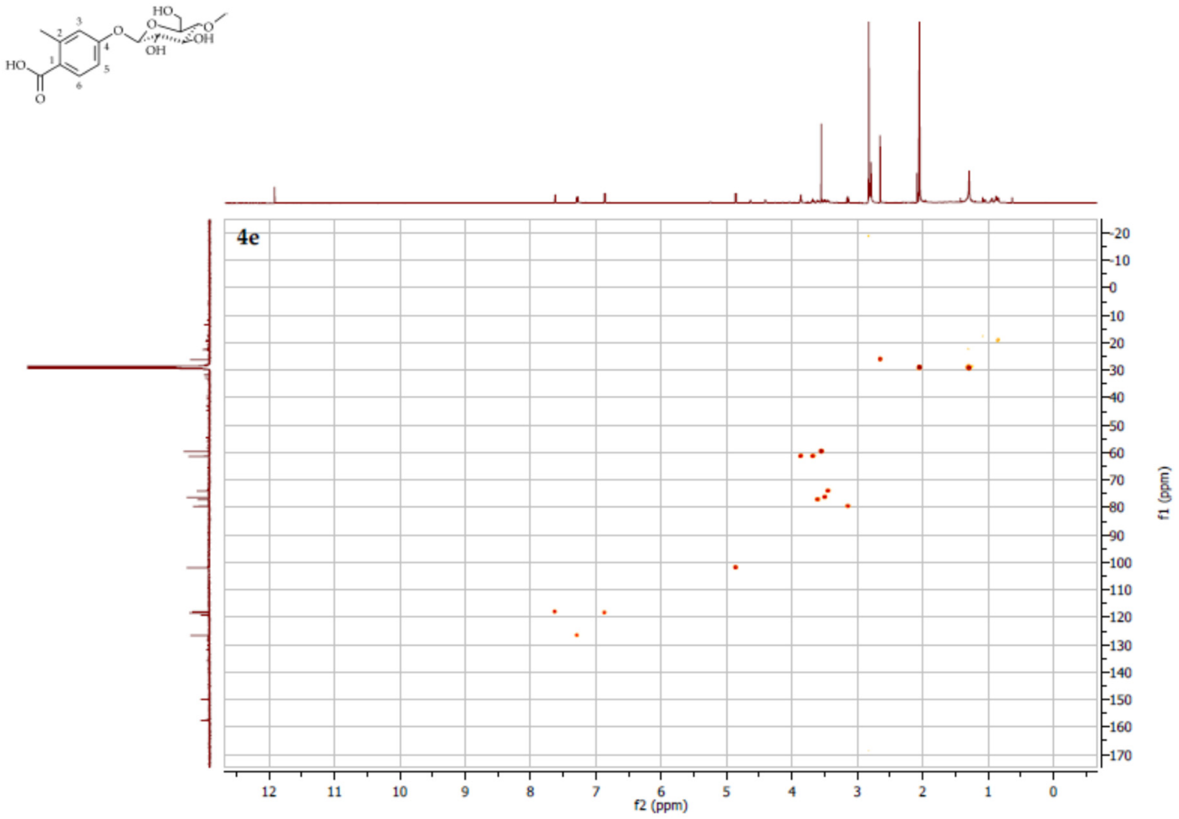

Figure S99. HSQC contour map – ^{1}H x ^{13}C 2-methylbenzoic acid 4-*O*-*β*-D-(4′-*O*-methyl)-glucopyranoside (**4e**)

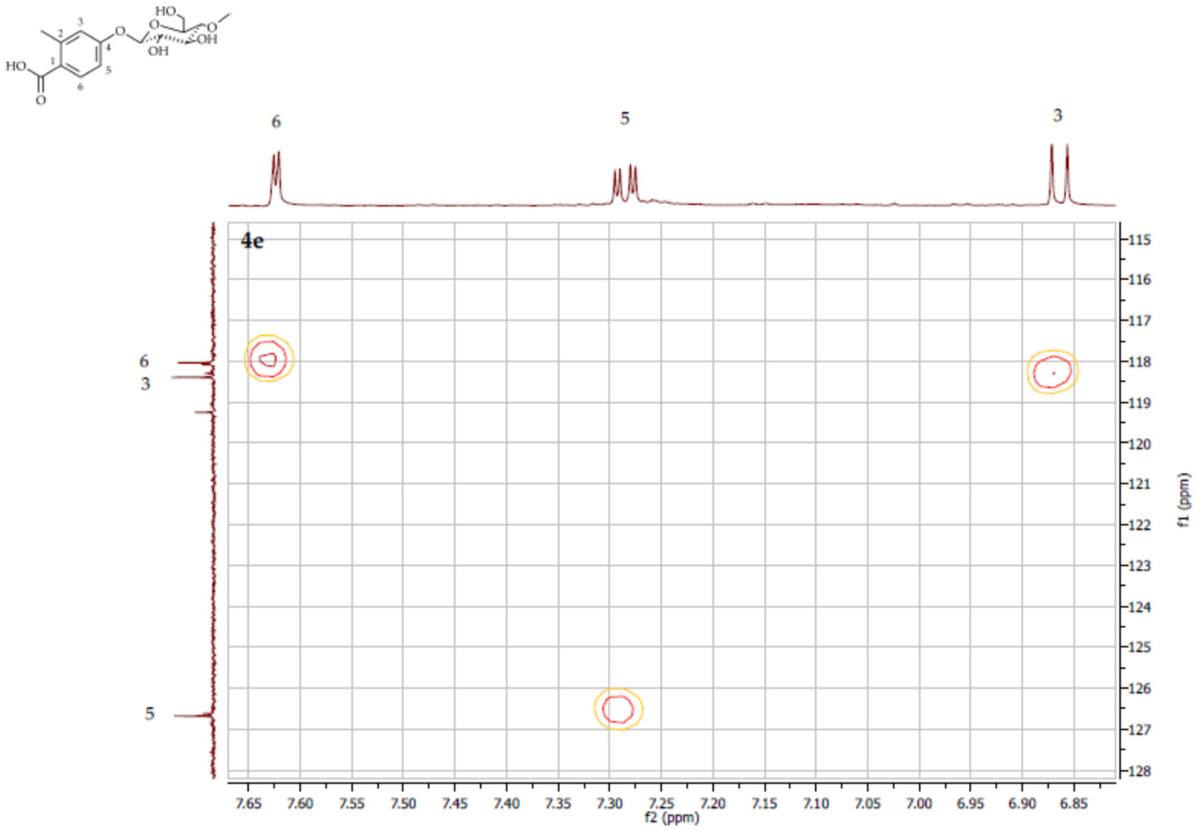

Figure S100. HSQC contour map – ^{1}H x ^{13}C expansion of 2-methylbenzoic acid 4-*O*-*β*-D-(4′-*O*-methyl)-glucopyranoside (**4e**)

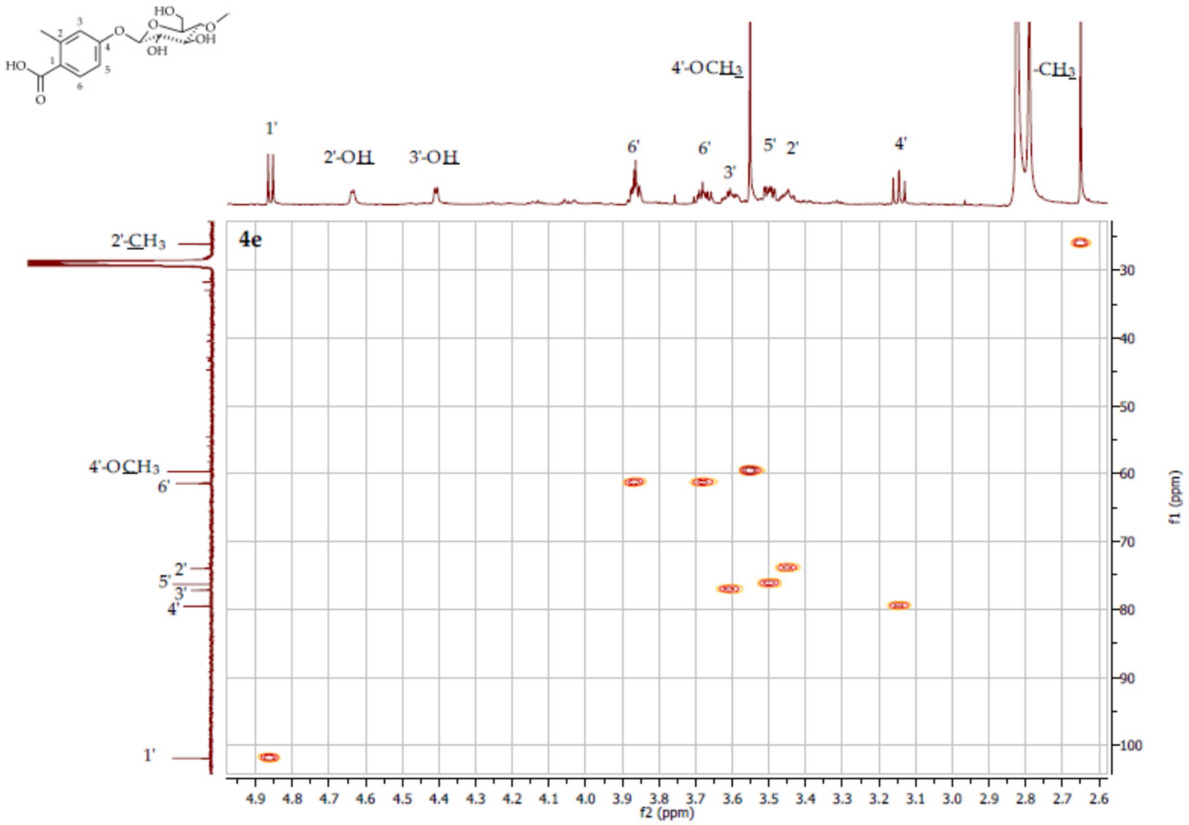

Figure S101. HSQC contour map – ^{1}H x ^{13}C expansion of 2-methylbenzoic acid 4-*O*-*β*-D-(4′-*O*-methyl)-glucopyranoside (**4e**)

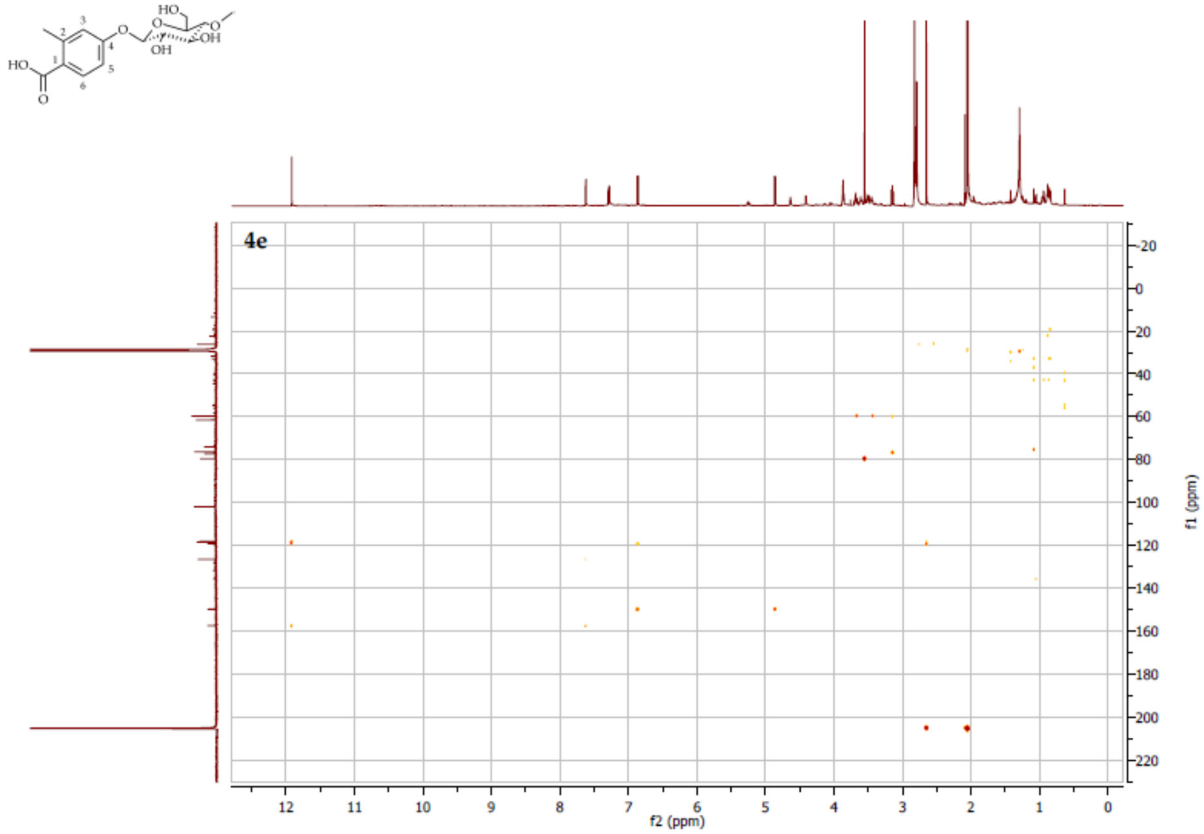

Figure S102. HMBC contour map – ^{1}H x ^{13}C of 2-methylbenzoic acid 4-*O*-*β*-D-(4′-*O*-methyl)-glucopyranoside (**4e**)

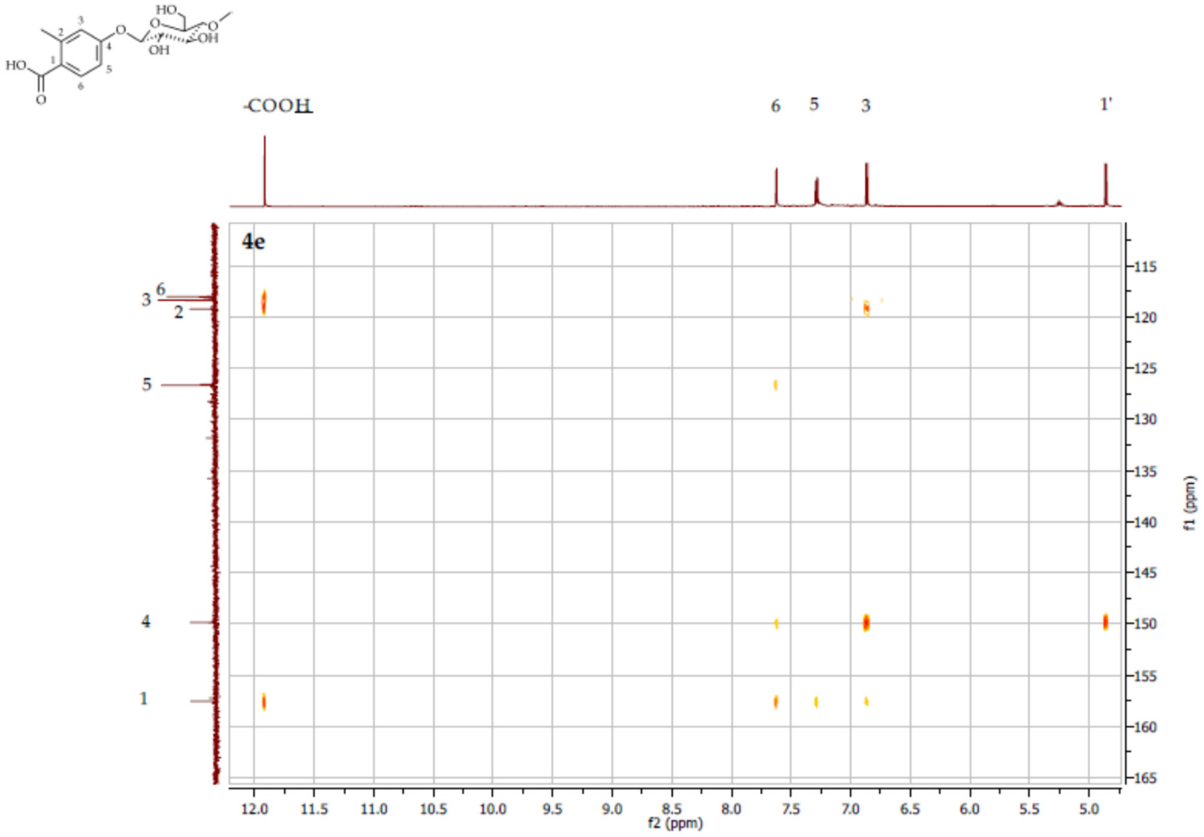

Figure S103. HMBC contour map – $^{1}H \times ^{13}C$ expansion of 2-methylbenzoic acid 4-*O*-*β*-D-(4′-*O*-methyl)-glucopyranoside (**4e**)

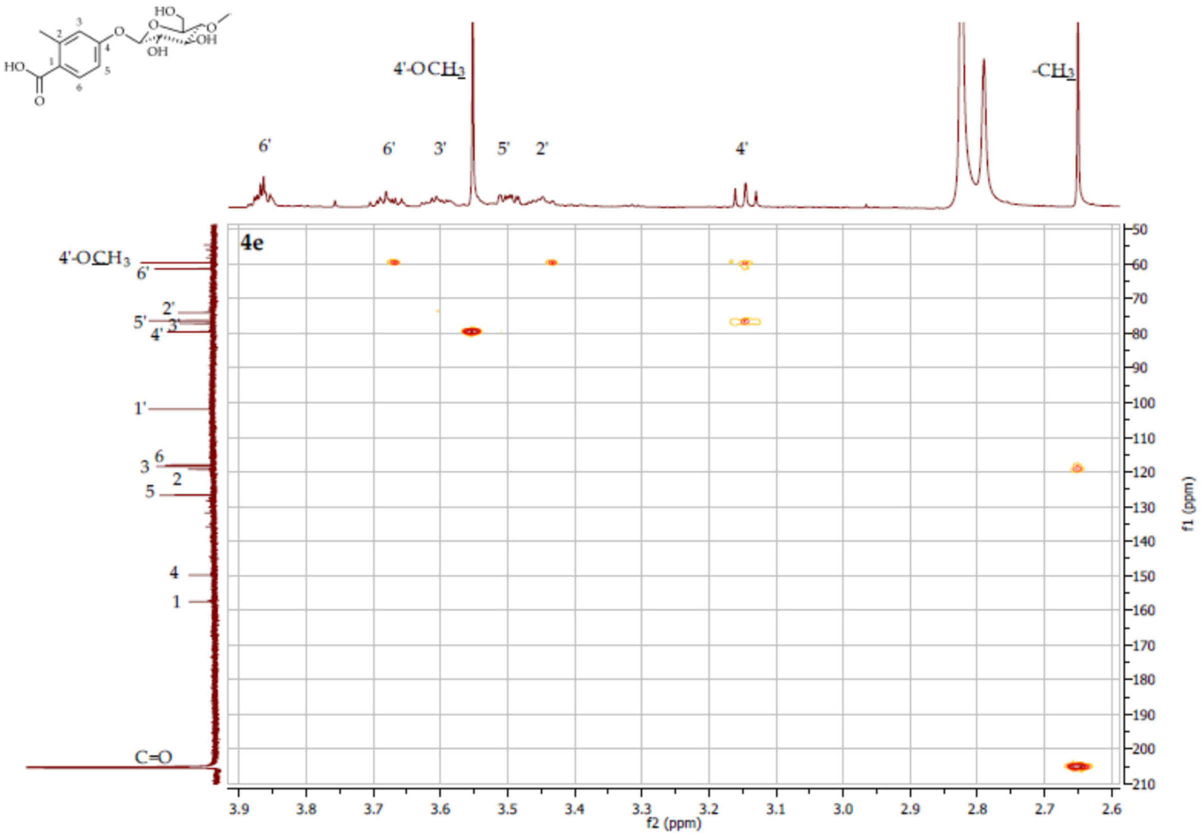

Figure S104. HMBC contour map – $^{1}H \times ^{13}C$ expansion of 2-methylbenzoic acid 4-*O*-*β*-D-(4′-*O*-methyl)-glucopyranoside (**4e**)

Molecular formula: $C_{16}H_{12}O_2$

Formula weight: 236.08

Ionization mode: positive

Precursor: $[M + H]^+$ 237.10

237.1000>65.0500 CE: -51.0

237.1000>121.0500 CE: -31.0

237.1000>115.1000 CE: -39.0

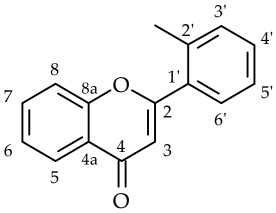

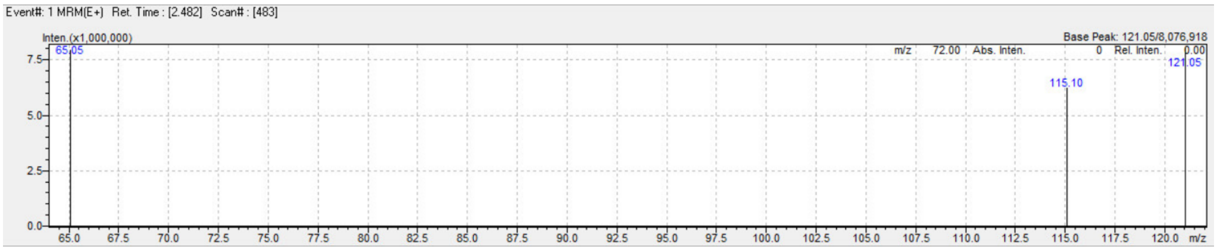

Figure S105. MS analysis of 2′-methylflavone (**5**)

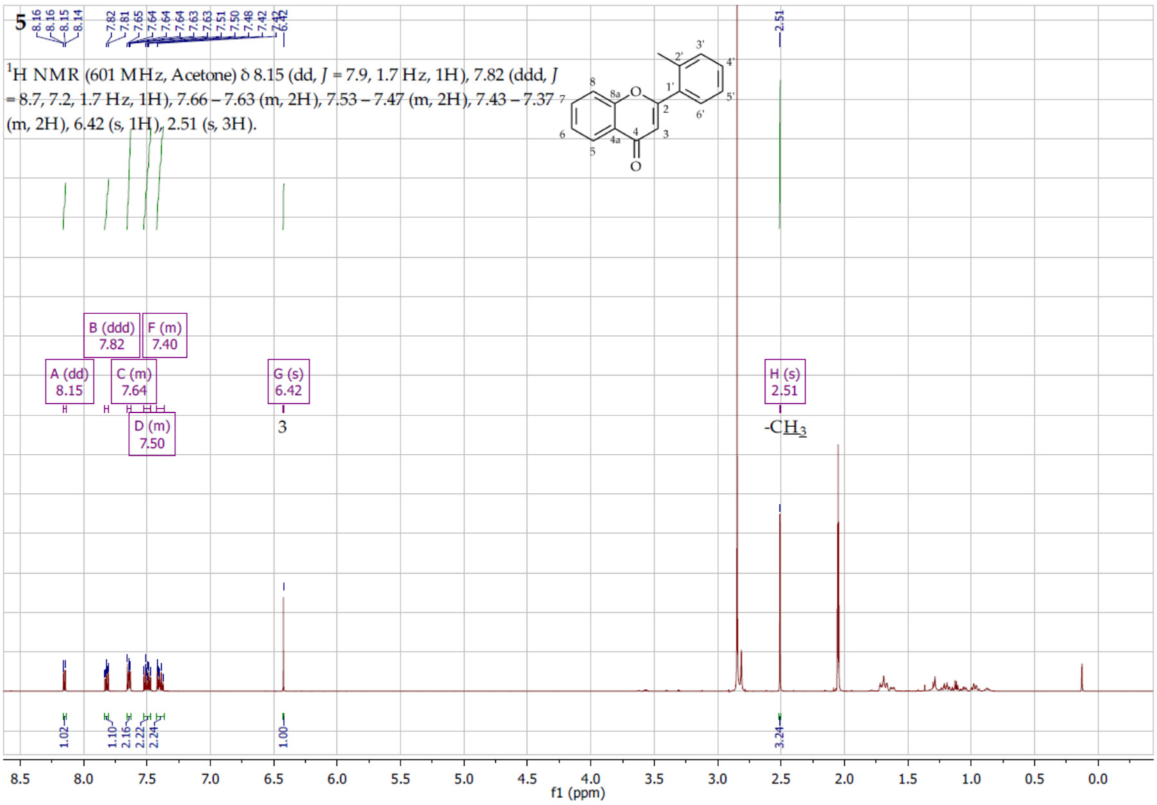

Figure S106. ¹H NMR spectrum (δ, acetone-d6, 600 MHz) of 2′-methylflavone (**5**)

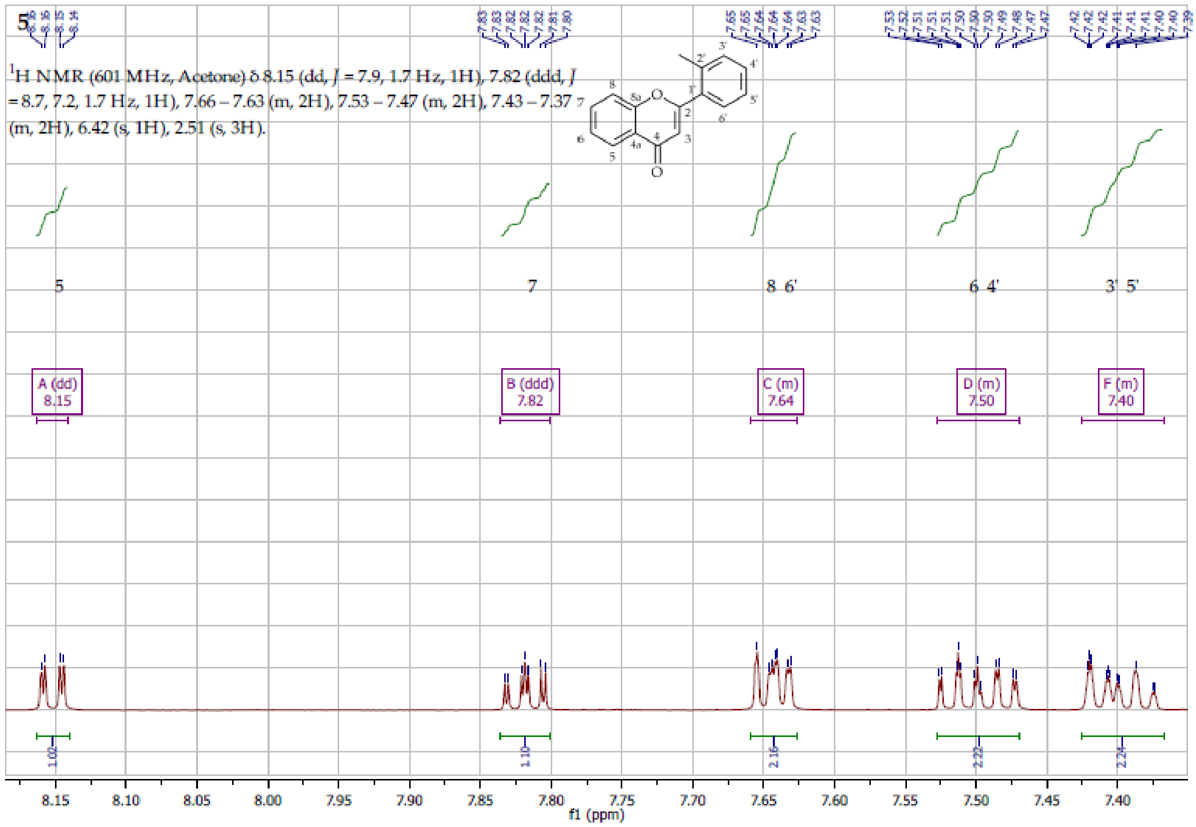

Figure S107. ^{1}H NMR spectrum expansion (δ, acetone-d6, 600 MHz) 2′-methylflavone (**5**)

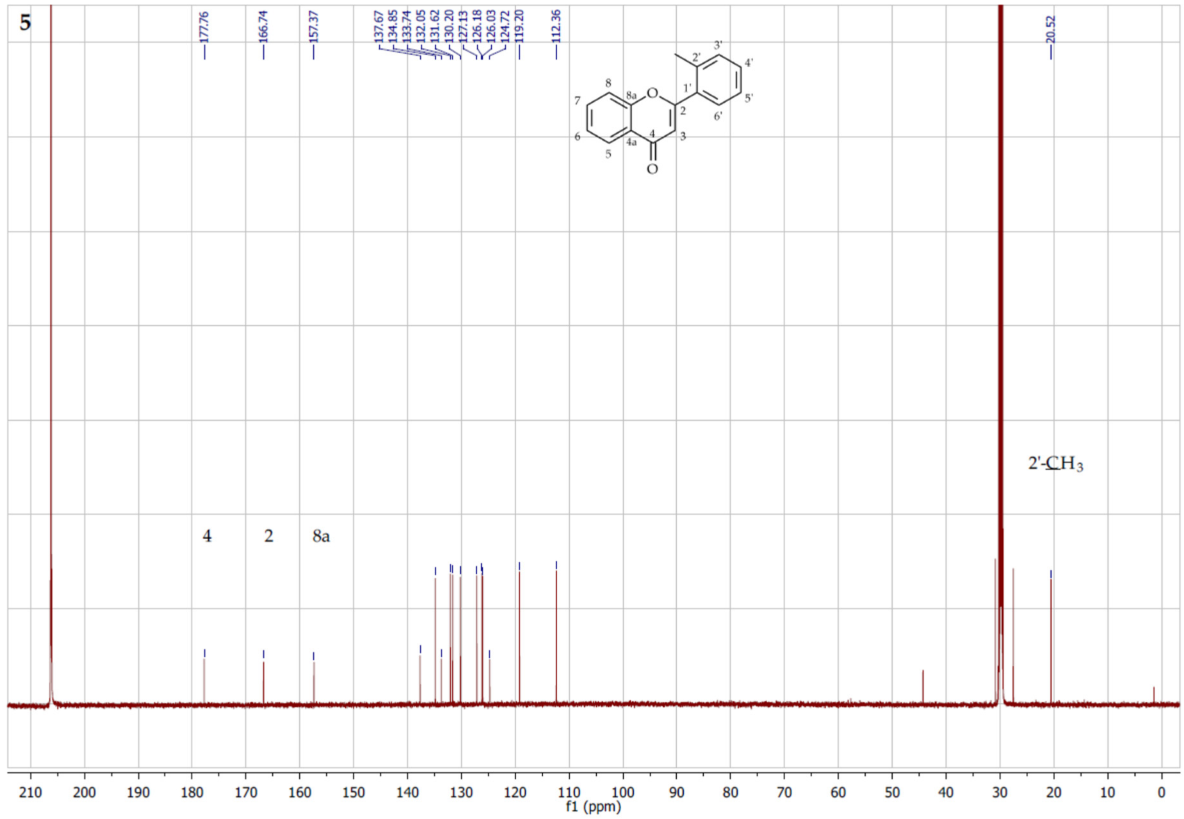

Figure S108. ^{13}C NMR spectrum (δ, acetone-d6, 151 MHz) of 2′-methylflavone (**5**)

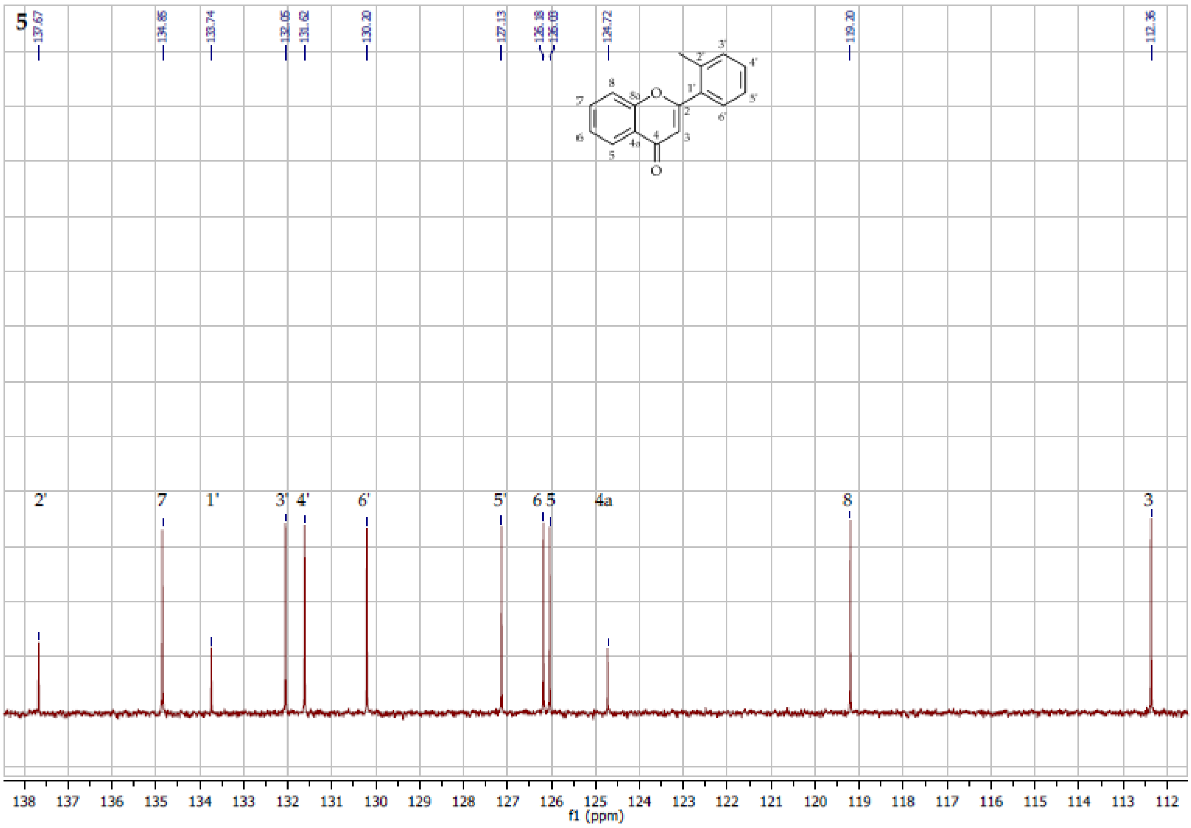

Figure S109. ^{13}C NMR spectrum expansion (δ, acetone-d6, 151 MHz) of 2′-methylflavone (**5**)

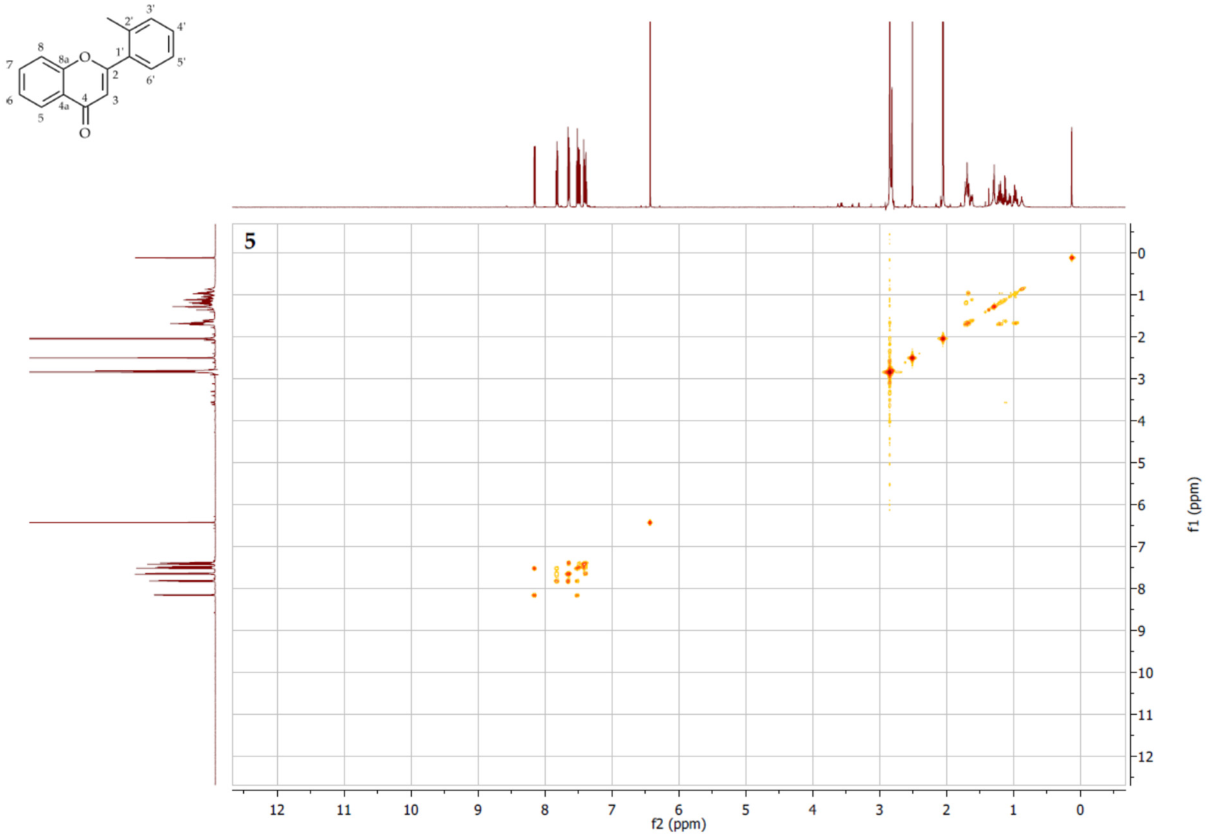

Figure S110. COSY contour map – ^{1}H x ^{1}H of 2′-methylflavone (**5**)

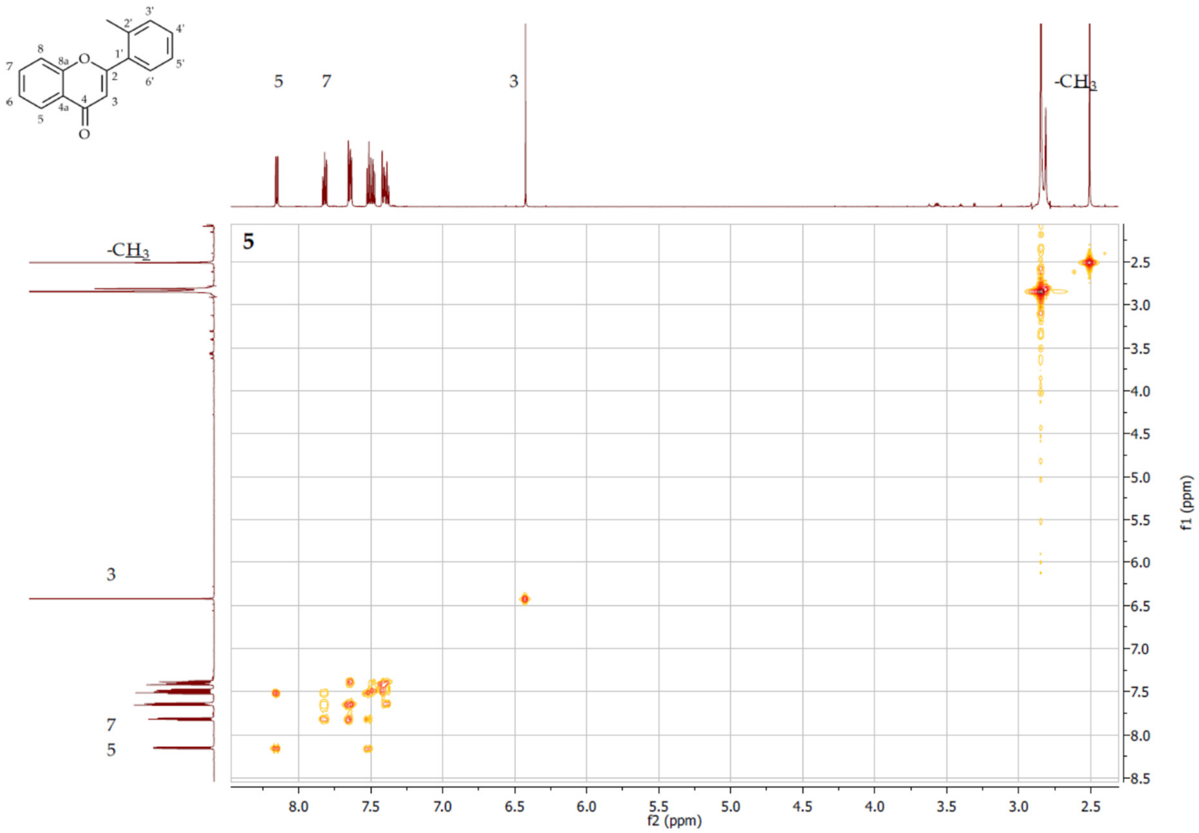

Figure S111. COSY contour map – ^{1}H x ^{1}H expansion of 2′-methylflavone (**5**)

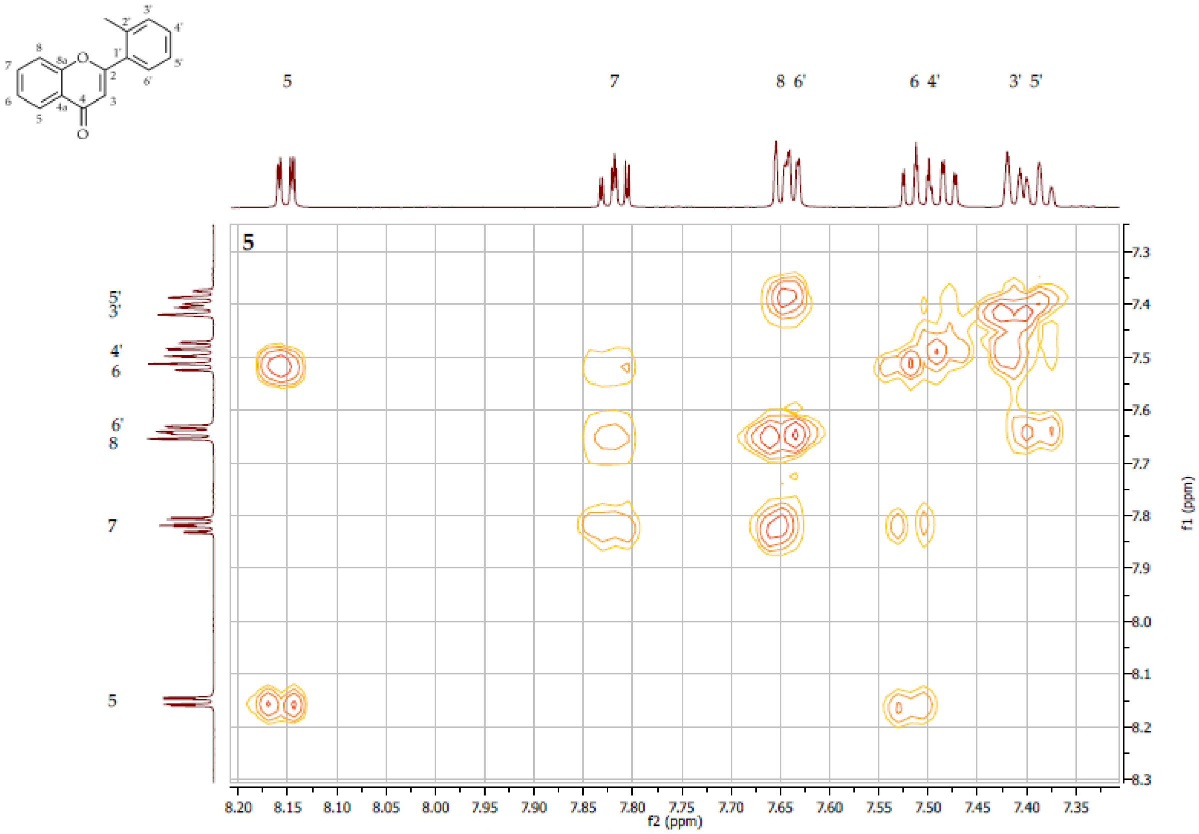

Figure S112. COSY contour map – ^{1}H x ^{1}H expansion of 2′-methylflavone (**5**)

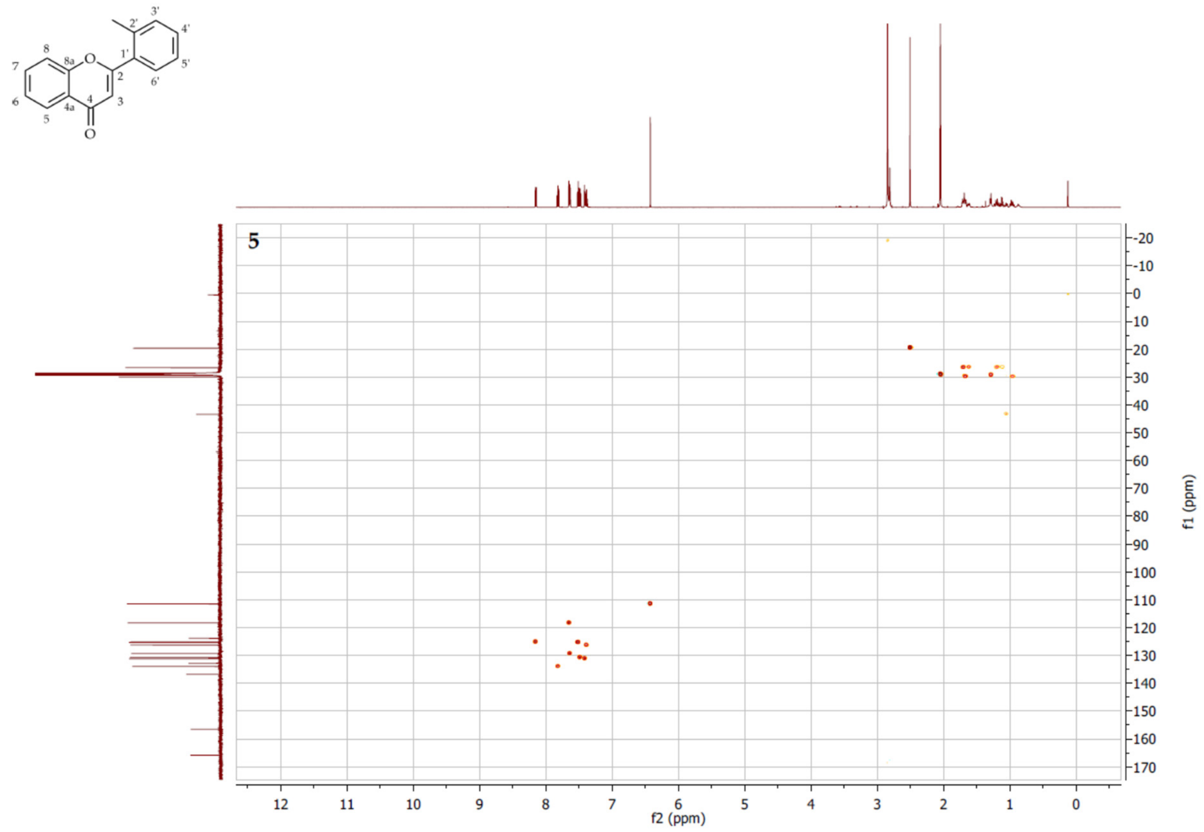

Figure S113. HSQC contour map – ^{1}H x ^{13}C of 2′-methylflavone (**5**)

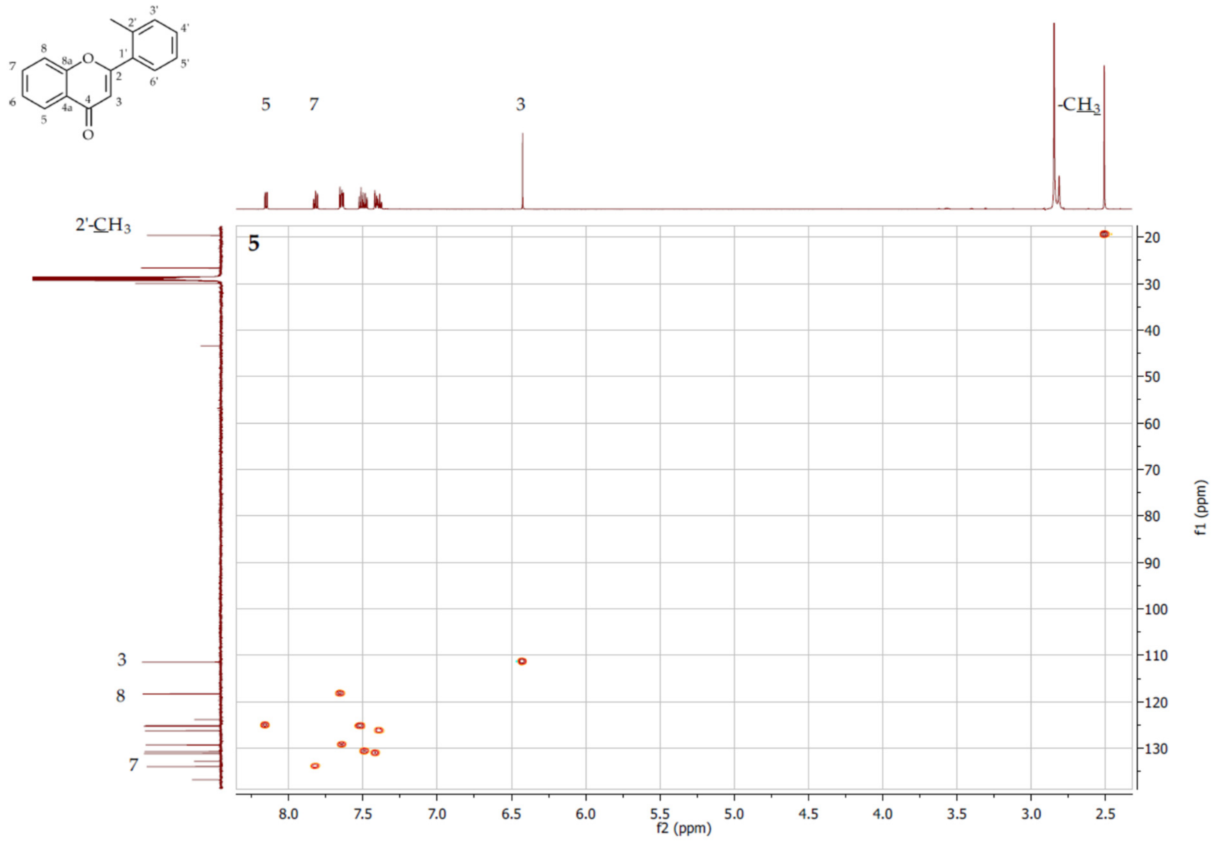

Figure S114. HSQC contour map – ^{1}H x ^{13}C expansion of 2′-methylflavone (**5**)

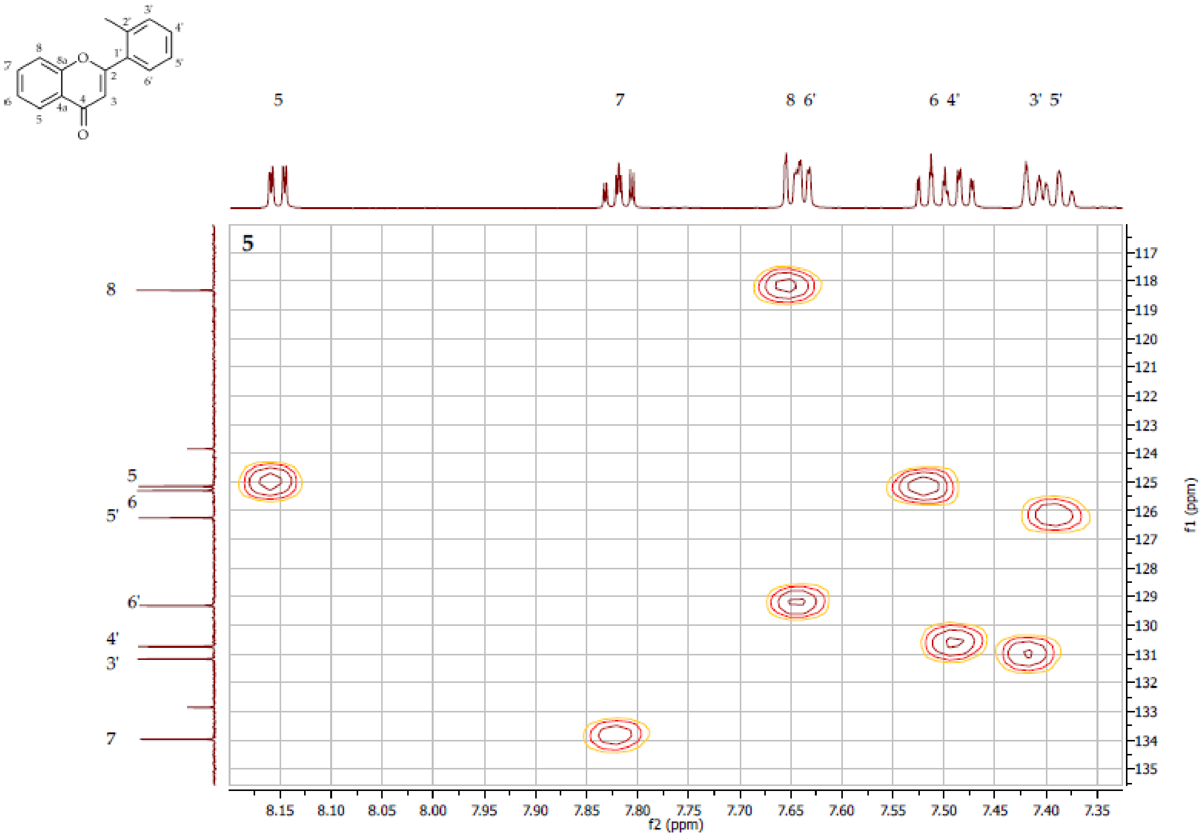

Figure S115. HSQC contour map – ¹H x ¹³C expansion of 2′-methylflavone (**5**)

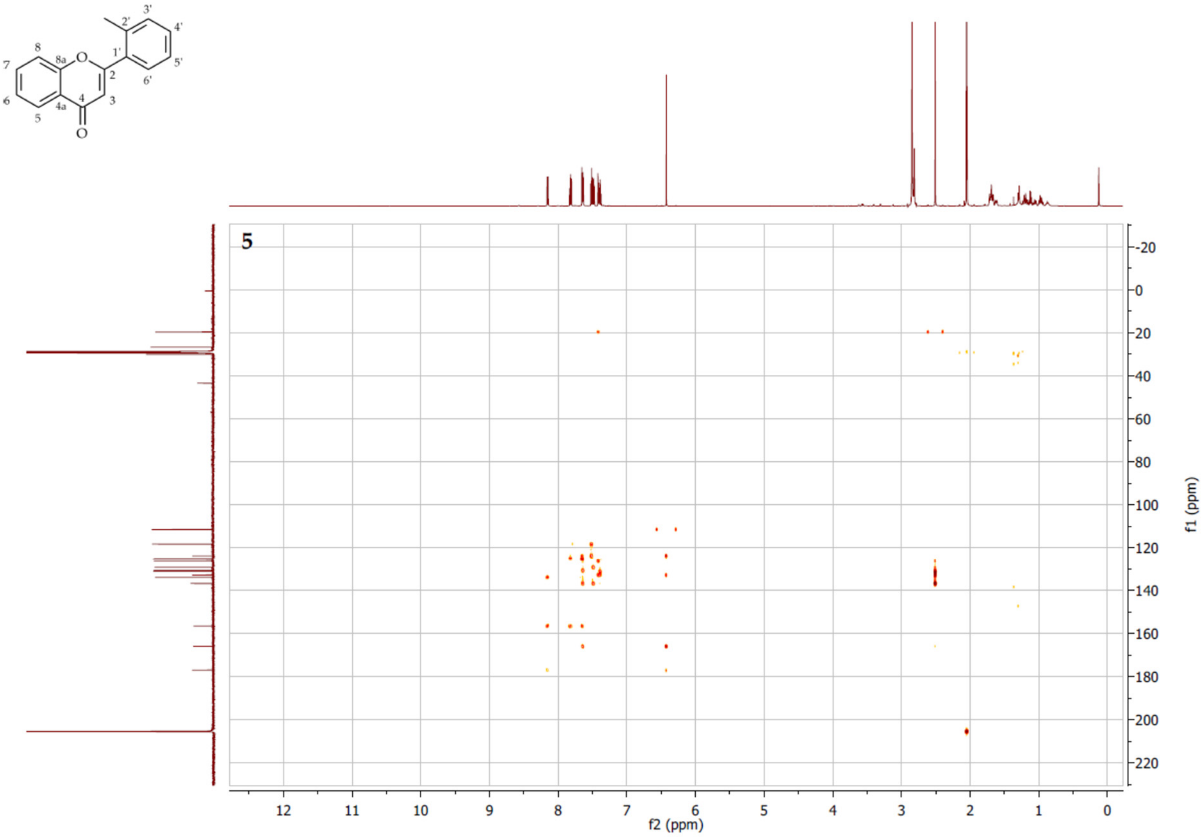

Figure S116. HMBC contour map – ¹H x ¹³C of 2′-methylflavone (**5**)

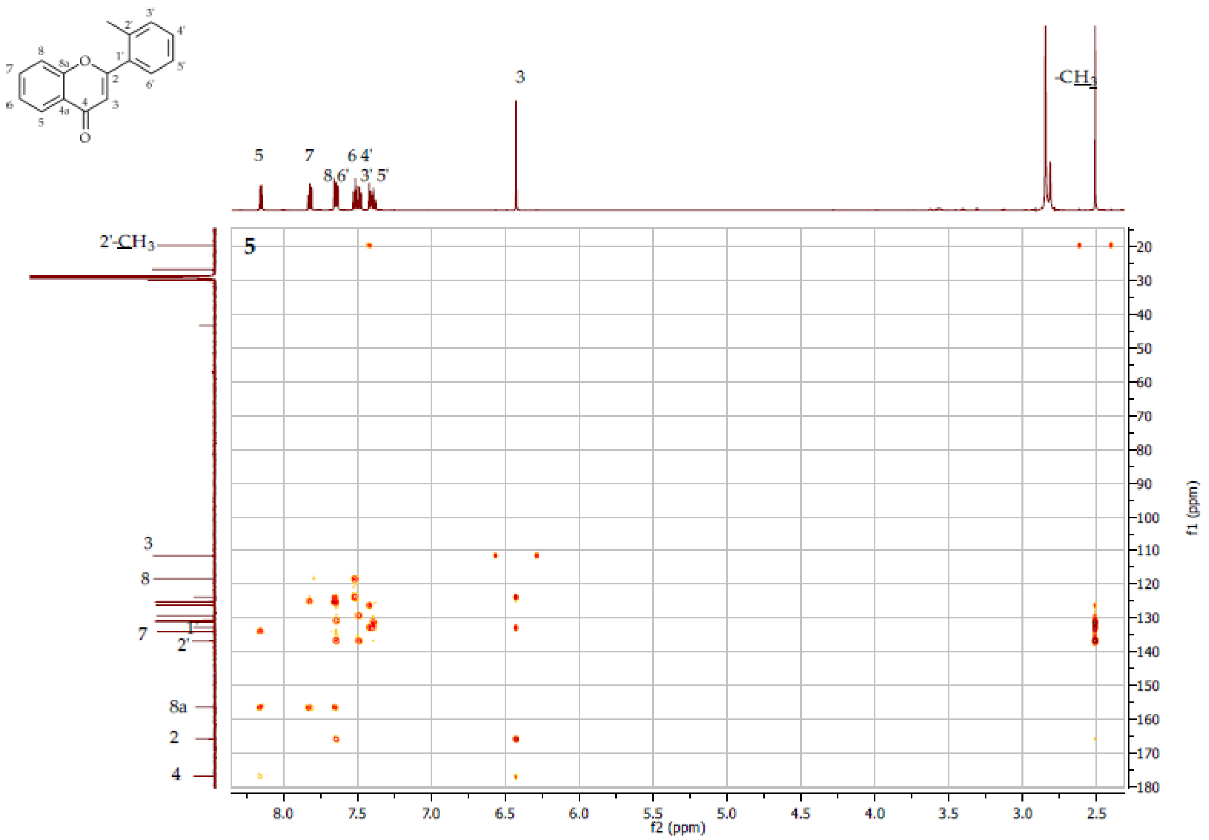

Figure S117. HMBC contour map – ^{1}H x ^{13}C expansion of 2′-methylflavone (**5**)

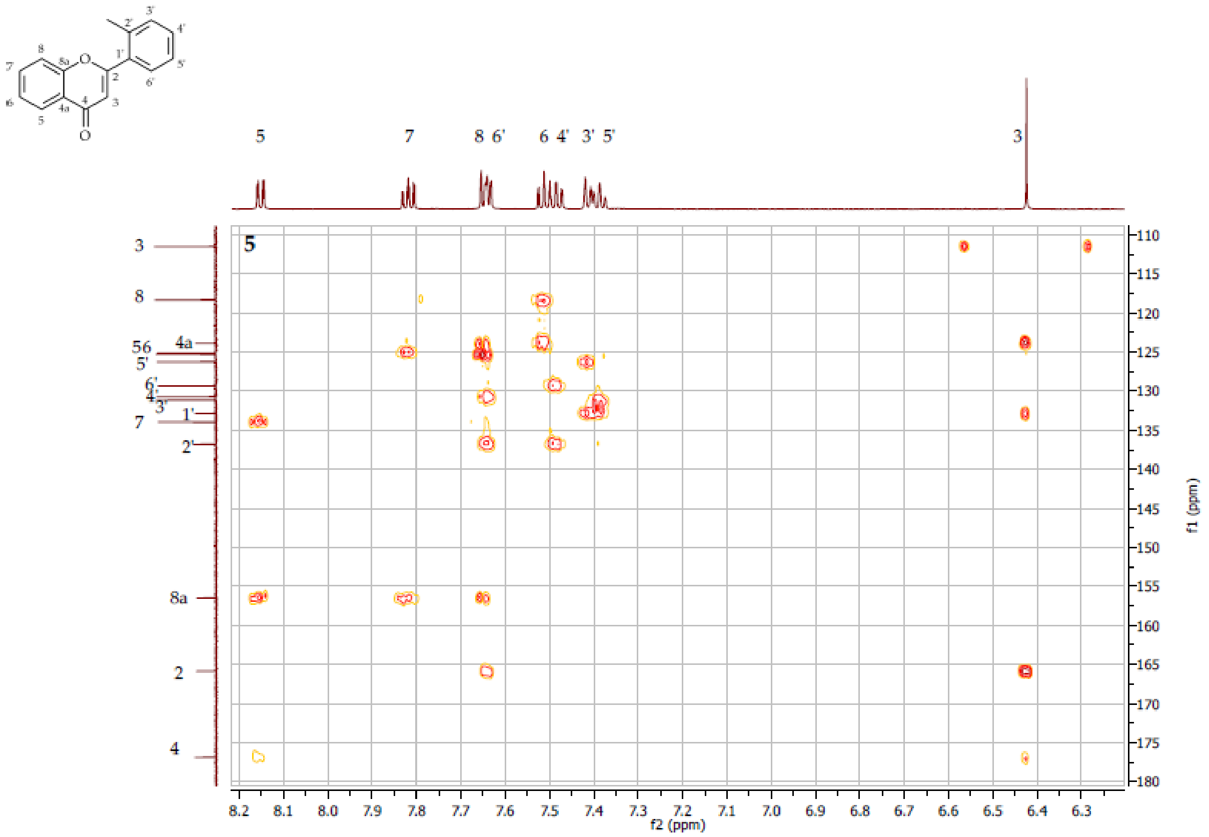

Figure S118. HMBC contour map – ^{1}H x ^{13}C expansion of 2′-methylflavone (**5**)

Molecular formula: $C_{23}H_{24}O_8$
Formula weight: 428.15
Ionization mode: positive
Precursor: $[M + H]^+$ 429.10
429.1000>253.0500 CE: -31.0
429.1000>121.0500 CE: -55.0

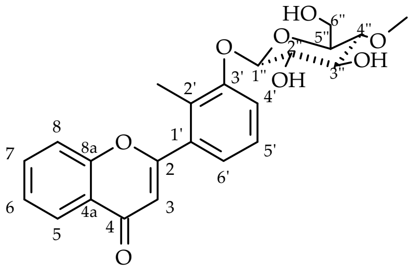

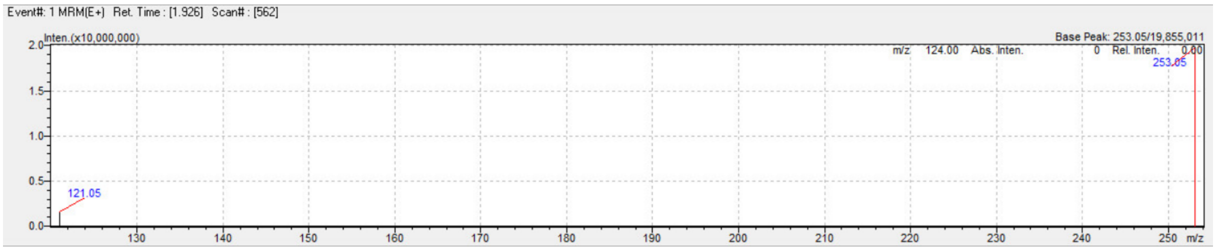

Figure S119. MS analysis of 2′-methylflavone 3′-*O*-*β*-D-(4′′-*O*-methyl)-glucopyranoside (**5a**)

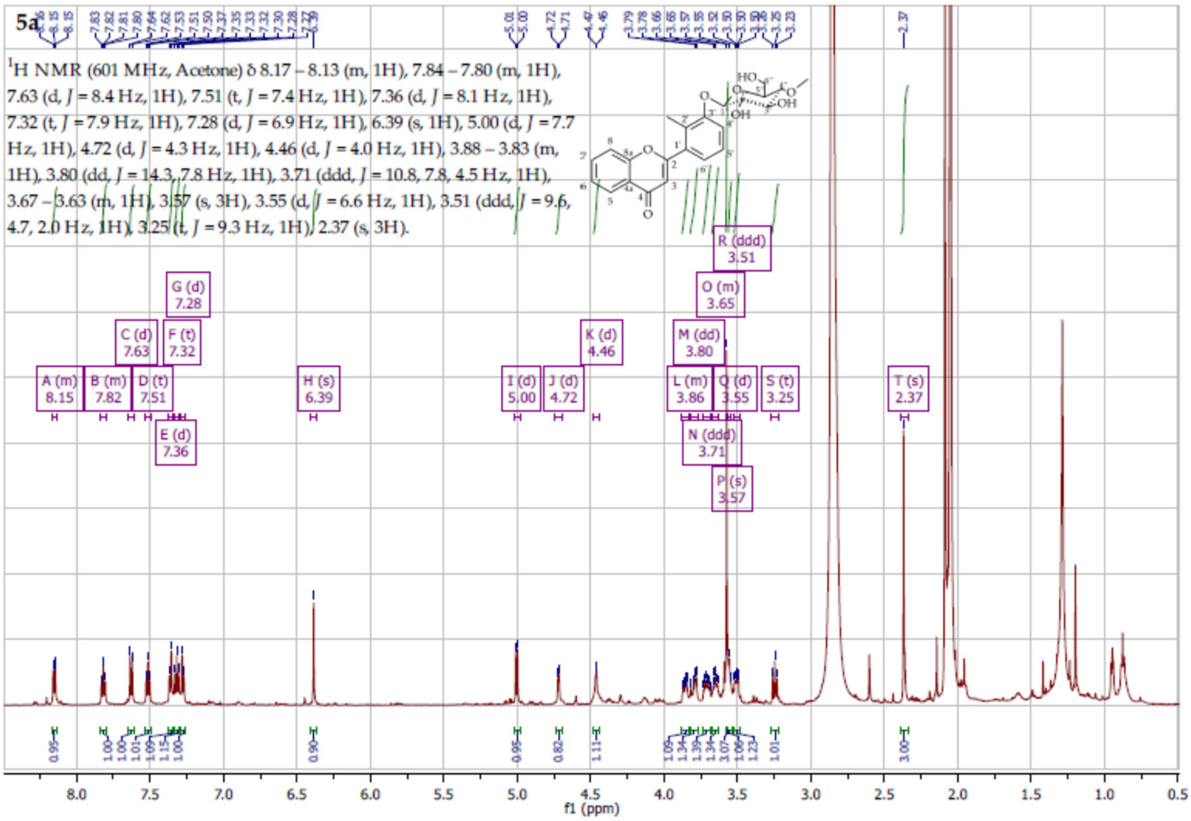

Figure S120. ^{1}H NMR spectrum (*δ*, acetone-d6, 600 MHz) of 2′-methylflavone 3′-*O*-*β*-D-(4′′-*O*-methyl)-glucopyranoside (**5a**)

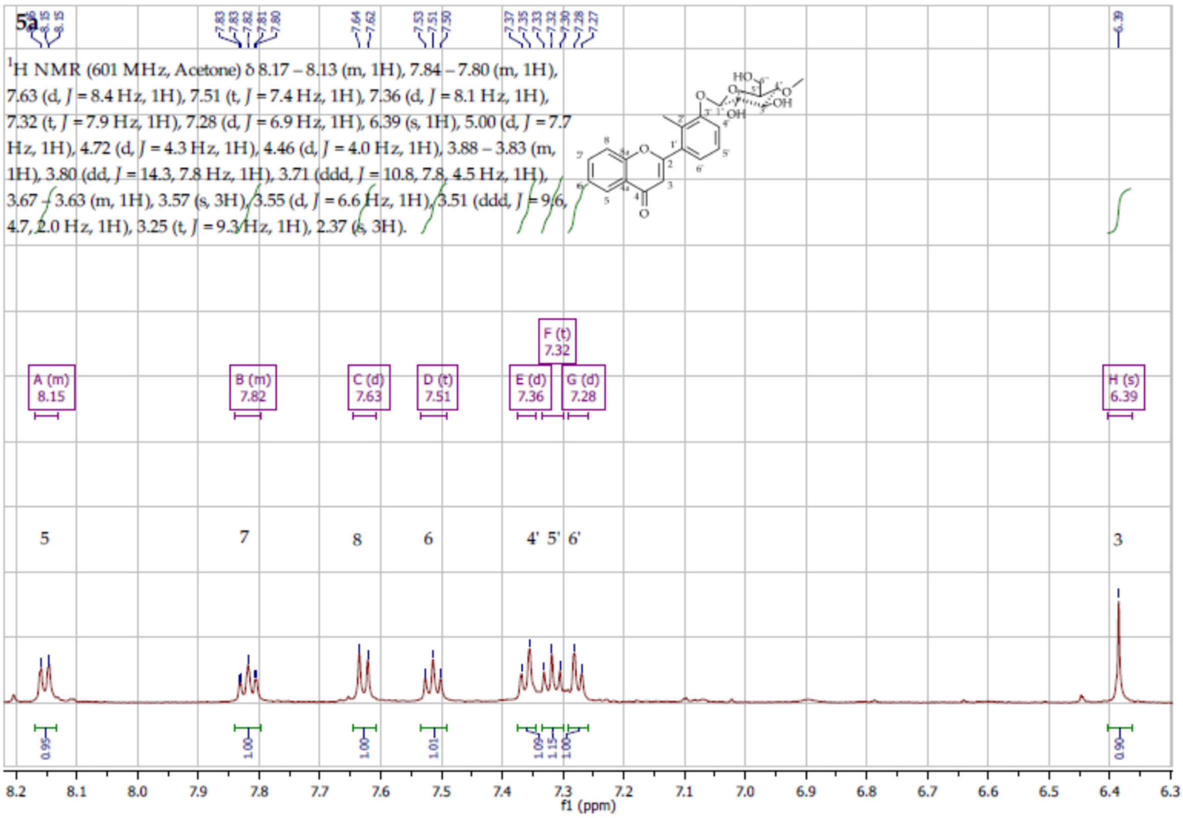

Figure S121. ^{1}H NMR spectrum expansion (*δ*, acetone-d6, 600 MHz) of 2′-methylflavone 3′-*O*-*β*-D-(4′′-*O*-methyl)-glucopyranoside (**5a**)

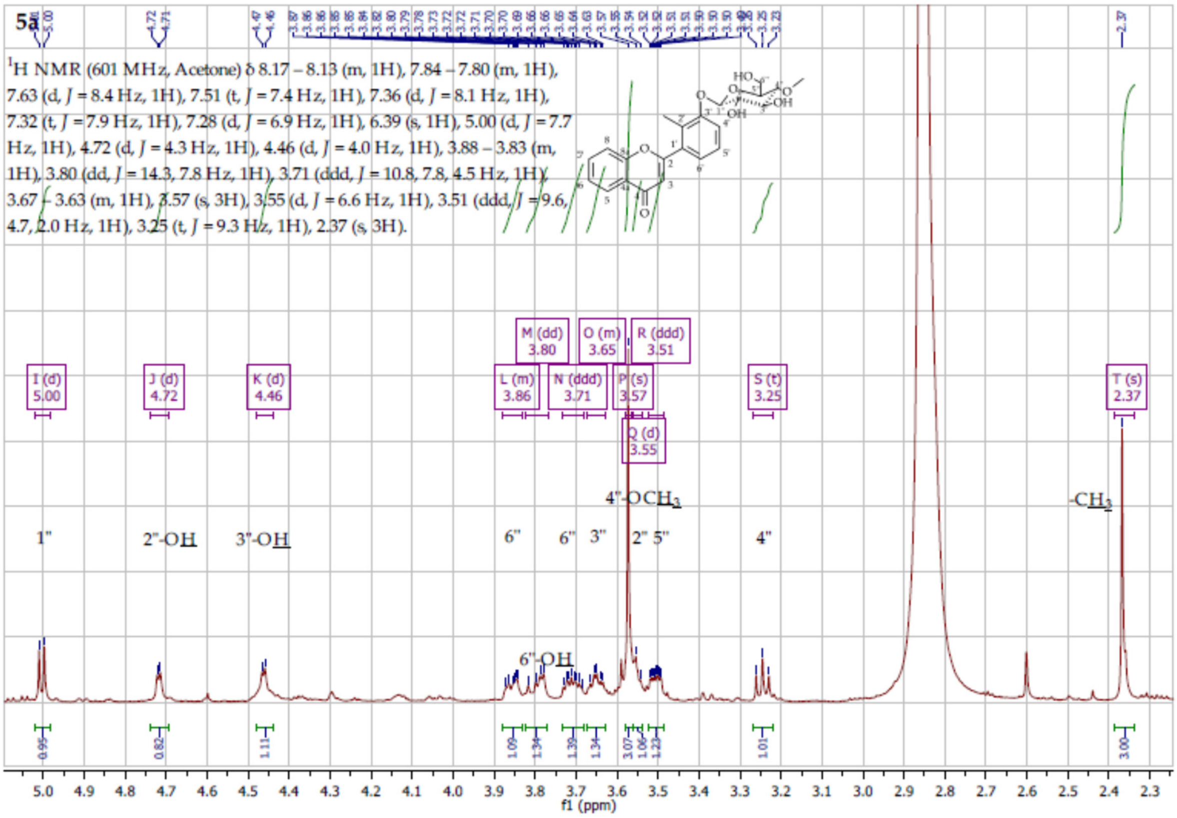

Figure S122. ^{1}H NMR spectrum expansion (*δ*, acetone-d6, 600 MHz) of 2′-methylflavone 3′-*O*-*β*-D-(4′′-*O*-methyl)-glucopyranoside (**5a**)

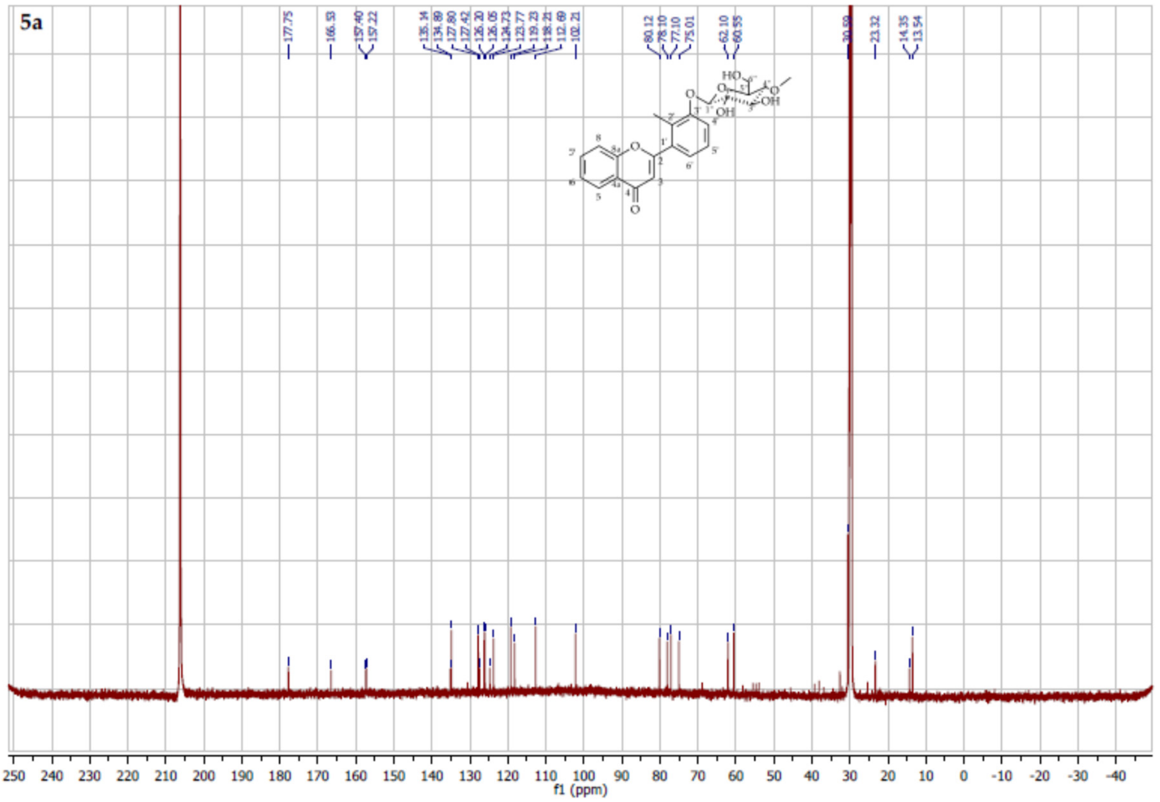

Figure S123. ^{13}C NMR spectrum (*δ*, acetone-d6, 151 MHz) of 2′-methylflavone 3′-*O*-*β*-D-(4′′-*O*-methyl)-glucopyranoside (**5a**)

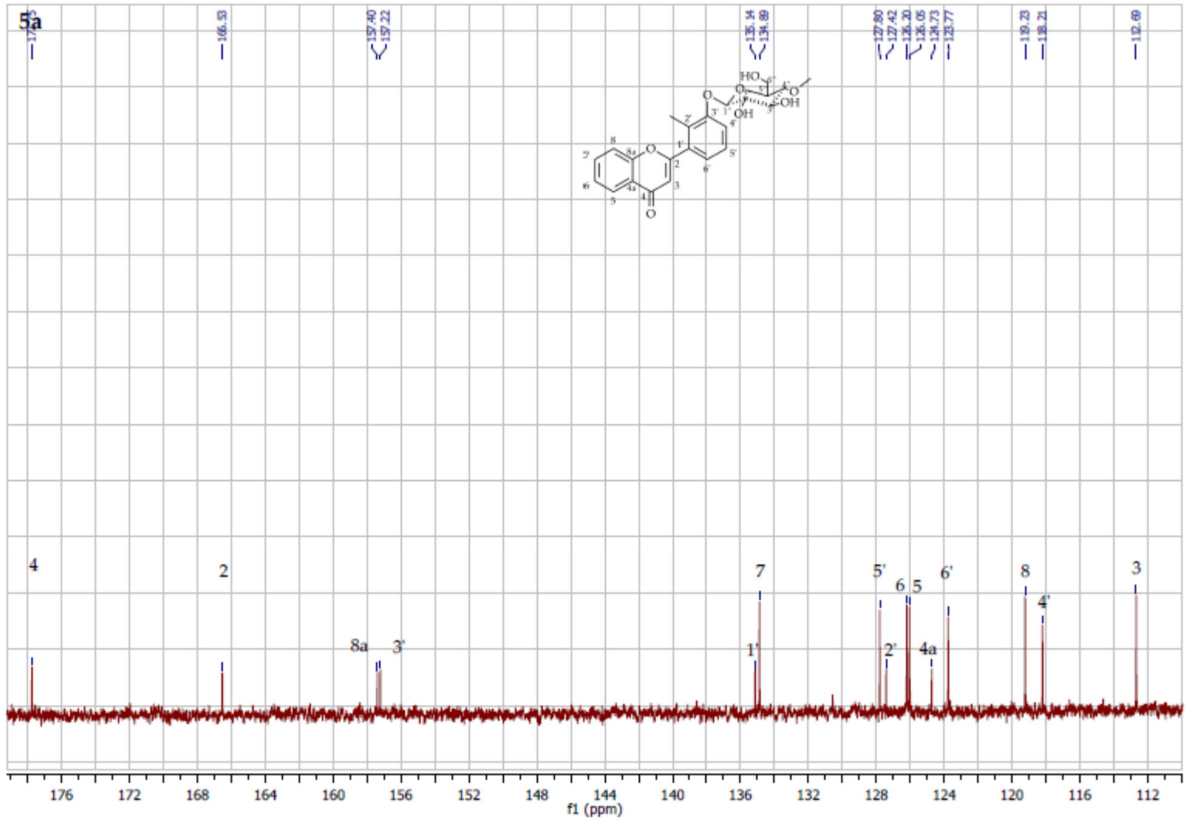

Figure S124. ^{13}C NMR spectrum expansion (*δ*, acetone-d6, 151 MHz) of 2′-methylflavone 3′-*O*-*β*-D-(4′′-*O*-methyl)-glucopyranoside (**5a**)

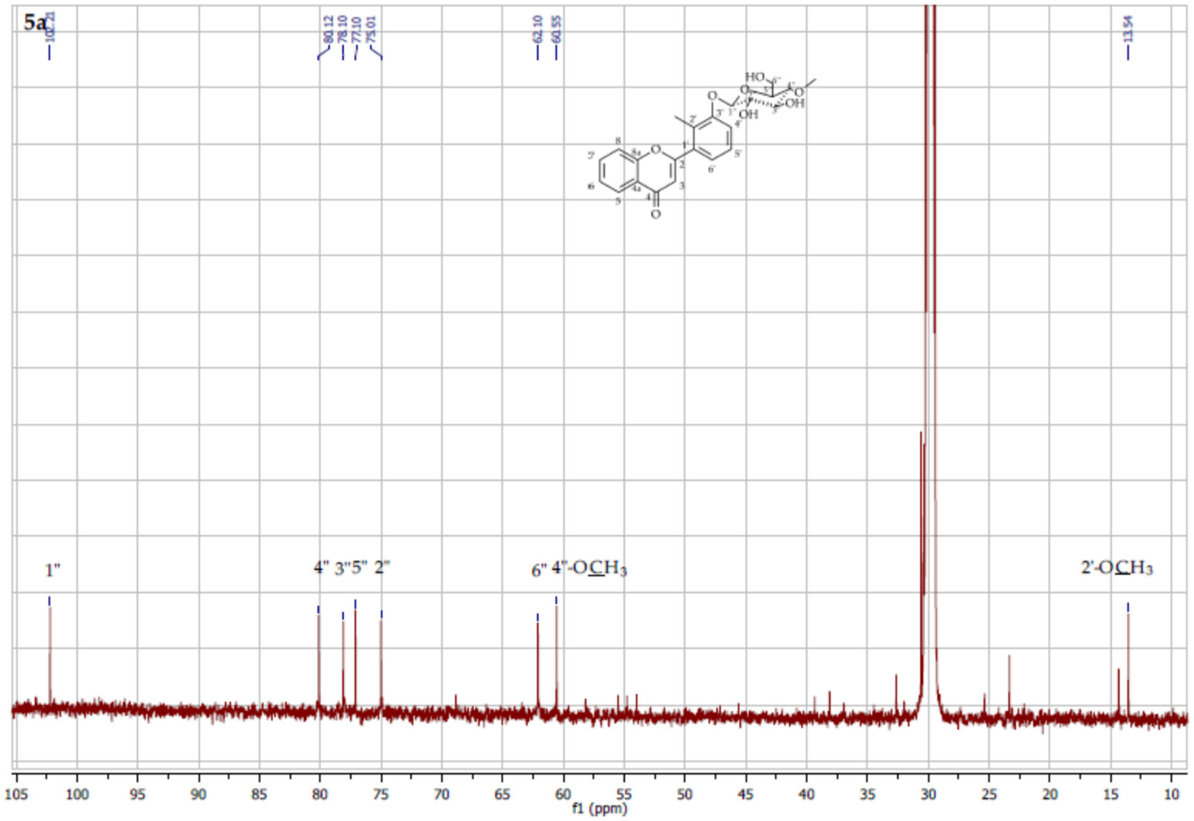

Figure S125. ^{13}C NMR spectrum expansion (*δ*, acetone-d6, 151 MHz) of 2′-methylflavone 3′-*O*-*β*-D-(4′′-*O*-methyl)-glucopyranoside (**5a**)

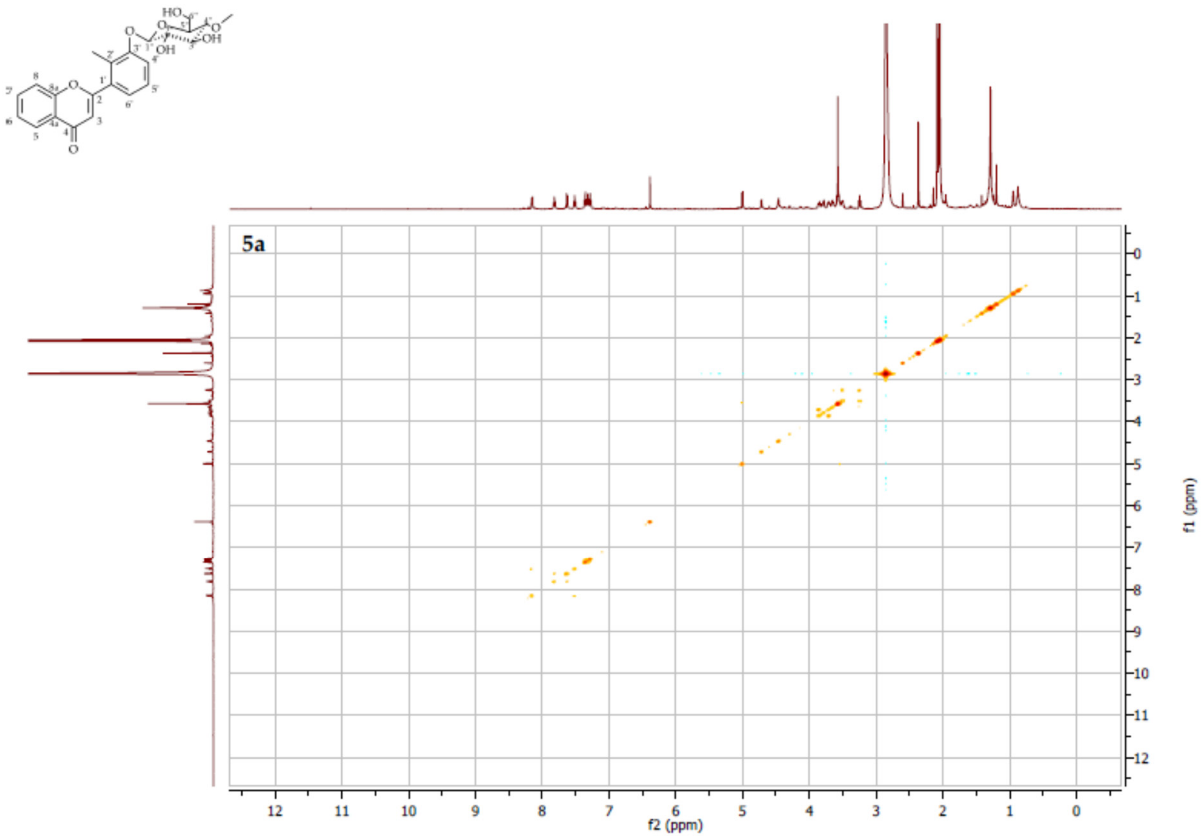

Figure S126. COSY contour map – ^{1}H x ^{1}H of 2′-methylflavone 3′-*O*-*β*-D-(4′′-*O*-methyl)-glucopyranoside (**5a**)

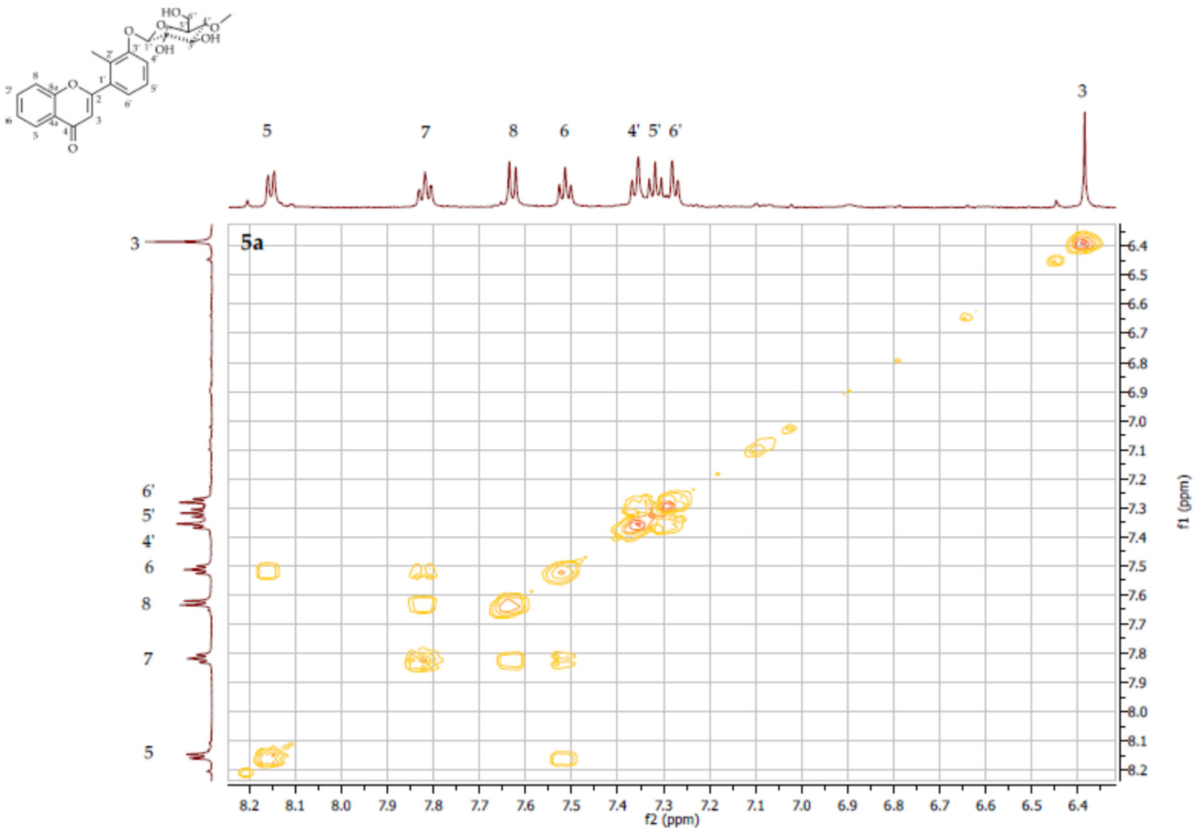

Figure S127. COSY contour map – ^{1}H x ^{1}H expansion of 2′-methylflavone 3′-*O*-*β*-D-(4′′-*O*-methyl)-glucopyranoside (**5a**)

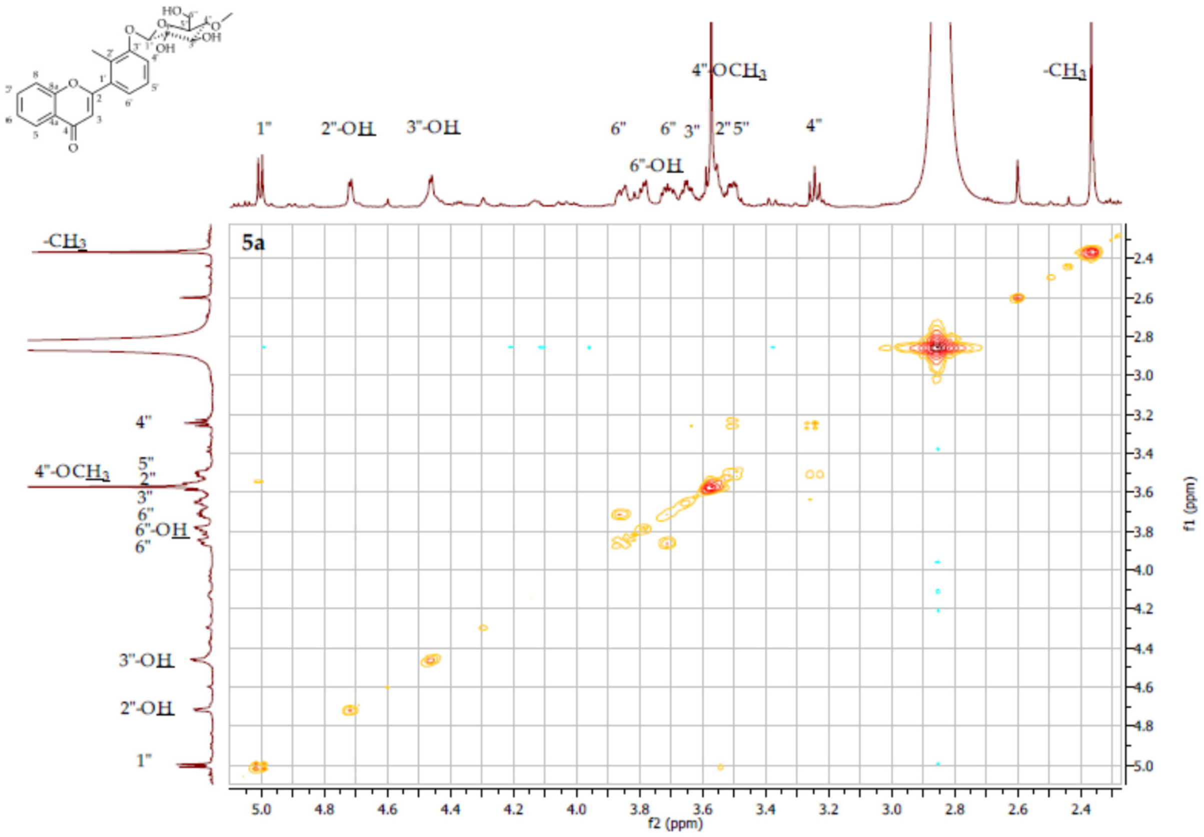

Figure S128. COSY contour map – ^{1}H x ^{1}H expansion of 2′-methylflavone 3′-*O*-*β*-D-(4′′-*O*-methyl)-glucopyranoside (**5a**)

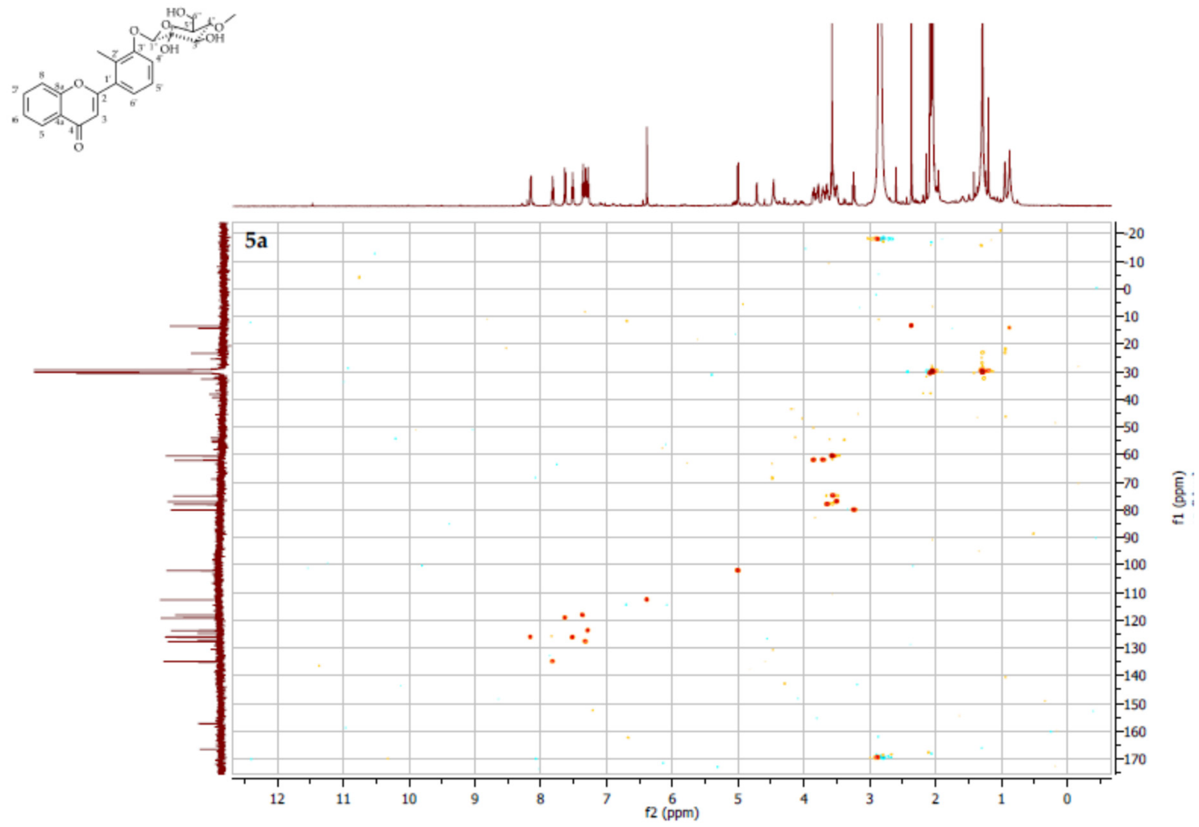

Figure S129. HSQC contour map – ^{1}H x ^{13}C of 2′-methylflavone 3′-*O*-*β*-D-(4′′-*O*-methyl)-glucopyranoside (**5a**)

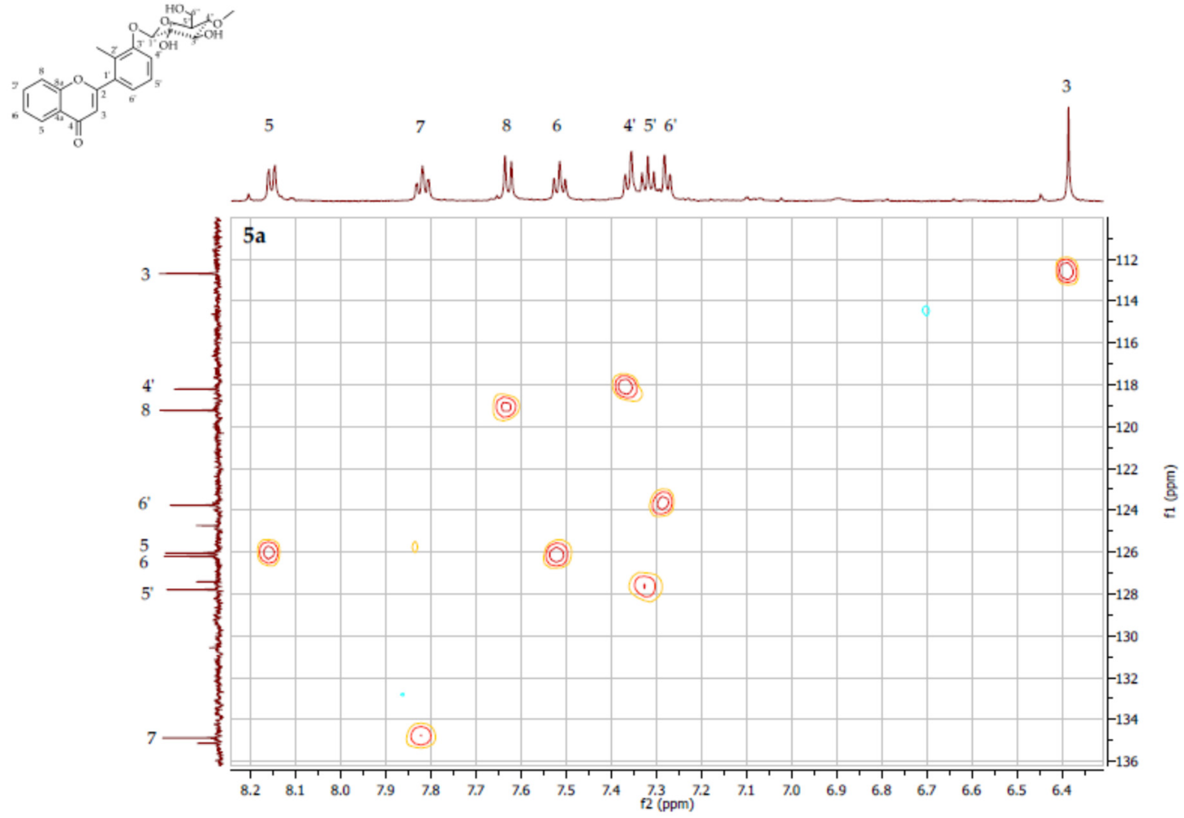

Figure S130. HSQC contour map – ^{1}H x ^{13}C expansion of 2′-methylflavone 3′-*O*-*β*-D-(4′′-*O*-methyl)-glucopyranoside (**5a**)

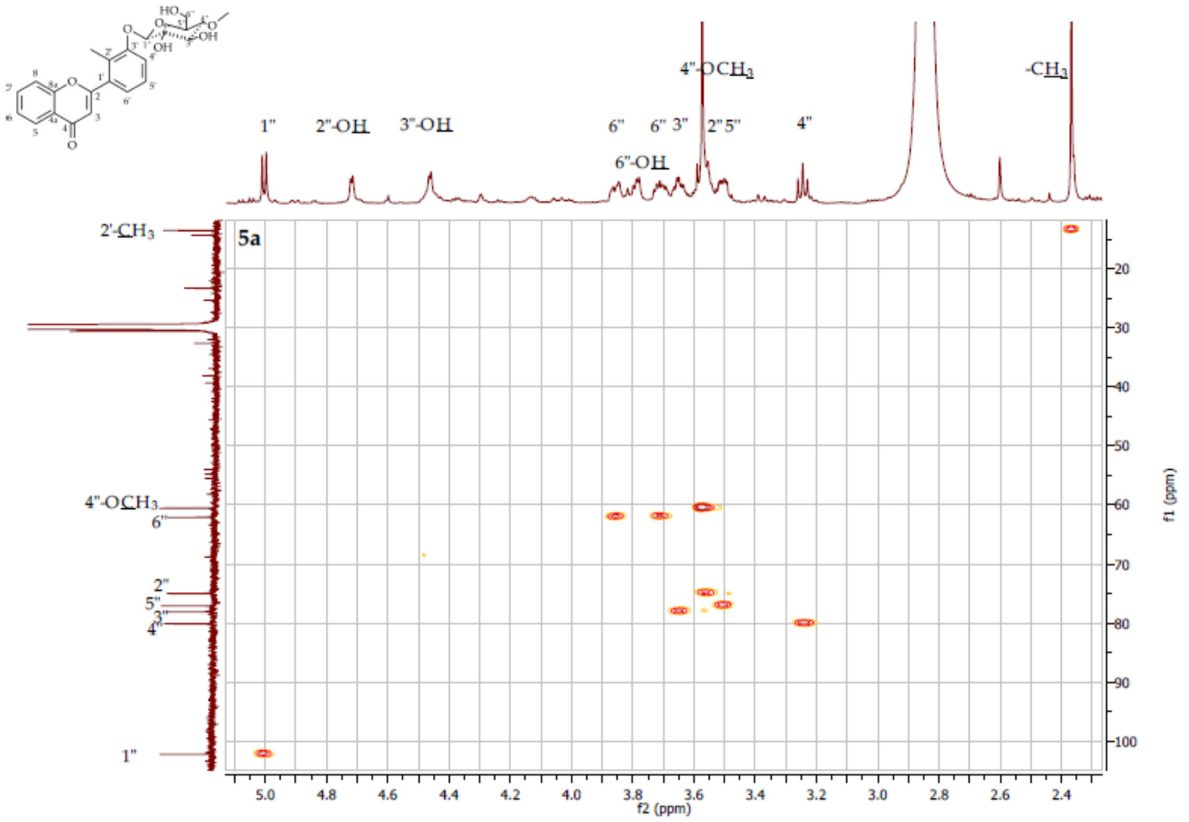

Figure S131. HSQC contour map – ^{1}H x ^{13}C expansion of 2′-methylflavone 3′-*O*-*β*-D-(4′′-*O*-methyl)-glucopyranoside (**5a**)

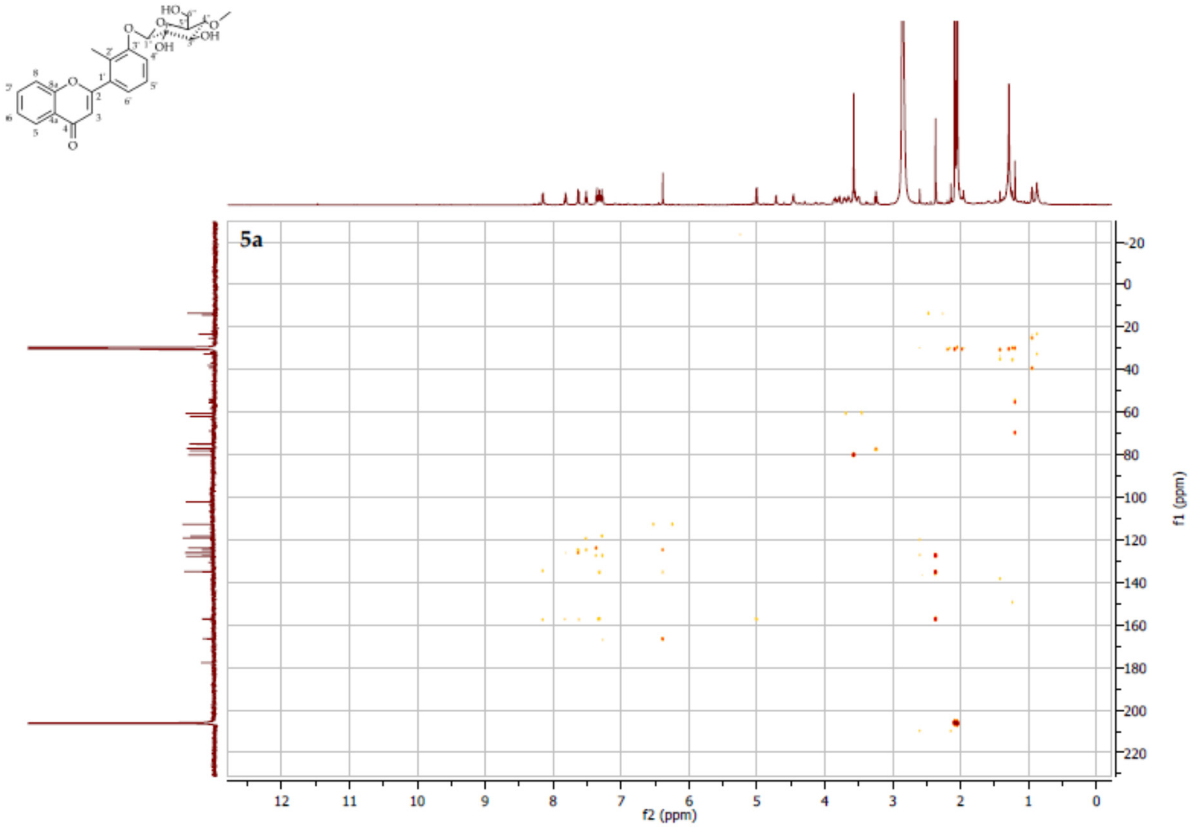

Figure S132. HMBC contour map – ^{1}H x ^{13}C expansion of 2′-methylflavone 3′-*O*-*β*-D-(4′′-*O*-methyl)-glucopyranoside (**5a**)

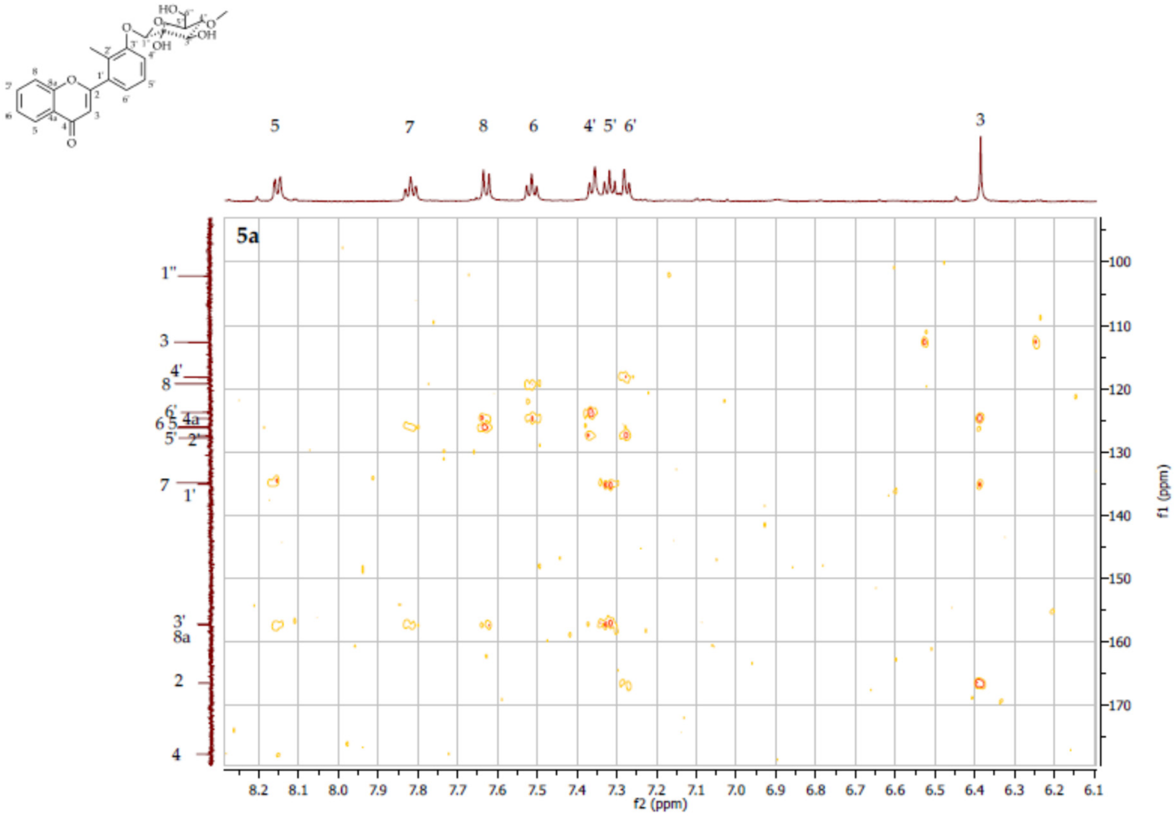

Figure S133. HMBC contour map – ^{1}H x ^{13}C expansion of 2′-methylflavone 3′-*O*-*β*-D-(4′′-*O*-methyl)-glucopyranoside (**5a**)

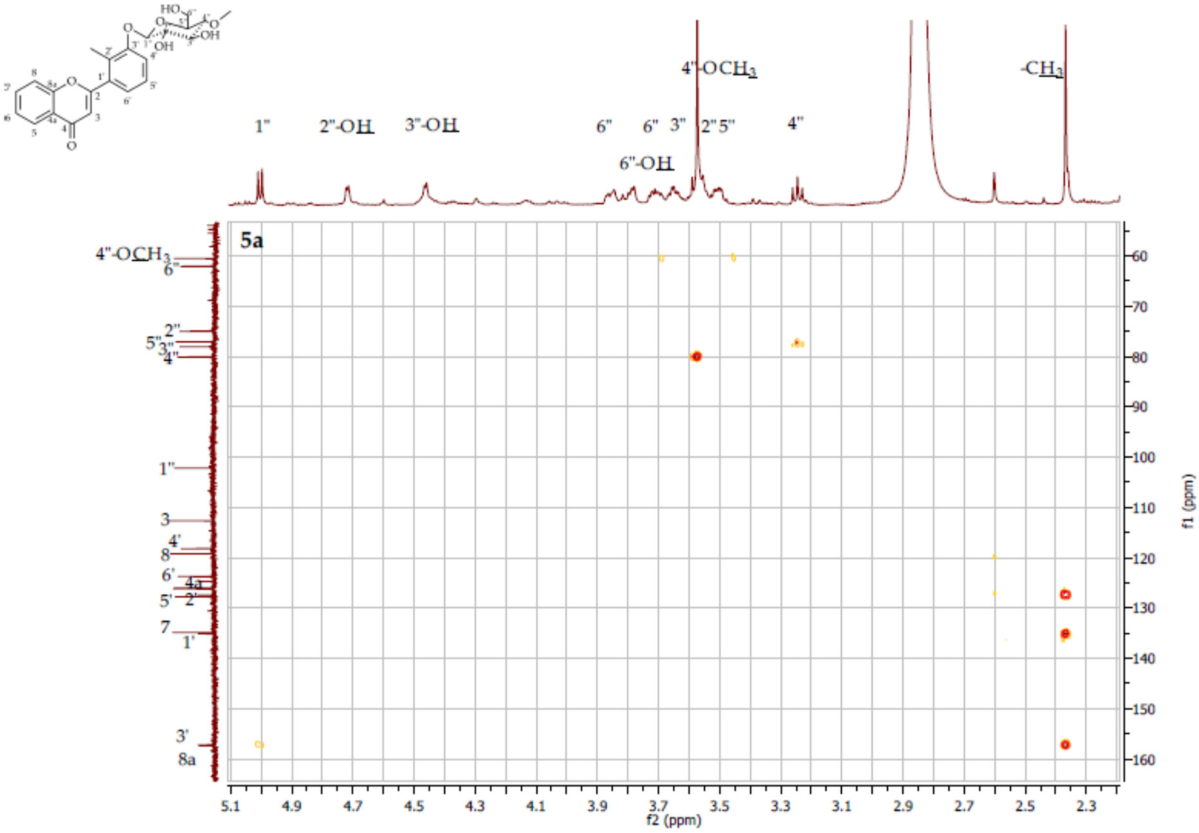

Figure S134. HMBC contour map – ^{1}H x ^{13}C expansion of 2′-methylflavone 3′-*O*-*β*-D-(4′′-*O*-methyl)-glucopyranoside (**5a**)

Molecular formula: $C_{23}H_{24}O_8$
Formula weight: 428.15
Ionization mode: positive
Precursor: $[M + H]^+$ 429.10
429.1000>253.1000 CE: -22.0
429.1000>121.0000 CE: -55.0
429.1000>159.0000 CE: -53.0

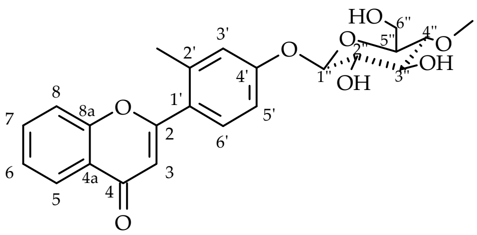

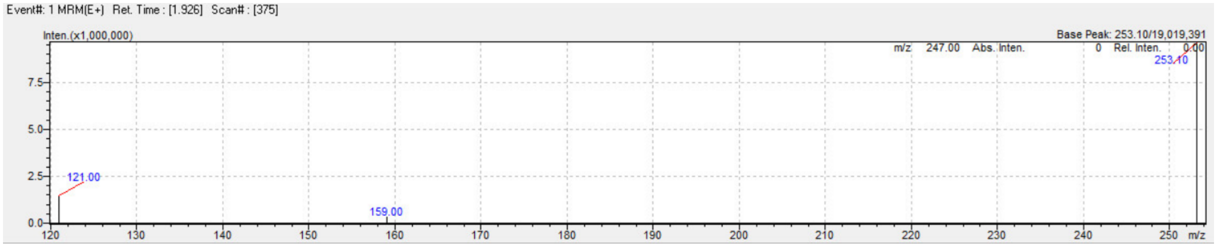

Figure S135. MS analysis of 2′-methylflavone 4′-*O*-*β*-D-(4′′-*O*-methyl)-glucopyranoside (**5b**)

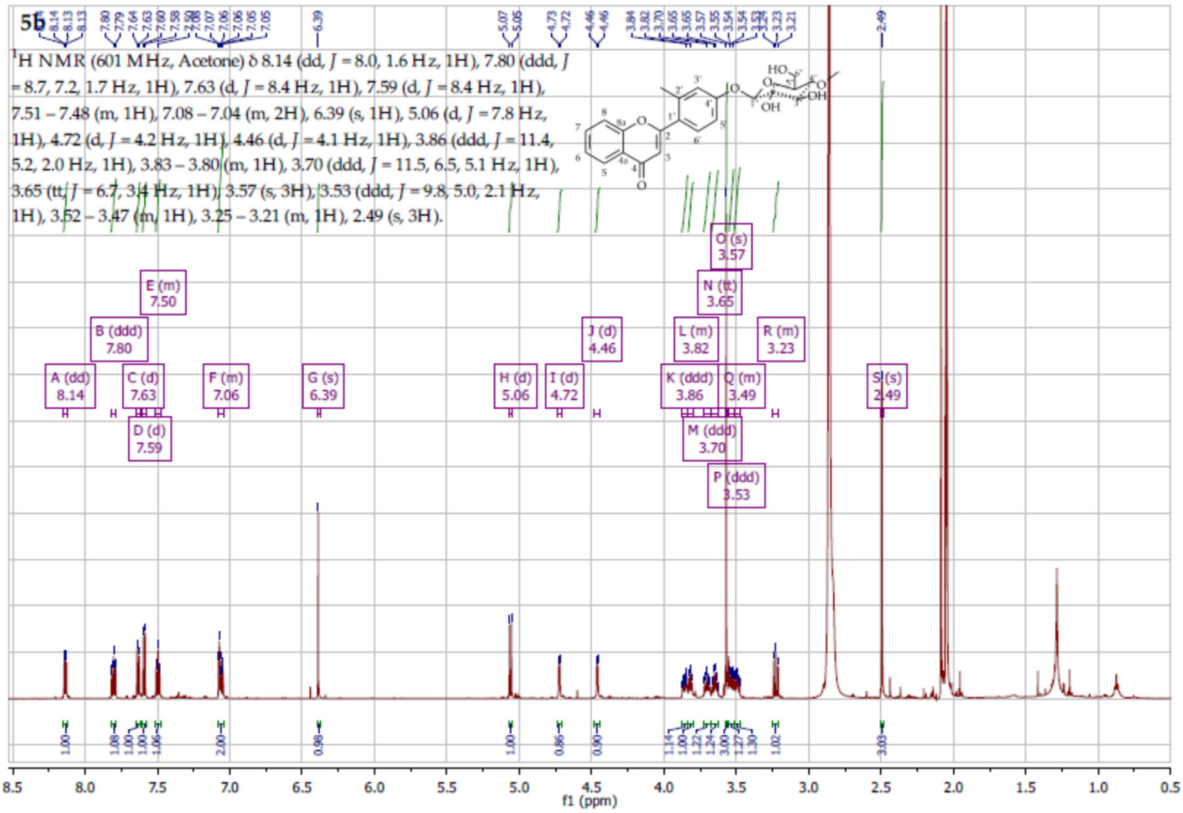

Figure S136. ¹H NMR spectrum (*δ*, acetone-d6, 600 MHz) of 2′-methylflavone 4′-*O*-*β*-D-(4′′-*O*-methyl)-glucopyranoside (**5b**)

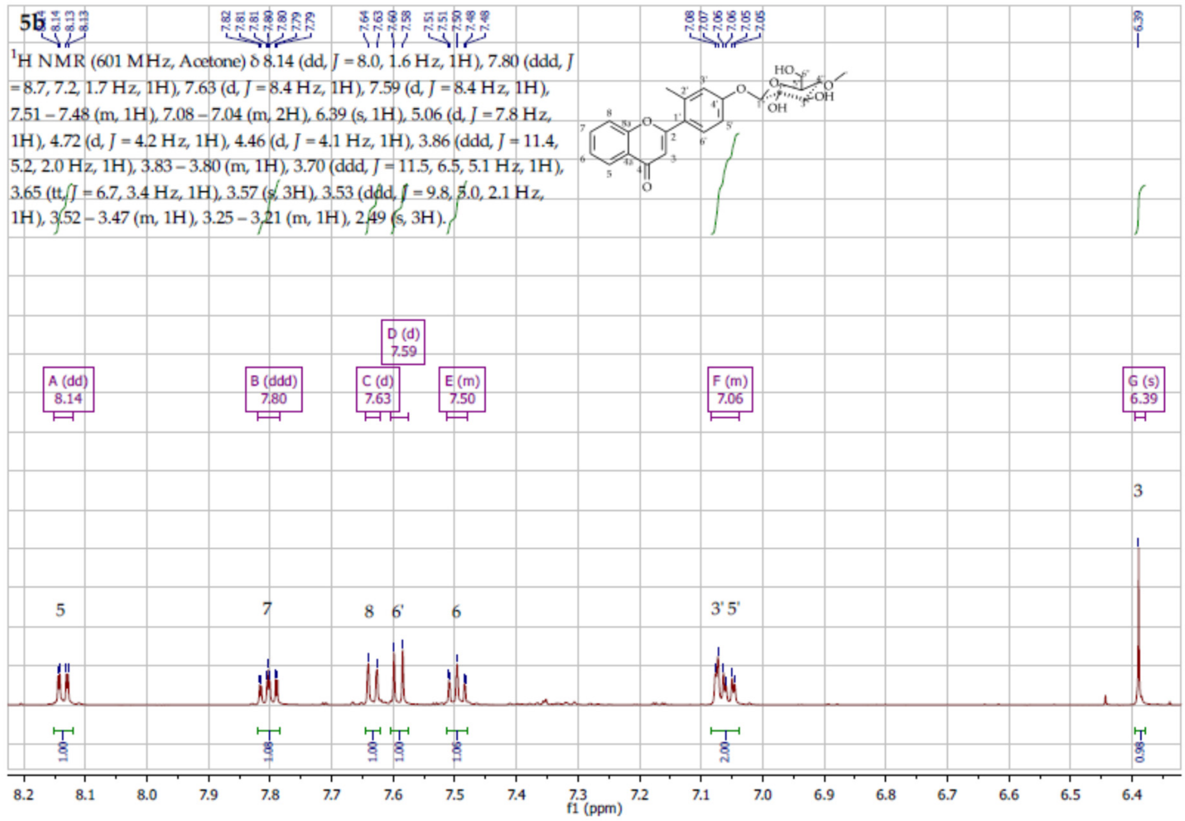

Figure S137. ¹H NMR spectrum expansion (δ, acetone-d6, 600 MHz) of 2′-methylflavone 4′-*O*-*β*-D-(4′′-*O*-methyl)-glucopyranoside (**5b**)

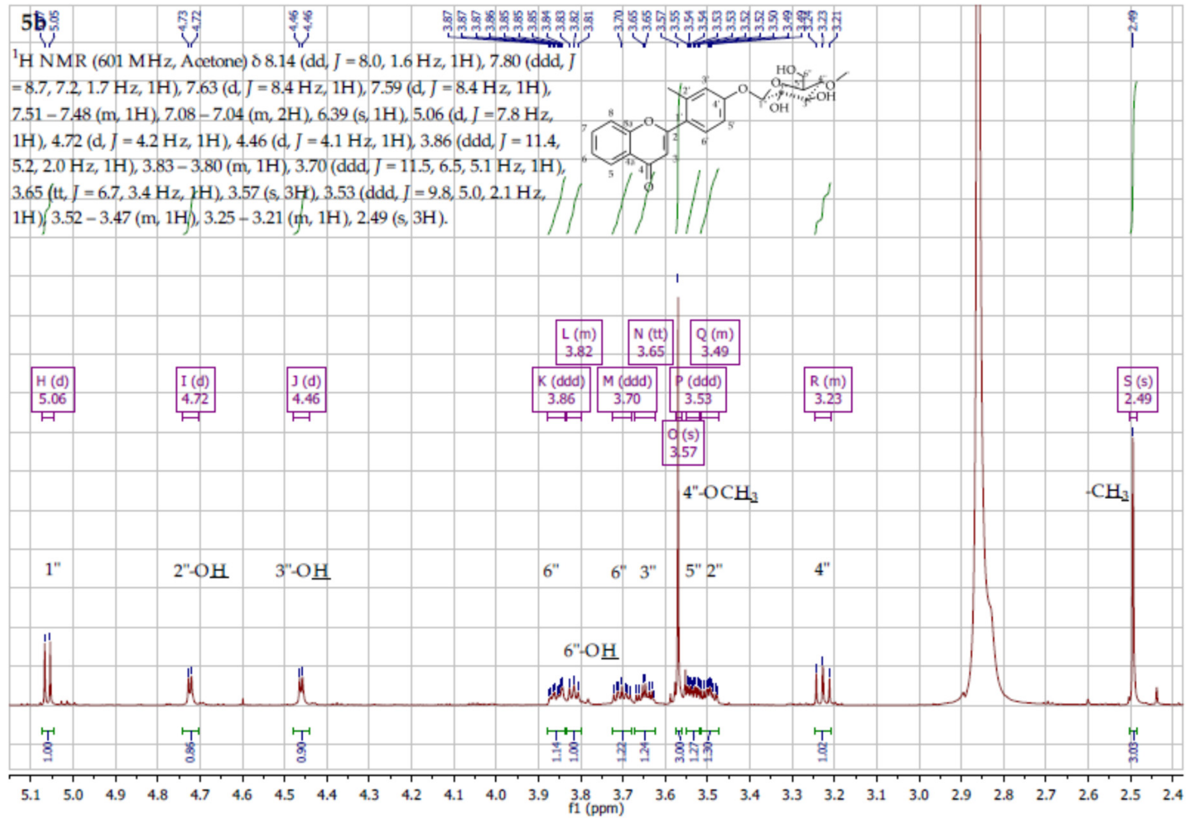

Figure S138. ¹H NMR spectrum expansion (δ, acetone-d6, 600 MHz) of 2′-methylflavone 4′-*O*-*β*-D-(4′′-*O*-methyl)-glucopyranoside (**5b**)

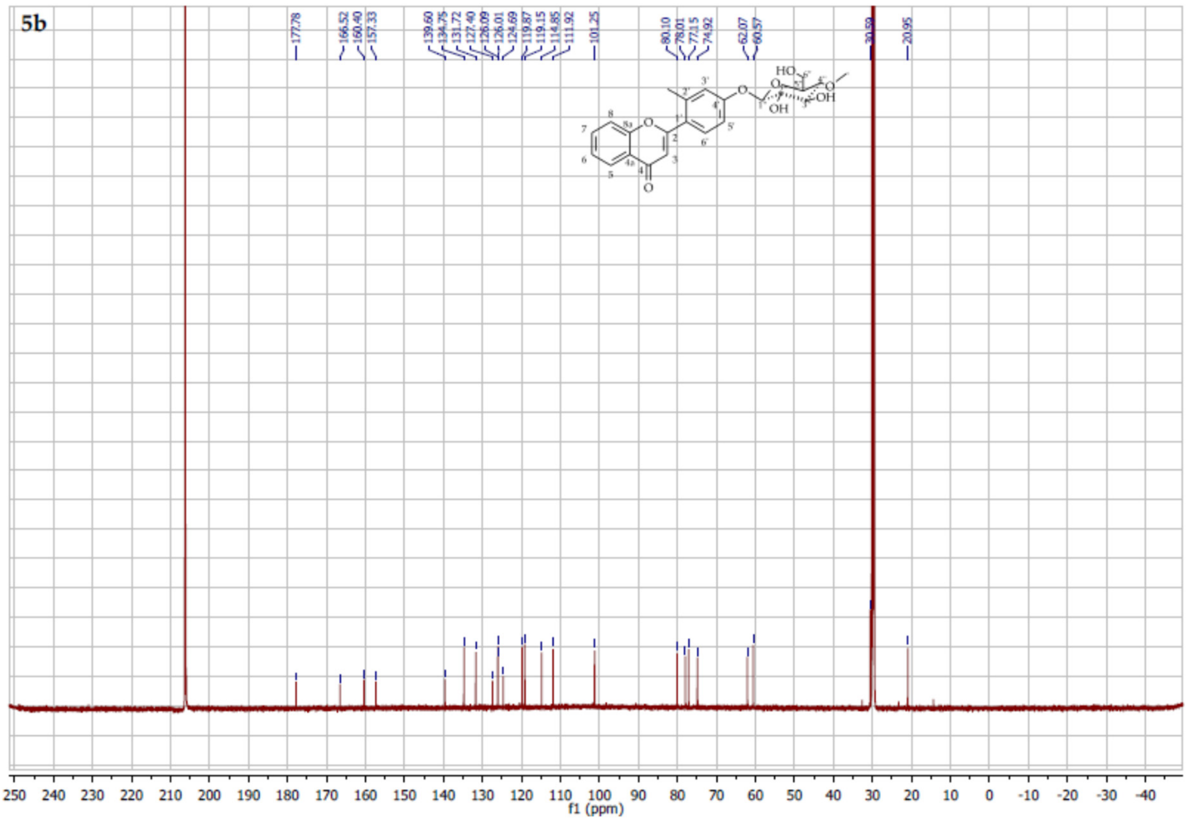

Figure S139. ^{13}C NMR spectrum of (*δ*, acetone-d6, 151 MHz) 2′-methylflavone 4′-*O*-*β*-D-(4′′-*O*-methyl)-glucopyranoside (**5b**)

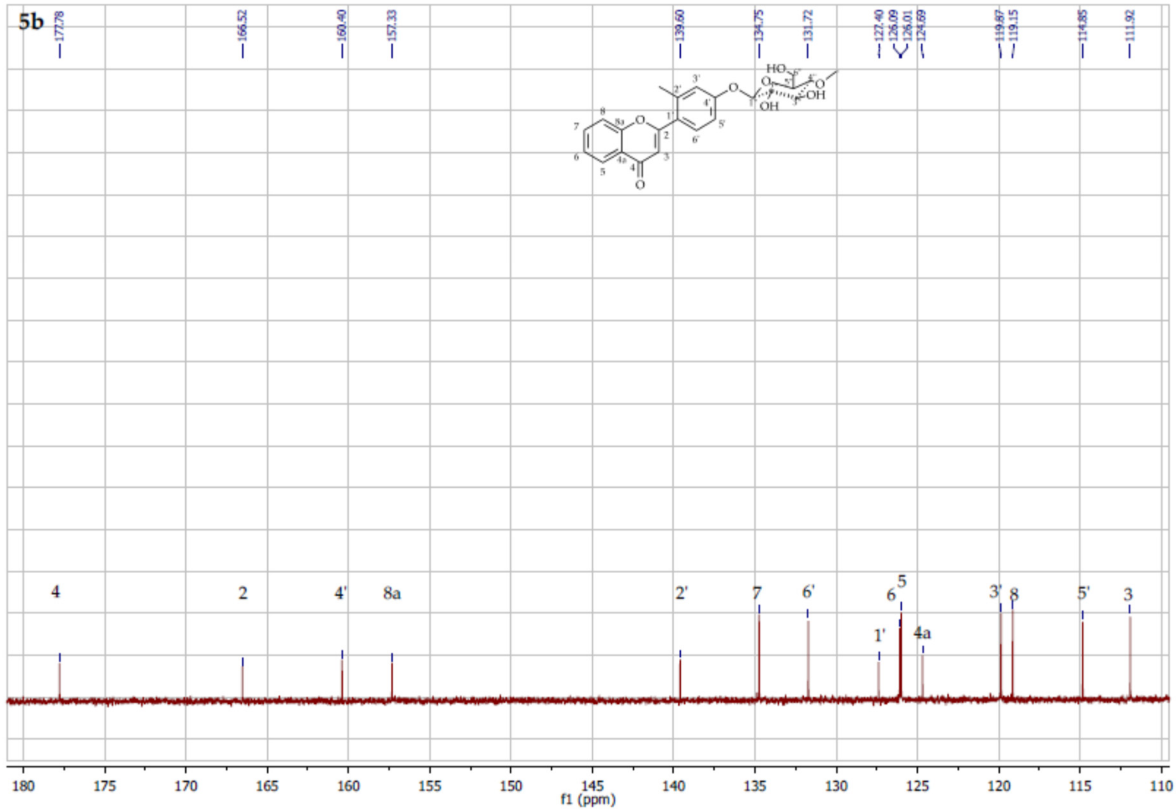

Figure S140. ^{13}C NMR spectrum expansion (*δ*, acetone-d6, 151 MHz) of 2′-methylflavone 4′-*O*-*β*-D-(4′′-*O*-methyl)-glucopyranoside (**5b**)

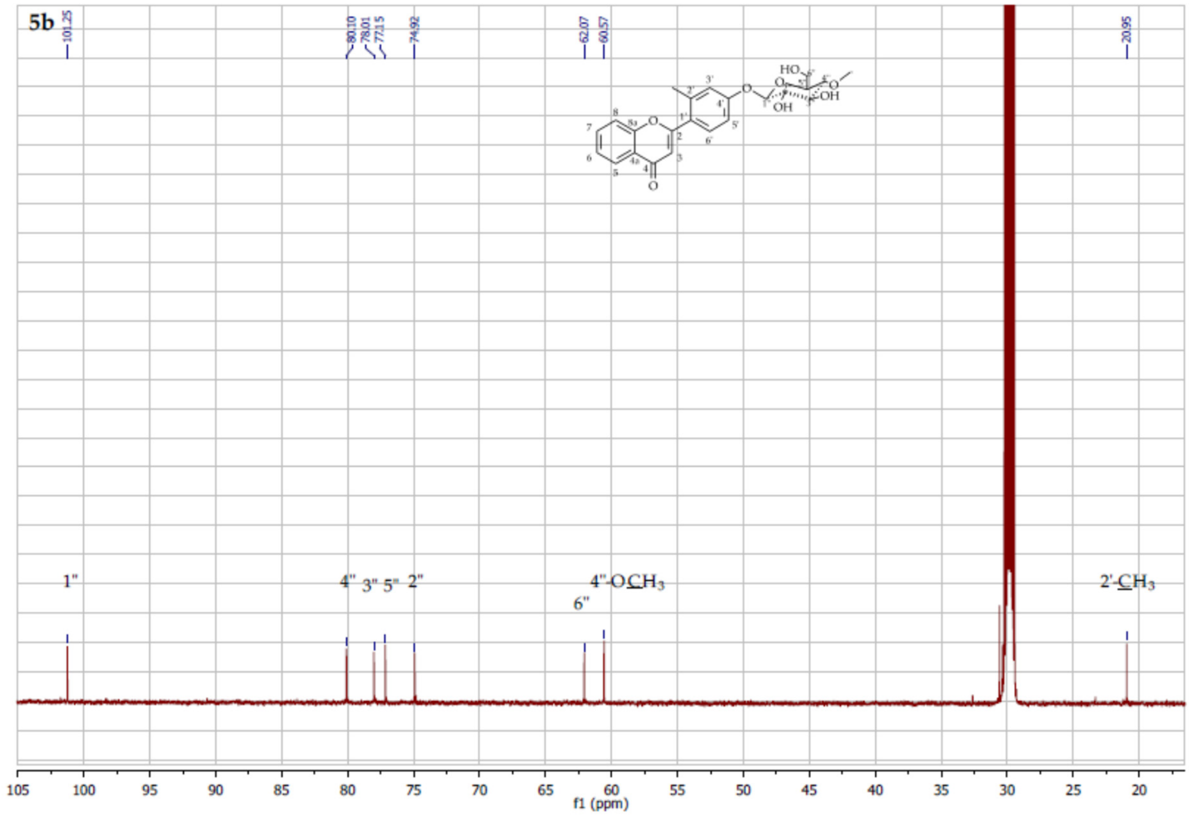

Figure S141. ^{13}C NMR spectrum expansion (δ, acetone-d6, 151 MHz) of 2′-methylflavone 4′-*O*-*β*-D-(4′′-*O*-methyl)-glucopyranoside (**5b**)

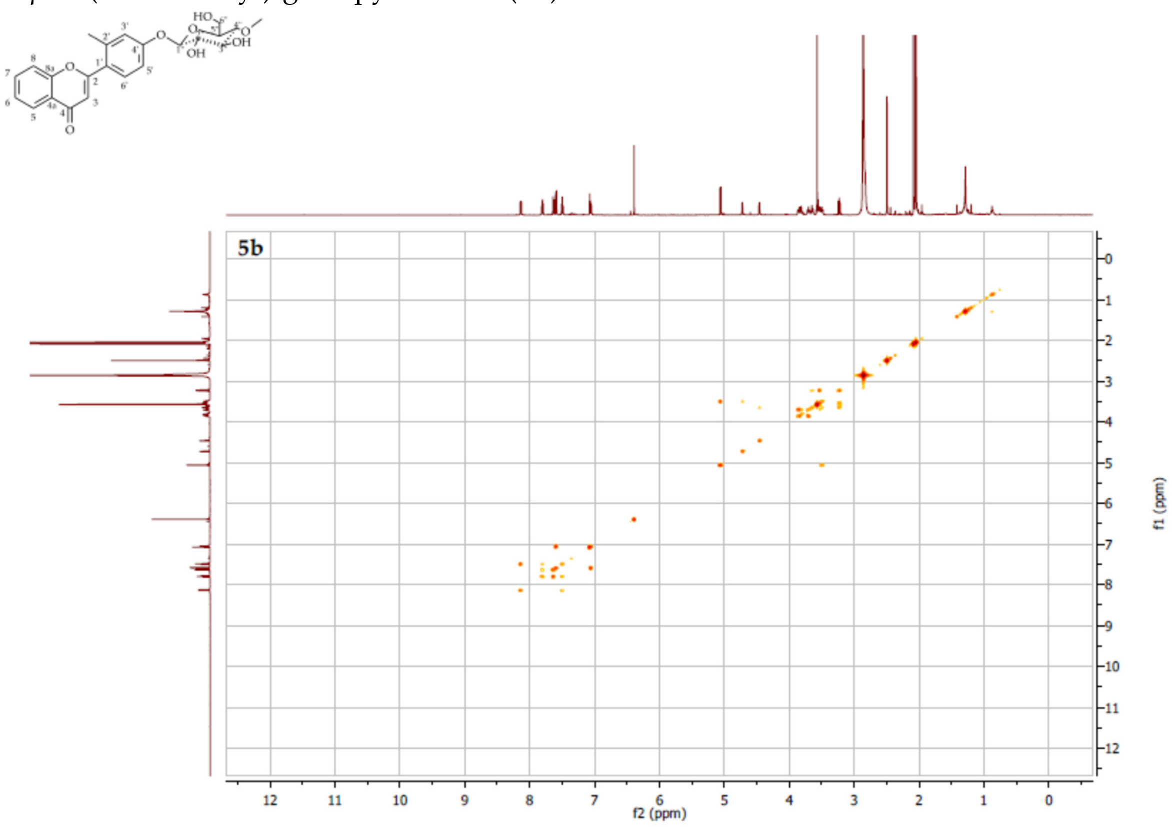

Figure S142. COSY contour map – ^{1}H x ^{1}H 2′-methylflavone 4′-*O*-*β*-D-(4′′-*O*-methyl)-glucopyranoside (**5b**)

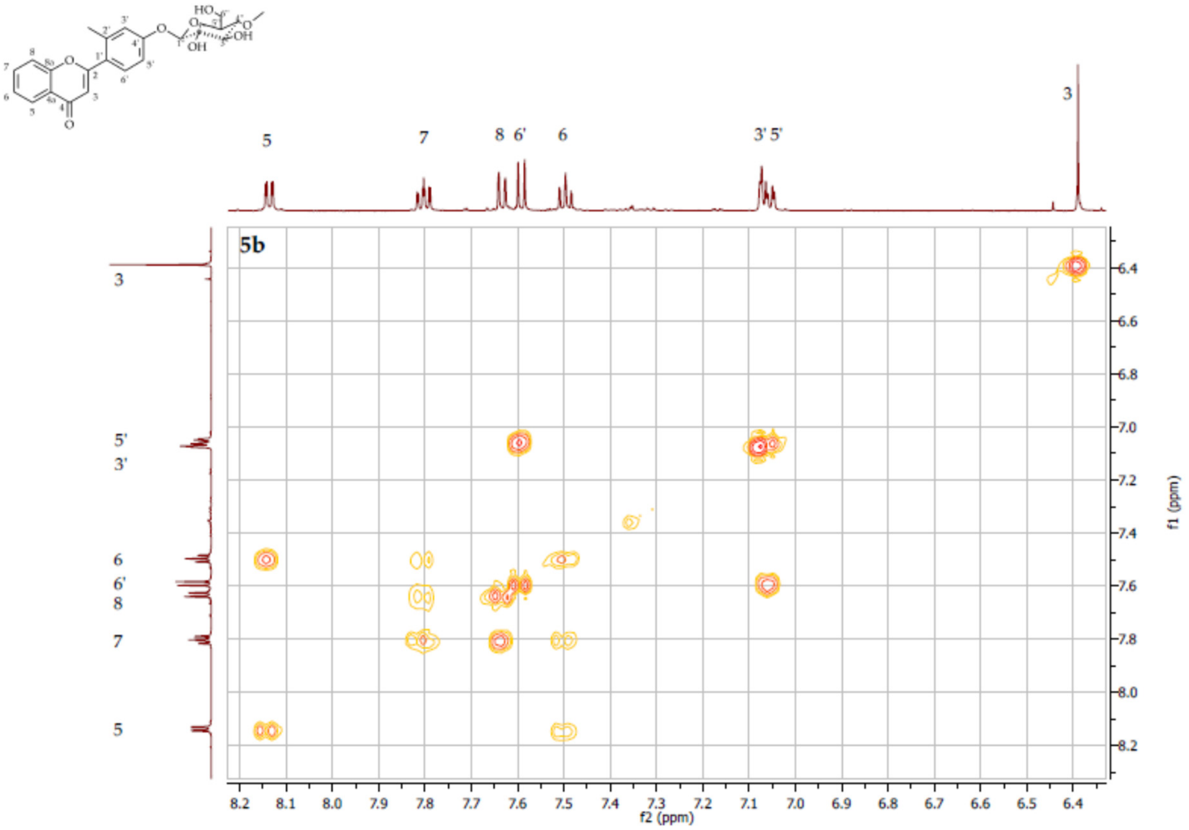

Figure S143. COSY contour map – ^1H x ^1H expansion of 2′-methylflavone 4′-*O*-*β*-D-(4′′-*O*-methyl)-glucopyranoside (**5b**)

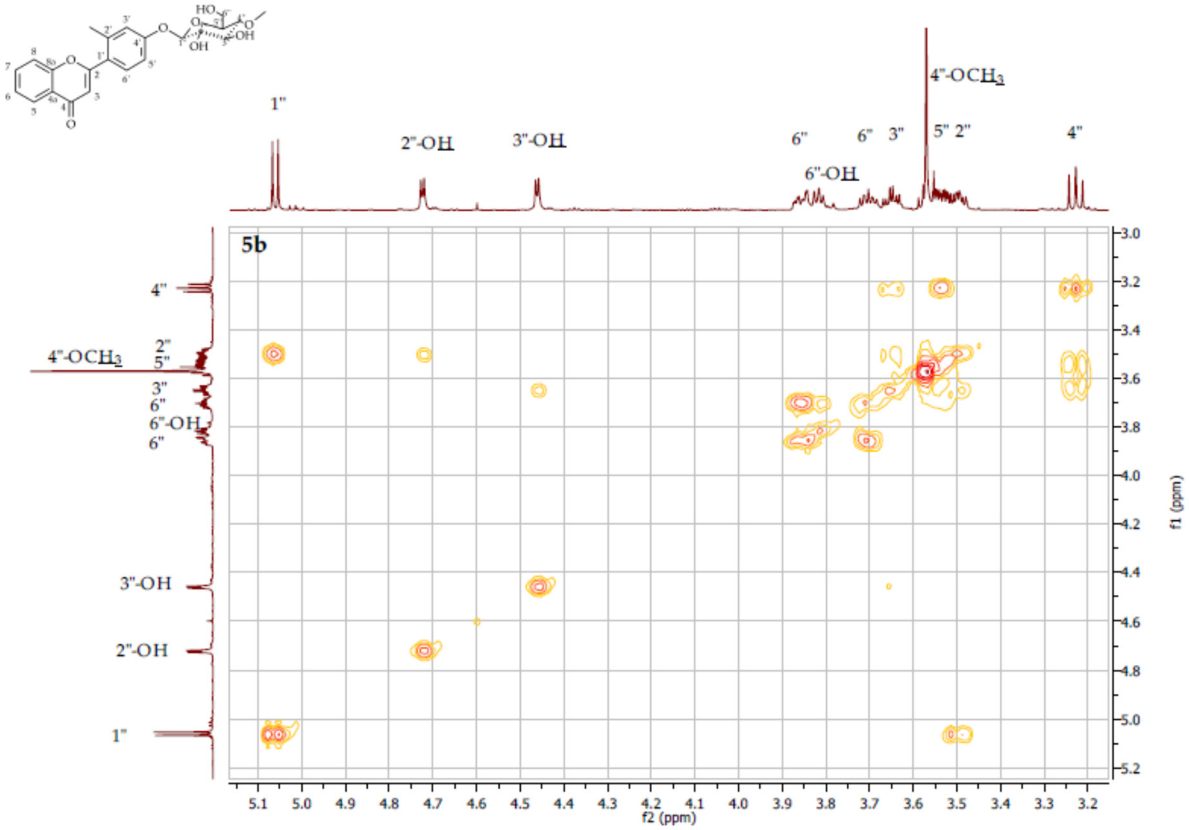

Figure S144. COSY contour map – ^1H x ^1H expansion of 2′-methylflavone 4′-*O*-*β*-D-(4′′-*O*-methyl)-glucopyranoside (**5b**)

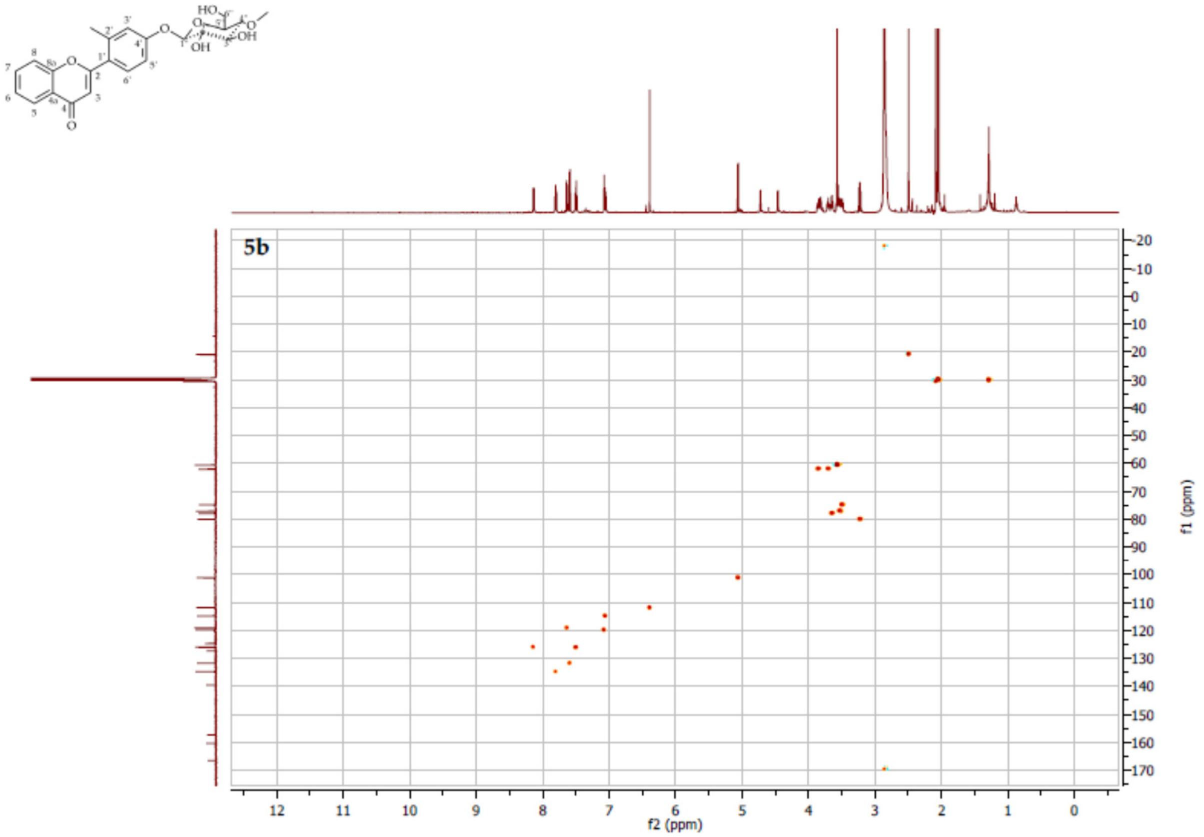

Figure S145. HSQC contour map – ^{1}H x ^{13}C of 2′-methylflavone 4′-*O*-*β*-D-(4′′-*O*-methyl)-glucopyranoside (**5b**)

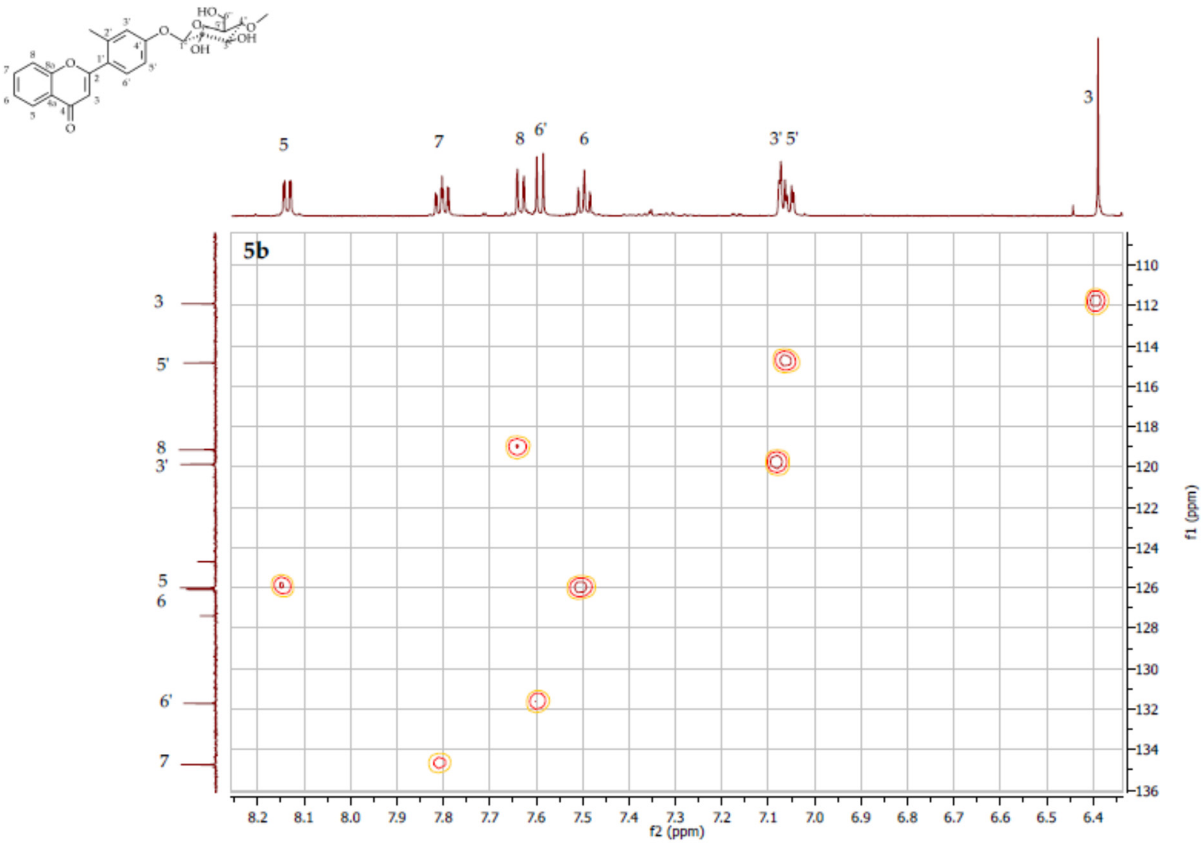

Figure S146. HSQC contour map – ^{1}H x ^{13}C expansion of 2′-methylflavone 4′-*O*-*β*-D-(4′′-*O*-methyl)-glucopyranoside (**5b**)

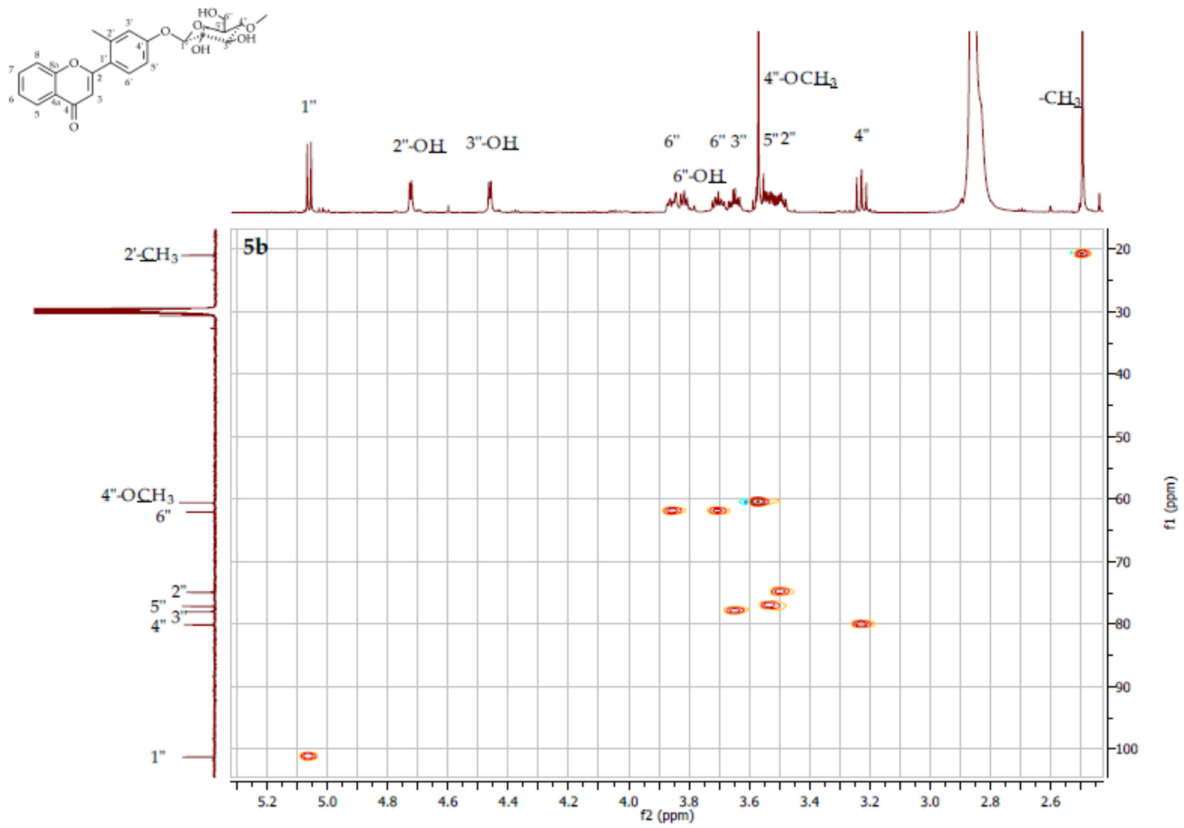

Figure S147. HSQC contour map – ^{1}H x ^{13}C expansion of 2′-methylflavone 4′-*O*-*β*-D-(4′′-*O*-methyl)-glucopyranoside (**5b**)

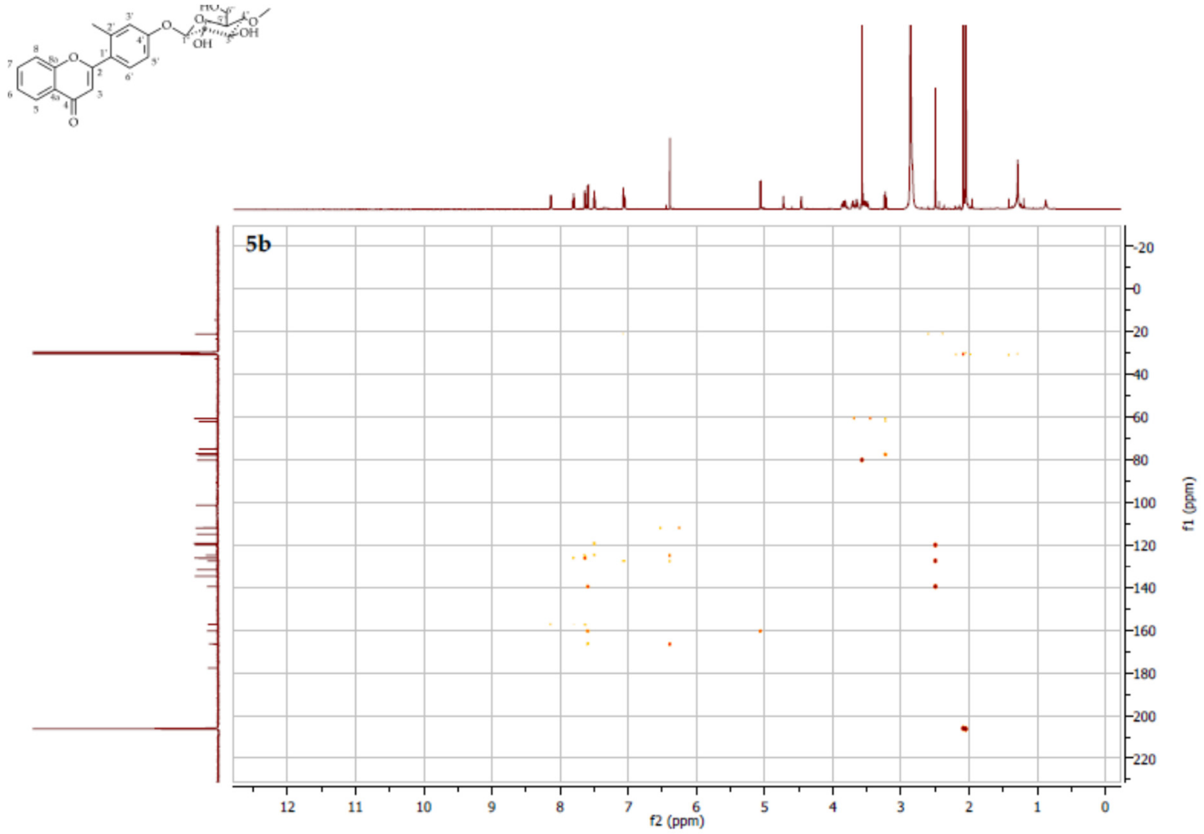

Figure S148. HMBC contour map – ^{1}H x ^{13}C of 2′-methylflavone 4′-*O*-*β*-D-(4′′-*O*-methyl)-glucopyranoside (**5b**)

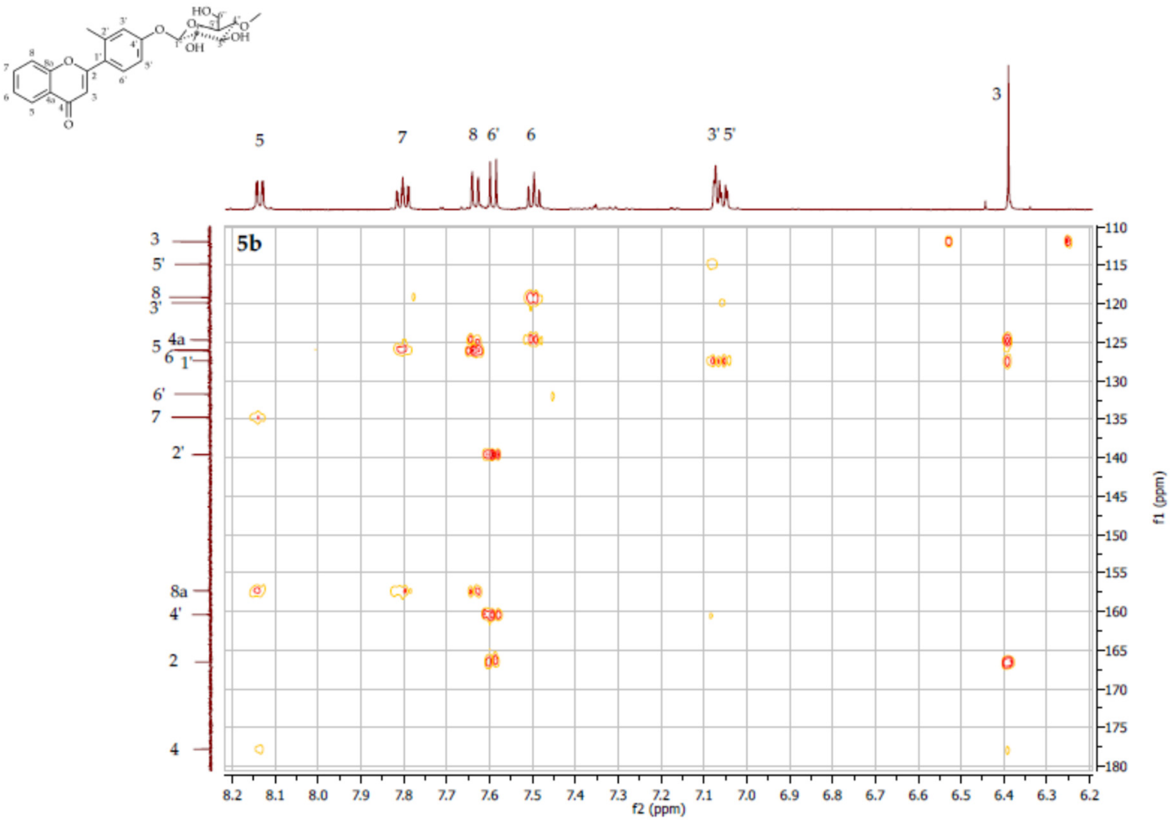

Figure S149. HMBC contour map – ^{1}H x ^{13}C expansion of 2′-methylflavone 4′-*O*-*β*-D-(4′′-*O*-methyl)-glucopyranoside (**5b**)

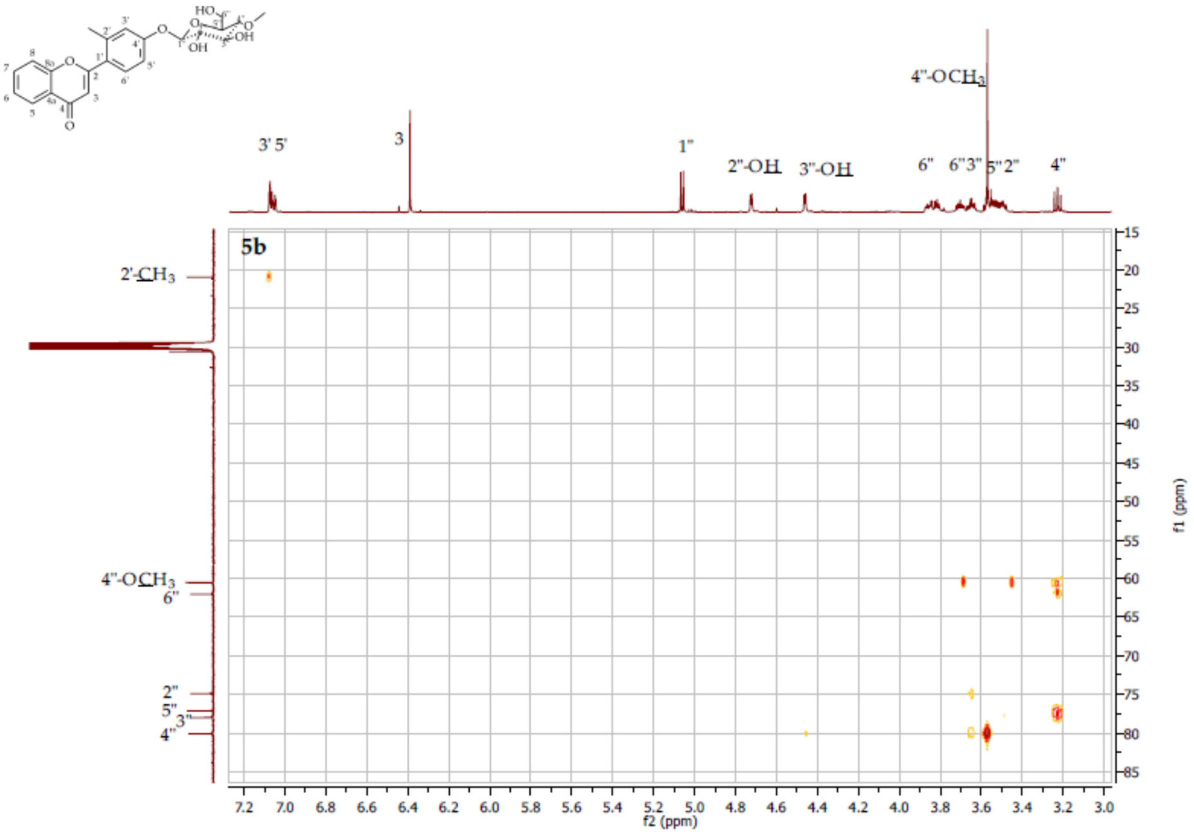

Figure S150. HMBC contour map – ^{1}H x ^{13}C expansion of 2′-methylflavone 4′-*O*-*β*-D-(4′′-*O*-methyl)-glucopyranoside (**5b**)

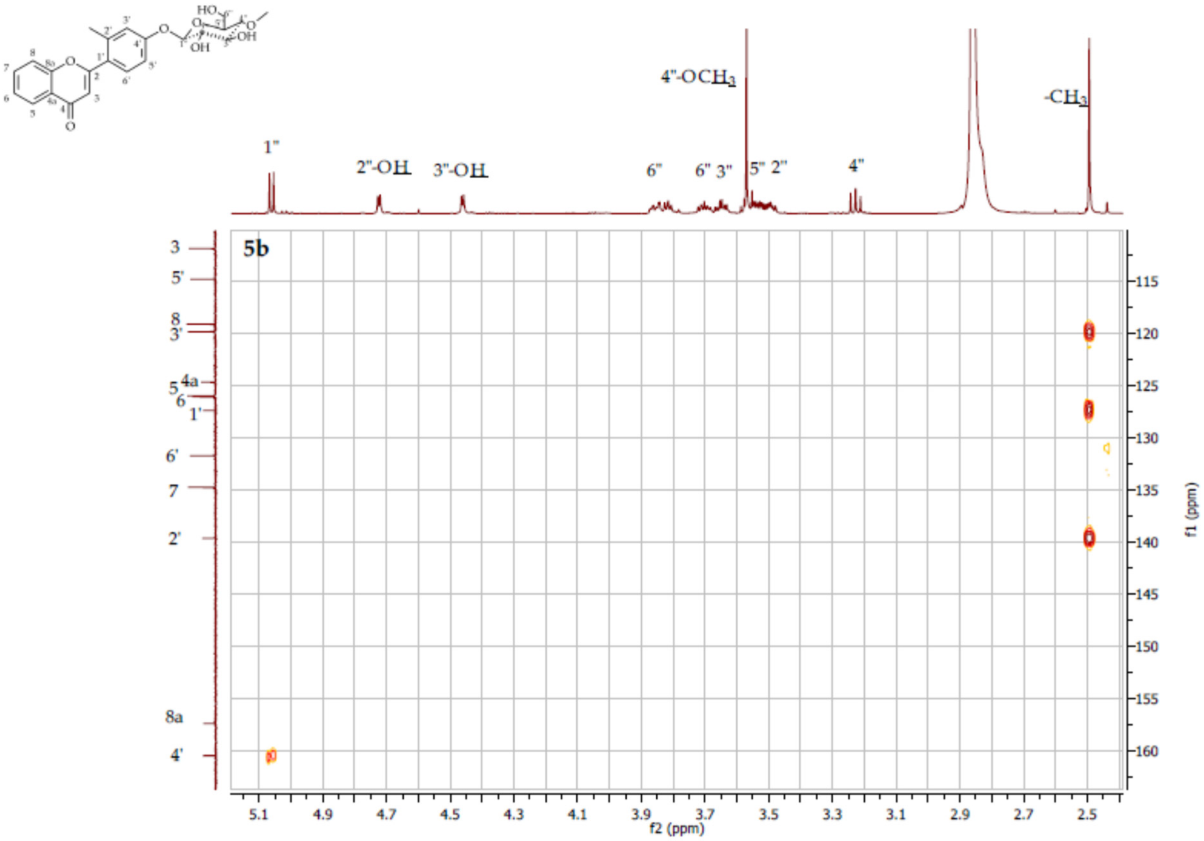

Figure S151. HMBC contour map – ^{1}H x ^{13}C expansion of 2′-methylflavone 4′-*O*-*β*-D-(4′′-*O*-methyl)-glucopyranoside (**5b**)

Molecular formula = $C_{23}H_{24}O_8$
Formula weight = 428.15
Ionization mode = positive
Precursor: $[M + H]^+$ 429.20
429.2000>253.0500 CE: -32.0
429.2000>294.9500 CE: -25.0
429.2000>121.0500 CE: -55.0

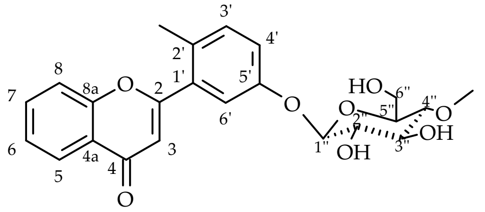

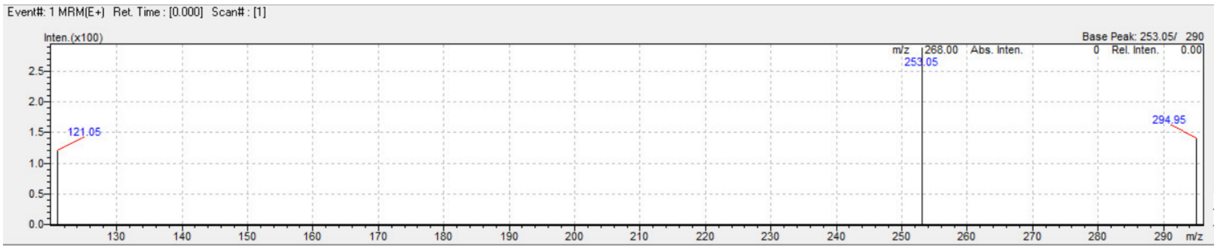

Figure S152. MS analysis of 2′-methylflavone 5′-*O*-*β*-D-(4′′-*O*-methyl)-glucopyranoside (**5c**)

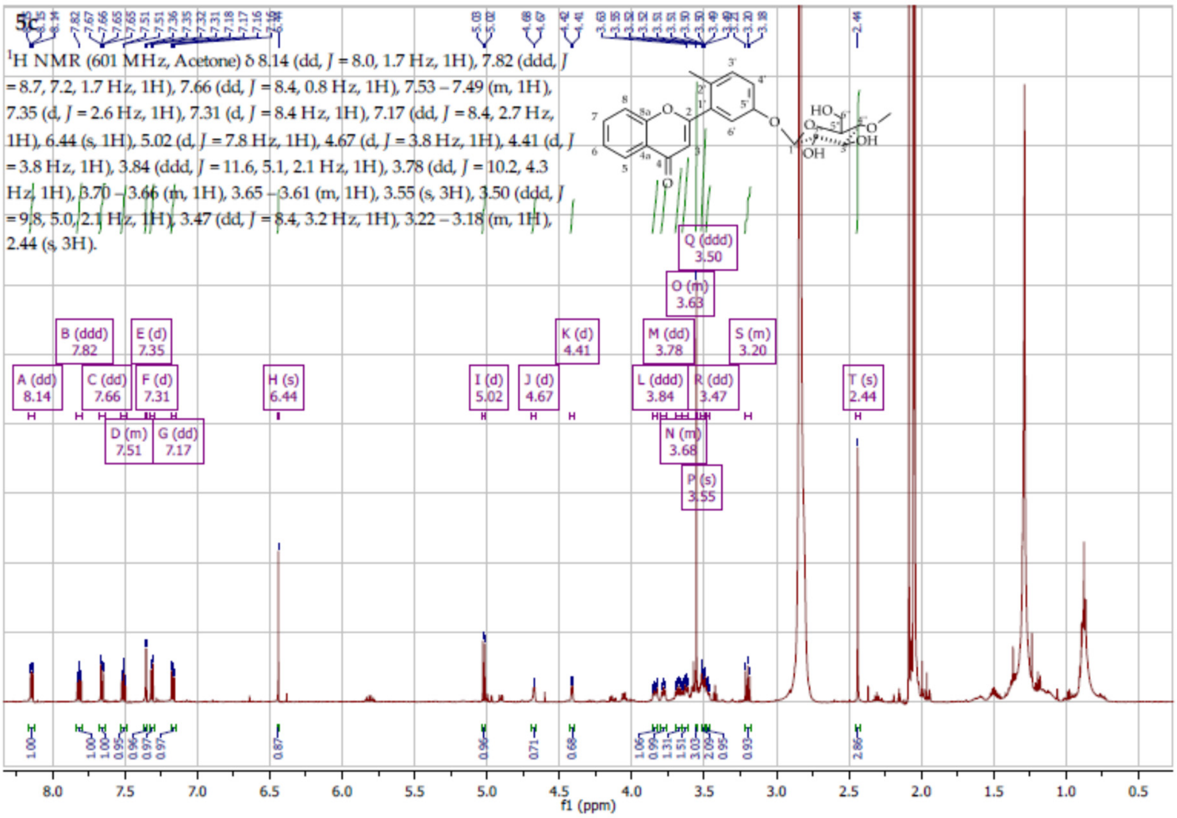

Figure S153. ¹H NMR spectrum (*δ,* acetone-d6, 600 MHz) of 2′-methylflavone 5′-*O*-*β*-D-(4′′-*O*-methyl)-glucopyranoside (**5c**)

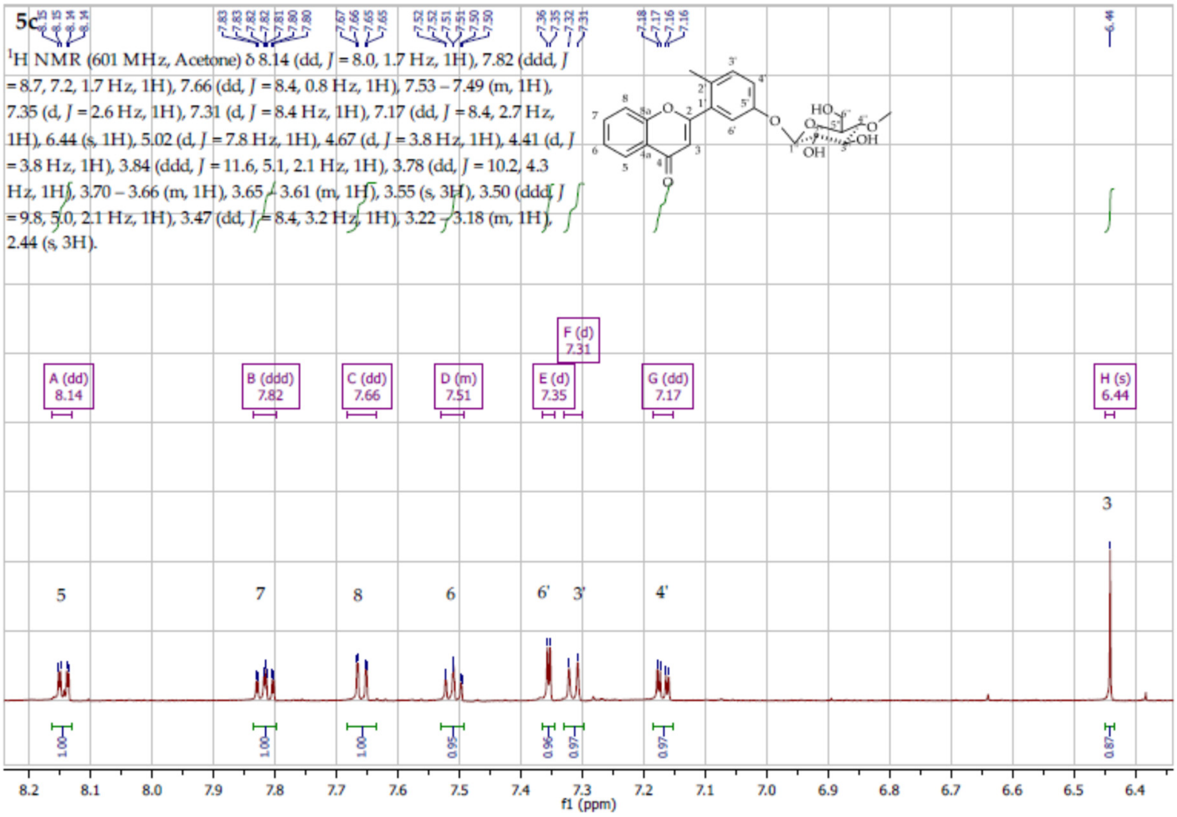

Figure S154. ¹H NMR spectrum expansion (*δ,* acetone-d6, 600 MHz) of 2′-methylflavone 5′-*O*-*β*-D-(4′′-*O*-methyl)-glucopyranoside (**5c**)

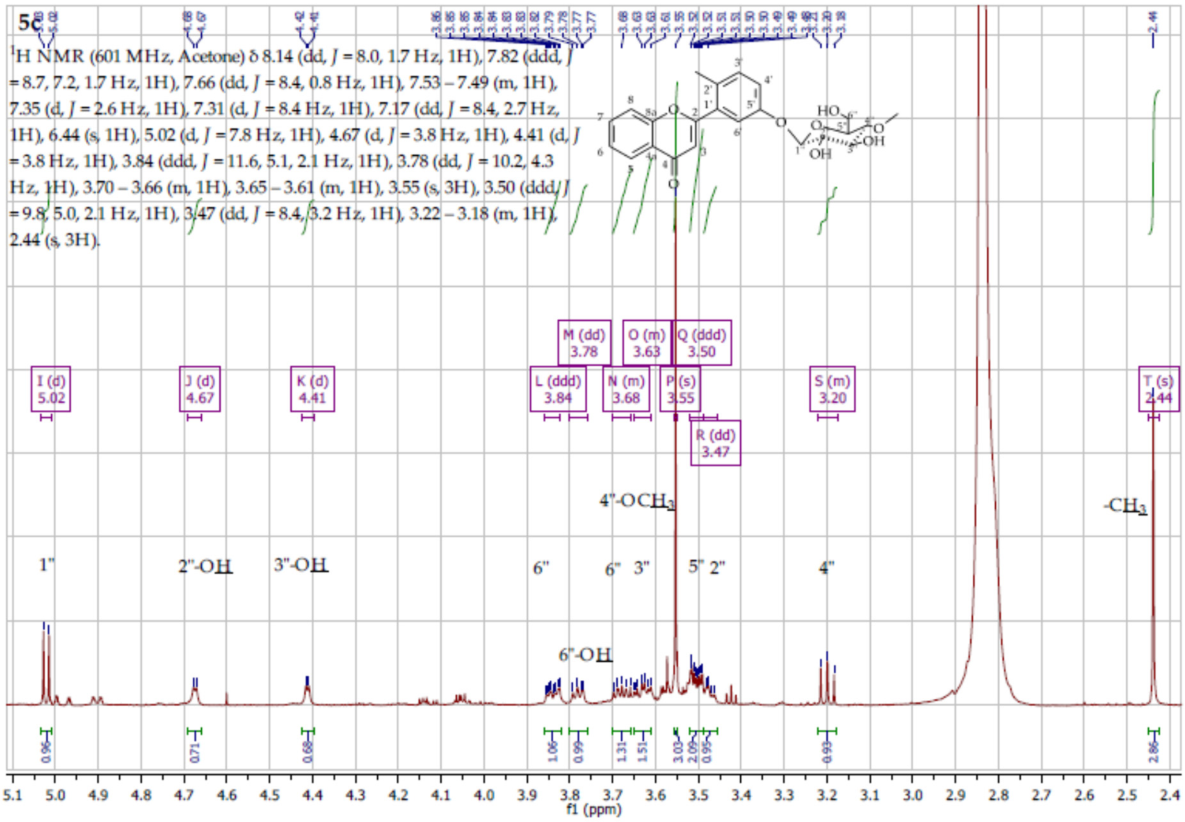

Figure S155. ^{1}H NMR spectrum expansion (*δ*, acetone-d6, 600 MHz) of 2′-methylflavone 5′-*O*-*β*-D-(4′′-*O*-methyl)-glucopyranoside (**5c**)

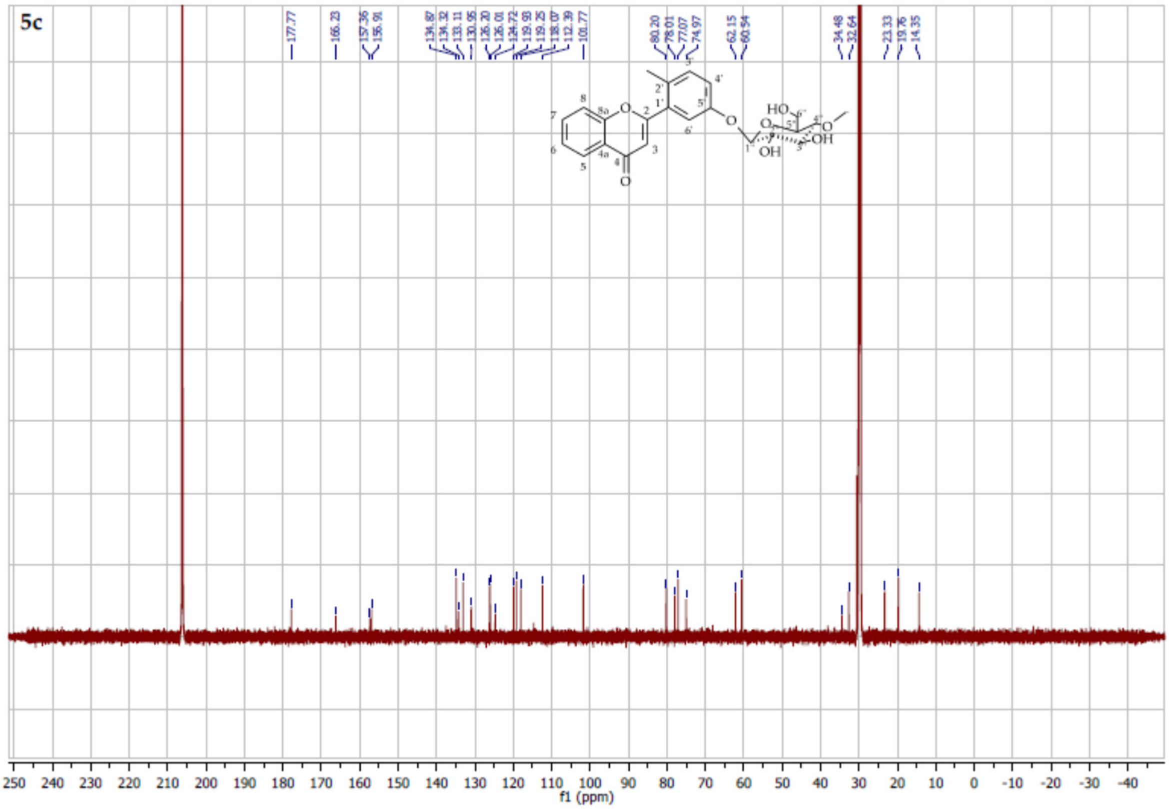

Figure S156. ^{13}C NMR spectrum (*δ*, acetone-d6, 151 MHz) of 2′-methylflavone 5′-*O*-*β*-D-(4′′-*O*-methyl)-glucopyranoside (**5c**)

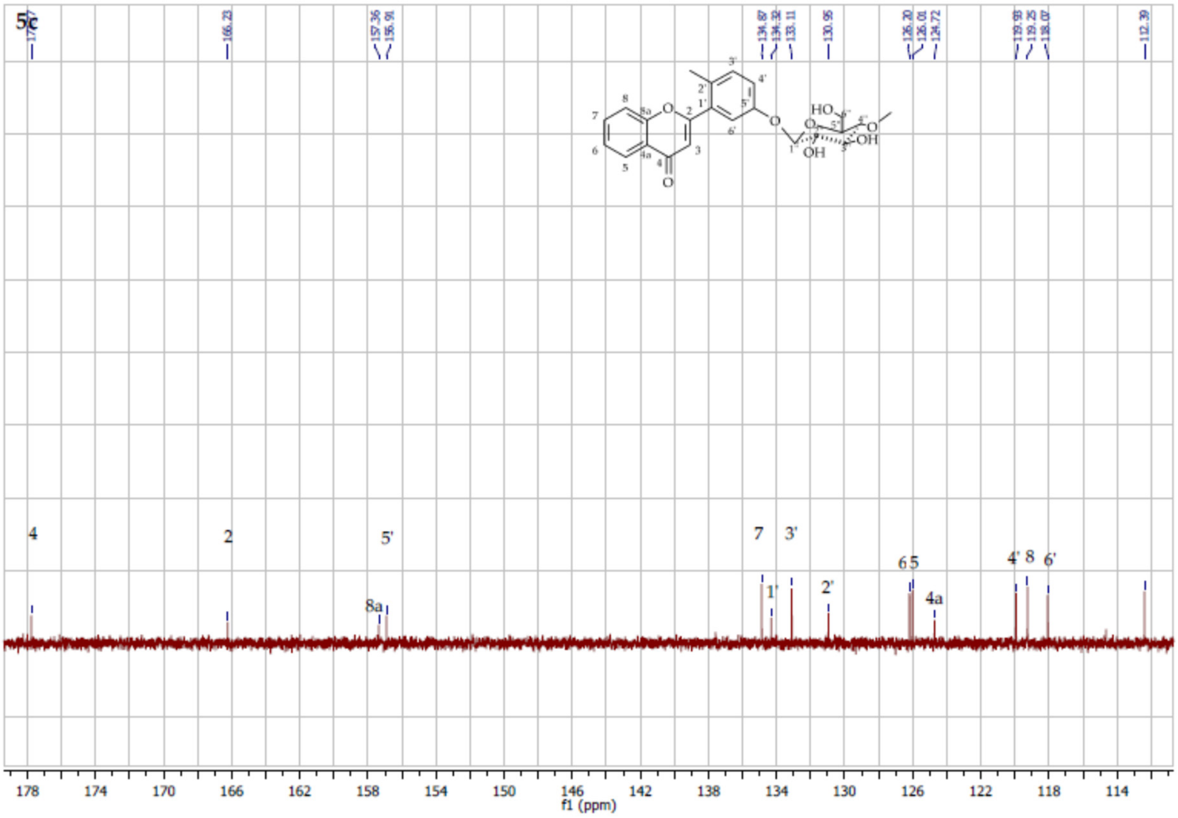

Figure S157. ^{13}C NMR spectrum expansion (δ, acetone-d6, 151 MHz) of 2′-methylflavone 5′-*O*-β-D-(4′′-*O*-methyl)-glucopyranoside (**5c**)

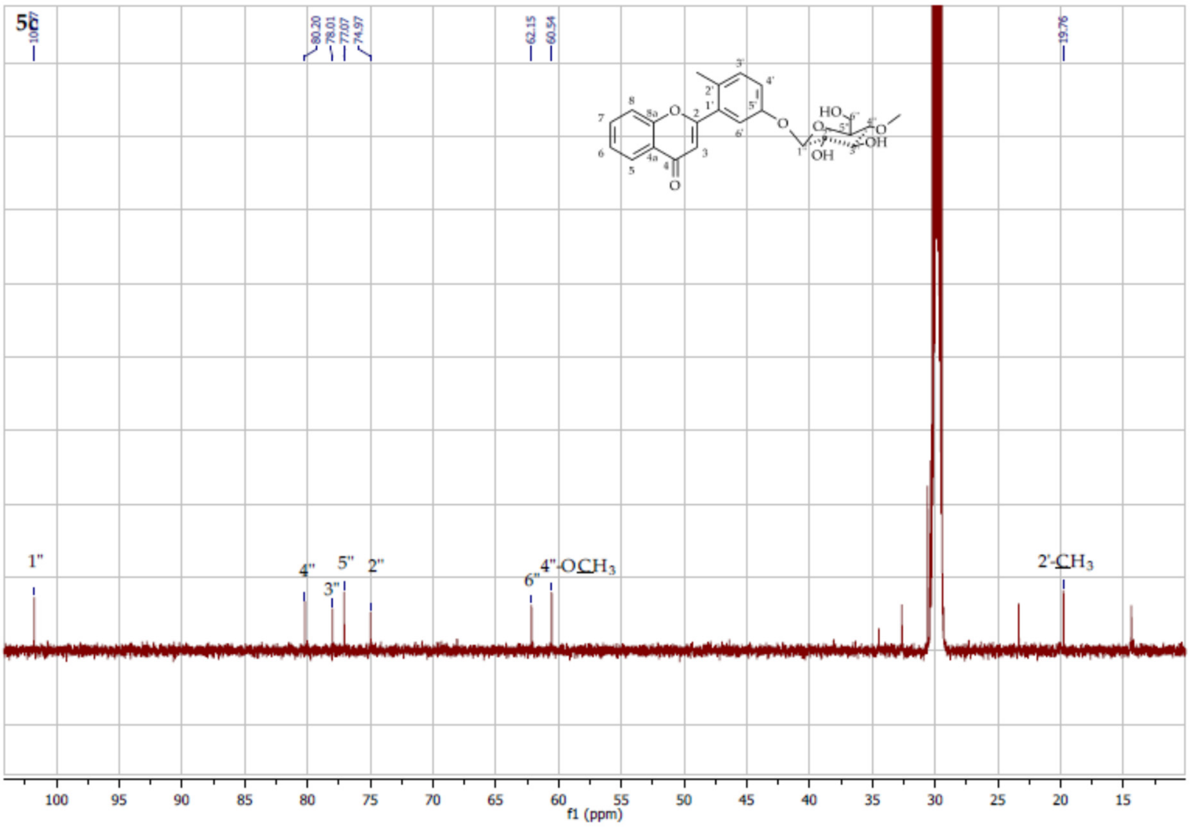

Figure S158. ^{13}C NMR spectrum expansion (δ, acetone-d6, 151 MHz) of 2′-methylflavone 5′-*O*-β-D-(4′′-*O*-methyl)-glucopyranoside (**5c**)

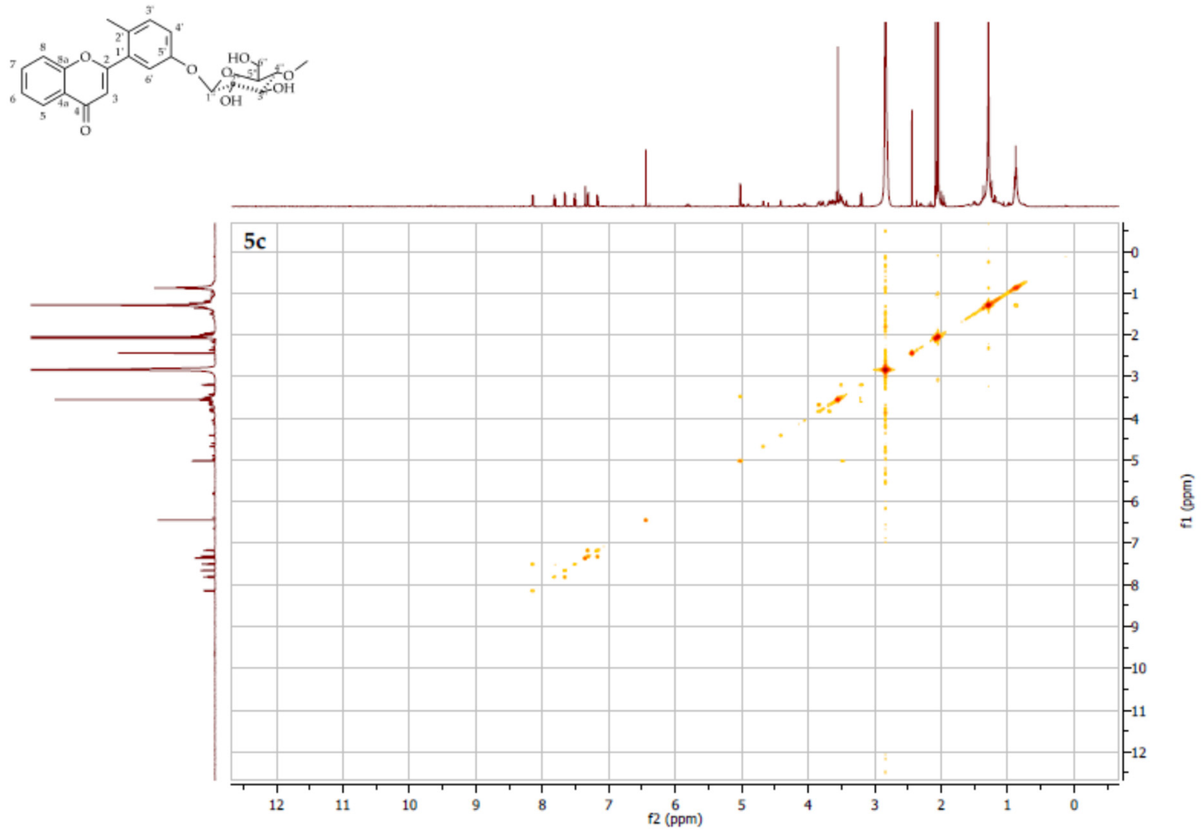

Figure S159. COSY contour map – ^{1}H x ^{1}H of 2′-methylflavone 5′-*O*-*β*-D-(4′′-*O*-methyl)-glucopyranoside (**5c**)

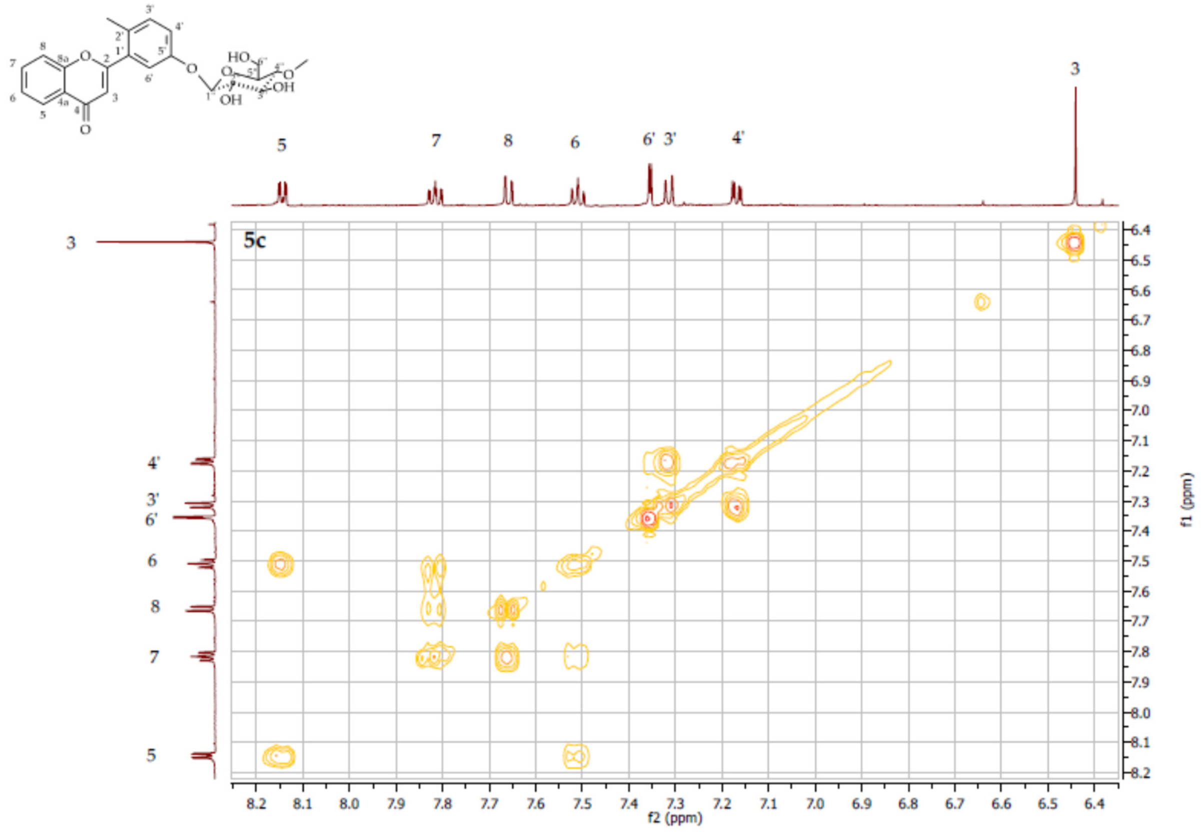

Figure S160. COSY contour map – ^{1}H x ^{1}H expansion of 2′-methylflavone 5′-*O*-*β*-D-(4′′-*O*-methyl)-glucopyranoside (**5c**)

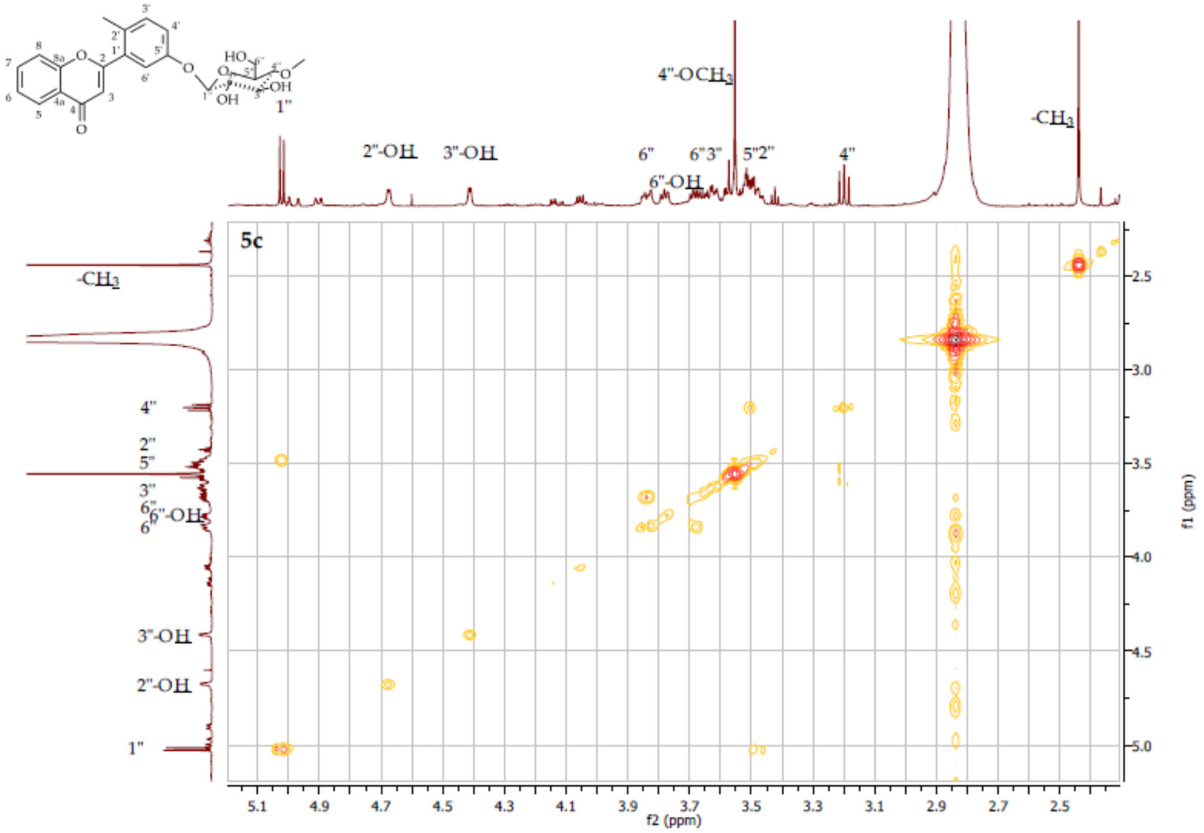

Figure S161. COSY contour map – ^{1}H x ^{1}H expansion of 2′-methylflavone 5′-*O*-*β*-D-(4′′-*O*-methyl)-glucopyranoside (**5c**)

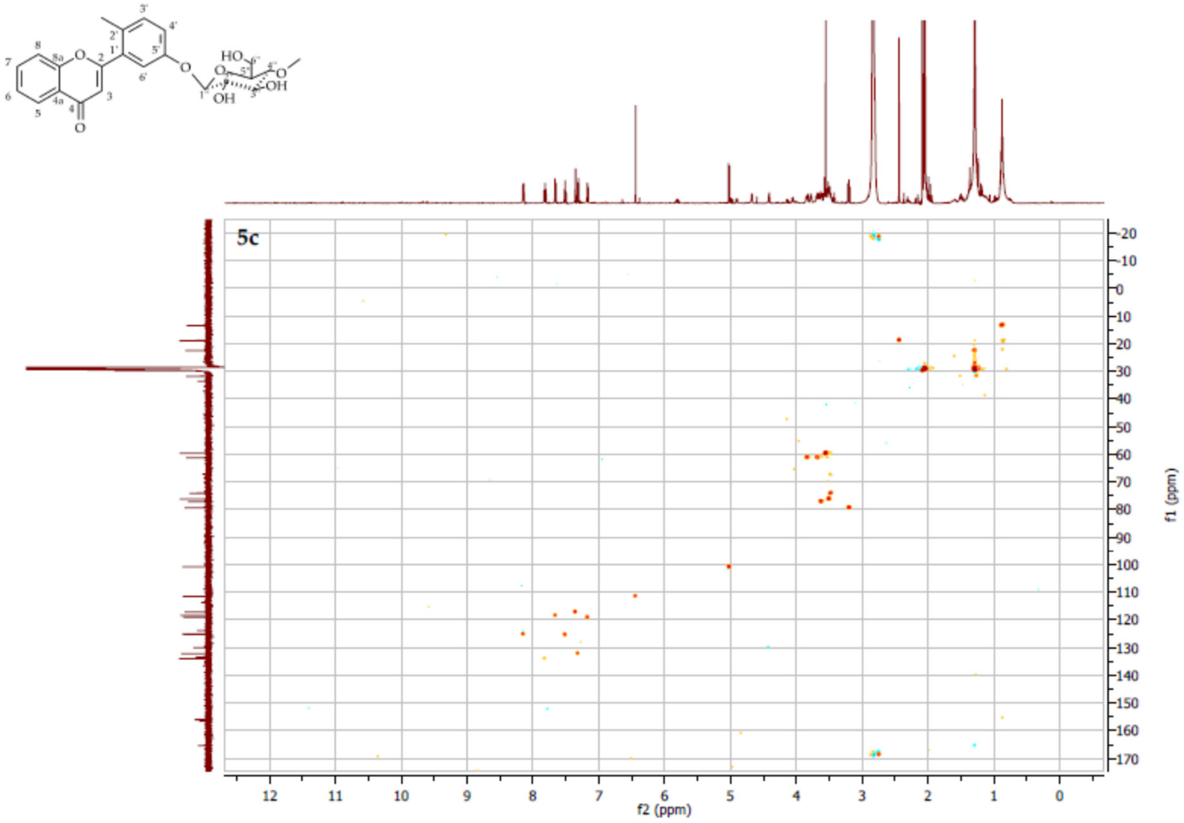

Figure S162. HSQC contour map – ^{1}H x ^{13}C of 2′-methylflavone 5′-*O*-*β*-D-(4′′-*O*-methyl)-glucopyranoside (**5c**)

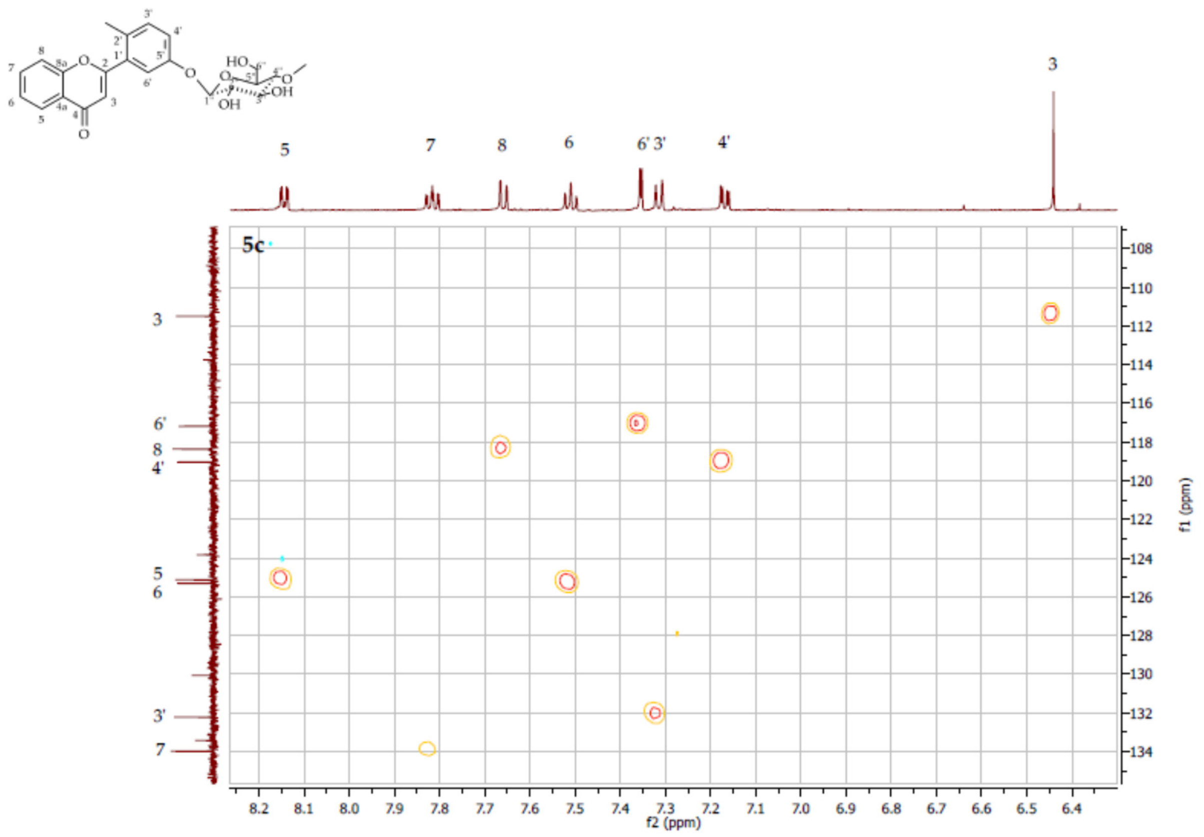

Figure S163. HSQC contour map – ^{1}H x ^{13}C expansion of 2′-methylflavone 5′-*O*-*β*-D-(4′′-*O*-methyl)-glucopyranoside (**5c**)

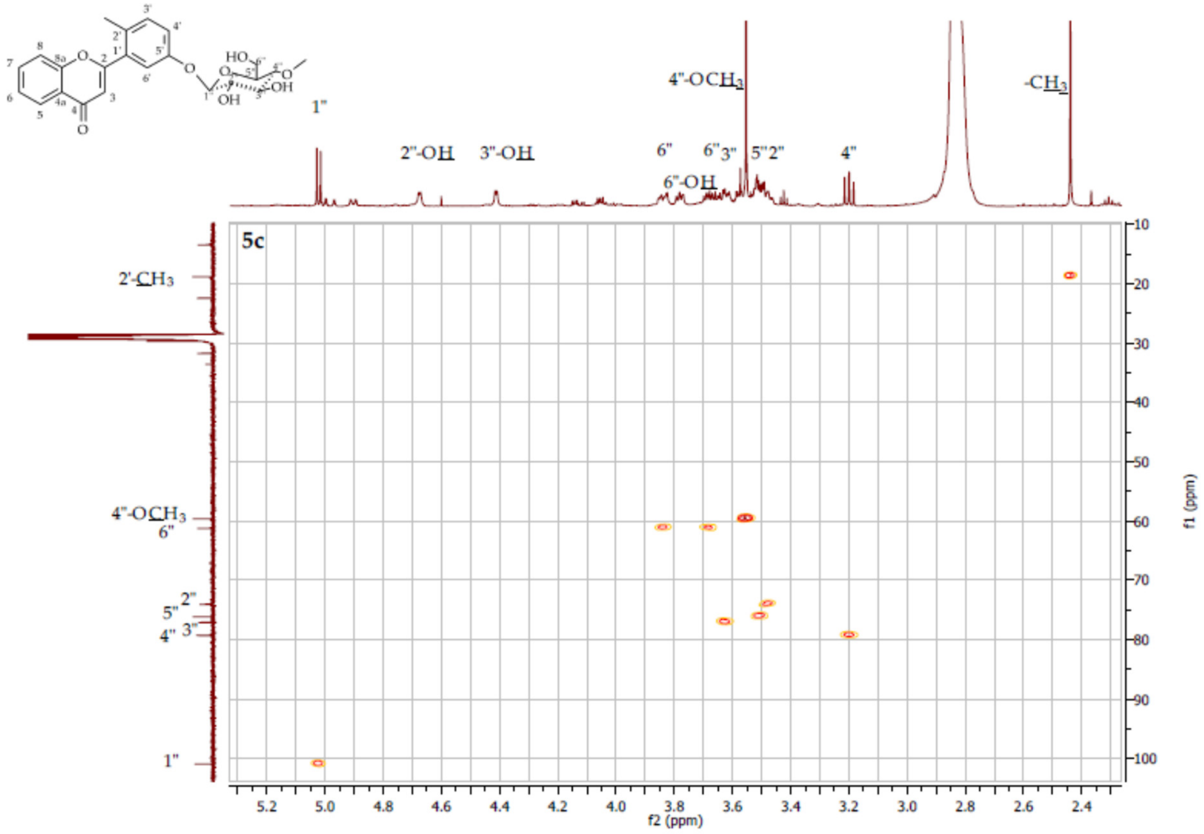

Figure S164. HSQC contour map – ^{1}H x ^{13}C expansion of 2′-methylflavone 5′-*O*-*β*-D-(4′′-*O*-methyl)-glucopyranoside (**5c**)

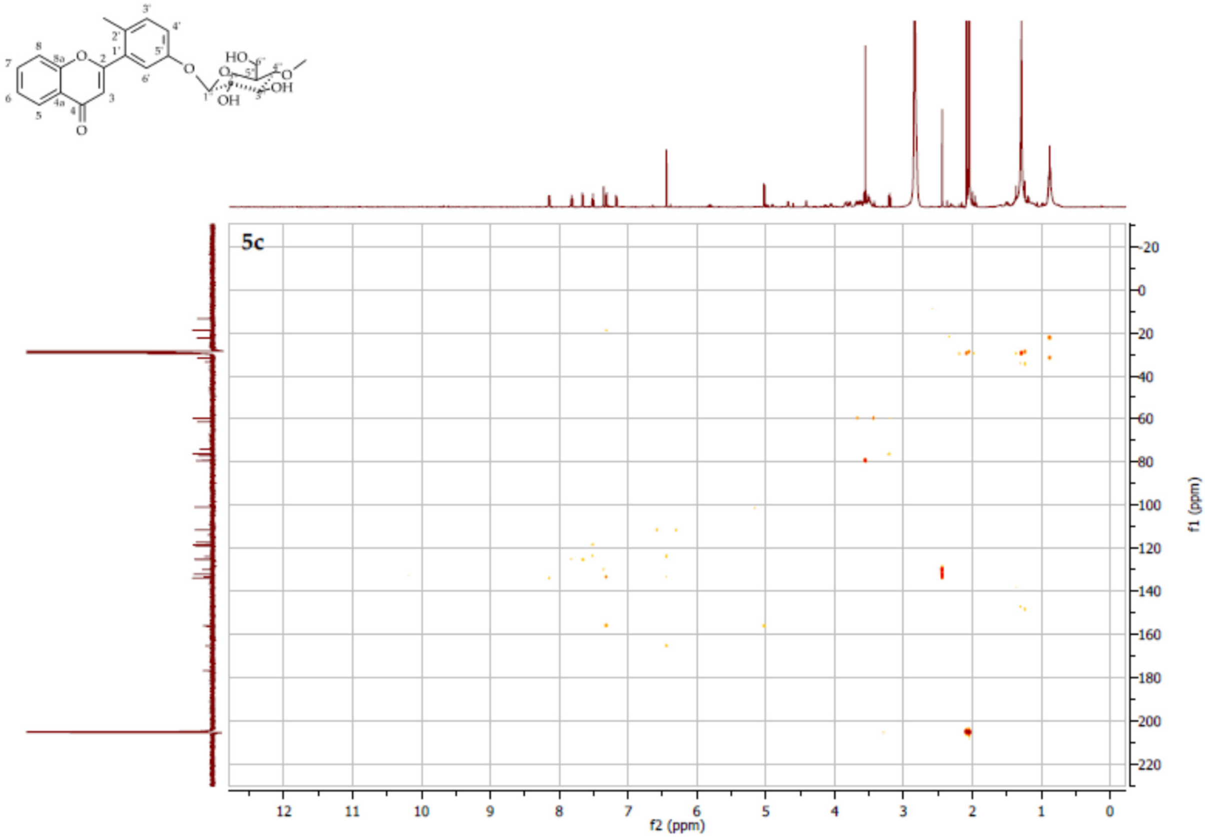

Figure S165. HMBC contour map – ^{1}H x ^{13}C of 2′-methylflavone 5′-*O*-*β*-D-(4′′-*O*-methyl)-glucopyranoside (**5c**)

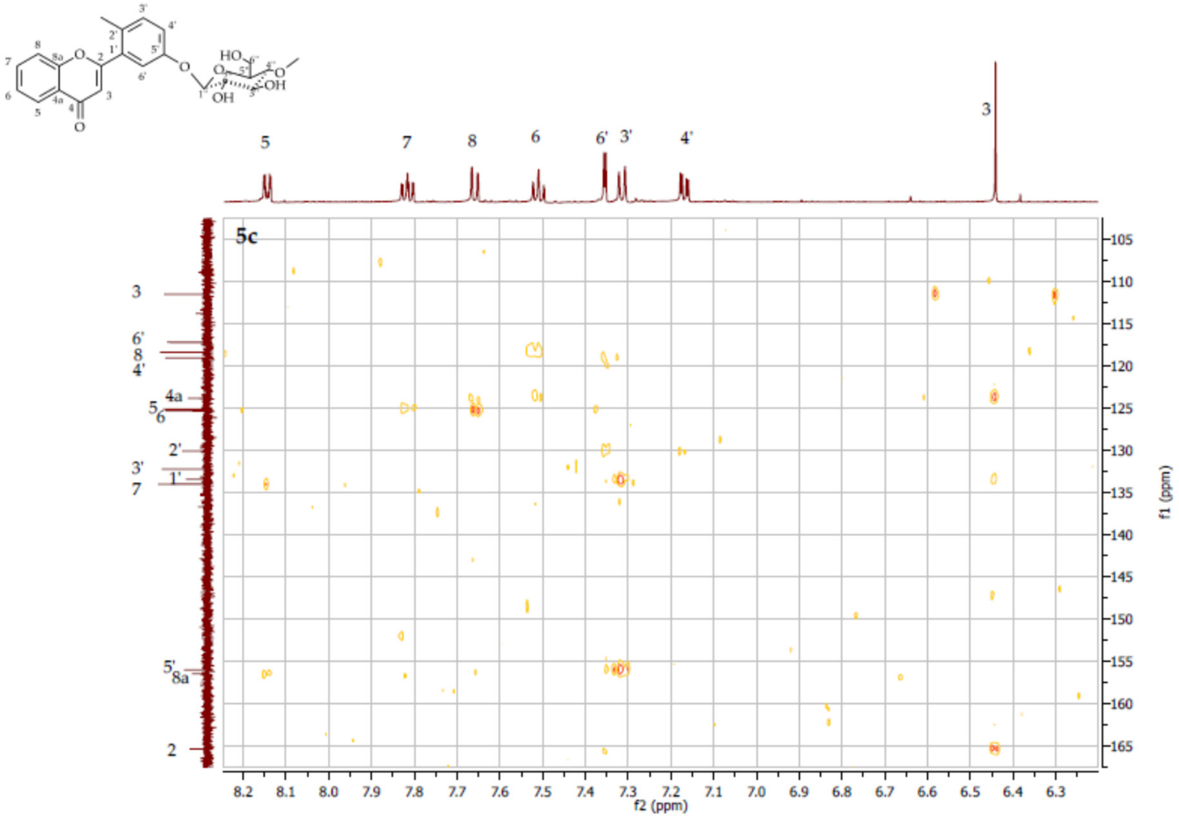

Figure S166. HMBC contour map – ^{1}H x ^{13}C expansion of 2′-methylflavone 5′-*O*-*β*-D-(4′′-*O*-methyl)-glucopyranoside (**5c**)

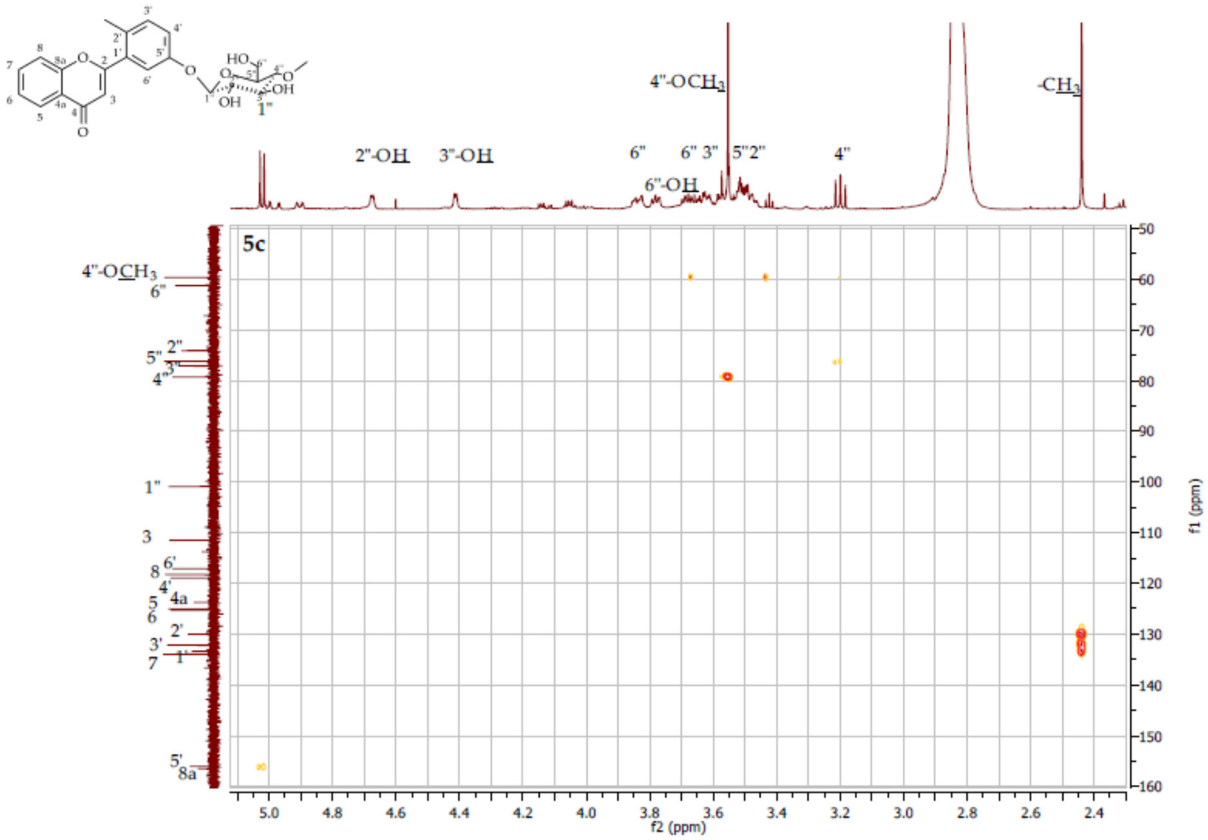

Figure S167. HMBC contour map – ^{1}H x ^{13}C expansion of 2′-methylflavone 5′-*O*-*β*-D-(4′′-*O*-methyl)-glucopyranoside (**5c**)
